# Imidazolidine Hydride Donors in Palladium-Catalyzed Alkyne Hydroarylation

**DOI:** 10.1021/acs.joc.2c00725

**Published:** 2022-06-01

**Authors:** Soe L. Tun, S. V. Santhana Mariappan, F. Christopher Pigge

**Affiliations:** † Department of Chemistry, University of Iowa, Iowa City, Iowa 52242, United States; ‡ Central NMR Facility, University of Iowa, Iowa City, Iowa 52242, United States

## Abstract

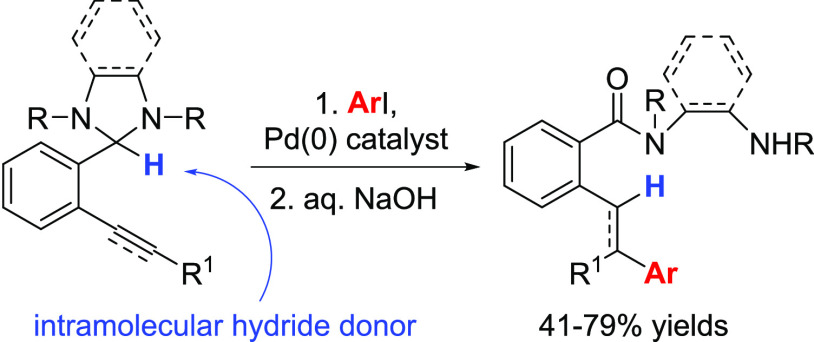

Aldehyde-derived imidazolidines participate as hydride donors in intramolecular reductive Heck-type reactions. *N*,*N*′-Diphenylimidazolidines prepared from *ortho*-alkynyl benzaldehydes underwent regio- and stereoselective palladium-catalyzed hydroarylation followed by formal 1,5-hydride transfer and reductive elimination to afford substituted alkenes and imidazolium moieties, the latter conveniently converted in situ to ring-opened benzanilides to simplify product isolation. Internal alkynes were converted to trisubstituted alkenes via a *syn* hydroarylation process, while a terminal alkyne was converted to a *cis* alkene via a formal *trans* hydroarylation reaction. Benzanilide products could be converted to carboxylic acid derivatives under basic conditions, resulting in the net conversion of alkynyl aldehydes to alkenyl carboxylic acids. A styrene derivative with an attached *N*,*N*′-dimethylbenzimidazoline hydride donor was also found to undergo an analogous hydroarylation/benzimidazoline oxidation to give a diarylethane product.

## Introduction

**Scheme 1:**
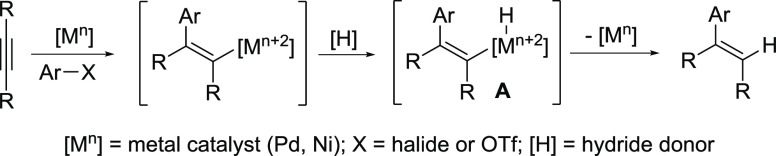
Mechanistic Overview of Alkyne Hydroarylation under Reductive Heck Conditions

The hydroarylation of alkynes and alkenes (reductive Heck-type reactions) is an important transformation in organic synthesis. The sequential formation of C–C and C–H bonds at the expense of C–C π bonds often occurs stereoselectively when performed in the presence of appropriate transition metal catalysts, and developing new tactics to achieve hydrocarbonation continues to garner considerable interest from the organic research community.^1,2^ A key feature of hydroarylation reactions that proceed through M^*n*^/M^*n*+2^ catalytic cycles is the in situ generation of metal–hydride intermediates in advance of C–H reductive elimination (**A**, Scheme 1).^3^ This typically requires use of stoichiometric additives as hydride sources. Common hydride donors include various salts of formic acid,^4−7^ organosilanes,^8^ alcohols and hemiacetals,^9−14^ tertiary amines,^15^ and even H_2_O when activated by boron-based Lewis acids.^16^

**Scheme 2:**
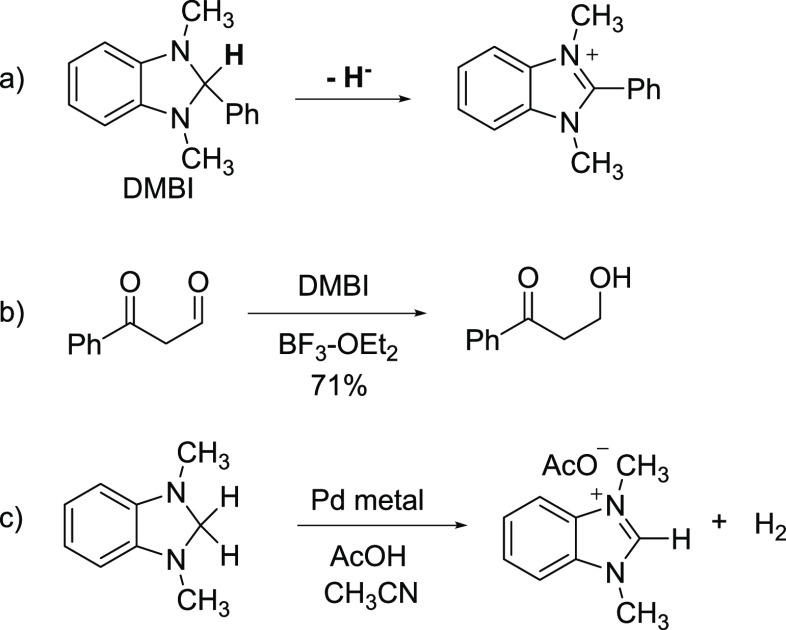
Benzimidazolines as Hydride Sources

Imidazolidines (cyclic aminals) derived from the condensation of aldehydes and 1,2-diamines along with related benzimidazolines have emerged as neutral organic hydride donors in a number of settings.^17−20^ For example, 1,3-dimethyl-2-phenylbenzimidazoline (DMBI) exhibits a hydride-donating ability comparable to cyanoborohydride (Scheme 2a).^21,22^ Accordingly, DMBI has been employed as a formal hydride-reducing agent toward various organic functional groups, such as α-halocarbonyls and β-ketoaldehydes (Scheme 2b).^23,24^ Notably, in some instance, these reactions appear to proceed via initial single-electron transfer (SET).^25^ Similarly, benzothiazolines have been utilized as hydride donors toward organic electrophiles (e.g., enones) in the presence of Lewis or Brønsted acids.^26,27^ Applications of imidazolidines and thiazolidines as reductants in photocatalytic transformations have been reported as well.^28,29^

Exploiting aminals and related heterocycles as formal hydride donors in transition metal-catalyzed reactions, however, has not been extensively explored. A few studies have examined the C–H activation of imidazolidines by late transition metal complexes (Rh and Ir) as a means of generating metal hydrides and, ultimately, *N*-heterocyclic carbene–metal complexes.^30,31^ Additionally, the oxidation of imidazolidines in the presence of catalytic amounts of Pd(II) complexes has been reported.^32^ Interestingly, DMBI and analogues have been advanced as potential components of chemical hydrogen storage devices owing to facile H_2_ evolution when combined with protic acid and a Pd catalyst (Scheme 2c).^33^ As synthetic applications of aminal reductants in concert with transition metal catalysis are underdeveloped, we became intrigued by the possibility of using simple imidazolidine or benzimidazoline derivatives as hydride donors in metal-mediated reductive Heck-type reactions. Successful harnessing of cyclic aminals as hydride donors in metal-catalyzed transformations may then result in novel approaches to reductive C–C coupling and demonstrate new applications of imidazolidine heterocycles in organometallic chemistry. We report here the results of our initial investigations in which imidazolidine heterocycles conveniently prepared from benzaldehyde derivatives serve as intramolecular hydride donors in Pd-catalyzed alkyne hydroarylation reactions.

## Results and Discussion

**Scheme 3:**
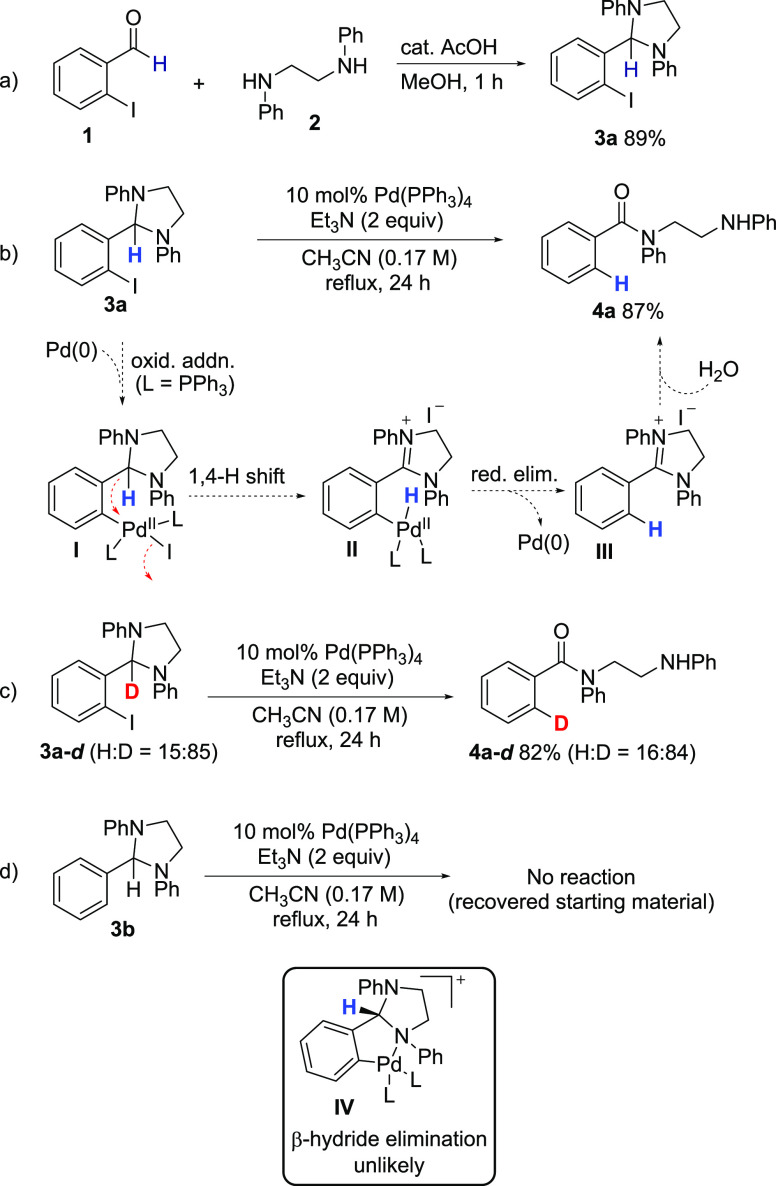
Reductive Dehalogenation of a 2-Iodophenyl Imidazolidine

At the outset, we reasoned that aminals prepared from 2-alkynylbenzaldehyde derivatives would be suitable substrates on which to test the feasibility of intramolecular imidazolidine hydride donation in metal-catalyzed reductive coupling. As a prelude to these studies, we first examined the reactivity of an aminal prepared from 2-iodobenzaldehyde toward reductive dehalogenation. The aminal **3a** was easily prepared in good yield upon reaction of **1** with *N*,*N*′-diphenylethylene diamine **2** as shown in Scheme 3a. We reasoned that a diphenyl-substituted imidazolidine would exhibit attenuated Lewis basicity and metal-ligating ability while retaining its hydride donor ability. We envisioned that reaction of **3a** with a Pd(0) complex would give (aryl)Pd(II) intermediate **I**, from which a Pd-H intermediate **II** could be generated via imidazolidine hydride transfer (Scheme 3b). Reductive elimination would then produce an imidazoline benzoic acid equivalent (**III**) and regenerate Pd(0). In the event, Pd-catalyzed reduction of aryl iodide did in fact take place upon treatment with Pd(PPh_3_)_4_ and Et_3_N with concomitant oxidation of the imidazolidine, and the ring-opened benzanilide **4a** was isolated in 87% yield (we attribute ring-opening of the putative imidazolinium salt to the presence of adventitious water). A deuterium labeling study was performed to confirm the imidazolidine as the hydride source in this reductive dehalogenation. Thus, exposure of **3a**-***d*** to Pd(PPh_3_)_4_ resulted in virtually complete deuterium transfer to the aryl position (Scheme 3c). While we do not know the mechanism by which presumed Pd–H intermediate **II** is formed, we observed no reaction between imidazolidine **3b** and Pd(PPh_3_)_4_, indicating that hydride transfer occurs after Pd(0) oxidative addition (Scheme 3d). Additionally, Pd–H formation from β-hydride elimination involving the aminal hydrogen of an in situ generated palladacycle such as **IV** seems unlikely due to geometrical constraints.^32^ Consequently, we show Pd–H formation via formal 1,4-hydride shift in Scheme 3b,^34,35^ although other possibilities exist (e.g., SET/H-atom transfer).

**Scheme 4:**
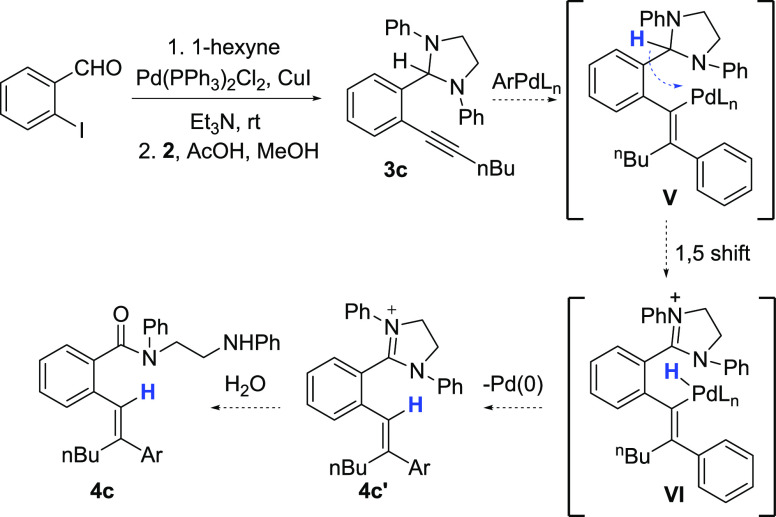
Preparation of Alkynyl Aminal **3c** and Postulated Hydroarylation Sequence

With the feasibility of metal-mediated imidazolidine hydride transfer established, we next sought to extend this reactivity profile to include participation in intramolecular alkyne hydroarylation. Sonogashira coupling between 2-iodobenzaldehyde and 1-hexyne followed by aminal formation afforded initial hydroarylation substrate **3c** (Scheme 4). We anticipated that **3c** would undergo regio- and stereoselective carbometallation upon treatment with an aryl halide and a Pd(0) catalyst. Interception of the resulting (alkenyl)Pd(II) intermediate **V** by formal 1,5-hydride shift would give Pd(II)-hydride intermediate **VI**, and reductive elimination would then afford the expected hydroarylation product **4c′** and/or **4c** depending on the facility of imidazolinium ring opening under the reaction conditions. To simplify product isolation in favor of **4c**, aqueous NaOH solution was added to crude reaction mixtures after complete consumption of the starting alkynyl aminal (as determined by TLC).

**Figure 1:**
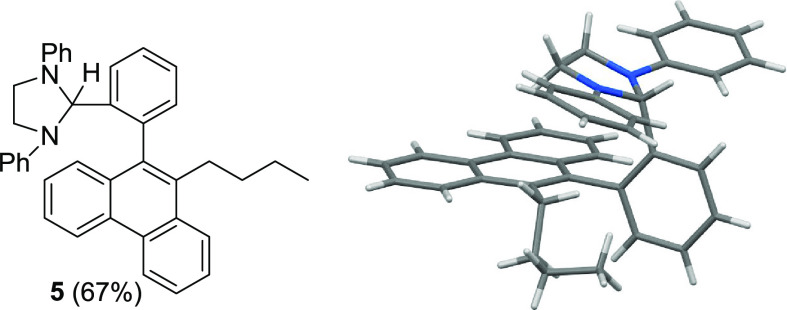
Line drawing and X-ray crystal structure of phenanthrene **5**.

**Scheme 5:**
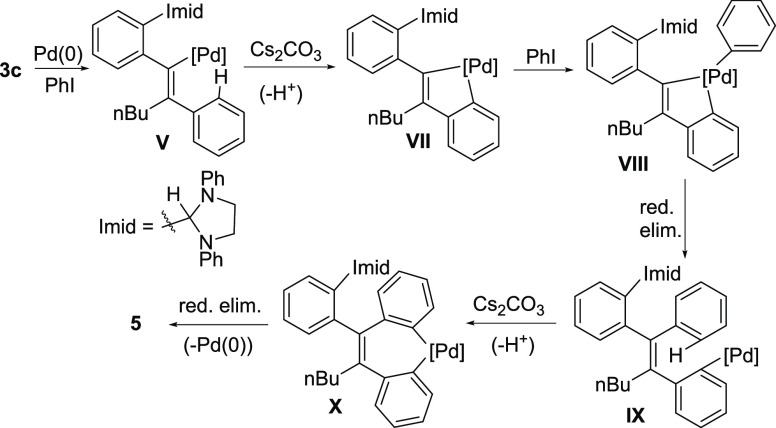
Plausible Sequence Leading to **5**

Gratifyingly, exposure of **3c** to iodobenzene (4 equiv) in the presence of Pd(PPh_3_)_4_ and Et_3_N in CH_3_CN followed by addition of aq. NaOH gave the desired hydroarylation product **4c** as a single-alkene isomer in good isolated yield (Table 1, entries 1 and 2). Combinations of other palladium sources, ligands, and solvents (DMF, 1,4-dioxane) also were effective to varying degrees (Table 1) with the exception of 1,2-dichloroethane (DCE, entry 5). Notably, the reaction was successful under ligand-free conditions using Pd(OAc)_2_ as the catalyst (Table 1, entry 8). Omitting Et_3_N from the reaction, however, resulted in sluggish transformation and lower isolated yields (Table 1, entries 9, 15). According the mechanistic rationale illustrated in Scheme 4 (vide supra), the presence of base does not appear to be necessary for the transformation, and so, we speculate that Et_3_N exerts a beneficial effect by preventing aminal hydrolysis. Indeed, generation of free diamine **2** was observed (TLC) in reactions performed in the absence of base. Other bases, such as DBU (Table 1, entry 17) and Cs_2_CO_3_ (Table 1, entry 18), were ineffective in promoting hydroarylation. In the reaction with Cs_2_CO_3_, however, the substituted phenanthrene **5** was obtained (67% isolated yield) with an intact imidazolidine group (structure confirmed by X-ray diffractometry; Figure 1). The formation of **5** is attributed to the sequential coupling of two aryl iodide reactants with the aryl acetylene, in line with similar Pd-catalyzed routes to phenanthrenes that have been previously reported.^36^ One plausible mechanistic rationale along the lines of the Catellani reaction^37,38^ is outlined in Scheme 5 and entails initial carbometallation of **3c** to give **V**, which then undergoes aryl C–H activation in the presence of Cs_2_CO_3_ in lieu of hydride transfer. The resulting palladacycle **VII** reacts with another molecule of iodobenzene to give Pd(IV) intermediate **VIII**. Reductive elimination, a second C–H activation, and a final reductive elimination then afford **5** while regenerating Pd(0).

**Table 1:**
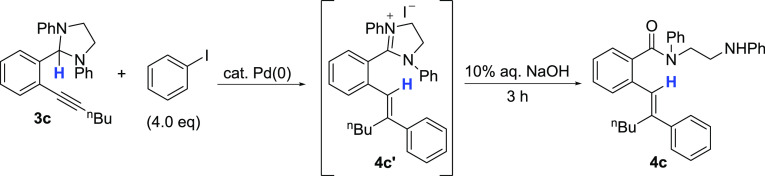
Survey of Reaction Conditions for Pd-Catalyzed Hydroarylation of Imidazolidine Alkynes^a^

entry	Pd catalyst (mol %)	ligand (mol %)	base^b^	solvent	time (h)^c^	yield (%)^d^
1	Pd(PPh_3_)_4_ (5)	none	Et_3_N	MeCN	36	65
2	Pd(PPh_3_)_4_ (10)	none	Et_3_N	MeCN	20	71
3	Pd(PPh_3_)_4_ (10)	none	Et_3_N	1,4-dioxane	18	76
4	Pd(PPh_3_)_4_ (10)	none	Et_3_N	DMF	18	71
5	Pd(PPh_3_)_4_ (10)	none	Et_3_N	DCE	72^e^	trace
6	Pd_2_dba_3_ (5)	PPh_3_ (20)	Et_3_N	1,4-dioxane	23	62
7	Pd_2_dba_3_ (10)	PPh_3_ (40)	Et_3_N	1,4-dioxane	23	70
8	Pd(OAc)_2_ (10)	none	Et_3_N	1,4-dioxane	27	63
9	Pd(OAc)_2_ (10)	none	none	1,4-dioxane	27	27
10	Pd(OAc)_2_ (10)	P(o-Tol)_3_ (40)	Et_3_N	1,4-dioxane	22	46
11	Pd(OAc)_2_ (10)	PCy_3_ (40)	Et_3_N	1,4-dioxane	22	68
12	Pd(OAc)_2_ (10)	PPh_3_ (20)	Et_3_N	1,4-dioxane	24	71
13	Pd(OAc)_2_ (10)	dppe (20)	Et_3_N	1,4-dioxane	27	66
14	Pd(OAc)_2_ (10)	dppf (11)	Et_3_N	1,4-dioxane	22	70
15	Pd(OAc)_2_ (10)	dppf (11)	none	1,4-dioxane	27	51
16	Pd(OAc)_2_ (10)	dppf (11)	DIPEA	1,4-dioxane	22	63
17	Pd(OAc)_2_ (10)	dppf (11)	DBU	1,4-dioxane	72^e^	12
18	Pd(OAc)_2_ (10)	dppf (11)	Cs_2_CO_3_	1,4-dioxane	16	22^f^
19^g^	Pd(OAc)_2_ (10)	dppf (11)	Et_3_N	1,4-dioxane	22	79

a Reactions performed using 0.4 mmol of **3c** in solvent at 80 °C with [**3c**] = 0.4–0.6 M for the indicated time. Ten percent aq. NaOH solution was then added (5 mL) with continued heating for 3 h to ensure conversion to **4c**.

b Two equivalents unless otherwise noted.

c Time for consumption of **3c** according to TLC.

d Isolated yield of **4c** after purification by flash column chromatography.

e Reaction stopped after 72 h.

f Phenanthrene **5** obtained as the major product.

g A total of 2.3 equiv of PhI used.

Our optimized conditions for conversion of **3c** to **4c** are shown in Table 1 (entry 19) and feature a catalyst prepared in situ from 10 mol % Pd(OAc)_2_, 11 mol % diphenylphosphinoferrocene (dppf) and 3.5 equiv of Et_3_N in 1,4-dioxane followed by addition of 10% aq. NaOH. Under these conditions, the amount of iodobenzene could be reduced to 2.3 equiv, and **4c** was obtained in 79% isolated yield.

**Figure 2:**
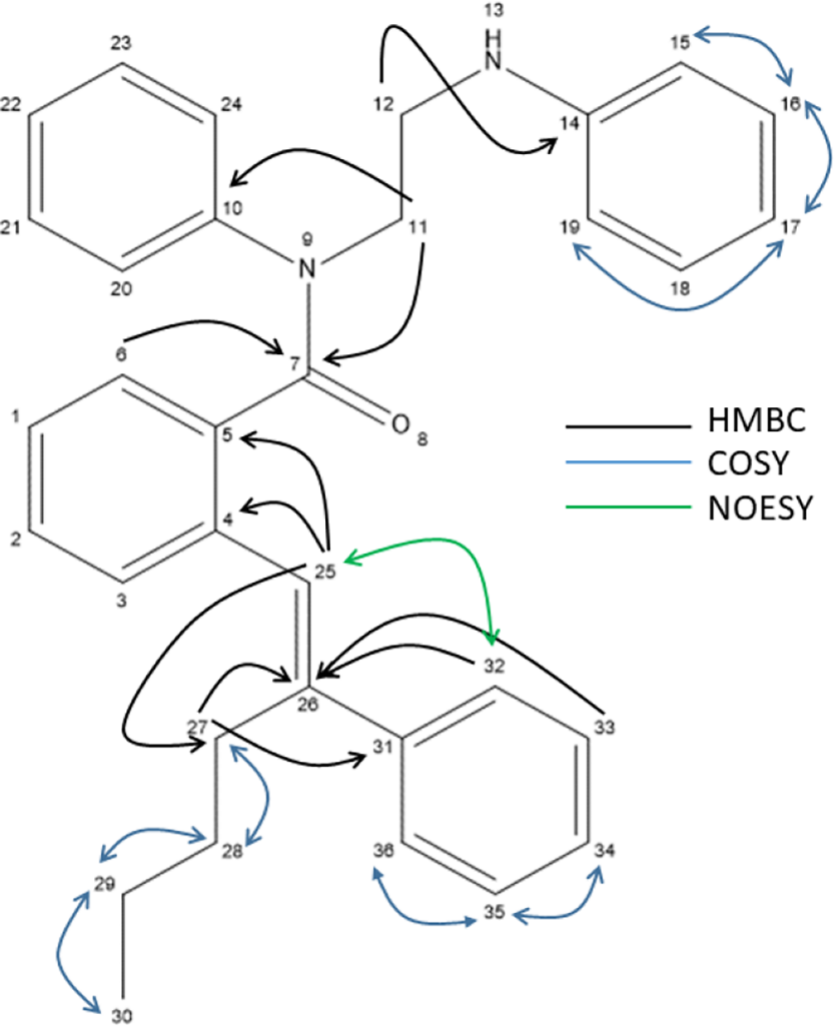
Partial atom connectivity map of **4c** derived from 2D NMR data.

The structure of **4c** was established on the basis of extensive 2D NMR spectroscopy experiments (see Figure 2 and the Supporting Information). Briefly, 1D and 2D ^1^H, ^13^C, and homonuclear and heteronuclear correlation data through scalar couplings allowed the resonance assignments of ^1^H and ^13^C signals, whereas ^1^H–^1^H NOESY data allowed the mapping of through-space interactions. The anticipated *E*-olefin geometry was confirmed from NOE correlations between alkene hydrogen H25 and the aromatic hydrogens H32/H36 as well as the absence of any correlation between H25 and the H27 methylene hydrogens in the butyl chain.

**Scheme 6:**
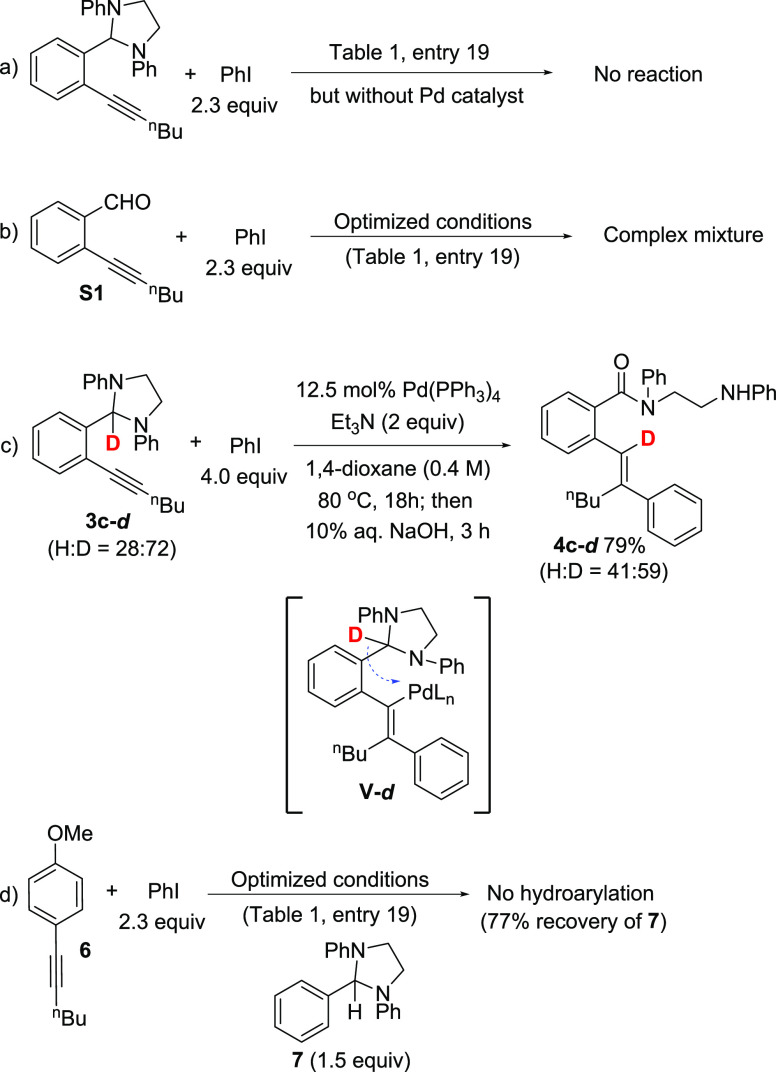
Control Experiments

Several control experiments were performed to gain further insight into the hydroarylation process. Not surprisingly, a Pd catalyst is essential for reaction as exposure of **3c** to optimized reaction conditions in the absence of Pd resulted in no reaction (Scheme 6a). The imidazolidine moiety is also crucial for conversion as treatment of alkynyl aldehyde **S1** with iodobenzene under optimized hydroarylation conditions returned a complex mixture with no evidence of hydroarylation by ^1^H NMR (Scheme 6b). Finally, the reaction of deuterated alkynyl imidazolidine **3c-*d*** and iodobenzene in the presence of Pd(PPh_3_)_4_ and Et_3_N gave the expected hydroarylation product with transfer of the deuterium to the vinylic position (Scheme 6c). This is consistent with the initial *syn* carbopalladation of the alkyne and interception of (alkenyl)Pd(II) intermediate **V-*d*** by formal intramolecular 1,5-hydride transfer from the imidazolidine.^34,35^ At this time, it is unclear whether imidazolidine coordination to the Pd(II) center plays a role in this process; however, the attempted hydroarylation of alkyne **6** in the presence of added imidazolidine **7** was unsuccessful (**7** was largely recovered intact; Scheme 6d). Thus, a pathway for intramolecular hydride transfer appears to be an important feature of this reaction.

**Scheme 7:**
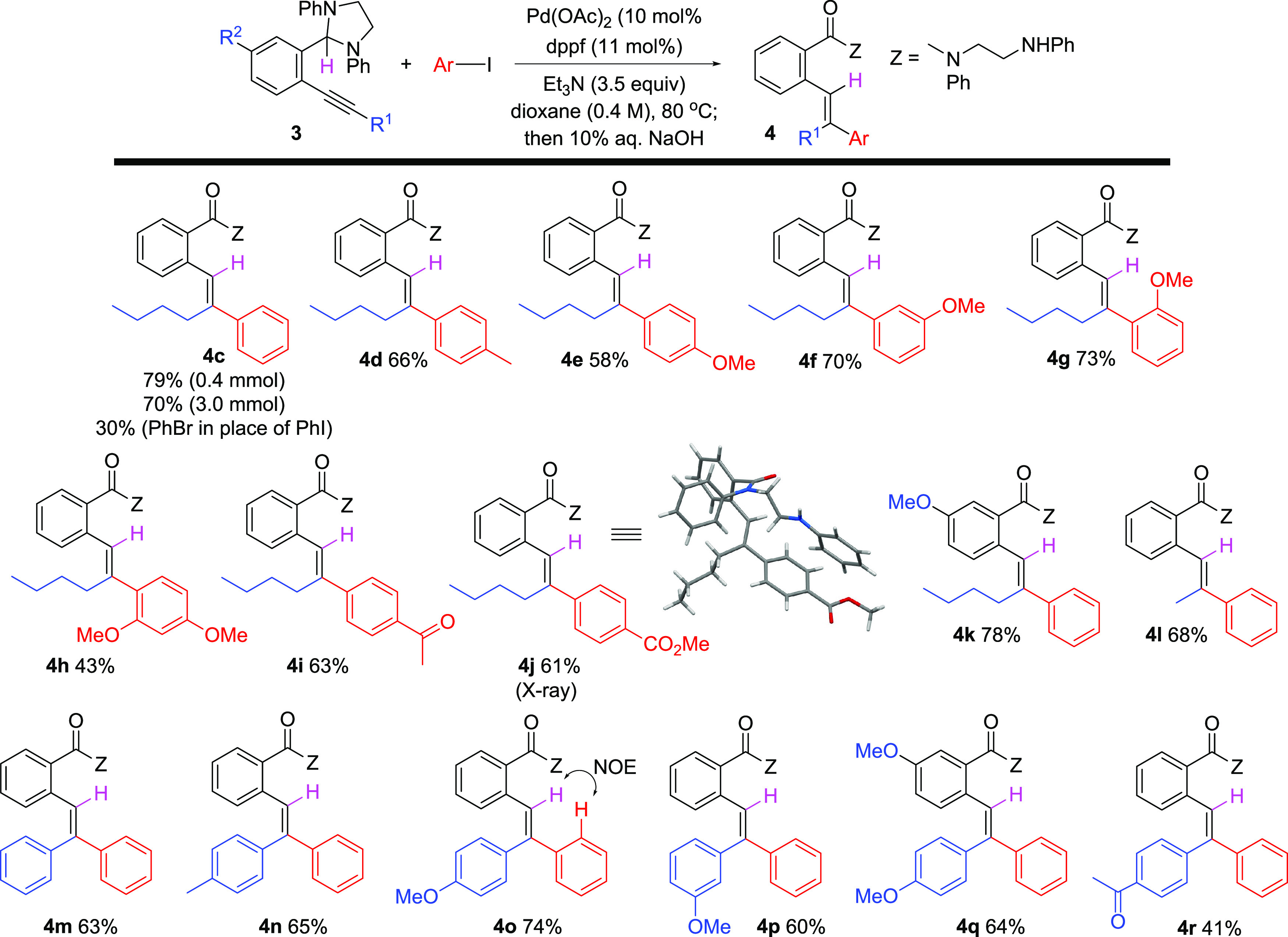
Scope of Pd-Catalyzed Alkyne Hydroarylation

With the identification of a reliable hydroarylation procedure (Table 1, entry 19), the scope of the reaction was examined using various aryl halides as organic electrophiles along with several different alkynyl imidazolidines (Scheme 7). First, scalability of the reaction was demonstrated by conversion of **3c** to **4c** on a 3.0 mmol (0.991 g) scale in 70% isolated yield. Using bromobenzene in place of iodobenzene, however, resulted in significantly decreased yield of **4c**. Both electron-rich and electron-deficient aryl iodides proved to be acceptable reaction partners. Thus, reaction of **3c** with various methyl- and methoxy-substituted aryl iodides returned products **4d**–**4h**. *para*-Iodo acetophenone and 4-iodo methyl benzoate were also suitable reactants, and **4i**–**4j** were isolated in reasonable yields. An additional substituent (R^2^ = OMe) on the 2-phenylimidazolidine fragment (**3k**, R^1^ = nBu, R^2^ = OMe) was tolerated as well, and Pd-catalyzed hydroarylation with iodobenzene gave **4k** in good isolated yield. A methyl-substituted alkyne also gave the reaction (**4l**). Alkene geometry in each case was assigned by analogy to **4c** and further confirmed in the case of **4j** through X-ray crystallography (see the Supporting Information). Several imidazolidine-substituted diaryl acetylenes equipped with electron-donating (OMe) or electron-withdrawing (methyl ketone) groups were subjected to these reaction conditions in the presence of iodobenzene, and in each case, regio- and stereoselective Pd-catalyzed hydroarylation was observed. Triaryl alkenes **4m**–**4r** were generated upon initial arylation of the alkyne terminus distal from the *ortho*-substituted 2-phenylimidazolidine group followed by hydride transfer. Regioselectivity in these cases is attributed primarily to steric effects such that arylation occurs at the more accessible alkyne carbon, although a directing effect via transient coordination of organopalladium intermediates by an imidazolidine nitrogen atom may play a role as well. Once again, alkene stereochemistry was assigned by analogy to **4c**. Additionally, the structure of **4o** was confirmed using 2D NMR spectroscopy, and a key NOE correlation was observed between the alkene hydrogen and the indicated aromatic hydrogens.

**Scheme 8:**
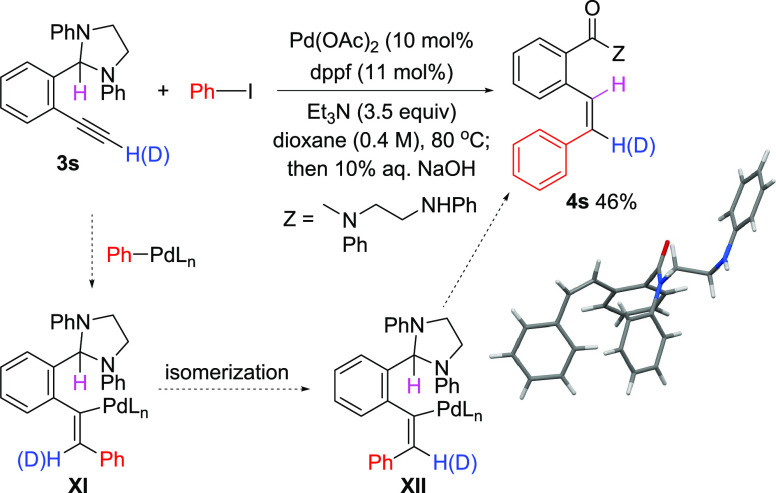
*trans* Hydroarylation of Terminal Alkyne **3s**

Hydroarylation of the terminal alkyne **3s** also was observed to proceed regio- and stereoselectively; however, in this instance, the *cis*-alkene **4s** was obtained—the product of formal *trans* hydroarylation (Scheme 8). The structure of **4s** was definitively established by X-ray crystallography. To gain insight into the mechanistic features of this transformation, we examined the reaction of deuterated **3s** (alkynyl C–H replaced with deuterium) and observed the formation of deuterated **4s** with no scrambling of the deuterium label as determined by 2D NMR analysis (see the Supporting Information). This result indicates that a metal vinylidene intermediate is not formed in the reaction. Accordingly, then, we speculate that this unexpected stereochemical outcome may reflect rapid isomerization of an initially formed *syn* carbo-palladated intermediate **XI** to the *trans* isomer **XII**, followed by Pd–H formation and reductive elimination. Isomerization may be driven by relief of steric strain in **XI**, and similar reactivity has been observed in other Pd-catalyzed additions to alkynes.^39−41^ In contrast, reactions of internal alkynes (**3c**–**3r**) proceed via conventional *syn* carbopalladation pathways as the additional alkyne substituent (alkyl or aryl) removes the driving force for isomerization.

**Figure 3:**
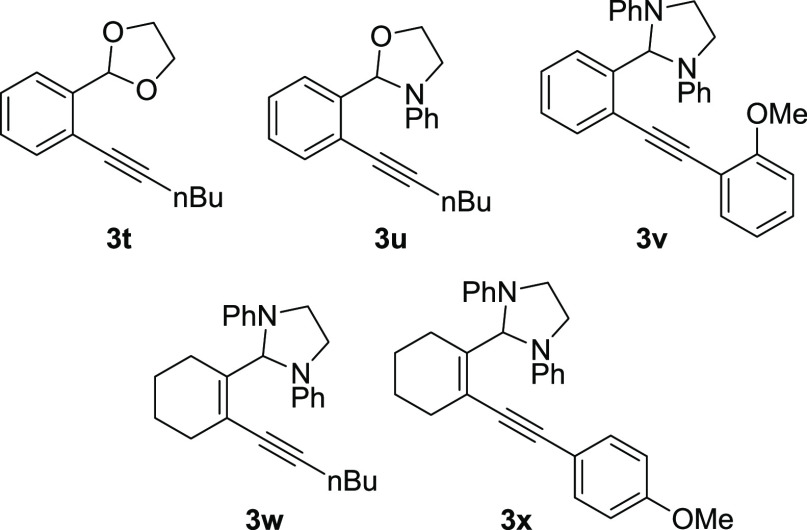
Unreactive hydroarylation substrates.

Several additional compounds (Figure 3) were examined as hydroarylation substrates but were found to be either unreactive or returned intractable mixtures under standard conditions (Table 1, entry 19). The acetal **3t** gave a complicated product mixture when exposed to typical hydroarylation conditions, while the N,O-acetal **3u** was unreactive. These results highlight the importance of a good hydride donor, as embodied in the *N*,*N*′-diphenylimidazolidine ring, for successful transformations. Diarylalkyne **3v** was also unreactive, presumably due to steric congestion at both alkyne carbons. Cyclohexene-derived substrates **3w** and **3x** were unreactive as well, and both substrates were recovered in high yield after attempted hydroarylation.

**Scheme 9:**
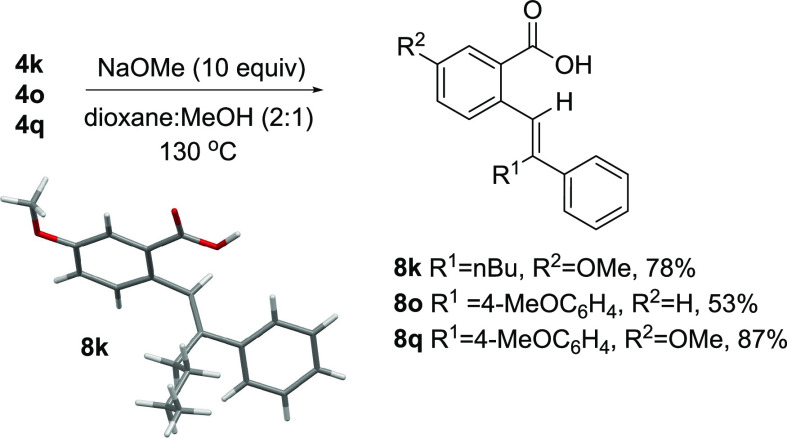
Anilide Saponification

Removal of the anilide group in hydroarylated products **4** was achieved under basic conditions. Combining NaOMe with **4** in a dioxane/MeOH solution and heating to 130 °C provided carboxylic acids **8** (Scheme 9). Apparently, the water content in MeOH was sufficient to induce saponification of any methyl ester derivatives initially formed. An X-ray crystal structure of product **8k** was obtained, which confirmed the preservation of alkene stereochemistry under these conditions. Attempts to hydrolyze anilides **4** under acidic conditions were unsuccessful.

**Scheme 10:**
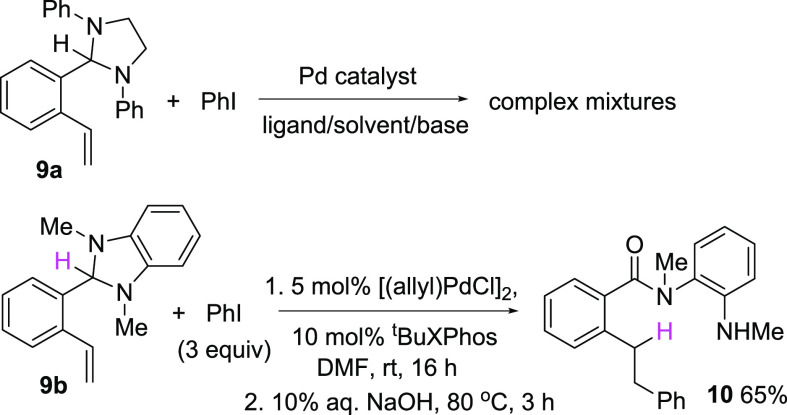
Benzimidazoline-Mediated Alkene Hydroarylation

In a final series of experiments, we sought to extend this imidazolidine-mediated reductive Heck-type transformation to include alkene hydroarylation. We initially investigated the reactivity of **9a**, an analogue of **3s** in which the terminal alkyne group was replaced with a simple vinyl substituent (i.e., the styrene analogue of **3s**). Exposure of this material to conditions that were effective for alkyne hydroarylation, however, returned only complex reaction mixtures (Scheme 10). Varying the source of the Pd catalyst and ligand system failed to improve the outcome, so we examined alternative imidazolidine substrates. We reasoned that incorporating a better hydride donor into the styrene substrate may be beneficial, and benzimidazoline moieties are better hydride donors than imidazolidines due to the aromaticity of benzimidazolium cations generated upon loss of H^–^. Consequently, styrene derivative **9b** was prepared in which an *N*,*N*-dimethylbenzimidazoline fragment resembling the potent hydride donor DMBI (see Scheme 2) was positioned to act as an internal hydride donor. Initial attempts to perform the Pd-catalyzed hydroarylation of **9b** were encouraging in that the desired diarylethane product **10** was detected by mass spectrometry along with the stilbene product arising from a conventional Heck reaction. Ultimately, we found that exposure of **9b** and iodobenzene to a catalyst generated from [(allyl)PdCl]_2_ and the Buchwald ligand ^t^BuXPhos at room temperature in the absence of base followed by basic hydrolysis of a putative benzimidazolium cation intermediate afforded hydroarylated product **10** exclusively in 65% isolated yield. This result demonstrates the feasibility of using an internal benzimidazoline hydride donor to mediate Pd-catalyzed alkene hydroarylation at the expense of β-hydride elimination under mild conditions and establishes a foundation for future studies exploring asymmetric variations of this reaction in structurally related systems.

## Conclusions

Appropriately positioned imidazolidine and benzimidazoline derivatives are viable hydride donors in Pd-catalyzed alkyne and alkene hydroarylation reactions. These heterocycles are shown to participate in intramolecular formal 1,4- and 1,5-hydride transfers in concert with the formation of aryl, alkenyl, and alkyl palladium complex intermediates. Alkyne hydroarylation occurs stereoselectively with the concomitant generation of oxidized imidazolium fragments as carboxylic acid equivalents. The reaction was successfully extended to include alkene hydroarylation in the presence of a benzimidazoline group as a hydride source. The utilization of imidazolidine hydride donors in reductive Heck-type transformations demonstrates the compatibility of these heterocycle reactants with organotransition metal-catalyzed processes and establishes an additional approach to achieving metal-promoted hydrocarbonation. Efforts to apply imidazolidine hydride donors in asymmetric transformations are the subject of ongoing investigations.

## Experimental Section

### General Considerations

All commercially available starting materials and reagents were used as received unless otherwise noted. Reactions were performed under an argon atmosphere unless otherwise noted. Hydrogen (^1^H) and carbon (^13^C) NMR spectra were recorded on Bruker Fourier 300, DRX 400, AVANCE NEO 400, AVANCE 500, or AVANCE 600 spectrometers. Chemical shifts are reported as δ values in parts per million (ppm) relative to TMS for ^1^H NMR in CDCl_3_ and residual nondeuterated solvent for all other spectra. Column chromatography was done using Silicycle SilicaFlash F60 silica gel (230–400 mesh) as the stationary phase. The silica gel was deactivated by flushing the column with 1% Et_3_N in hexane as an eluent system twice after packing the column. High resolution mass spectrometry (HRMS) was performed on a Waters Q-ToF Premier mass spectrometer using positive ion electrospray ionization (ESI). Melting points were recorded using a capillary tube on a Mel-Temp apparatus and were uncorrected. Procedures for the preparation of **3t**–**3x**, structures of known substrate precursors **S1**–**S12** and **6**, details of X-ray crystallography and 2D NMR experiments, and deuterium content incorporation using ^1^H NMR can be found in the Supporting Information.

### General Procedure (GP1) for the Synthesis of Imidazolidines **3a**–**3c**, **3k**–**3s**, **3v**–**3x**, and **9a**

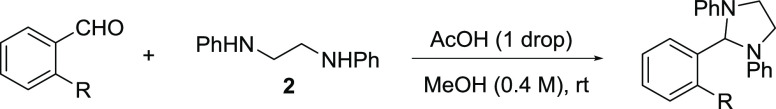



Benzaldehyde derivative (1.0 equiv) and *N*^1^,*N*^2^-diphenylethane-1,2-diamine (**2**, 1.1 equiv)^42^ were dissolved in methanol (0.4 M) with a drop of glacial acetic acid, and the reaction mixture was vigorously stirred at room temperature overnight (∼15 h). The solid precipitate was collected by filtration, washed with cold methanol, and recrystallized from ethyl acetate to give the desired product.

#### 2-(2-Iodophenyl)-1,3-diphenylimidazolidine (**3a**)

**3a** was prepared from 2-iodobenzaldehyde (0.3 g, 1.3 mmol) following GP1 and obtained as a white solid (0.49 g, 89%). Mp: 97–98 °C; ^1^H NMR (CDCl_3_, 400 MHz): δ = 7.84 (dd, *J* = 7.9, 1.0 Hz, 1H), 7.32–7.21 (m, 6H), 6.93 (td, *J* = 7.5, 1.8 Hz, 1H), 6.84–6.78 (m, 6H), 6.05 (s, 1H), 3.87 (m, 2H), 3.65 (m, 2H); ^13^C{^1^H} NMR (CDCl_3_, 100 MHz): δ = 146.8, 142.3, 140.4, 129.9, 129.2, 129.1, 128.6, 119.2, 116.0, 99.9, 81.7, 48.2; HRMS (ESI) *m*/*z* calcd for C_21_H_20_IN_2_ [M + H]^+^: 427.0666, found: 427.0661.

#### 1,2,3-Triphenylimidazolidine (**3b**/**7**)

**3b**/**7** was prepared from benzaldehyde (0.32 g, 0.31 mL, 3.0 mmol) following GP1 and obtained as an off-white solid (0.65 g, 71%). Mp: 131–132 °C; ^1^H NMR (CDCl_3_, 400 MHz): δ = 7.44 (d, *J* = 7.7 Hz, 2H), 7.28–7.23 (m, 2H), 7.18 (t, *J* = 7.9 Hz, 5H), 6.71 (m, 6H), 6.02 (s, 1H), 3.91 (m, 2H), 3.75 (m, 2H); ^13^C{^1^H} NMR (CDCl_3_, 100 MHz): δ = 145.7, 141.3, 129.2, 128.5, 128.1, 127.8, 117.8, 113.7, 77.3, 46.2; HRMS (ESI) *m*/*z* calcd for C_21_H_21_N_2_ [M + H]^+^: 301.1699, found: 301.1697.

#### 2-(2-(Hex-1-yn-1-yl)phenyl)-1,3-diphenylimidazolidine (**3c**)

**3c** was prepared from **S1** (0.3 g, 1.63 mmol; see the Supporting Information) following GP1 and obtained as a white crystalline solid (0.48 g, 92%). Mp: 98–100 °C; ^1^H NMR (CDCl_3_, 400 MHz): δ = 7.35 (d, *J* = 7.5 Hz, 1H), 7.27 (d, J = 7.7 Hz, 1H), 7.22–7.06 (m, 6H), 6.85 (d, *J* = 8.5 Hz, 4H), 6.70 (t, *J* = 7.3 Hz, 2H), 6.45 (s, 1H), 4.03 (m, 2H), 3.77 (m, 2H), 2.54 (t, *J* = 7.0 Hz, 2H), 1.64 (m, 2H), 1.5 (m, 2H), 0.93 (t, *J* = 7.2 Hz, 3H); ^13^C{^1^H} NMR (CDCl_3_, 100 MHz): δ = 145.8, 143.5, 132.2, 129.1, 128.5, 127.7, 127.3, 124.3, 117.8, 113.8, 97.3, 79.1, 74.4, 46.9, 31.0, 22.3, 19.5, 13.8. HRMS (ESI) *m*/*z* calcd for C_27_H_29_N_2_ [M + H]^+^: 381.2325, found: 381.2328.

#### 2-(2-(Hex-1-yn-1-yl)-5-methoxyphenyl)-1,3-diphenylimidazolidine (**3k**)

**3k** was prepared from **S7** (0.4 g, 1.85 mmol) following GP1 and obtained as a white solid (0.60 g, 80%). Mp: 97–99 °C; ^1^H NMR (CDCl_3_, 400 MHz): δ = 7.29 (d, *J* = 6.5 Hz, 1H), 7.18 (m, 4H), 6.86 (m, 4H), 6.78 (d, *J* = 2.6 Hz, 1H), 6.71 (t, *J* = 7.3 Hz, 2H), 6.63 (dd, *J* = 8.6, 2.7 Hz, 1H), 6.42 (s, 1H), 4.01 (m, 2H), 3.76 (m, 2H), 3.63 (s, 3H), 2.51 (t, *J* = 7.0 Hz, 2H), 1.63 (m, 2H), 1.52 (m, 2H), 0.93 (t, *J* = 7.3 Hz, 3H); ^13^C{^1^H} NMR (CDCl_3_, 100 MHz): δ = 159.6, 145.6, 145.2, 133.4, 129.1, 117.7, 116.9, 113.8, 113.3, 113.0, 95.6, 78.9, 74.3, 55.1, 46.8, 31.1, 22.3, 19.4, 13.8; HRMS (ESI) *m*/*z* calcd for C_28_H_31_N_2_O [M + H]^+^: 411.2431, found: 411.2428.

#### 1,3-Diphenyl-2-(2-(prop-1-yn-1-yl)phenyl)imidazolidine (**3l**)

**3s** (0.41 g, 1.26 mmol) was added to an oven-dried round-bottom flask containing dry THF (10 mL, 0.13 M) and the mixture cooled to −78 °C in a dry ice/acetone bath. nBuLi (0.76 mL, 1.89 mmol) was then added dropwise. Once the addition was complete, the reaction was allowed stir for 30 min. Methyl iodide (0.157 mL, 2.52 mmol) was then added and stirring continued at room temperature overnight. The mixture was then extracted with diethyl ether (3 × 10 mL) and the combined extracts dried over anhydrous Na_2_SO_4_. Filtration and concentration in vacuo afforded a crude product that was recrystallized from EtOAc to give **3l** as a white solid (0.24 g, 56%). Mp: 172-174 °C; ^1^H NMR (CDCl_3_, 400 MHz): δ = 7.36 (dd, *J* = 7.6, 1.4 Hz, 1H), 7.28 (m, 1H) 7.18 (m, 4H), 7.14–7.08 (m, 2H), 6.84 (d, *J* = 8.0 Hz, 4H), 6.72 (t, *J* = 7.3 Hz, 2H), 6.44 (s, 1H), 4.01 (m, 2H), 3.76 (m, 2H), 2.12 (s, 3H); ^13^C{^1^H} NMR (CDCl_3_, 100 MHz): δ = 146.0, 143.6, 132.2, 129.1, 128.5, 127.7, 127.3, 124.2, 117.9, 114.0, 92.7, 78.4, 74.8, 47.0, 4.6; HRMS (ESI) *m*/*z* calcd for C_24_H_23_N_2_ [M + H]^+^: 339.1856, found: 339.1853.

#### 1,3-Diphenyl-2-(2-(phenylethynyl)phenyl)imidazolidine (**3m**)

**3m** was prepared from **S3** (0.26 g, 1.26 mmol) following GP1 and obtained as a white solid (0.37 g, 73%). Mp: 141–143 °C; ^1^H NMR (CDCl_3_, 400 MHz): δ = 7.52–7.48 (m, 3H), 7.37–7.32 (m, 4H), 7.25–7.16 (m, 6H), 6.89 (d, *J* = 7.8 Hz, 4H), 6.72 (t, *J* = 7.3, 2H), 6.55 (s, 1H), 4.06 (m, 2H), 3.79 (m, 2H); ^13^C{^1^H} NMR (CDCl_3_, 100 MHz): δ = 145.9, 143.8, 132.4, 131.6, 129.3, 129.2, 128.7, 128.6, 127.9, 127.6, 123.3, 123.1, 118.0, 114.0, 95.8, 87.9, 74.9, 47.1; HRMS (ESI) *m*/*z* calcd for C_29_H_25_N_2_ [M + H]^+^: 401.2012, found: 401.2014.

#### 1,3-Diphenyl-2-(2-(*p*-tolylethynyl)phenyl)imidazolidine (**3n**)

**3n** was prepared from **S4** (0.32 g, 1.45 mmol) following GP1 and obtained as a white solid (0.60 g, 93%). Mp: 172–174 °C; ^1^H NMR (CDCl_3_, 400 MHz): δ = 7.49 (d, *J* = 7.3 Hz, 1H), 7.39 (d, *J* = 8.1 Hz, 2H), 7.35 (d, *J* = 8.2 Hz, 1H), 7.22–7.13 (m, 8H), 6.89 (d, *J* = 8.1 Hz, 4H), 6.71 (t, *J* = 7.2 Hz, 2H), 6.55 (s, 1H), 4.05 (m, 2H), 3.78 (m, 2H), 2.37 (s, 3H); ^13^C{^1^H} NMR (CDCl_3_, 100 MHz): δ = 145.9, 143.6, 138.9, 132.3, 131.5, 129.3, 129.2, 129.1, 127.9, 127.6, 123.5, 120.0, 118.0, 114.0, 96.0, 87.2, 74.8, 47.1, 21.7; HRMS (ESI) *m*/*z* calcd for C_30_H_27_N_2_ [M + H]^+^: 415.2169, found: 415.2172.

#### 2-(2-((4-Methoxyphenyl)ethynyl)phenyl)-1,3-diphenylimidazolidine (**3o**)

**3o** was prepared from **S5** (0.23 g, 0.97 mmol) following GP1 and obtained as a white solid (0.35 g, 83%). Mp: 161–163 °C; ^1^H NMR (CDCl_3_, 400 MHz): δ = 7.48 (dd, *J* = 7.1, 1.6 Hz, 1H), 7.43 (m, 2H), 7.34 (dd, *J* = 7.4, 1.4 Hz, 1H), 7.20–7.16 (m, 6H), 6.9–6.85 (m, 6H), 6.72 (t, *J* = 7.3 Hz, 2H), 6.54 (s, 1H), 4.05 (m, 2H), 3.82 (s, 3H), 3.79 (m, 2H); ^13^C{^1^H} NMR (CDCl_3_, 100 MHz): δ = 160.0, 146.0, 143.5, 133.1, 132.2, 129.2, 128.9, 127.9, 127.5, 123.7, 118.0, 115.2, 114.2, 114.0, 95.9, 86.6, 74.9, 55.5, 47.1; HRMS (ESI) *m*/*z* calcd for C_30_H_27_N_2_O [M + H]^+^: 431.2118, found: 431.2122.

#### 2-(2-((3-Methoxyphenyl)ethynyl)phenyl)-1,3-diphenylimidazolidine (**3p**)

**3p** was prepared from **S6** (0.58 g, 2.45 mmol) following GP1 and obtained as a white solid (0.71 g, 68%). Mp: 149–150 °C; ^1^H NMR (CDCl_3_, 400 MHz): δ = 7.50 (dd, *J* = 7.6, 1.2 Hz, 1H), 7.36 (dd, *J* = 7.8, 1.1 Hz, 1H), 7.26–7.15 (m, 7H), 7.10 (m, 1H), 7.07 (m, 1H), 6.92–6.88 (m, 5H), 6.71 (t, *J* = 7.3 Hz, 2H), 6.53 (s, 1H), 4.05 (m, 2H), 3.80–3.77 (m, 5H); ^13^C{^1^H} NMR (CDCl_3_, 100 MHz): δ = 159.6, 145.8, 143.8, 132.4, 129.7, 129.3, 129.2, 127.9, 127.6, 124.2, 124.1, 123.2, 118.0, 116.3, 115.4, 114.0, 95.7, 87.7, 74.8, 55.5, 47.0; HRMS (ESI) *m*/*z* calcd for C_30_H_27_N_2_O [M + H]^+^: 431.2118, found: 431.2119.

#### 2-(5-Methoxy-2-((4-methoxyphenyl)ethynyl)phenyl)-1,3-diphenylimidazolidine (**3q**)

**3q** was prepared from **S8** (0.6 g, 2.25 mmol) following GP1 except using toluene as the solvent and obtained as a white solid (0.75 g, 75%). Mp: 172–173 °C; ^1^H NMR (CDCl_3_, 400 MHz): δ = 7.45–7.40 (m, 3H), 7.25–7.16 (m, 4H), 6.91–6.83 (m, 7H), 6.74–6.70 (m, 3H), 6.50 (s, 1H), 4.04 (m, 2H), 3.82 (s, 3H), 3.78 (m, 2H), 3.70 (s, 3H); ^13^C{^1^H} NMR (CDCl_3_, 100 MHz): δ = 160.1, 159.8, 145.8, 145.4, 133.5, 132.9, 129.2, 118.0, 116.2, 115.6, 114.2, 114.0, 113.5, 113.3, 94.5, 86.5, 74.8, 55.5, 55.3, 47.0; HRMS (ESI) *m*/*z* calcd for C_31_H_29_N_2_O_2_ [M + H]^+^: 461.2224, found: 461.2219.

#### 1-(4-((2-(1,3-Diphenylimidazolidin-2-yl)phenyl)ethynyl)phenyl)ethan-1-one (**3r**)

**3r** was prepared from **S9** (0.33 g, 1.33 mmol) following GP1 and obtained as a pale yellow solid (0.41 g, 71%)*.* Mp: 147-149 °C; ^1^H NMR (CDCl_3_, 400 MHz): δ = 7.90 (d, *J =* 8.3 Hz, 2H), 7.54 (dd, *J =* 7.5, 1.1 Hz, 1H), 7.49 (d, *J* = 8.3 Hz, 2H), 7.38 (m, 1H), 7.28–7.17 (m, 6H), 6.87 (d, *J =* 8.0 Hz, 4H), 6.75 (t, *J* = 7.3 Hz, 2H), 6.51 (s, 1H), 4.01 (m, 2H), 3.77 (m, 2H), 2.61 (s, 3H); ^13^C{^1^H} NMR (CDCl_3_, 100 MHz): δ = 197.4, 146.2, 144.0, 136.6, 132.7, 131.7, 129.7, 129.3, 128.5, 128.0, 127.9, 127.6, 122.6, 118.4, 114.3, 95.0, 91.1, 75.3, 47.3, 26.8; HRMS (ESI) *m*/*z* calcd for C_31_H_27_ON_2_ [M + H]^+^: 443.2118, found: 443.2110.

#### 2-(2-Ethynylphenyl)-1,3-diphenylimidazolidine (**3s**)

**3s** was prepared from **S2** (0.64 g, 4.91 mmol) following GP1 and obtained as a white solid (1.31 g, 82%). Mp: 135–136 °C; ^1^H NMR (CDCl_3_, 400 MHz): δ = 7.45 (dd, *J* = 7.7, 1.3 Hz, 1H), 7.34 (dd, *J* = 8.1, 1.0 Hz, 1H), 7.25–7.11 (m, 6H), 6.83 (m, 4H), 6.71 *J* = 7.3 Hz, 2H), 6.46 (s, 1H), 4.05 (m, 2H), 3.78 (m, 2H), 3.58 (s, 1H); ^13^C{^1^H} NMR (CDCl_3_, 100 MHz): δ = 145.6, 144.6, 133.0, 129.8, 129.1, 127.9, 127.7, 122.3, 117.9, 113.9, 83.8, 82.2, 74.4, 46.9; HRMS (ESI) *m*/*z* calcd for C_23_H_21_N_2_ [M + H]^+^: 325.1699, found 325.1695.

#### *N*-Phenyl-*N*-(2-(phenylamino)ethyl)benzamide (**4a**)

Triethylamine (0.072 mL, 0.52 mmol) was added to a solution of **3a** (0.11 g, 0.26 mmol) in 1.5 mL of acetonitrile, and the reaction mixture was deoxygenated with a stream of Ar for 30 min. Pd(PPh_3_)_4_ (30 mg, 10 mol %) was added, and the reaction mixture was heated in an 80 °C oil bath. After consumption of **3a** as indicated by TLC, the reaction mixture was allowed to cool to room temperature, filtered through a Celite plug, and concentrated in vacuo. The crude mixture was purified by flash column chromatography using 5–10% ethyl acetate in hexanes as the eluent to give **4a** as a colorless oil (71 mg, 87%); ^1^H NMR (CDCl_3_, 400 MHz): δ = 7.22–7.20 (m, 2H), 7.17–7.05 (m, 8H), 6.95–6.93 (m, 2H), 6.60 (t, *J* = 7.3 Hz, 1H), 6.51 (d, *J* = 7.8 Hz, 2H), 4.36 (br s, 1H), 4.13 (t, *J* = 6.1 Hz, 2H), 3.30 (t, *J* = 6.1 Hz, 2H); ^13^C{^1^H} NMR (CDCl_3_, 100 MHz): δ = 171.8, 148.2, 143.2, 135.8, 129.9, 129.4, 129.4, 128.9, 128.0, 127.9, 127.0, 117.3, 112.6, 49.9, 42.5; HRMS (ESI) *m*/*z* calcd for C_21_H_21_N_2_O [M + H]^+^: 317.1654, found: 317.1660.

### General Procedure (GP2) for the Synthesis of Hydroarylated Products (**4c**–**4s**)

Reactions were performed in a 20 mL scintillation vial. To a solution of imidazolidine **3** (0.4 mmol, 1.0 equiv) in 1,4-dioxane (0.4 M) was added aryl iodide (0.92 mmol, 2.3 equiv) and Et_3_N (1.4 mmol, 3.5 equiv). The reaction mixture was deoxygenated with a stream of Ar for 20 min followed by addition of Pd(OAc)_2_ (0.04 mmol, 10 mol %) and dppf (0.044 mmol, 11 mol %). The vial was capped and placed in a J-KEM Lab benchtop shaker heating block set to 80 °C and agitated until completion of the reaction as indicated by TLC (24–48 h). An aqueous 10% NaOH solution (5 mL) was then added to the reaction vessel, and heating was maintained for 3 h. The reaction was allowed to cool to room temperature and filtered through a Celite plug. The filtrate was extracted with ethyl acetate (3 × 10 mL), and the combined organic layer was washed with 1 M aqueous HCl (3 × 5 mL) and brine (1 × 5 mL) and then dried over anhydrous Na_2_SO_4_. Filtration and evaporation of the solvent gave a crude product that was purified by flash column chromatography to afford **4**.

#### (*E*)-*N*-Phenyl-*N*-(2-(phenylamino)ethyl)-2-(2-phenylhex-1-en-1-yl)benzamide (**4c**)

Obtained as orange oil (150 mg, 79%) from **3c** and iodobenzene; chromatography conditions: 5–12% ethyl acetate in hexanes; ^1^H NMR (CDCl_3_, 400 MHz): δ = 7.46–7.43 (m, 2H), 7.37–7.30 (m, 3H); 7.25–7.16 (m, 2H), 7.10–7.05 (m, 7 H), 6.88 (d, *J* = 6.9 Hz, 2H), 6.73 (s, 1H), 6.64 (t, *J* = 7.3 Hz, 1H), 6.38 (d, *J* = 7.8 Hz, 2H), 4.21 (br s, 1H), 4.10 (t, *J* = 5.6 Hz, 2H), 3.26 (t, *J* = 5.6 Hz, 2H), 2.30 (t, *J* = 7.5 Hz, 2H), 1.33–1.21 (m, 4H), 0.79 (t, *J* = 7.1 Hz, 3H); ^13^C{^1^H} NMR (CDCl_3_, 125 MHz): δ = 172.1, 148.1, 144.6, 142.4, 142.1, 136.9, 135.4, 129.3, 128.9, 128.8, 128.7, 128.7, 128.2, 127.9, 127.5, 127.0, 126.7, 126.4, 125.6, 117.2, 112.6, 49.0, 42.6, 31.1, 29.9, 23.0, 14.0; HRMS (ESI) *m*/*z* calcd for C_33_H_35_N_2_O [M + H]^+^: 475.2744, found: 475.2745.

#### (*E*)-*N*-Phenyl-*N*-(2-(phenylamino)ethyl)-2-(2-(*p*-tolyl)hex-1-en-1-yl)benzamide (**4d**)

Obtained as yellow oil (129 mg, 66%) from **3c** and *p*-iodotoluene; chromatography conditions: 5–12% ethyl acetate in hexanes; ^1^H NMR (CDCl_3_, 300 MHz): δ = 7.34 (d, *J* = 8.0 Hz, 2H), 7.25–7.05 (m, 11H), 6.89 (d, 2H), 6.70–6.62 (m, 2H), 6.39 (d, *J* = 7.5 Hz, 2H), 4.18 (br s, 1H), 4.10 (m, 2H), 3.27 (m, 2H), 2.39 (s, 3H), 2.26 (m, 2H), 1.31–1.20 (m, 4H), 0.79 (t, *J* = 7.2 Hz, 3H); ^13^C{^1^H} NMR (CDCl_3_, 100 MHz): δ = 172.1, 148.1, 144.4, 142.1, 139.4, 137.3, 137.0, 135.4, 129.4, 129.3, 128.9, 128.7, 128.7, 128.1, 127.9, 127.0, 126.5, 126.3, 124.8, 117.2, 112.6, 49.1, 42.6, 31.1, 29.8, 23.0, 21.3, 14.0; HRMS (ESI) *m*/*z* calcd for C_34_H_37_N_2_O [M + H]^+^: 489.2900, found: 489.2889.

#### (*E*)-2-(2-(4-Methoxyphenyl)hex-1-en-1-yl)-*N*-phenyl-*N*-(2-(phenylamino)ethyl)benzamide (**4e**)

Obtained as orange oil (117 mg, 58%) from **3c** and *p*-iodoanisole; chromatography conditions: 5–12% ethyl acetate in hexanes; ^1^H NMR (CDCl_3_, 400 MHz): δ = 7.39 (d, *J* = 8.7 Hz, 2H), 7.25–7.10 (m, 9H), 6.90–6.86 (m, 4H), 6.68–6.64 (m, 2H), 6.40 (d, *J* = 7.6 Hz, 2H), 4.26 (br s, 1H), 4.12 (m, 2H), 3.84 (s, 3H), 3.27 (m, 2H), 2.26 (t, J = 7.3 Hz, 2H), 1.32–1.21 (m, 4H), 0.80 (t, *J* = 7.1 Hz, 3H); ^13^C{^1^H} NMR (CDCl_3_, 100 MHz): δ = 172.2, 159.2, 148.2, 143.9, 142.1, 136.9, 135.5, 134.7, 129.3, 128.9, 128.7, 128.7, 128.1, 127.9, 127.7, 127.0, 126.3, 124.1, 117.2, 114.0, 112.6, 55.4, 49.0, 42.7, 31.2, 29.8, 23.0, 14.0; HRMS (ESI) *m*/*z* calcd for C_34_H_37_N_2_O_2_ [M + H]^+^: 505.2850, found: 505.2849.

#### (*E*)-2-(2-(3-Methoxyphenyl)hex-1-en-1-yl)-*N*-phenyl-*N*-(2-(phenylamino)ethyl)benzamide (**4f**)

Obtained as orange oil (141 mg, 70%) from **3c** and *m*-iodoanisole; chromatography conditions: 5–12% ethyl acetate in hexanes; ^1^H NMR (CDCl_3_, 400 MHz): δ = 7.29–7.23 (m, 2H), 7.21–7.17 (m, 1H), 7.11–7.04 (m, 8H), 6.98 (t, *J* = 2.0 Hz, 1H), 6.89–6.86 (m, 3H), 6.73 (s, 1H), 6.64 (t, *J* = 7.6 Hz, 1H), 6.39 (d, *J =* 7.6 Hz, 2H), 4.17 (br s, 1H), 4.10 (t, *J* = 5.7 Hz, 2H), 3.83 (s, 3H), 3.27 (m, 2H), 2.25 (m, 2H), 1.33–1.21 (m, 4H), 0.80 (t, *J* = 7.2 Hz, 3H); ^13^C{^1^H} NMR (CDCl_3_, 100 MHz): δ = 172.0, 159.8, 148.0, 144.5, 144.0, 142.0, 136.9, 135.2, 129.6, 129.3, 128.8, 128.7, 128.2, 127.9, 127.0, 126.9, 126.4, 125.6, 119.2, 117.1, 112.6, 112.6, 112.5, 55.3, 49.0, 42.4, 31.0, 30.0, 23.0, 14.0; HRMS (ESI) *m*/*z* calcd for C_34_H_37_N_2_O_2_ [M + H]^+^: 505.2850, found: 505.2848.

#### (*E*)-2-(2-(2-Methoxyphenyl)hex-1-en-1-yl)-*N*-phenyl-*N*-(2-(phenylamino)ethyl)benzamide (**4g**)

Obtained as orange oil from **3c** and *o*-iodoanisole (147 mg, 73%); chromatography conditions: 1–5% ethyl acetate in toluene; ^1^H NMR (CDCl_3_, 400 MHz): δ = 7.29–7.25 (m, 1H), 7.22–7.02 (m, 12H), 6.92 (t, *J* = 7.4 Hz, 1H), 6.84 (d, *J* = 8.1 Hz, 1H), 6.65 (t, *J* = 7.5 Hz, 1H), 6.56 (s, 1H), 6.41 (d, *J* = 8.2 Hz, 2H), 4.28 (br s, 1H), 4.14 (t, *J* = 5.3 Hz, 2H), 3.75 (s, 3H), 3.30 (m, 2H), 2.43 (m, 2H), 1.21 (m, 4H), 0.77 (t, *J* = 6.6 Hz, 3H); ^13^C{^1^H} NMR (CDCl_3_, 100 MHz): δ = 172.1, 156.7, 148.2, 144.8, 142.1, 136.9, 135.2, 132.9, 130.6, 129.3, 129.3, 128.9, 128.4, 128.3, 128.3, 127.5, 127.3, 126.8, 126.1, 120.7, 117.1, 112.5, 110.8, 55.4, 48.7, 42.4, 31.0, 30.9, 23.0, 14.0; HRMS (ESI) *m*/*z* calcd for C_34_H_37_N_2_O_2_ [M + H]^+^: 505.2850, found: 505.2848.

#### (*E*)-2-(2-(2,4-Dimethoxyphenyl)hex-1-en-1-yl)-*N*-phenyl-*N*-(2-(phenylamino)ethyl)benzamide (**4h**)

Obtained as colorless oil from **3c** and 2,4-dimethoxyiodobenzene (92 mg, 43%); chromatography conditions: 5–10% ethyl acetate in hexanes; ^1^H NMR (CDCl_3_, 400 MHz): δ = 7.20–7.01 (m, 12H), 6.67 (t, *J* = 7.8 Hz, 1 H), 6.56 (s, 1H), 6.46–6.40 (m, 4H), 4.17 (t, *J* = 5.7 Hz, 2H), 3.83 (s, 3H), 3.74 (s, 3H), 3.32 (t, *J* = 5.7 Hz, 2H), 2.42 (m, 2H), 1,25–1.21 (m, 4H), 0.79 (t, *J* = 6.9 Hz, 3H); ^13^C{^1^H} NMR (CDCl_3_, 100 MHz): δ = 172.2, 160.1, 157.7, 148.0, 144.6, 142.1, 136.8, 135.3, 130.9, 129.3, 128.9, 128.2, 127.4, 127.3, 126.4, 125.9, 125.6, 117.2, 112.6, 104.1, 98.8, 55.4, 55.3, 48.8, 42.6, 31.0, 30.8, 22.9, 14.1; HRMS (ESI) *m*/*z* calcd for C_35_H_39_N_2_O_3_ [M + H]^+^: 535.2955, found: 535.2957.

#### (*E*)-2-(2-(4-Acetylphenyl)hex-1-en-1-yl)-*N*-phenyl-*N*-(2-(phenylamino)ethyl)benzamide (**4i**)

Obtained as yellow oil from **3c** and *p*-iodoacetophenone (130 mg, 63%); chromatography conditions: 5–15% ethyl acetate in hexanes; ^1^H NMR (CDCl_3_, 300 MHz): δ = 7.90 (d, *J* = 8.2 Hz, 2H), 7.5 (d, *J* = 8.2 Hz, 2H), 7.23–7.04 (m, 9H), 6.90 (m, 2H), 6.83 (s, 1H), 6.66 (t, *J* = 6.9 Hz, 1H), 6.40 (d, *J* = 8.2 Hz, 2H), 4.29 (br s, 1H), 4.13 (m, 2 H), 3.26 (t, *J* = 5.5 Hz, 2H), 2.61 (s, 3H), 2.35 (m, 2H), 1.27–1.21 (m, 4H), 0.80 (t, *J* = 6.9 Hz, 3H); ^13^C{^1^H} NMR (CDCl_3_, 75 MHz): δ = 197.8, 172.0, 148.2, 147.3, 144.9, 143.7, 142.1, 137.0, 136.2, 134.9, 131.2, 129.3, 128.9, 128.8, 128.8, 128.2, 127.9, 127.4, 127.2, 126.8, 117.3, 112.6, 49.1, 42.9, 31.0, 29.8, 26.8, 22.9, 13.9; HRMS (ESI) *m*/*z* calcd for C_35_H_37_N_2_O_2_ [M + H]^+^: 517.2850, found: 517.2846.

#### Methyl (*E*)-4-(1-(2-(Phenyl(2-(phenylamino)ethyl)carbamoyl)phenyl)hex-1-en-2-yl)benzoate (**4j**)

Obtained as an off-white solid from **3c** and *p*-iodo methyl benzoate (130 mg, 61%). Mp: 103–105 °C; chromatography conditions: 5–15% ethyl acetate in hexanes; ^1^H NMR (CDCl_3_, 400 MHz): δ = 8.00 (d, *J* = 8.4 Hz, 2H), 7.49 (d, *J* = 8.3 Hz, 2H), 7.26–7.18 (m, 2H), 7.12–7.04 (m, 7H), 6.87 (d, *J* = 6.7 Hz, 2H), 6.81 (s, 1H), 6.65 (t, *J* = 7.3 Hz, 1H), 6.38 (d, *J* = 7.9 Hz, 2H), 4.27 (br s, 1H), 4.12 (m, 2H), 3.93 (s, 3H), 3.25 (t, *J* = 5.8 Hz, 2H), 2.32 (m, 2H), 1.31–1.21 (m, 4H), 0.79 (t, *J* = 7.0 Hz, 3H); ^13^C{^1^H} NMR (CDCl_3_, 100 MHz): δ = 171.9, 167.1, 148.1, 147.1, 143.7, 142.0, 136.9, 134.9, 130.0, 129.3, 129.1, 128.8, 128.8, 128.8, 128.3, 127.8, 127.3, 127.1, 126.8, 126.6, 117.3, 112.5, 52.2, 49.0, 42.7, 30.9, 29.7, 22.9, 13.9; HRMS (ESI) *m*/*z* calcd for C_35_H_37_N_2_O_3_ [M + H]^+^: 533.2799, found: 533.2800. A single crystal of **4j** was obtained by crystallization from CDCl_3_.

#### (*E*)-5-Methoxy-*N*-phenyl-*N*-(2-(phenylamino)ethyl)-2-(2-phenylhex-1-en-1-yl)benzamide (**4k**)

Obtained as yellow oil from **3k** and iodobenzene (157 mg, 78%); chromatography conditions: 5–12% ethyl acetate in hexanes; ^1^H NMR (CDCl_3_, 400 MHz): δ = 7.44–7.41 (m, 2H), 7.36–7.27 (m, 3H), 7.14–7.06 (m, 5H), 7.02 (d, *J* = 8.3 Hz, 1H), 6.89 (d, *J* = 6.9 Hz, 2H), 6.79–6.72 (m, 2H), 6.66–6.62 (m, 2H), 6.39 (d, *J =* 7.9 Hz, 2H), 4.17 (br s, 1H), 4.09 (t, *J =* 5.8 Hz, 2H), 3.71 (s, 3H), 3.27 (t, J = 5.8 Hz, 2H), 2.27 (t, *J =* 7.7 Hz, 2H), 1.32–1.21 (m, 4H), 0.80 (t, *J* = 7.1 Hz, 3H); ^13^C{^1^H} NMR (CDCl_3_, 100 MHz): δ = 171.7, 157.9, 148.1, 143.6, 142.5, 142.0, 138.0, 130.1, 129.3, 128.7, 128.6, 128.0, 127.8, 127.3, 127.0, 126.6, 125.2, 117.2, 114.9, 113.1, 112.6, 55.5, 49.1, 42.5, 31.0, 29.8, 23.0, 14.0; HRMS (ESI) *m*/*z* calcd for C_34_H_37_N_2_O_2_ [M + H]^+^: 505.2850, found: 505.2856.

#### (*E*)-*N*-Phenyl-*N*-(2-(phenylamino)ethyl)-2-(2-phenylprop-1-en-1-yl)benzamide (**4l**)

Obtained as yellow oil from **3l** and iodobenzene (117 mg, 68%); chromatography conditions: 5–12% ethyl acetate in hexanes; ^1^H NMR (CDCl_3_, 400 MHz): δ = 7.52 (m, 2H), 7.34–7.16 (m, 5H), 7.12–7.07 (m, 7H), 6.93–6.89 (m, 3H), 6.67 (t, *J* = 7.3 Hz, 1H), 6.47 (d, *J* = 7.9 Hz,2H), 4.35 (br s, 1H), 4.15 (t, *J* = 5.8 Hz, 2H), 3.32 (t, *J* = 5.8 Hz, 2H), 1.89 (s, 3H); ^13^C{^1^H} NMR (CDCl_3_, 100 MHz): δ = 172.2, 148.2, 142.9, 142.0, 138.5, 136.9, 135.3, 129.7, 129.3, 128.7, 128.6, 128.6, 128.0, 127.9, 127.6, 127.1, 126.4, 125.9, 125.2, 117.2, 112.6, 49.1, 42.8, 17.1; HRMS (ESI) *m*/*z* calcd for C_30_H_29_ON_2_ [M + H]^+^: 433.2274, found: 433.2270.

#### 2-(2,2-Diphenylvinyl)-*N*-phenyl-*N*-(2-(phenylamino)ethyl)benzamide (**4m**)

Obtained as yellow oil from **3m** and iodobenzene (125 mg, 63%); chromatography conditions: 5–15% ethyl acetate in hexanes; NMR (CDCl_3_, 400 MHz): δ = 7.32–7.28 (m, 5H), 7.24–7.16 (m, 4H), 7.13–7.06 (m, 5H), 7.01–6.94 (m, 4H), 6.79 (t, *J* = 7.9 Hz, 1H), 6.67–6.58 (m, 4H), 6.40 (d, *J* = 8.5 Hz, 2H), 4.27 (br s, 1H), 4.17 (t, *J* = 5.8 Hz, 2H), 3.31 (t, *J* = 5.8 Hz, 2H); ^13^C{^1^H} NMR (CDCl_3_, 100 MHz): δ = 172.2, 148.1, 144.1, 143.7, 142.2, 139.7, 137.3, 134.4, 130.7, 129.6, 129.3, 129.1, 128.5, 128.3, 128.2, 128.1, 128.1, 127.9, 127.6, 127.2, 126.5, 125.2, 117.2, 112.6, 49.3, 42.6; HRMS (ESI) *m*/*z* calcd for C_35_H_31_N_2_O [M + H]^+^: 495.2431, found: 495.2431.

#### (*Z*)-*N*-Phenyl-2-(2-phenyl-2-(*p*-tolyl)vinyl)-*N*-(2-(phenylamino)ethyl)benzamide (**4n**)

Obtained as orange oil from **3n** and iodobenzene (132 mg, 65%); chromatography conditions: 5–15% ethyl acetate in hexanes; ^1^H NMR (CDCl_3_, 400 MHz): δ = 7.31–7.27 (m, 5H), 7.24–7.22 (m, 1H), 7.18–7.15 (m, 3H), 7.07 (t, *J* = 7.9 Hz, 2H), 6.98–6.94 (m, 4H), 6.91 (d, *J* = 7.8 Hz, 2H), 6.79 (t, *J* = 7.8 Hz, 1H), 6.66–6.62 (m, 2H), 6.47 (d, J = 7.9 Hz, 2H), 6.39 (d, *J* = 7.9 Hz, 2H), 4.24 (br s, 1H), 4.15 (t, *J* = 5.8 Hz, 2H), 3.29 (t, *J* = 5.8 Hz, 2H), 2.28 (s, 3H); ^13^C{^1^H} NMR (CDCl_3_, 100 MHz): δ = 172.2, 148.1, 144.1, 143.9, 142.2, 137.3, 137.3, 136.6, 134.6, 130.6, 129.6, 129.3, 129.1, 128.9, 128.4, 128.3, 128.3, 128.1, 128.1, 127.8, 127.1, 126.3, 124.9, 117.2, 112.6, 49.3, 42.6, 21.3; HRMS (ESI) *m*/*z* calcd for C_36_H_33_N_2_O [M + H]^+^: 509.2587, found: 509.2587.

#### (*Z*)-2-(2-(4-Methoxyphenyl)-2-phenylvinyl)-*N*-phenyl-*N*-(2-(phenylamino)ethyl)benzamide (**4o**)

Obtained as orange oil from **3o** and iodobenzene (155 mg, 74%); chromatography conditions: 5–15% ethyl acetate in hexanes; ^1^H NMR (CDCl_3_, 600 MHz): δ = 7.32–7.25 (m, 6H), 7.17–7.16 (m, 3H), 7.08 (t, *J* = 7.8 Hz, 2H), 6.98–6.97 (m, 3H), 6.9 (s, 1H), 6.83 (t, *J* = 7.8 Hz, 1H), 6.67–6.62 (m, 4H), 6.47 (d, *J* = 8.4 Hz, 2H), 6.40 (d, *J* = 7.9 Hz, 2H), 4.25 (s, 1H), 4.16 (t, *J* = 5.7 Hz, 2H), 3.76 (s, 3H), 3.3 (t, *J* = 5.7 Hz, 2H); ^13^C{^1^H} NMR (CDCl_3_, 150 MHz): δ = 172.3, 159.1, 148.1, 144.1, 143.7, 142.2, 137.3, 134.7, 132.0, 131.8, 129.5, 129.3, 129.1, 128.4, 128.3, 128.2128.2, 127.9, 127.1, 126.3, 124.6, 117.2, 114.3, 113.5, 112.6, 55.3, 49.3, 42.6; HRMS (ESI) *m*/*z* calcd for C_36_H_33_N_2_O_2_ [M + H]^+^: 525.2537, found: 525.2528.

#### (*Z*)-2-(2-(3-Methoxyphenyl)-2-phenylvinyl)-*N*-phenyl-*N*-(2-(phenylamino)ethyl)benzamide (**4p**)

Obtained as yellow oil from **3p** and iodobenzene (126 mg, 60%); chromatography conditions: 5–15% ethyl acetate in hexanes; ^1^H NMR (CDCl_3_, 400 MHz): δ = 7.33–7.29 (m, 5H), 7.22–7.16 (m, 4H), 7.10–7.03 (m, 4H), 6.99–6.94 (m, 3H), 6.81 (t, *J* = 7.6 Hz, 1H), 6.76 (dd, *J* = 8.1, 2.1 Hz, 1H), 6.67–6.63 (m, 2H), 6.40 (d, *J* = 8.0 Hz, 2H), 6.32 (s, 1H), 6.23 (d, *J* = 7.4 Hz, 1H), 4.31 (br s, 1H), 4.17 (t, *J* = 5.8 Hz, 2H), 3.57 (s, 3H), 3.31 (t, *J* = 5.8 Hz, 2H); ^13^C{^1^H} NMR (CDCl_3_, 100 MHz): δ = 172.2, 159.6, 148.1, 143.8, 143.5, 142.2, 141.1, 137.3, 134.4, 129.5, 129.3, 129.3, 129.1, 128.5, 128.3, 128.2, 128.2, 128.0, 127.9, 127.3, 126.5, 125.3, 123.2, 117.2, 115.7, 113.7, 112.6, 55.4, 49.3, 42.7; HRMS (ESI) *m*/*z* calcd for C_36_H_33_N_2_O_2_ [M + H]^+^: 525.2537, found: 525.2538.

#### (*Z*)-5-Methoxy-2-(2-(4-methoxyphenyl)-2-phenylvinyl)-*N*-phenyl-*N*-(2-(phenylamino)ethyl)benzamide (**4q**)

Obtained as yellow oil from **3q** and iodobenzene (142 mg, 64%); chromatography conditions: 5–15% ethyl acetate in hexanes; ^1^H NMR (CDCl_3_, 400 MHz): δ = 7.31–7.27 (m, 5H), 7.19–7.18 (m, 3H), 7.09 (t, *J* = 7.8 Hz, 2H), 6.99–6.97 (m, 2H), 6.84 (s, 1H), 6.79 (d, *J* = 2.3 Hz, 1H), 6.67–6.63 (m, 3H), 6.57 (d, *J* = 8.7 Hz, 1H), 6.47–6.38 (m, 5H), 4.23 (br s, 1H), 4.14 (t, *J* = 5.8 Hz, 2H), 3.76 (s, 3H), 3.68 (s, 3H), 3.32 (t, *J* = 5.8 Hz, 2H); ^13^C{^1^H} NMR (CDCl_3_, 100 MHz): δ = 171.9, 158.9, 157.8, 148.1, 144.3, 142.2, 142.1, 138.5, 132.1, 131.9, 130.8, 129.3, 129.0, 128.4, 128.2, 128.0, 127.6, 127.3, 127.2, 124.3, 117.2, 115.0, 113.8, 113.6, 112.6, 55.4, 55.2, 49.5, 42.6; HRMS (ESI) *m*/*z* calcd for C_37_H_35_N_2_O_3_ [M + H]^+^: 555.2642, found: 555.2645.

#### (*Z*)-2-(2-(4-Acetylphenyl)-2-phenylvinyl)-*N*-phenyl-*N*-(2-(phenylamino)ethyl)benzamide (**4r**)

Obtained as an orange oil from **4r** and iodobenzene (87 mg, 41%); chromatography conditions: 5–15% ethyl acetate in hexanes; ^1^H NMR (CDCl_3_, 400 MHz): δ = 7.79 (d, *J =* 8.2 Hz, 2H), 7.35–7.24 (m, 6H), 7.19 (m, 3H), 7.10–7.06 (m, 3H), 7.02–6.99 (m, 3H), 6.80 (t, *J* = 7.6 Hz, 1H), 6.68–6.64 (m, 3H), 6.54 (d, *J =* 7.9 Hz, 1H), 6.40 (d, *J* = 7.9 Hz, 2H), 4.26 (br s, 1H), 5.17 (t, *J* = 5.8 Hz, 2H), 3.31 (t, *J* = 5.8 Hz, 2H), 2.56 (s, 3H); ^13^C{^1^H} NMR (CDCl_3_, 100 MHz): δ = 197.9, 172.0, 148.1, 145.0, 143.1, 142.9, 142.2, 137.4, 136.1, 134.0, 131.0, 129.7, 129.4, 129.2, 128.7, 128.6, 128.4, 128.3, 128.3, 128.2, 128.1, 127.3, 126.9, 126.7, 117.3, 112.6, 49.4, 42.7, 26.7; HRMS (ESI) *m*/*z* calcd for C_37_H_33_O_2_N_2_ [M + H]^+^: 537.2537, found: 537.2534.

#### (*Z*)-*N*-Phenyl-*N*-(2-(phenylamino)ethyl)-2-styrylbenzamide (**4s**)

Obtained as colorless solid from **3s** and iodobenzene (77 mg, 46%). Mp: 127–129 °C; chromatography conditions: 5–20% ethyl acetate in hexanes; ^1^H NMR (CDCl_3_, 400 MHz): δ = 7.19–7.08 (m, 12H), 7.01 (t, *J* = 7.3 Hz, 1H), 6.94–6.89 (m, 3H), 6.74–6.68 (m, 2H), 6.61–6.57 (m, 3H),4.41 (br s, 1H), 4.23 (t, *J* = 5.8 Hz, 2H), 3.38 (t, *J* = 5.8 Hz, 2H);^13^C{^1^H} NMR (CDCl_3_, 100 MHz): δ = 172.0, 148.3, 142.0, 136.6, 136.4, 134.6, 131.5, 129.5, 129.4, 129.3, 129.1, 128.4, 128.3, 128.1, 128.0, 127.6, 127.4, 127.4, 126.8, 117.3, 112.6, 49.0, 42.5; HRMS (ESI) *m*/*z* calcd for C_29_H_27_N_2_O [M + H]^+^: 419.2118, found: 419.2109. A single crystal of **4s** was obtained by slow solvent evaporation from EtOAc.

### Gram-Scale Synthesis of **4c**

To a solution of **3c** (0.991 g, 3 mmol) in 7.5 mL of 1,4-dioxane in an oven-dried 25 mL round-bottom flask was added iodobenzene (0.77 mL, 6.9 mmol) and Et_3_N (1.47 mL, 10.5 mmol), and then the mixture was deoxygenated for 30 min with a stream of Ar. Pd(OAc)_2_ (67 mg, 10 mol %) and dppf (180 mg, 11 mol %) were added, and the reaction mixture was stirred in an 80 °C oil bath until completion as indicated by TLC (17 h). Aqueous 10% NaOH solution was then added (30 mL), and the reaction was maintained for an additional 3 h. The reaction was allowed to cool to room temperature, filtered through a Celite plug, and the filtrate extracted with ethyl acetate (3 × 15 mL). The combined organic layer was washed with 1 M aqueous HCl (4 × 10 mL) and brine (1 × 10 mL) and dried over anhydrous Na_2_SO_4_. After filtration and removal of the solvent in vacuo*,* the crude mixture was purified by flash column chromatography on deactivated silica gel using 7–10% ethyl acetate in hexanes as the eluent to afford **4c** as an orange oil (0.98 g, 70%).

#### 2-(2-(10-Butylphenanthren-9-yl)phenyl)-1,3-diphenylimidazolidine (**5**)

To a solution of **4c** (0.09 g, 0.24 mmol, 1.0 equiv) in 1,4-dioxane (0.6 mL, 0.4 M) was added iodobenzene (0.105 mL, 0.95 mmol, 4.0 equiv) and Cs_2_CO_3_ (0.15 g, 0.47 mmol, 2.0 equiv). Pd(OAc)_2_ (5.3 mg, 0.024 mmol, 10 mol %) and dppf (14.4 mg, 0.026 mmol, 11 mol %) were added to the reaction mixture, and the reaction mixture was stirred at 80 °C until completion of the reaction as indicated by TLC (16 h). The mixture was allowed to cool to room temperature, filtered through a Celite plug, concentrated in vacuo, and purified by flash column chromatography on deactivated silica using 1–3% ethyl acetate in hexanes as the eluent to give **5** as a white solid. Mp: 145–148 °C; ^1^H NMR (CDCl_3_, 400 MHz): δ = 8.70 (d, *J* = 7.5 Hz, 1H), 8.62 (d, *J* = 8.3 Hz, 1H), 7.91 (d, *J* = 7.9 Hz, 1H), 7.82 (d, *J* = 7.6 Hz, 1H), 7.64–7.40 (m, 5H), 7.22–7.20 (m, *J* = 7.4 Hz, 1H), 7.09–7.01 (m, 3H), 6.87 (m, 3H), 6.61 (t, *J* = 7.3 Hz, 1H), 6.51 (t, *J* = 7.3 Hz, 1H), 6.43 (d, *J* = 8.0 Hz, 2H), 6.11 (d, *J* = 8.0 Hz, 2H), 5.86 (s, 1H), 3.14–3.04 (m, 2H), 2.89 (m, 1H), 2.56 (m, 1H), 2.40 (m, 1H), 2.24 (m, 1H), 1.62–1.53 (m, 1H), 1,28–1.00 (m, 3H), 0.61 (t, *J* = 7.3 Hz, 3H); ^13^C{^1^H} NMR (CDCl_3_, 100 MHz): δ = 146.3, 146.2, 140.0, 138.9, 136.0, 135.0, 132.5, 132.2, 131.0, 130.4, 130.4, 129.1, 128.8, 128.5, 128.1, 127.9, 127.1, 126.5, 126.1, 125.9, 125.5, 125.2, 123.0, 121.9, 117.6, 117.2, 113.7, 113.1, 111.3, 47.3, 47.0, 32.3, 31.1, 23.2, 13.5; HRMS (ESI) *m*/*z* calcd for C_39_H_37_N_2_ [M + H]^+^: 533.2951, found: 533.2952. Single crystals of **5** were formed from crystallization with CDCl_3._

### General Procedure (GP3) for Benzanilide Cleavage

Tertiary anilide was dissolved in a 2:1 mixture of 1,4-dioxane:methanol (0.09 M) in a 20 mL scintillation vial. Sodium methoxide (10.0 equiv) was added, and the vial was sealed with a Teflon-lined cap and placed in a 130 °C J-KEM-Lab benchtop shaker until complete consumption of the starting material (as indicated by TLC). After cooling, the reaction mixture was diluted with 10 mL of EtOAc, transferred to a separatory funnel, and washed with 1 M aqueous HCl (4 × 5 mL) and brine (1 × 5 mL), and dried over anhydrous Na_2_SO_4_. After removal of solvent in vacuo, the crude mixture was purified by flash column chromatography to afford carboxylic acid product **8**.

#### (*E*)-5-Methoxy-2-(2-phenylhex-1-en-1-yl)benzoic acid (**8k**)

**8k** was synthesized from **4k** (75 mg, 0.15 mmol) following GP3 and obtained as a yellow crystalline solid (36 mg, 78%). Mp: 149–150 °C; chromatography conditions: 5–10% ethyl acetate in hexanes; ^1^H NMR (CDCl_3_, 400 MHz): δ = 7.65 (d, *J* = 2.8 Hz, 1H), 7.5 (m, 2H), 7.34–7.24 (m, 4H), 7.12 (dd, *J* = 8.5, 2.8 Hz, 1H), 7.04 (s, 1H), 3.88 (s, 3H), 2.53 (t, J = 7.6 Hz, 2H), 1.30–1.14 (m, 4H), 0.71 (t, *J* = 7.2 Hz, 3H); ^13^C{^1^H} NMR (CDCl_3_, 100 MHz): δ = 172.8, 158.3, 142.9, 141.3, 133.6, 132.3, 129.3, 128.4, 127.9, 127.0, 126.9, 119.5, 115.5, 55.7, 30.7, 29.7, 22.5, 13.9; HRMS (ESI) *m*/*z* calcd for C_20_H_23_O_3_ [M + H]^+^: 311.1642, found: 311.1646; C_20_H_21_O_3_ [M-H]^−^ 309.1496, found 309.1498. A single crystal of **8k** was obtained by recrystallization from 1:1 EtOAc/MeOH.

#### (*Z*)-2-(2-(4-Methoxyphenyl)-2-phenylvinyl)benzoic acid (**8o**)

**8o** was synthesized from **4o** (0.12 g, 0.23 mmol) following GP3 and obtained as a white solid (75 mg, 53%). Mp: 135–138 °C; chromatography conditions: 5–10% ethyl acetate in hexanes; ^1^H NMR (CDCl_3_, 400 MHz): δ = 8.00 (m, 1H), 7.38–7.16 (m, 8H), 7.01–6.98 (m, 3H), 6.74–6.70 (m, 2H), 3.76 (s, 3H); ^13^C{^1^H} NMR (CDCl_3_, 150 MHz): δ = 172.8, 158.9, 143.5, 143.0, 141.0, 132.4, 132.3, 132.3, 132.1, 131.2, 128.8, 128.5, 128.3, 127.7, 127.3, 126.5, 113.6, 55.3; HRMS (ESI) *m*/*z* calcd for C_22_H_19_O_3_ [M + H]^+^: 331.1329, found: 331.1332; C_22_H_17_O_3_ [M-H]^−^: 329.1183, found: 329.1183.

#### (*Z*)-5-Methoxy-2-(2-(4-methoxyphenyl)-2-phenylvinyl)benzoic acid (**8q**)

**8q** was synthesized from **4q** (70 mg, 0.13 mmol) following GP3 and obtained as a yellow solid (39 mg, 87%). Mp: 163–165 °C; chromatography conditions: 5–10% ethyl acetate in hexanes; ^1^H NMR (CDCl_3_, 400 MHz): δ = 7.51 (d, *J* = 2.8 Hz, 1H), 7.36–7.27 (m, 6H), 7.02–6.99 (m, 2H), 6.90 (d, *J* = 8.7 Hz, 1H), 6.77–6.72 (m, 3H), 3.79 (s, 3H), 3.77 (s, 3H); ^13^C{^1^H} NMR (CDCl_3_, 100 MHz): δ = 172.5, 158.8, 157.8, 143.7, 142.1, 133.5, 133.3, 132.6, 132.4, 129.7, 128.4, 128.2, 127.5, 127.0, 119.4, 114.9, 113.7, 55.5, 55.3; HRMS (ESI) *m*/*z* calcd for C_23_H_21_O_4_ [M + H]^+^: 361.1434, found: 361.1438; C_23_H_19_O_4_ [M-H]^−^: 359.1289, found: 359.1292.

#### 1,3-Diphenyl-2-(2-vinylphenyl)imidazolidine (**9a**)

Prepared from 2-vinylbenzaldehyde (0.9 g, 6.8 mmol)^43^ according to GP1 and obtained as an off-white solid (1.6 g, 73%). Mp: 109–110 °C; ^1^H NMR (CDCl_3_, 400 MHz): δ = 7.46 (d, *J* = 7.2 Hz, 1H); 7.33–7.16 (m, 8H), 6.77 (td, *J* = 7.3 Hz, 0.9 Hz, 2H), 6.70 (m, 4H), 6.16 (s, 1H), 5.66 (dd, *J* = 17.3 Hz, 1.3 Hz, 1H), 5.35 (dd, *J* = 10.9 Hz, 1.3 Hz, 1H), 3.84 (m, 2H), 3.64 (m, 2H); ^13^C{^1^H} NMR (CDCl_3_, 100 MHz): δ = 146.9, 138.2, 137.6, 134.8, 129.2, 128.3, 128.0, 127.9, 126.9, 118.9, 117.0, 115.1, 75.7, 48.0; HRMS (ESI) *m*/*z* calcd for C_23_H_23_N_2_ [M + H]^+^: 327.1856, found: 327.1849.

#### 1,3-Dimethyl-2-(2-vinylphenyl)-2,3-dihydro-1*H*-benzo[*d*]imidazole (**9b**)

2-Vinylbenzaldehyde (1.1 g, 8.3 mmol)^43^ was added to a solution of *N*^1^,*N*^2^-dimethylbenzene-1,2-diamine (1.25 g, 9.2 mmol)^44^ in methanol (10 mL). One drop of glacial acetic acid was added, and the reaction mixture was stirred vigorously for 30 min. The reaction vessel was cooled in ice bath, and the product precipitate was collected by filtration, washed with cold methanol, and then recrystallized from ethanol to afford **9b** as a white solid (1.4 g, 67%). Mp: 92–93 °C; ^1^H NMR (CDCl_3_, 300 MHz): δ = 7.60–7.55 (m, 2H), 7.14–7.29 (m, 3H), 6.72 (m, 2H), 6.42 (m, 2H), 5.60 (dd, *J* = 17.3, 1.5 Hz, 1H), 5.28 (s, 1H), 5.22 (dd, *J* = 11.0, 1.3 Hz, 1H), 2.54 (s, 6H); ^13^C{^1^H} NMR (CDCl_3_, 100 MHz): δ = 142.2, 139.3, 135.3, 134.3, 130.3, 129.3, 127.9, 126.7, 119.4, 115.7, 105.7, 91.7, 33.2; HRMS (ESI) *m*/*z* calcd for C_17_H_19_N_2_ [M + H]^+^: 251.1543, found: 251.1540.

#### *N*-Methyl-*N*-(2-(methylamino)phenyl)-2-phenethylbenzamide (**10**)

Aminal **9b** (0.17 g, 0.68 mmol) and iodobenzene (0.22 mL, 2 mmol) were combined in DMF (0.4 M) in an oven-dried 5 mL round-bottom flask and deoxygenated for 30 min with a stream of Ar. [Pd(allyl)Cl]_2_ (12 mg, 5 mol %) and tBuXPhos (28.5 mg, 10 mol %) were added, and the reaction mixture was stirred at room temperature for 16 h until complete consumption of the starting material as indicated by TLC. Five milliliters of 10% aqueous NaOH solution was then added to the reaction, and the mixture was heated in an 80 °C oil bath for 3 h. The reaction was then allowed to cool to room temperature, filtered through a short Celite plug, and extracted with ethyl acetate (3 × 10 mL). The combined extracts were washed with brine (1 × 10 mL) and dried over anhydrous Na_2_SO_4_. Filtration and evaporation of the solvent gave a crude material that was purified by flash column chromatography on deactivated silica gel using 10–15% EtOAc in hexanes as the eluent to afford **10** as a white solid (mixture of rotamers, 135 mg, 65%). Mp: 125–127 °C; NMR (CDCl_3_, 400 MHz): δ = 7.36–7.2 (m, 5H), 7.12–7.09 (m, 2H), 7.04–6.80 (m, 3H), 6.70 (dd, *J* = 7.7 Hz, 1.39 Hz, 1H), 6.53 (d, *J* = 8.13 Hz, 1H), 6.39 (t, *J* = 7.55 Hz, 1H), 3.97 (d, *J* = 5.1 Hz, 1H), 3.33 (s, 3H), 3.09–2.90 (m, 4H), 2.87 (d, *J* = 5.1 Hz, 3H); ^13^C{^1^H} NMR (CDCl_3_, 100 MHz): δ = 172.5, 144.6, 142.2, 139.0, 135.9, 129.4, 129.2, 129.1, 129.0, 128.7, 128.5, 128.3, 126.1, 125.7, 125.2, 116.7, 110.8, 37.6, 36.0, 35.6, 30.3; HRMS (ESI) *m*/*z* calcd for C_23_H_25_ON_2_ [M + H]^+^: 345.1961, found: 345.1955.

## Supporting Information Available

Selected experimental details; X-ray crystallographic data; NMR spectra (PDF)

## Accession Codes

CCDC 2160100–2160103 contain the supplementary crystallographic data for this paper. These data can be obtained free of charge via www.ccdc.cam.ac.uk/data_request/cif, or by emailing data_request@ccdc.cam.ac.uk, or by contacting The Cambridge Crystallographic Data Centre, 12 Union Road, Cambridge CB2 1EZ, UK; fax: +44 1223 336033.
