# Supplementary material for: Imidazolidine Hydride Donors in Palladium-Catalyzed Alkyne Hydroarylation
Source: J Org Chem. 2022 Jun 1;87(12):8059–70. doi: 10.1021/acs.joc.2c00725 (PMC9490851; doi:10.1021/acs.joc.2c00725)
Supplement: Supplementary file 1 — jo2c00725_si_001.pdf [file jo2c00725_si_001.pdf]

Supplementary Information For

"Imidazolidine Hydride Donors in Palladium-Catalyzed Alkyne Hydroarylation"

Soe L. Tun,† S. V. Santhana Mariappan,†,‡ and F. Christopher Pigge*†

†Department of Chemistry, University of Iowa, Iowa City, Iowa, 52242, USA
‡Central NMR Facility, University of Iowa, Iowa City, Iowa, 52242, USA

Table of Contents:

Structures of Starting Materials (**S1-S12**) and **6**	S2
Experimental Procedures for Synthesis of **3t-3x, 3a-*d*, 3c-*d*, and 3s-*d***	S3-S5
References	S6
Single Crystal X-Ray Diffraction Data of **5, 4j, 4s, 8k**	S7-S10
2D NMR Analysis of **4c**	S11-S17
2D NMR Analysis of **4o**	S18-S19
2D NMR Analysis of **4s & 4s-*d***	S20-S24
^{1}H and ^{13}C NMR of All New Compounds	S25-S115

Structures of Starting Materials S1-S12 and 6

All starting materials **S1-S12** and compound **6** were prepared according to literature procedure.[1-8]

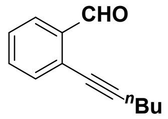

S1[1]

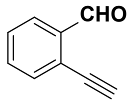

S2[1]

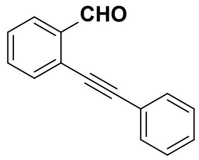

S3[2]

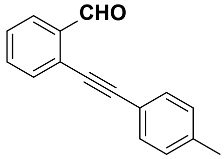

S4[3]

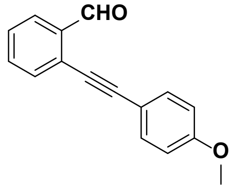

S5[2]

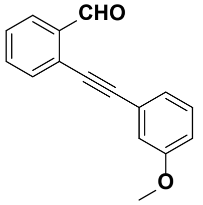

S6[2]

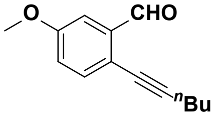

S7[4]

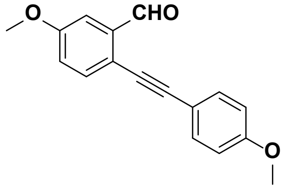

S8[5]

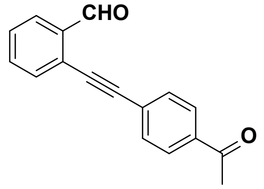

S9[7]

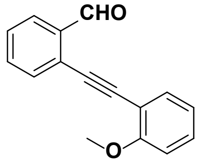

S10[2]

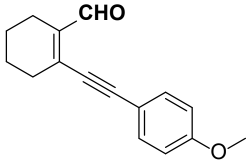

S11[6]

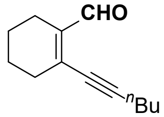

S12[7]

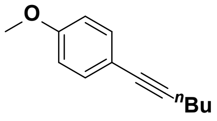

6[8]

Experimental Procedures for Synthesis of Compounds 3t-3x

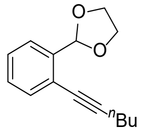

2-(2-(hex-1-yn-1-yl)phenyl)-1,3-dioxolane (3t). **S1** (3.0 mmol) was added to the solution of ethylene glycol (30.0 mmol), $CH(OMe)_3$ (0.495 mL, 4.5 mmol), and TBATB (14.5 mg, 0.03 mmol) in THF (3 mL), and the reaction mixure was stirred at room temperature overnight. The reaction mixture was then concentrated *in vacuo* and crude reaction mixture was purified using flash column chromatography using 0.5-3% EtOAc in hexane after deactivating silica gel (0.4 g, 58%) to afford **3t** as pale yellow oil; ^{1}H NMR ($CDCl_3$, 300 MHz) δ = 7.57-7.51 (m, 1H), 7.44-7.38 (m, 1H), 7.33-7.24 (m, 2H), 6.2 (s, 1H), 4.22-4.00 (m, 4H), 2.45 (t, J = 6.9 Hz, 2H), 1.66-1.43 (m, 4H), 0.95 (t, J = 7.2 Hz, 3H); $^{13}C\{^1H\}$ NMR ($CDCl_3$, 100 MHz) δ = 138.7, 132.5, 128.9, 127.7, 125.9, 123.5, 102.0, 95.3, 77.8, 65.5, 30.8. 22.0, 19.3, 13.7; HRMS (ESI) *m/z* Calcd for $C_{15}H_{19}O_2$ $[M+H]^+$ 231.1380, found 231.1378.

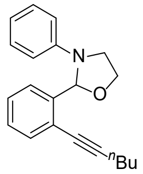

2-(2-(hex-1-yn-1-yl)phenyl)-3-phenyloxazolidine (3u). 2-(phenylamino)ethan-1-ol[9] (0.44 g, 3.2 mmol) was added to the solution of **S1** (0.5 g, 2.68 mmol) in water (4M). $Cu(OTf)_2$ (11mg, 1 mol%) was added, and the reaction mixture was stirred vigorously until complete consumption of **S1** as indicated by TLC (1.5 hr). The reaction was quenched with saturated $NaHCO_3$ (10 mL), and the mixture was extracted with diethyl ether (4 x 10 mL). The combined organic extracts were washed with brine (1 x 10 mL), and dried over anhydrous Na_2SO_4. The dried organic phase was then concentrated *in vacuo*, and the crude product was purified by flash column chromatography using 0.5% EtOAc in hexane as an eluent system after deactivating silica gel (0.38 g, 46%) to afford **3u** as pale yellow oil; ^{1}H NMR ($CDCl_3$, 300 MHz) δ = 7.49 (dd, J = 7.4 Hz, 1.4 Hz, 1H), 7.36 (dd, J = 7.6 Hz, 1.4 Hz, 1H), 7.27 (m, 1H), 7.23 (t, J = 2.0 Hz, 1H), 7.21-7.15 (m, 2H), 6.73 (m, J = 7.3 Hz, 1H), 6.49 (dd, J = 7.7 Hz, 1.2 Hz, 2H), 6.35 (s, 1H), 4.18 (m, 2H), 3.85 (m, 1H), 3.60 (m, 1H), 2.49 (t, J = 7.0 Hz, 2H), 1.66-1.46 (m, 4H), 0.94 (t, J = 7.2 Hz, 3H); $^{13}C\{^1H\}$ NMR ($CDCl_3$, 100 MHz) δ = 145.5, 140.6, 133.1, 129.3, 128.7, 127.9, 126.5, 123.8, 117.6, 113.2, 96.5, 89.9, 78.0, 65.3, 48.3, 31.0, 22.2, 19.5, 13.8; HRMS (ESI) *m/z* Calcd for $C_{21}H_{24}ON$ $[M+H]^+$ 306.1852, found 306.1847.

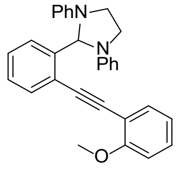

2-(2-((2-methoxyphenyl)ethynyl)phenyl)-1,3-diphenylimidazolidine (3v). **3v** was prepared from **S10** (0.25 g, 1.04 mmol) following GP1 (0.39 g, 89%), and obtained as a white solid. Mp – 158-159°C; ^{1}H NMR ($CDCl_3$, 400 MHz) δ = 7.50 (m, J = 7.6 Hz, 2H), 7.35-7.27 (m, 2H), 7.20-7.11 (m, 6H), 6.95-6.89 (m, 6H), 6.70-6.66 (m, 3H), 4.13-4.04 (m, 2H), 3.85-3.76 (m, 5H); $^{13}C\{^1H\}$

NMR ($CDCl_3$, 100 MHz) δ = 160.5, 145.6, 143.9, 133.3, 132.1, 130.1, 129.1, 127.8, 127.5, 123.9, 120.6, 117.7, 113.9, 112.5, 110.8, 92.1, 91.8, 74.3, 55.8, 46.8; HRMS (ESI) *m/z* Calcd for $C_{30}H_{27}ON_2$ $[M+H]^+$ 431.2118, found 431.2121.

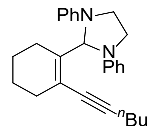

2-(2-(hex-1-yn-1-yl)cyclohex-1-en-1-yl)-1,3-diphenylimidazolidine (3w). **3w** was prepared from **S12** (1.0 g, 5.3 mmol) according to slight modification of GP1. After stirring the reaction mixture overnight, it was concentrated *in vacuo*, and purified using 0.5% EtOAc in hexane after deactivating the silica gel (1.98g, 99%) to afford **3u** as an orange oil; ^{1}H NMR ($CDCl_3$, 400 MHz) δ = 7.32 (m, 4H), 6.96 (m, J = 7.9 Hz, 4H), 6.83 (m, J = 7.3, 2H), 6.21 (s, 1H), 3.84 (m, 2H), 3.75 (m, 2H), 2.59 (t, J = 7.0 Hz , 2H), 2.22 (s, 2H), 1.81 (s, 2H), 1.72 (m, 2H), 1.56 (m, 2H), 1.44 (m, 4H), 1.01 (t, J = 7.1 Hz, 3H) ; $^{13}C\{^1H\}$ NMR ($CDCl_3$, 100 MHz) δ = 145.5, 140.6, 129.0, 122.0, 117.1, 113.5, 97.0, 80.5, 75.5, 46.6, 31.2, 30.9, 23.0, 22.2, 21.9, 19.4, 13.7; HRMS (ESI) *m/z* Calcd for $C_{27}H_{33}N_2$ $[M+H]^+$ 385.2638, found 385.2636.

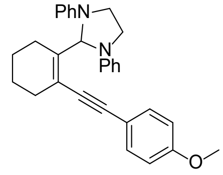

2-(2-((4-methoxyphenyl)ethynyl)cyclohex-1-en-1-yl)-1,3-diphenylimidazolidine (3x). **3x** was prepared from **S11** (0.5 g, 4.2 mmol) following GP1 (1.6 g, 89%), and obtained as a white solid. Mp – 99-101°C; ^{1}H NMR ($CDCl_3$, 400 MHz) δ = 7.49 (m, J = 8.8 Hz, 2H), 7.28-7.23 (m, 4H), 6.94 (m, 4H), 6.88-6.84 (m, J = 8.8 Hz, 2H), 6.75 (t, J = 7.3 Hz, 2H), 6.25 (s, 1H), 3.84-3.77 (m, 5H), 3.76-3.69 (m, 2H), 2.27 (m, 2H), 1.81 (m, 2H), 1.43 (m, 4H); $^{13}C\{^1H\}$ NMR ($CDCl_3$, 100 MHz) δ = 159.7, 145.4, 142.0, 132.8, 129.0, 121.5, 117.1, 115.6, 114.1, 113.4, 95.7, 87.9, 77.4, 75.6, 55.3, 46.7, 30.5, 23.2, 22.1, 21.8; HRMS (ESI) *m/z* Calcd for $C_{30}H_{31}ON_2$ $[M+H]^+$ 435.2431, found 435.2423.

Experimental Procedure for synthesis of deuterium-labelled compounds (3a-*d,* 3c-*d,* 3s-*d*)

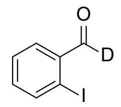

2-iodobenzaldehyde-*formyl-d* (**1a-*d***): 2-iodobenzaldehyde (0.3 g, 1.29 mmol) was added to the solution of $NaBD_4$ in methanol (2.5 mL), and the reaction mixture was stirred at room temperature overnight. The reaction was quenched with water and the mixture was extracted with EtOAc (3 x 10 mL). The combined extracts were washed with brine (1 x 10 mL) and dried over anhydrous Na_2SO_4. Filtration and removal of the solvent afforded crude benzyl alcohol derivative (0.25 g, 83%) that was used directly in the next step.

PCC (0.28 g, 1.32 mmol) was added to the flask containing crude benzyl alcohol (0.24 g, 1.02 mmol) dissolved in dichloromethane (2 mL), and the reaction mixture was stirred until complete consumption of the starting material as indicated by TLC. The reaction mixture was filtered through Celite plug, concentrated *in vacuo*, and the crude product purified by flash column chromatography

using 5% EtOAc in hexanes as the eluent to get 2-iodobenzaldehyde-*formyl-d* (**1a-*d***) as white solid (0.15 g, 63%).

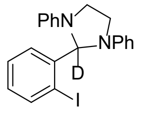

2-(2-Iodophenyl)-1,3-diphenylimidazolidine-2-*d* (**3a-*d***)**: 1a-*d*** (0.14 g, 0.6 mmol) was added to a solution of N,N'-diphenylethylenediamine (0.13 g, 0.63 mmol) in methanol (1.5 mL). One drop of glacial acetic acid was added and the reaction mixture was stirred vigorously for 16 h at room temperature. The product precipitate was collected by filtration and washed with cold methanol. The crude solid was recrystallized from EtOAc/methanol to give **3a-*d*** as a white solid (0.16 g, 64%, H:D = 15:85).

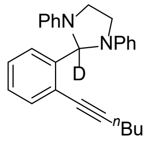

2-(2-(Hex-1-yn-1-yl)phenyl)-1,3-diphenylimidazolidine-2-*d* (**3c-*d***)**: 1a-*d*** (0.21g, 0.88 mmol) was dissolved in triethylamine (2 mL) and the solution deoxygenated for 10 minutes with a stream of Ar. $Pd(PPh_3)_4$ (50 mg, 5 mol%) and CuI (17 mg, 10 mol%) was added followed by dropwise addition of 1-hexyne (0.12 mL, 1.05 mmol). The reaction mixture was stirred in a 50 ºC oil bath until consumption of the starting aldehyde as indicated by TLC. After cooling, the mixture was filtered through short Celite plug, concentrated *in vacuo*, and purified by flash column chromatography using 0-2% EtOAc in hexane as the eluent system to give 2-(1-hexynyl)benzaldehyde-*formyl-d* as orange oil (0.16 g, 79%), which was used directly in the next step.
2-(1-Hexynyl)benzaldehyde-*formyl-d* (0.13 g, 0.69 mmol) was added to the solution of N,N'-diphenylethylenediamine (0.15 g, 0.69 mmol) in methanol (2.3 mL). One drop of glacial acetic acid was added, and the reaction mixture was stirred vigorously overnight at room temperature. The product precipitate was collected by filtration and washed with cold methanol. The crude solid was recrystallized from EtOAc/methanol to give **3c-*d*** as a white solid (0.21 g, 82%, H:D = 28:72)

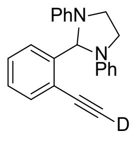

2-(2-(Ethynyl-d)phenyl)-1,3-diphenylimidazolidine (**3s-*d***)**: 3s** (120 mg, 0.37 mmol) was added to an oven-dried round bottomed flask containing dry THF (3.7 mL, 0.1 M) and the mixture cooled to -78 ºC in a dry ice/acetone bath. nBuLi (0.22 mL, 0.55 mmol) was then added dropwise. Once the addition was complete, the ice bath was removed and the reaction was allowed to warm to room temperature and stirred for 1 h. D_2O (2 mL) was then added and stirring continued overnight. The mixture was then extracted with ethyl acetate (3 x 5 mL) and the combined extracts dried over anhydrous Na_2SO_4. Filtration and concentration *in vacuo* afforded crude product that was recrystallized from EtOAc to give **3s-*d*** as a white solid (0.118 g, 98%, ~98% D).

References:

1. Bhunia, S.; Wang, K.-C.; Liu, R.-S., *Angew. Chem. Int. Ed.* **2008,** *47* (27), 5063-5066.
2. Too, P. C.; Chiba, S., *Chem. Commun.* **2012,** *48* (61), 7634-7636.
3. Tokimizu, Y.; Ohta, Y.; Chiba, H.; Oishi, S.; Fujii, N.; Ohno, H., *Tetrahedron* **2011,** *67* (29), 5168-5175.
4. Park, J. H.; Bhilare, S. V.; Youn, S. W., *Org. Lett.* **2011,** *13* (9), 2228-2231.
5. Mahoney, S. J.; Moon, D. T.; Hollinger, J.; Fillion, E., *Tetrahedron Lett.* **2009,** *50* (33), 4706-4709.
6. Zhang, Y.; Herndon, J. W., *Org. Lett.* **2003,** *5* (12), 2043-2045.
7. Yamada, T.; Park, K.; Tachikawa, T.; Fujii, A.; Rudolph, M.; Hashmi, A. S. K.; Sajiki, H., *Org. Lett.* **2020,** *22* (5), 1883-1888.
8. Lu, B.; Li, C.; Zhang, L., *J. Am. Chem. Soc.* **2010,** *132* (40), 14070-14072.
9. Igarashi, T.; Tayama, E.; Iwamoto, H.; Hasegawa, E., *Tetrahedron Lett.* **2013,** *54* (50), 6874-6877.

Single-crystal X-ray diffraction data

Single-crystal X-ray diffraction data were collected on a Bruker D8 Venture Duo diffractometer using MoKα radiation (λ=0.71073 Å) with APEX II detector. Crystals were mounted in paratone oil on a Mitegen magnetic mount. Lorentz and polarization corrections with programs from the APEXII package were used for data reduction. Structure solution and refinement were completed using SHELXT and SHELXL, respectively within the Olex2 graphical user interface. Non-hydrogen atoms were refined anisotropically. Hydrogen atoms were positioned geometrically and refined using a riding model. The total potential solvent accessible volume was calculated by PLATON using a spherical probe (7.2 Å, probe radius: 1.20 Å, approximate grid spacing: 0.2 Å).

Table S1. Crystallographic parameters for **5** and **4j**.

Compound name	**5**	**4j**
CCDC deposition number	2160100	2160101
Empirical formula	$C_{39}H_{36}N_2$	$C_{35}H_{36}N_2O_3$
Formula weight	532.70	532.66
Temperature/K	150.15	150.0
Crystal system	orthorhombic	orthorhombic
Space group	$Pca2_1$	*Pbca*
a/Å	14.5544(14)	9.320(2)
b/Å	10.5595(11)	22.246(9)
c/Å	37.136(3)	28.567(13)
α/°	90	90
β/°	90	90
γ/°	90	90
Volume/Å^3	5707.3(9)	5923(4)
Z	8	8
ρ_{calc}g/cm^3	1.240	1.195
μ/mm^{-1}	0.543	0.076
F(000)	2272.0	2272.0
Crystal size/mm^3	0.050 × 0.060 × 0.275	0.2 × 0.13 × 0.06
Radiation	CuKα (λ = 1.54178)	MoKα (λ = 0.71073)
2Θ range for data collection/°	4.758 to 151.17	3.93 to 50
Index ranges	$-18 \le h \le 17$, $-13 \le k \le 13$, $-46 \le l \le 46$	$-11 \le h \le 10$, $-26 \le k \le 26$, $-33 \le l \le 33$
Reflections collected	86001	102136
Independent reflections	11723 [R_{int} = 0.0519, R_{sigma} = 0.0361]	5215 [R_{int} = 0.0580, R_{sigma} = 0.0190]
Data/restraints/parameters	11723/1/741	5215/0/363
Goodness-of-fit on F^2	1.049	1.039
Final R indexes [I>=2σ (I)]	R_1 = 0.0361, wR_2 = 0.0966	R_1 = 0.0385, wR_2 = 0.0892
Final R indexes [all data]	R_1 = 0.0373, wR_2 = 0.0980	R_1 = 0.0495, wR_2 = 0.0966
Largest diff. peak/hole / e Å^{-3}	0.16/-0.18	0.24/-0.27

Table S2. Crystallographic parameters for **4s** and **8k**.

Compound name	**4s**	**8k**
CCDC deposition number	2160102	2160103
Empirical formula	$C_{29}H_{26}N_{2}O$	$C_{20}H_{22}O_{3}$
Formula weight	418.52	310.37
Temperature/K	150.15	150.15
Crystal system	triclinic	tetragonal
Space group	*P*-1	$P4_2/n$
a/Å	7.8415(8)	21.5060(10)
b/Å	10.8486(11)	21.5060(10)
c/Å	14.4448(14)	7.1371(7)
α/°	70.509(5)	90
β/°	78.800(5)	90
γ/°	88.821(5)	90
Volume/Å^3	1135.0(2)	3301.0(4)
Z	2	8
ρ_{calc}g/cm^3	1.225	1.249
μ/mm^{-1}	0.074	0.083
F(000)	444.0	1328.0
Crystal size/mm^3	0.17 × 0.165 × 0.05	0.23 × 0.17 × 0.035
Radiation	MoKα (λ = 0.71073)	MoKα (λ = 0.71073)
2Θ range for data collection/°	4.128 to 56.62	5.358 to 52.84
Index ranges	-10 ≤ h ≤ 10, -14 ≤ k ≤ 14, -19 ≤ l ≤ 19	-26 ≤ h ≤ 26, -26 ≤ k ≤ 26, -8 ≤ l ≤ 8
Reflections collected	28554	58687
Independent reflections	5608 [R_{int} = 0.0355, R_{sigma} = 0.0227]	3376 [R_{int} = 0.0420, R_{sigma} = 0.0173]
Data/restraints/parameters	5608/0/289	3376/0/214
Goodness-of-fit on F^2	1.060	1.036
Final R indexes [I>=2σ (I)]	R_1 = 0.0452, wR_2 = 0.1094	R_1 = 0.0384, wR_2 = 0.0968
Final R indexes [all data]	R_1 = 0.0555, wR_2 = 0.1190	R_1 = 0.0457, wR_2 = 0.1012
Largest diff. peak/hole / e Å^{-3}	0.36/-0.31	0.20/-0.18

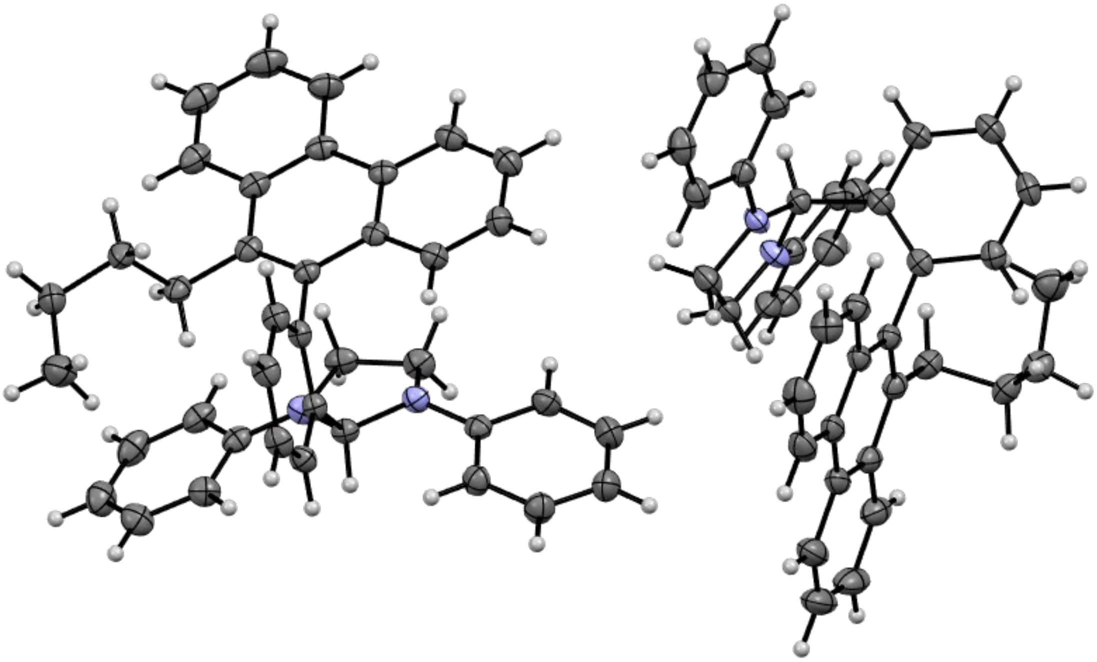

Figure S1. ORTEP plot of **5** (thermal ellipsoids shown at 50% probability).

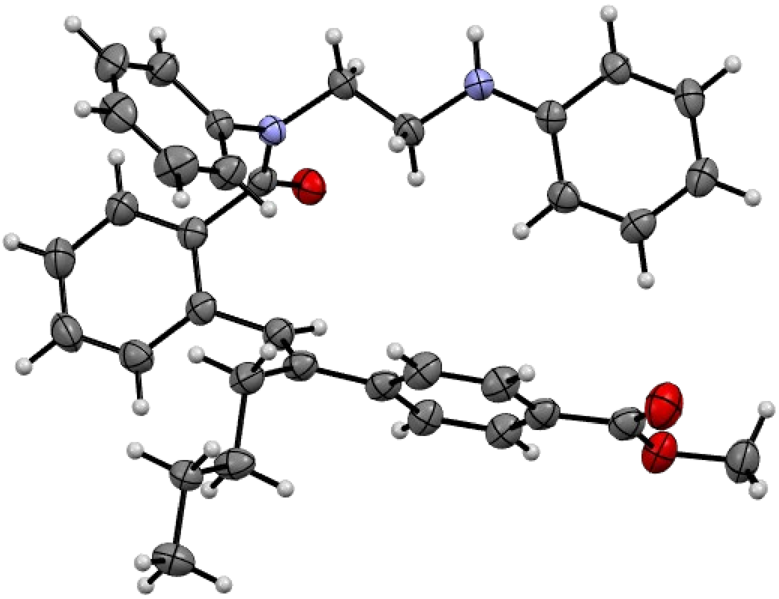

Figure S2. ORTEP plot of **4j** (thermal ellipsoids shown at 50% probability).

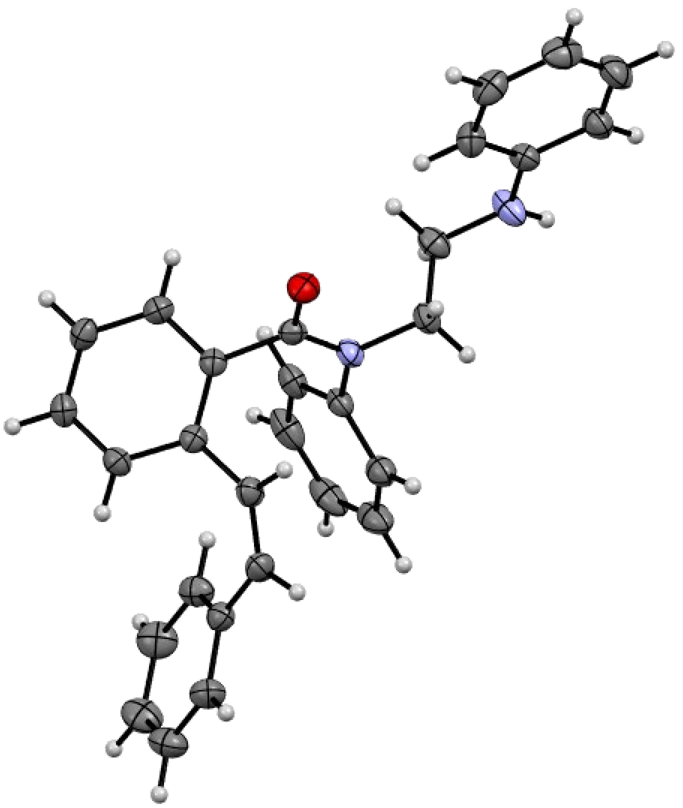

Figure S3. ORTEP plot of **4s** (thermal ellipsoids shown at 50% probability).

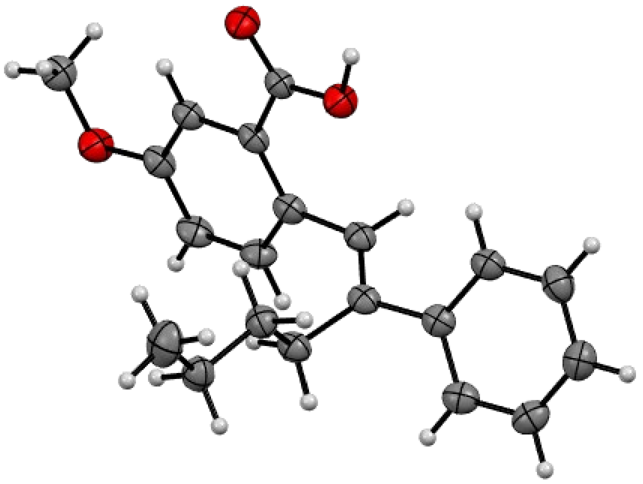

Figure S4. ORTEP plot of **8k** (thermal ellipsoids shown at 50% probability).

NMR spectroscopic analysis

The NMR analysis of the final products 4c, 4n, 4q and 4q-d were performed by a battery of one- and two-dimensional (1D and 2D) homonuclear and 1H-13C heteronuclear experiments [1H, 13C, 2D correlated spectroscopy (COSY), nuclear Overhauser effect spectroscopy (NOESY), heteronuclear single quantum coherence (HSQC), and heteronuclear multiple bond correlation (HMBC)] using NMR spectrometers operating at the proton frequencies of 400-600 MHz. The experiments on 400-Neo spectrometer used a nitrogen-cooled Prodigy cryoprobe, whereas 500-Neo and AVANCE-III-600 spectrometers used BBFO probes. Gradient-assisted versions of the pulse sequences and inverse detection were used for these 2D experiments. 1H spectral widths and 90o pulse widths are optimized for each sample. A recycle delay (D1) of 4.0s was used in all the 2D experiments. An optimal range of mixing times for NOESY experiments was determined using the one-dimensional version of spin-lattice relaxation measurements. Typical parameters for the NMR experiments were as follows: 1H [time domain data points (TD), 32k; NS, 32], 13C (TD, 64k; NS, 10k), 2D COSY (TD, 4k; TD1, 512; NS, 8; DS, 32), NOESY [TD, 4k; TD1, 300; NS, 16; DS, 64; mixing times, 0.05, 0.2, 0.5, 0.7, and 1.0s], 13C-1H HSQC (TD, 2k; TD1, 256; NS, 16; DS, 128) and 13C-1H HMBC (TD, 4k; TD1, 256; NS, 32; DS, 64). TD, NS, and DS refer to time domain data points, number of scans, and dummy scans, respectively. All NMR data were processed with TOPSPIN 3.6.1 suite of software programs. The 1D 1H data were processed with zero-filling to 64k data points and 0.3 Hz exponential line broadening, whereas 13C spectra were processed with zero-filling to 128k data points and 1.0 Hz of exponential line broadening. The 2D NMR data were processed with the zero-filling to 4,096 points and 1,024 points in acquisition and second dimension, respectively.

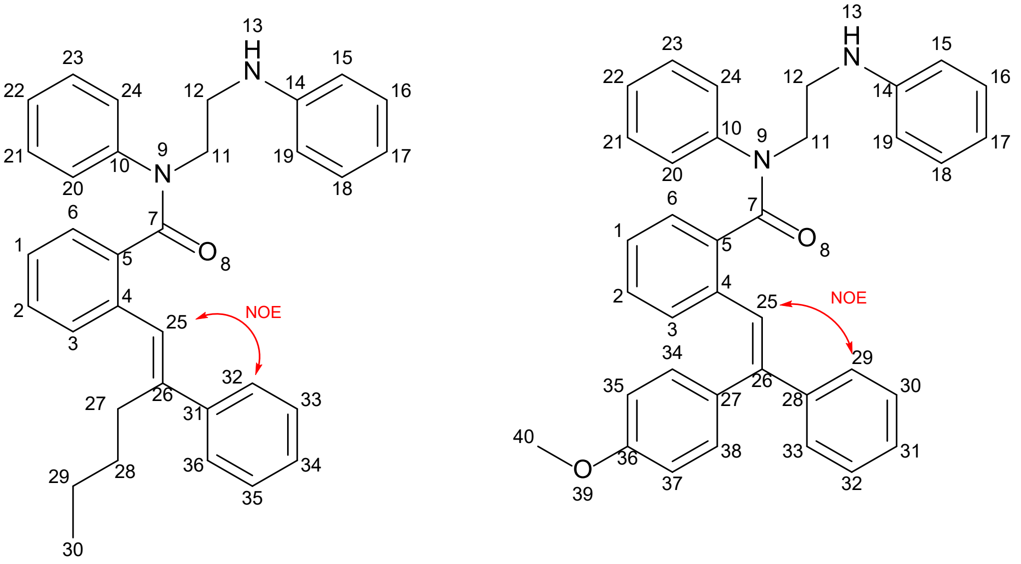

Figure S5. Structure of **4c** (left) and **4o** (right) with numbering.

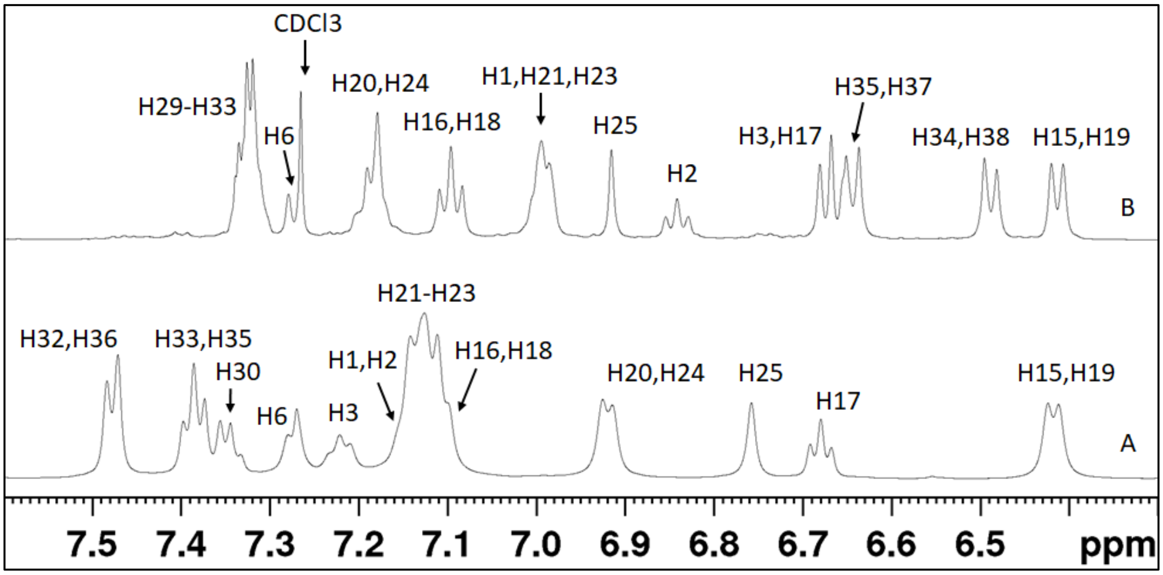

Figure S6. ^{1}H NMR sections of aromatic protons for A) **4c**, and B) **4o**. The resonance assignments were performed by using ^{1}H, ^{13}C, ^{13}C-DEPT, ^{1}H-^{1}H COSY, ^{1}H-^{13}C HSQC and ^{1}H-^{13}C HMBC data, the proton atom positions are marked accordingly. The unique NOE correlations observed in ^{1}H-^{1}H NOESY denoted by an arrow in structures (Figure S5) reveal that the olefinic proton and the phenyl ring are in *trans* orientation.

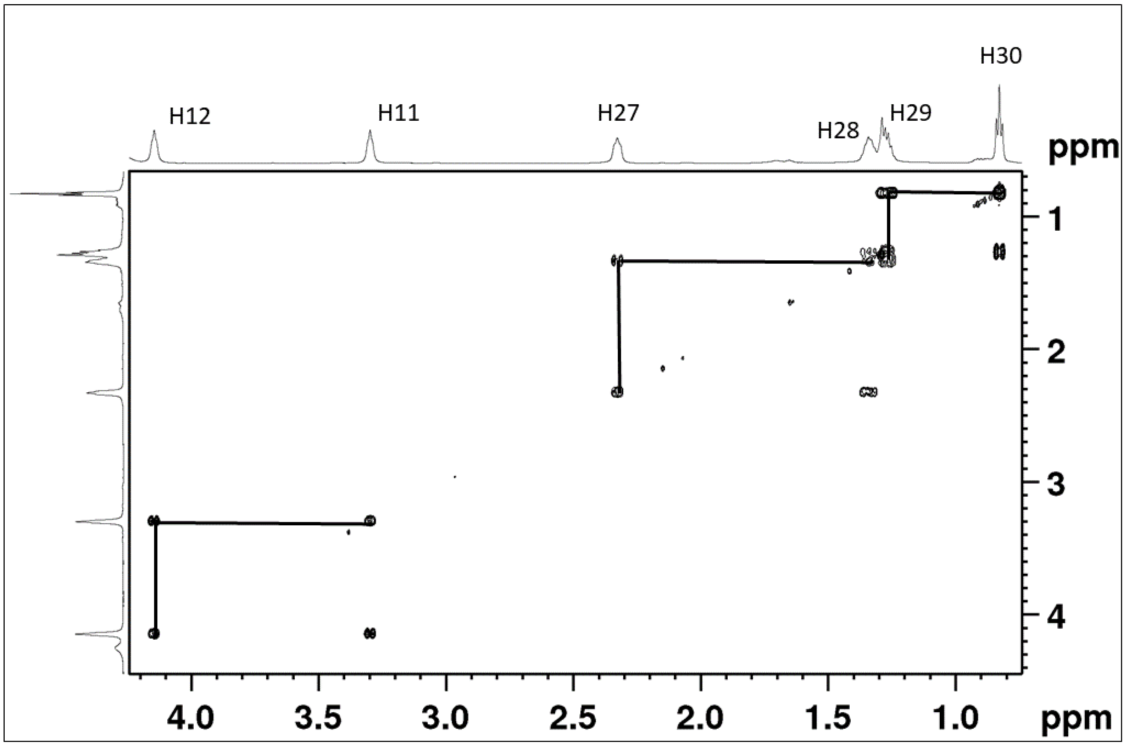

Figure S7. ^{1}H-^{1}H COSY cross-section of aliphatic protons for **4c**. The resonance assignments are marked accordingly.

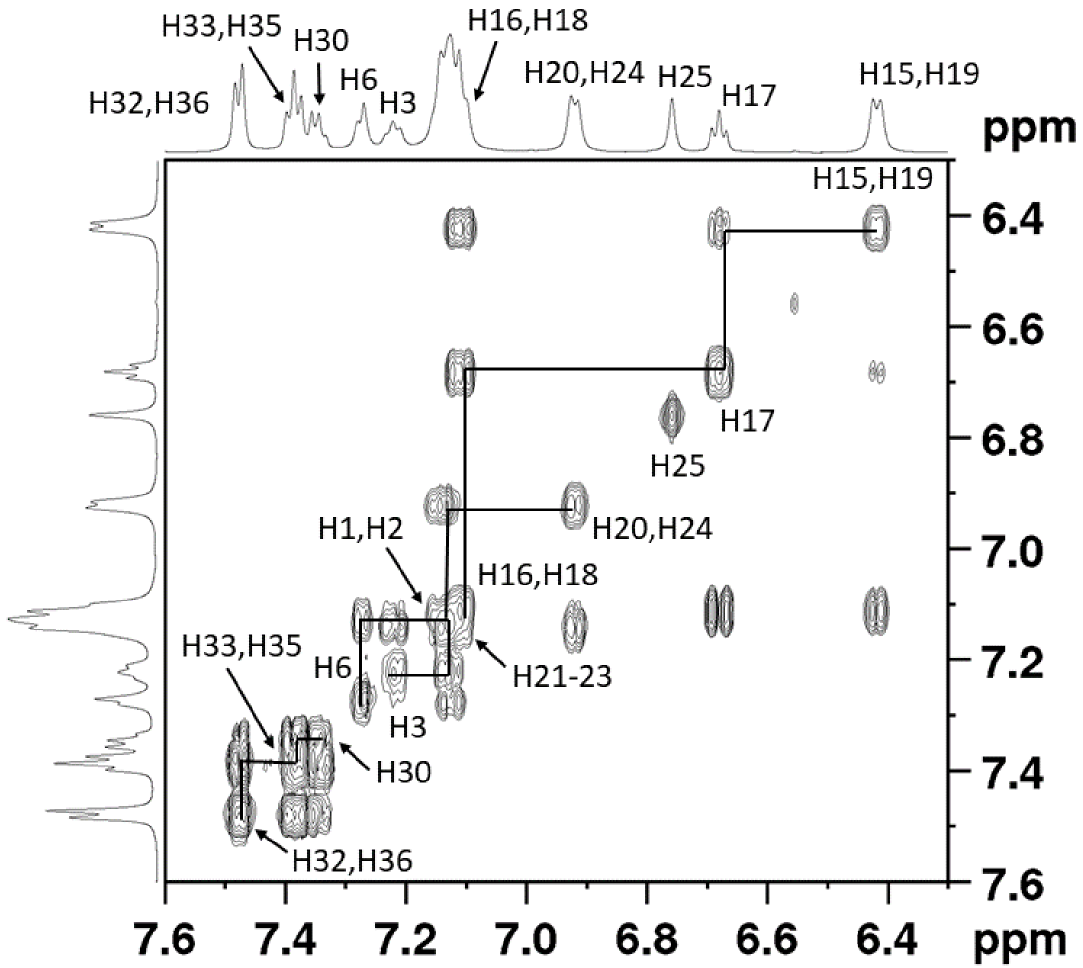

Figure S8. ^{1}H-^{1}H COSY cross-section of aromatic protons for **4c**. The resonance assignments are marked accordingly.

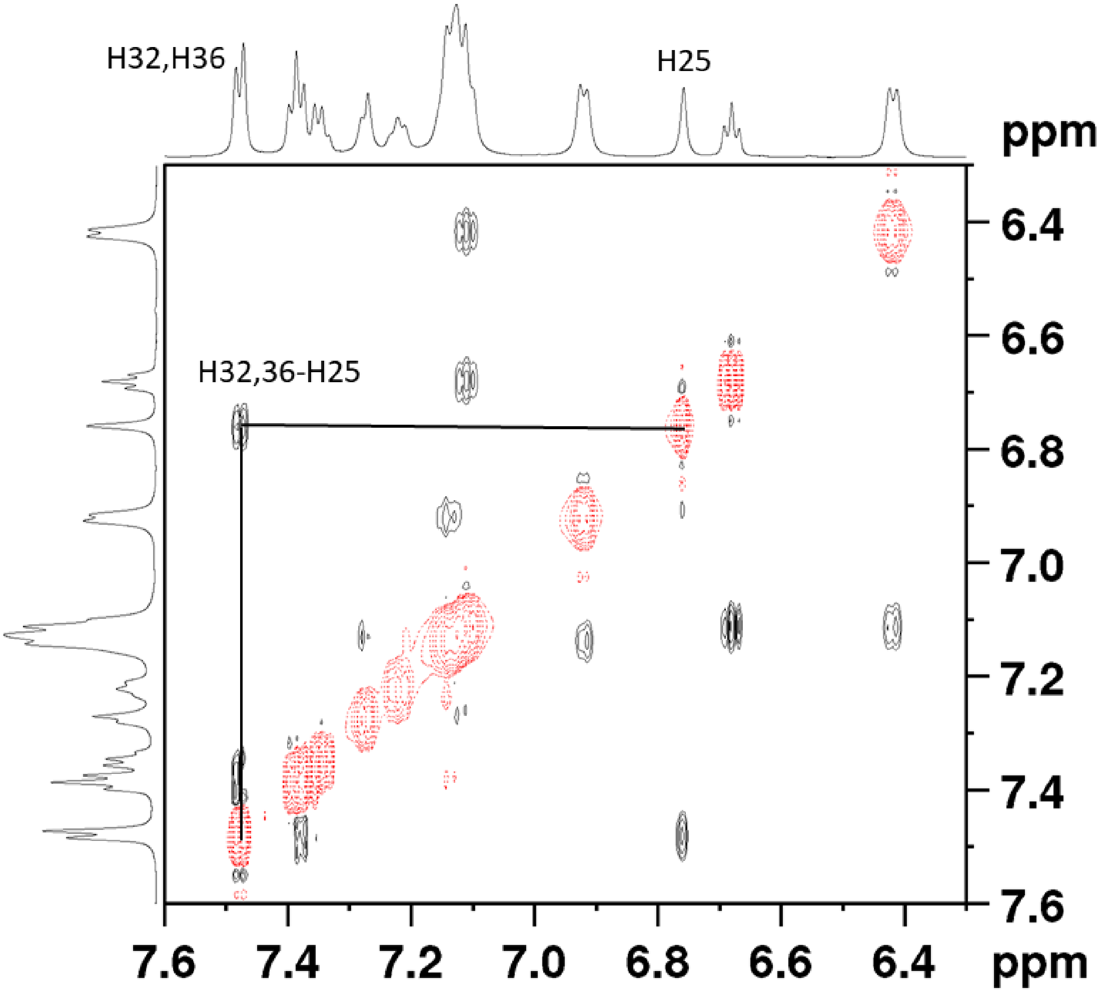

Figure S9. ^{1}H-^{1}H NOESY cross-section for **4c**. The NOE between the olefinic proton and the aromatic H32/H36 is marked. NOE mixing time=2.0s.

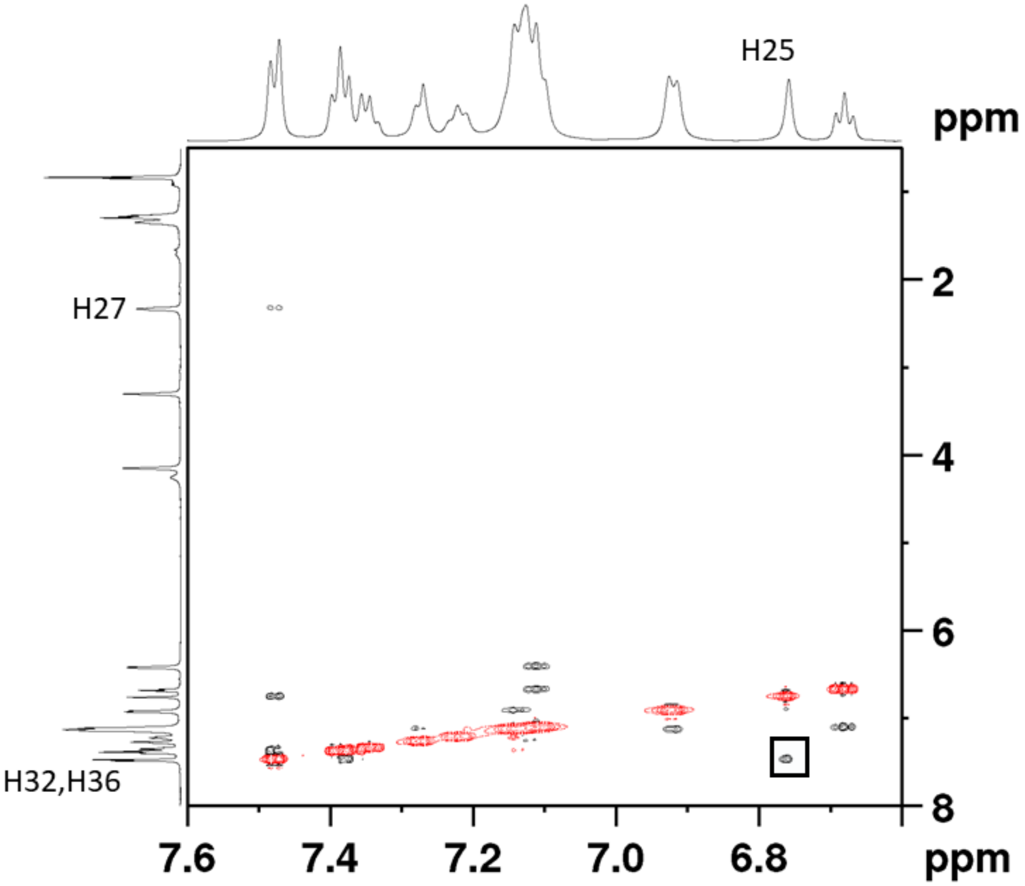

Figure S10. ^{1}H-^{1}H NOESY cross-section for **4c**. A strong NOE between the olefinic (H25) and the aromatic H32/H36 and the absence of NOE between H25 and H27 in the mixing time range of 0.05-2.0 s indicate that the butyl group and the aromatic ring are oriented *trans* and *cis* to H25 respectively. NOE mixing time=2.0 s. This is consistent with the bond distances observed in the X-ray crystal structure of **4j** where $r_{H25\text{-}H27\ (average)}$ is 3.71 Å, $r_{H25\text{-}H32}$ 2.27 Å and $r_{H25\text{-}H36}$ 4.16 Å respectively.

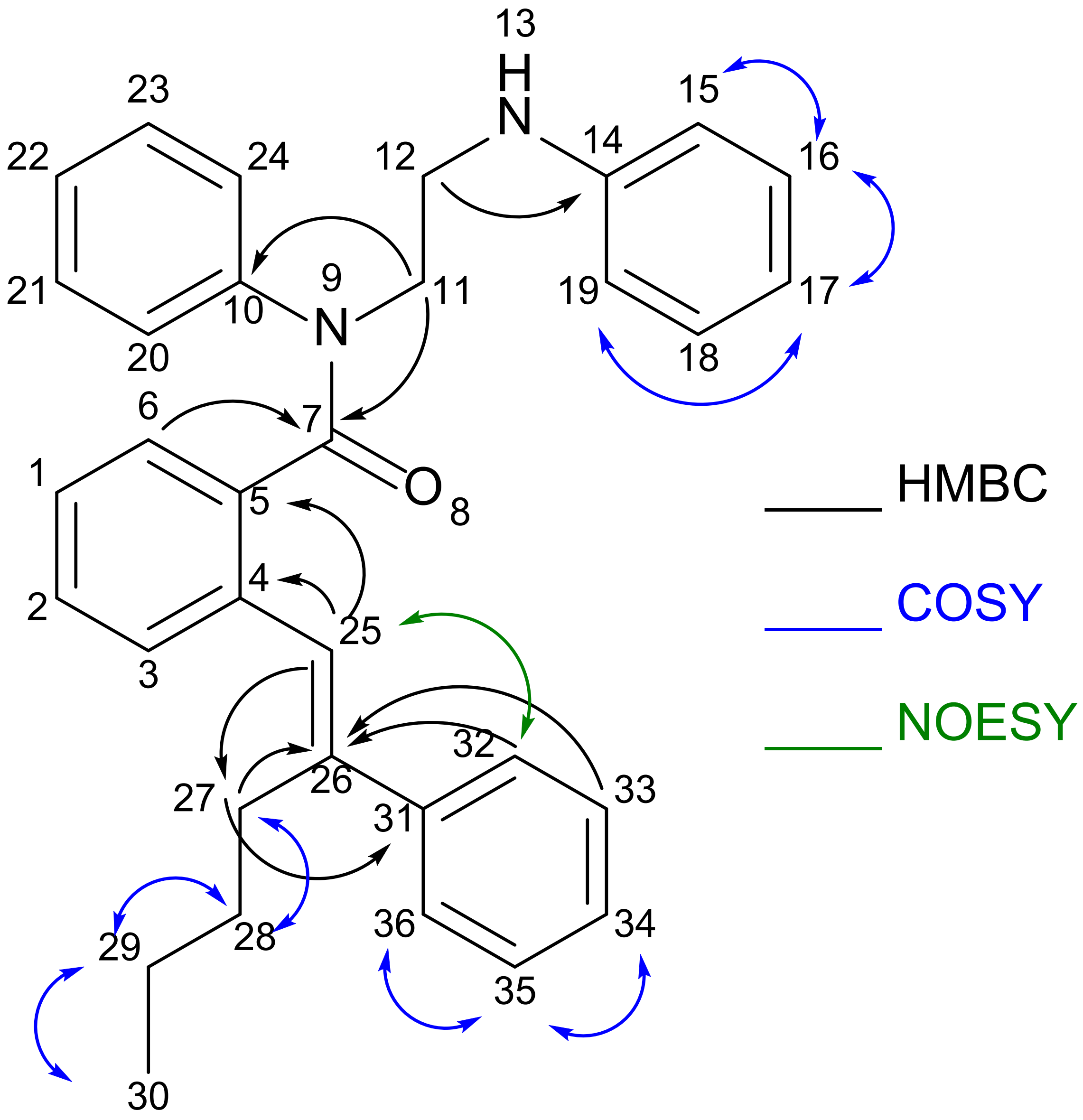

Figure S11. Atom connectivity map of **4c** derived from 2D NMR data. Note that only selective correlations are shown to improve clarity. Similar correlations are also observed for **4o**.

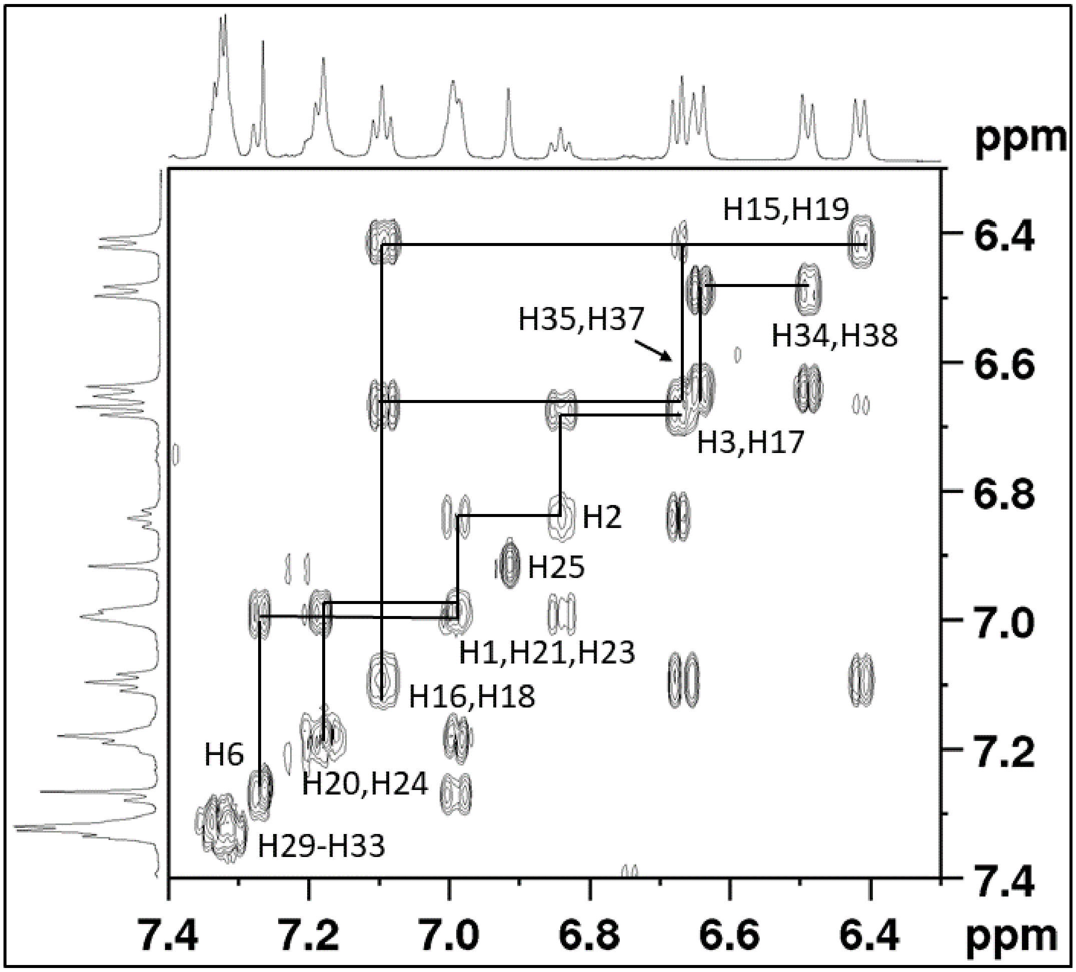

Figure S12. ^1H-^1H COSY cross-section of aromatic protons for **4o**. The resonance assignments are marked accordingly. The lack of COSY 1H-1H coupling correlations with any other protons, an integrated intensity of approximately 5.0 and distinctive HMBC correlations support the resonance assignment H29-H33.

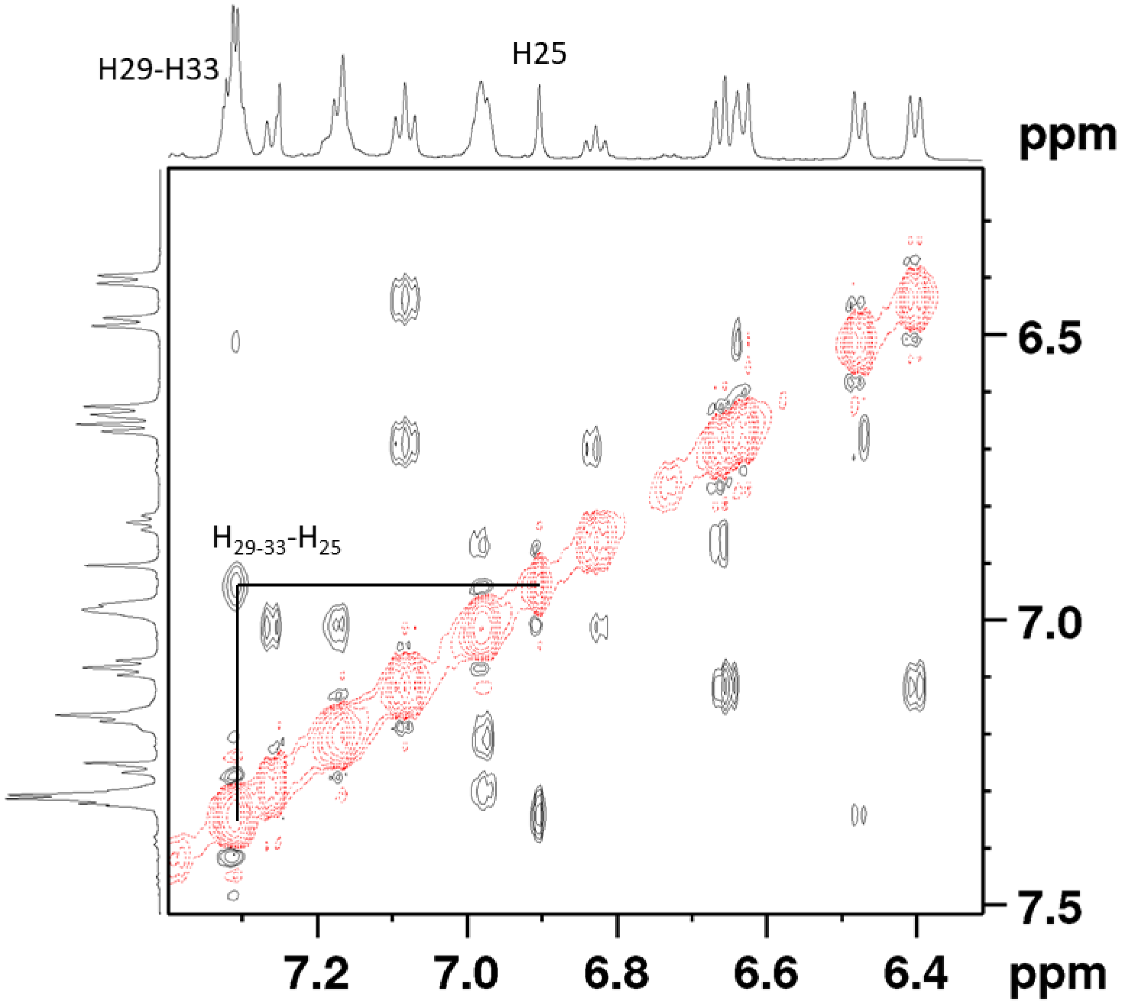

Figure S13. ^{1}H-^{1}H NOESY cross-section for **4o**. The NOE between the olefinic proton (H25) and the aromatic H_{29}-H_{33} is marked. Note that all five aromatic ring protons (H_{29}-H_{33}) resonate as an overlapping multiplet. NOE mixing time=2.0s.

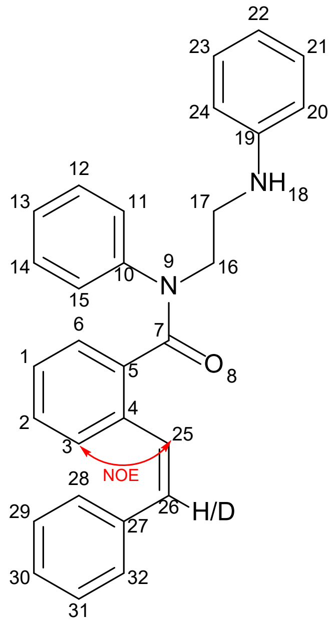

Figure S14. Structures of **4s** and **4s-*d*** with numbering.

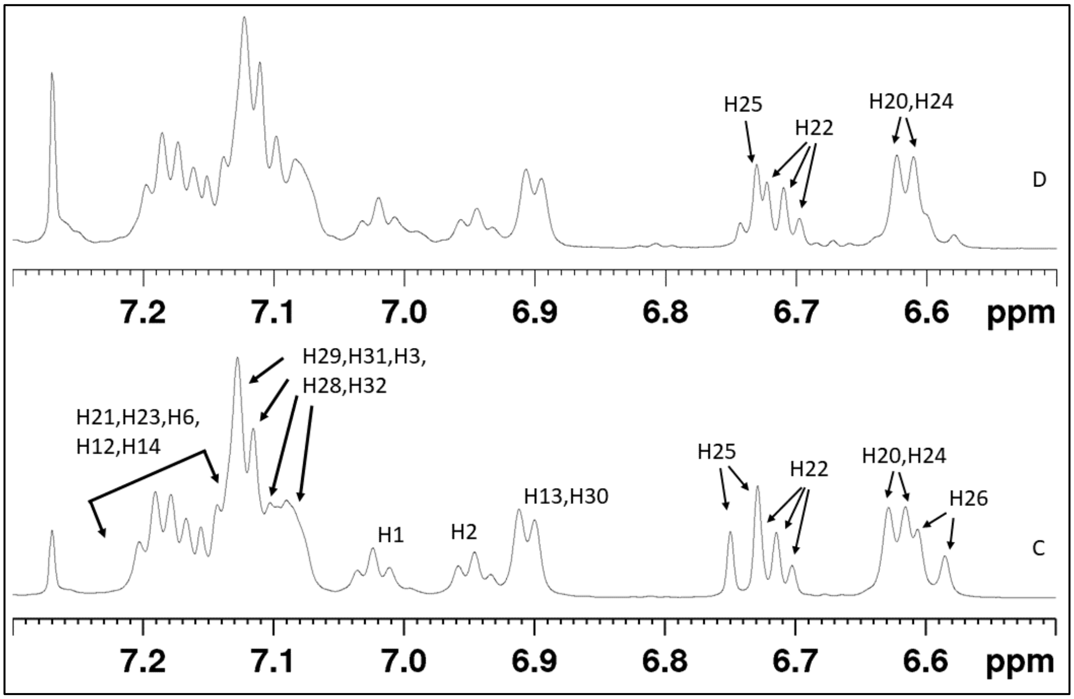

Figure S15. ^{1}H NMR sections of aromatic protons for C) **4s**, and D) **4s-*d***. The resonance assignments were performed by using ^{1}H, ^{13}C, ^{13}C-DEPT, ^{1}H-^{1}H COSY, ^{1}H-^{13}C HSQC and ^{1}H-^{13}C HMBC data, the proton atom positions are marked accordingly. The resonance assignments are tentative due to severe resonance overlap in the ^{1}H chemical shifts of aromatic protons and carbons (^{1}H, 6.85 - 7.25 δ; ^{13}C, 126 -131 δ).

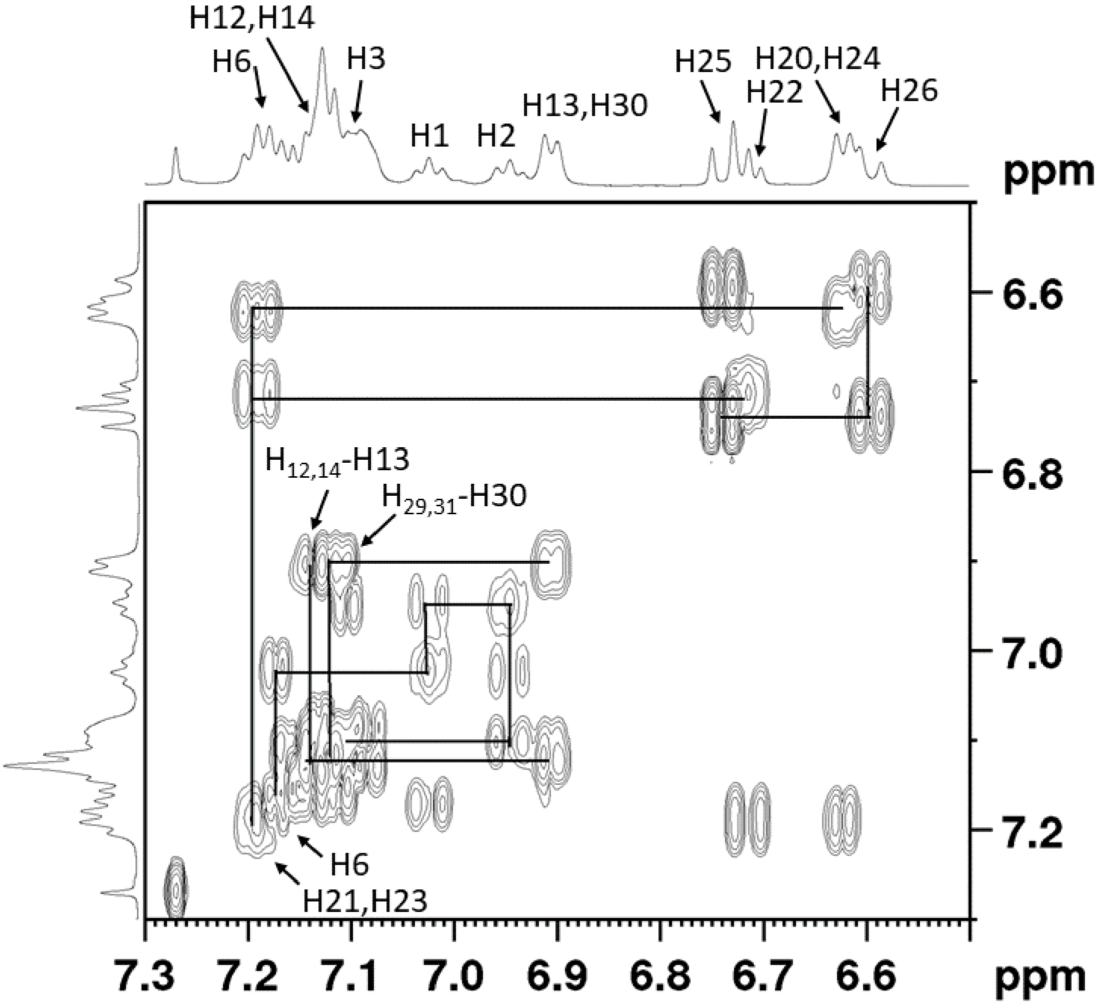

Figure S16. ${}^{1}H$-${}^{1}H$ COSY cross-section of aromatic protons for **4s**. The resonance assignments are marked accordingly. The resonance assignments are tentative due to severe resonance overlap in the ${}^{1}H$ chemical shifts of aromatic protons and carbons (${}^{1}H$, 6.85 - 7.25 δ; ${}^{13}C$, 126 - 131 δ).

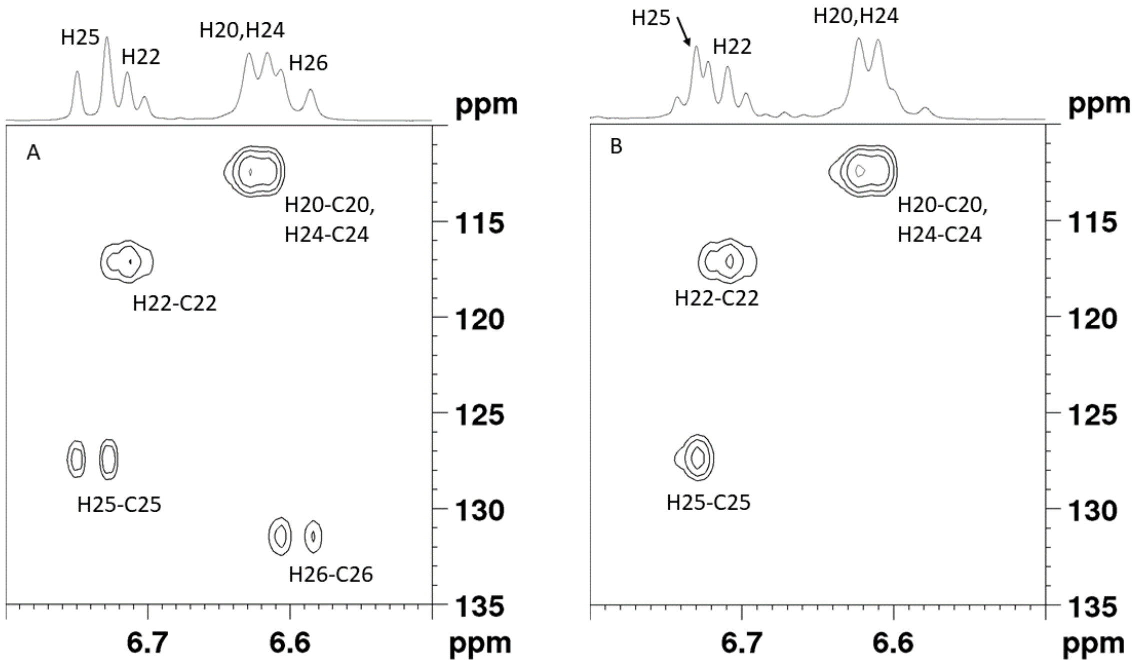

Figure S17. ^{1}H-^{13}C HSQC cross-sections of olefinic protons for A) **4s** and B) **4s-*d***. The resonance assignments are marked accordingly. Note that two cross peaks are observed for **4s**, whereas one for **4s-*d***. Also, H25 and H26 shows a fine structure (absent for **4s-*d***) of double cross peaks for **4s** as a result of ^{1}H-^{1}H scalar coupling to each other.

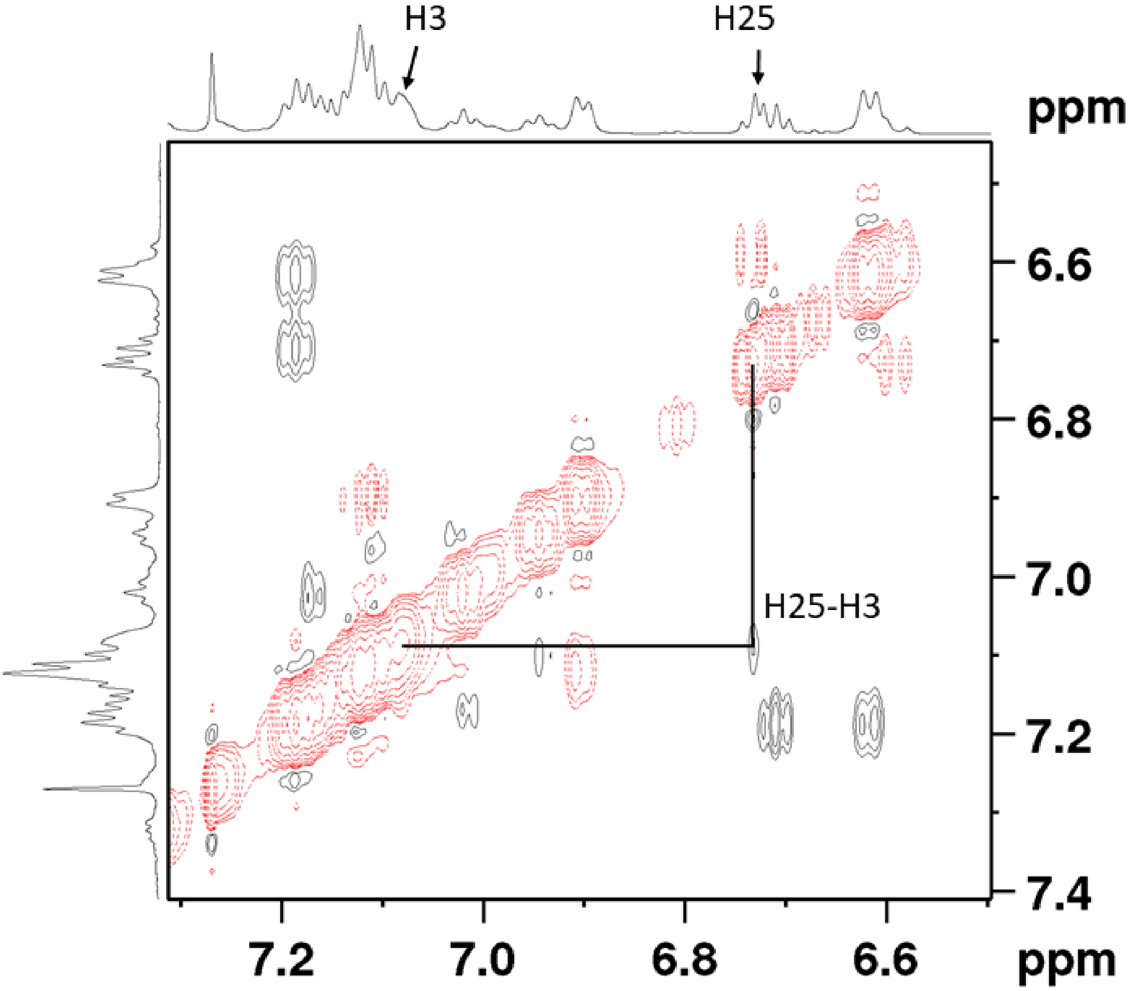

Figure S18. ^{1}H-^{1}H NOESY cross-section of aromatic protons for **4s-*d***. The NOE between H25 and H3 is marked. Note that the symmetric H3-H25 NOE is missing due to a longer spin lattice relaxation time of H25 (T_1^{H3} ~ 1.5s, T_1^{H25} ~3.7s); the dipolar pathway of relaxation from H26 is absent as a result of deuteration. NOE mixing time = 2.0 s.

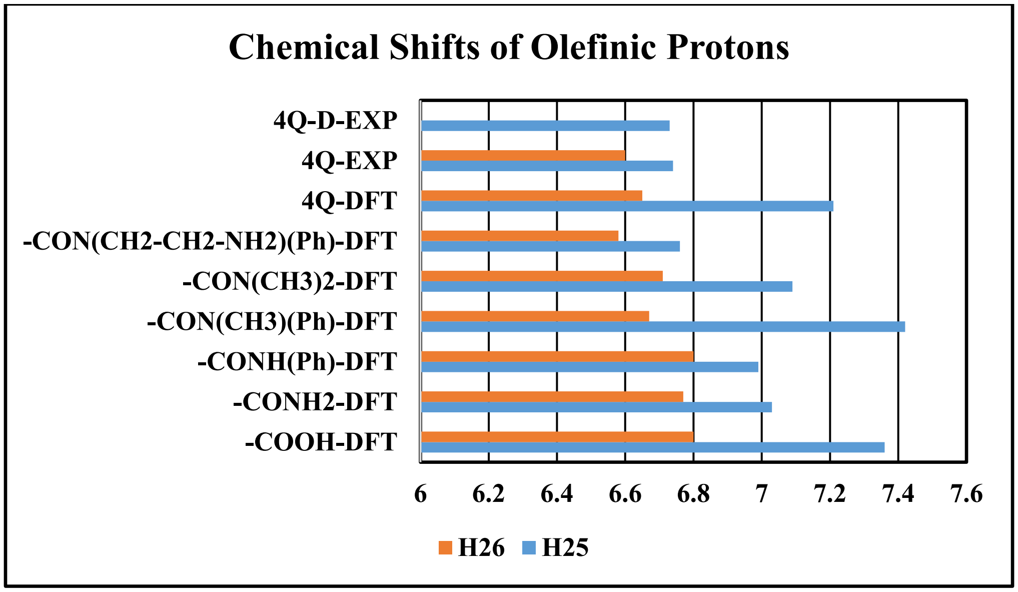

Figure S19. [1]H chemical shifts comparison of H25 and H26 of **4s** and their analogs including **4s-*d***. [1]H chemical shifts (H25, H26) of **4s** and its structural analogs were theoretically determined by performing calculations with the wB97X-V/6-311+G (2df,2p)[6-311G*] density functional model* (using the equilibrium geometries obtained from the wB97X-D/6-31G* model) in Spartan '20 (v.1.0.0) and compared with the experimental values to further confirm the position of deuteration in **4s-*d*** (C25 *Vs* C26) and the validity of their resonance assignments. The structural analogs were selected with incremental complexity using –H, -CH_3 and fragmental substituents. The computed chemical shifts of the olefinic protons fall in two groups one representing H25 (downfield, $\delta = 7.04 \pm 0.26$) and another representing H26 (upfield, $\delta = 6.70 \pm 0.09$) of **4s**. The singlet resonance observed for **4s-*d*** at ~6.73 ppm matches with the center of the doublet (6.74 ppm) of **4s** confirming that C26 is deuterated. The relative trend of chemical shifts between H25 and H26 is also preserved in all the analogs studied by chemical shift calculations supporting the conclusion about the position of deuteration in **4s-*d*** and resonance assignment of H25 and H26 in **4s** and H26 in **4s-*d***.

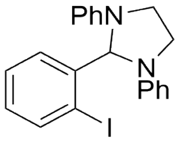

3a

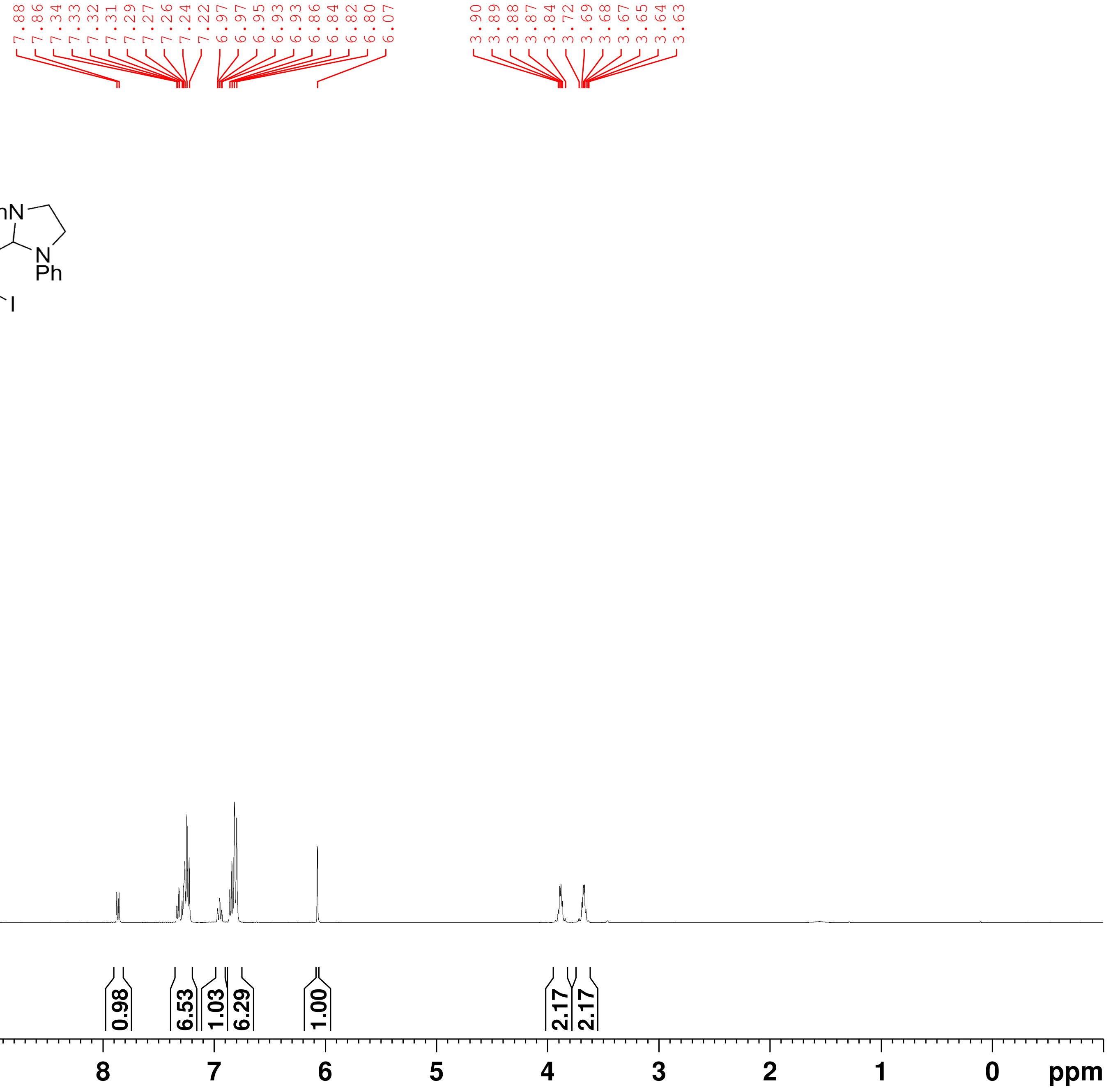

Current Data Parameters

NAME	1H_ST-3-103-reee
EXPNO	1
PROCNO	1

F2 - Acquisition Parameters

Date_	20190826
Time	11.18
INSTRUM	spect
PROBHD	5 mm Multinucl
PULPROG	zg30
TD	32768
SOLVENT	CDCl3
NS	8
DS	0
SWH	8012.820 Hz
FIDRES	0.244532 Hz
AQ	2.0447233 sec
RG	256
DW	62.400 usec
DE	6.50 usec
TE	298.2 K
D1	0.01000000 sec
TD0	1

======== CHANNEL f1 ========

NUC1	1H
P1	7.20 usec
PL1	-5.00 dB
SFO1	400.1332010 MHz

F2 - Processing parameters

SI	131072
SF	400.1300037 MHz
WDW	EM
SSB	0
LB	0.25 Hz
GB	0
PC	0.20

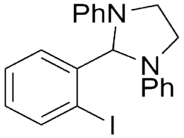

3a

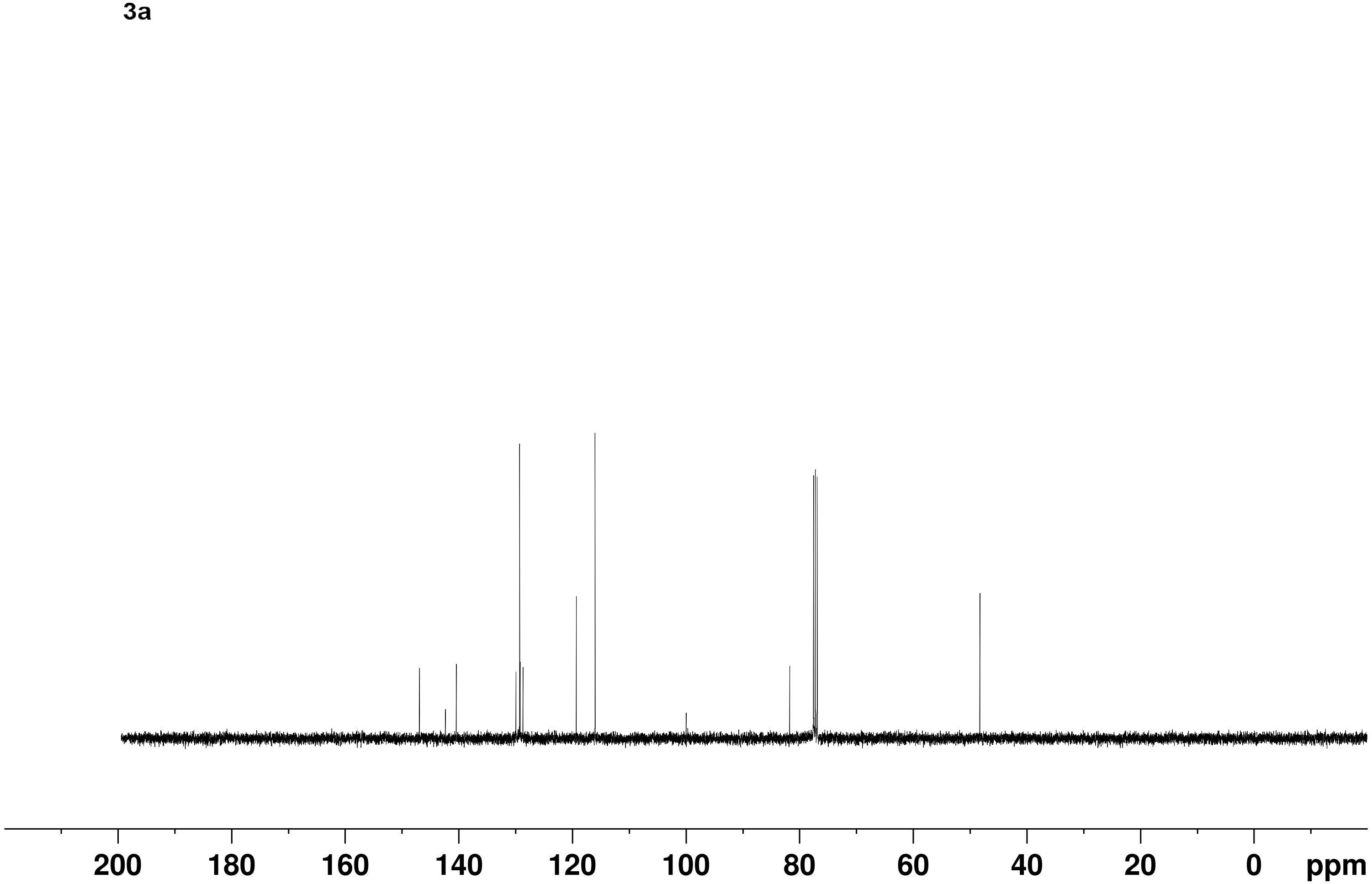


```
Current Data Parameters
NAME      13C_ST-3-103-reee
EXPNO                     2
PROCNO                    1

F2 - Acquisition Parameters
Date_              20190826
Time                  11.26
INSTRUM               spect
PROBHD     5 mm Multinucl
PULPROG             zgdc30
TD                   65536
SOLVENT              CDCl3
NS                     150
DS                       4
SWH             26246.719 Hz
FIDRES           0.400493 Hz
AQ              1.2484608 sec
RG                     181
DW                  19.050 usec
DE                    6.50 usec
TE                   298.2 K
D1              0.69999999 sec
d11             0.03000000 sec
TD0                      1

======== CHANNEL f1 ========
NUC1                   13C
P1                    8.07 usec
PL1                  -6.00 dB
SFO1           100.6196894 MHz

======== CHANNEL f2 ========
CPDPRG[2           waltz16
NUC2                    1H
PCPD2                80.00 usec
PL2        0 dB
PL12                 18.00 dB
SFO2           400.1318006 MHz

F2 - Processing parameters
SI                  131072
SF             100.6127564 MHz
WDW                     EM
SSB        0
LB                    0.80 Hz
GB         0
PC                    0.50
```

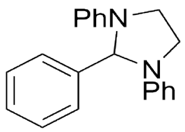

3b

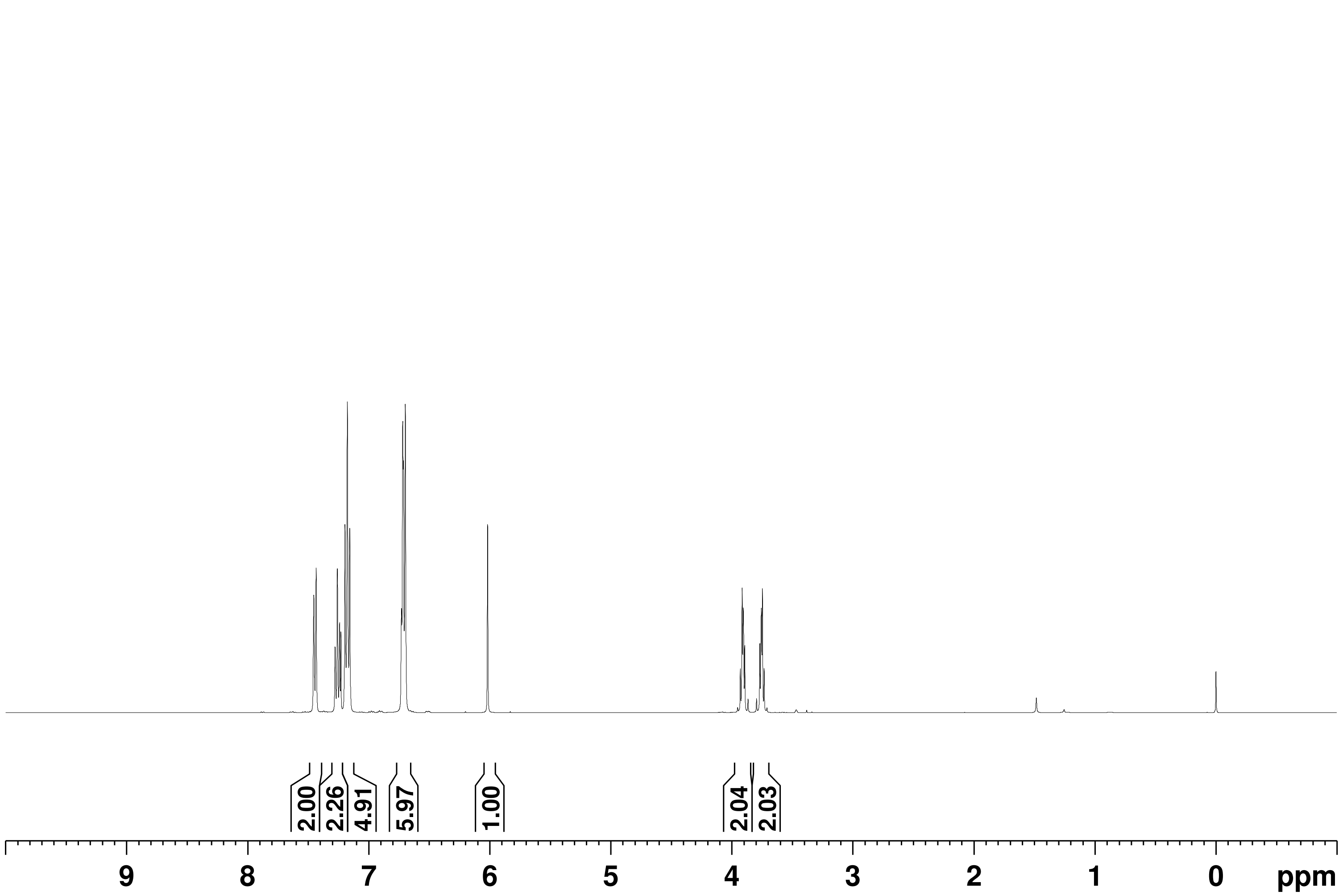

BRUKER

```
Current Data Parameters
NAME       1H_ST-6-281-re
EXPNO                   2
PROCNO                  1

F2 - Acquisition Parameters
Date_            20220103
Time                18.04 h
INSTRUM            Avance
PROBHD    Z167430_0032 (
PULPROG              zg30
TD                  65536
SOLVENT             CDCl3
NS                     16
DS                      2
SWH              8196.722 Hz
FIDRES           0.250144 Hz
AQ              3.9976959 sec
RG                    101
DW                 61.000 usec
DE                  13.20 usec
TE                  298.0 K
D1             1.00000000 sec
TD0                     1
SFO1          400.3024719 MHz
NUC1                   1H
P0                   4.00 usec
P1                  12.00 usec
PLW1           8.80000019 W

F2 - Processing parameters
SI                  65536
SF            400.3000223 MHz
WDW                    EM
SSB       0
LB                   0.30 Hz
GB        0
PC                   1.00
```

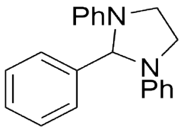

3b

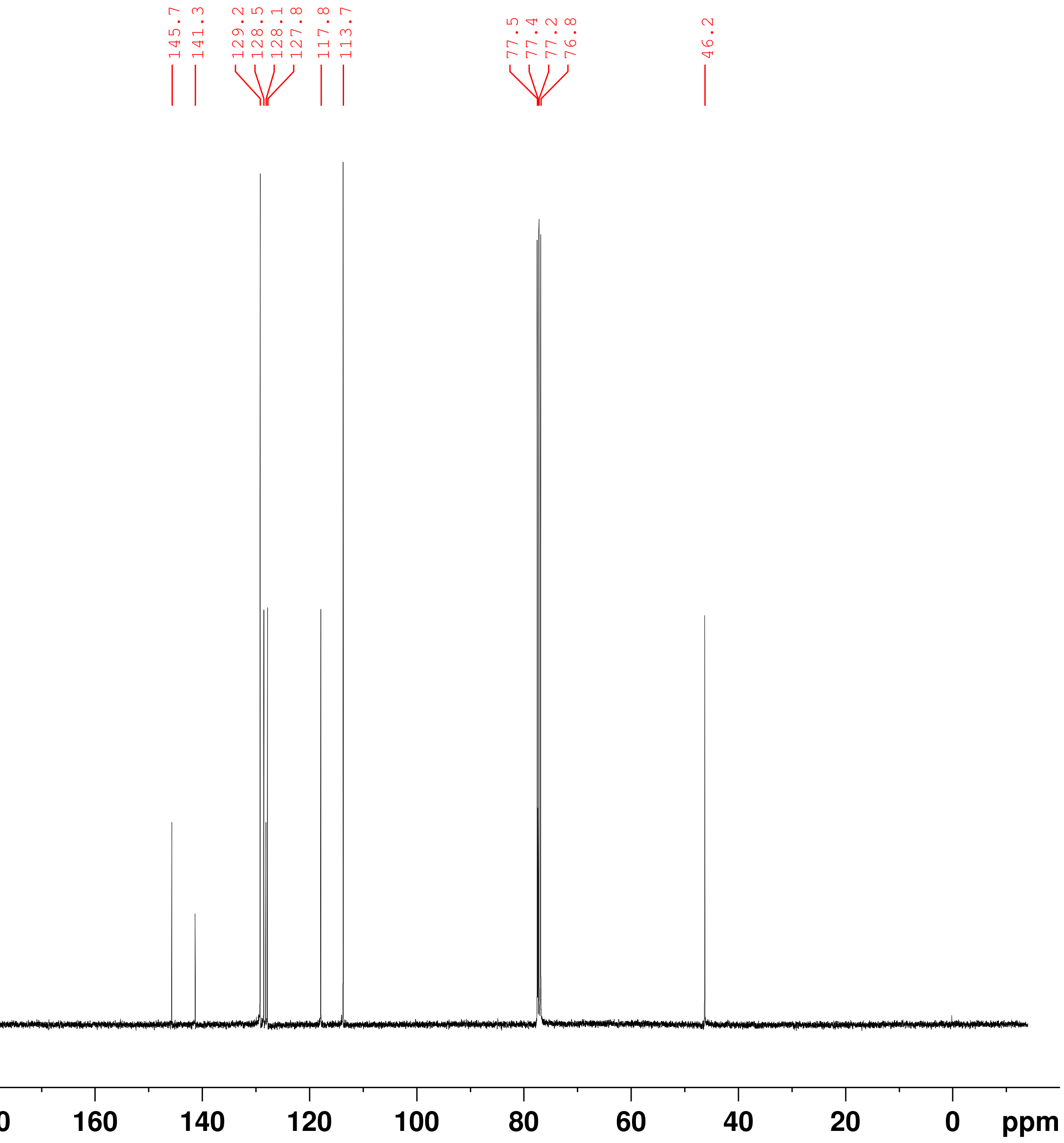


```
Current Data Parameters
NAME       13C_ST-6-281-re
EXPNO                    3
PROCNO                   1

F2 - Acquisition Parameters
Date_             20220103
Time                 18.02 h
INSTRUM             Avance
PROBHD     Z167430_0032 (
PULPROG            zgpg30
TD                   65536
SOLVENT              CDCl3
NS                     150
DS                       4
SWH              25000.000 Hz
FIDRES            0.762939 Hz
AQ               1.3107200 sec
RG                    3.25
DW                  20.000 usec
DE                   18.29 usec
TE                   298.0 K
D1              2.00000000 sec
D11             0.03000000 sec
TD0                      1
SFO1           100.6665872 MHz
NUC1                   13C
P0                    3.33 usec
P1                   10.00 usec
PLW1           39.31399918 W
SFO2           400.3016012 MHz
NUC2                    1H
CPDPRG[2           waltz64
PCPD2                80.00 usec
PLW2            8.80000019 W
PLW12           0.20176961 W
PLW13           0.10112690 W

F2 - Processing parameters
SI                  131072
SF             100.6555055 MHz
WDW                     EM
SSB       0
LB                    1.00 Hz
GB        0
PC                    1.40
```

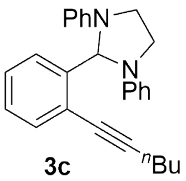

3c

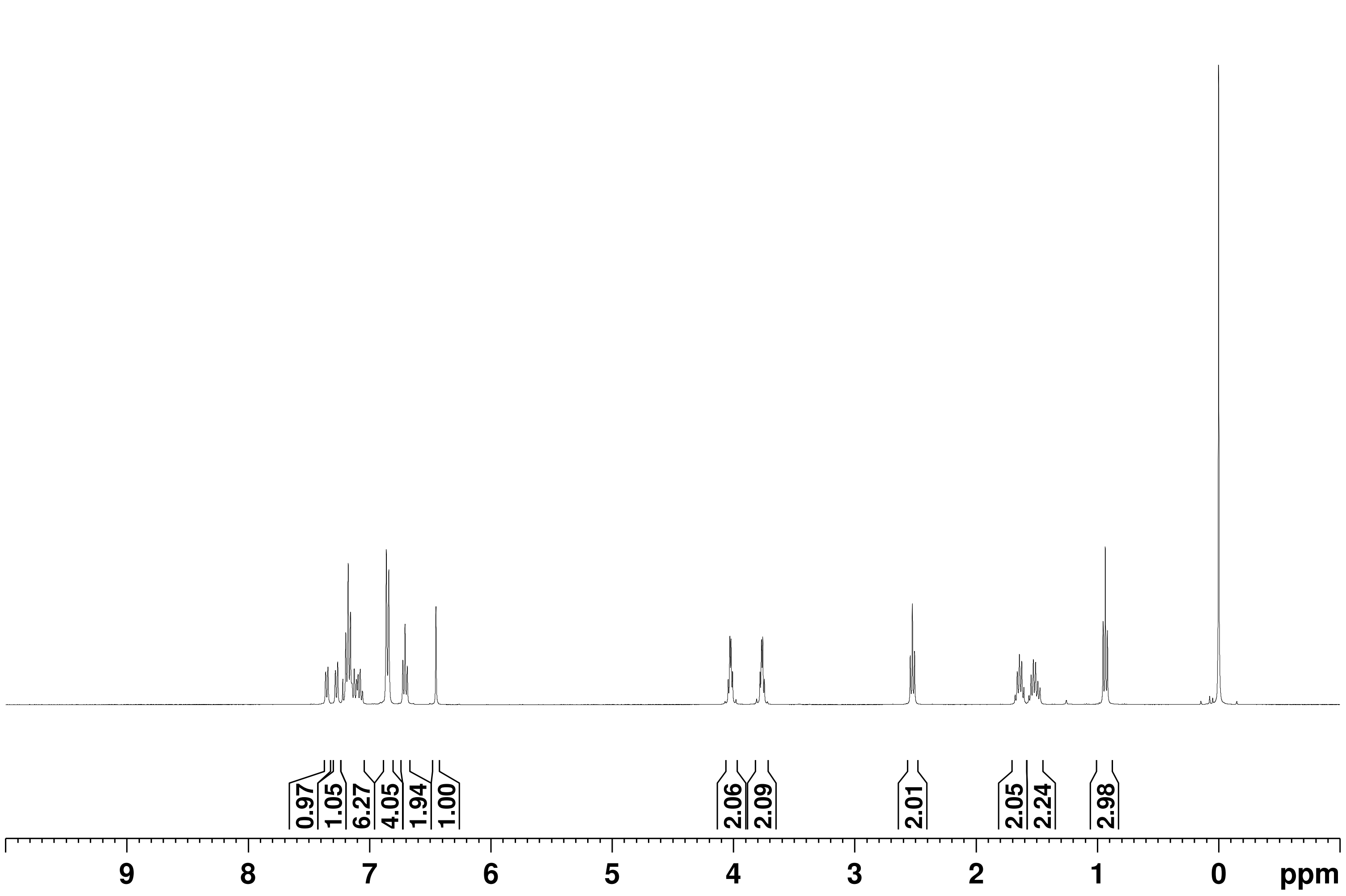


```
Current Data Parameters
NAME          1H_ST-3-195
EXPNO                   1
PROCNO                  1

F2 - Acquisition Parameters
Date_            20190925
Time                11.52
INSTRUM             spect
PROBHD   5 mm Multinucl
PULPROG              zg30
TD                  32768
SOLVENT             CDCl3
NS                     16
DS                      0
SWH              8012.820 Hz
FIDRES           0.244532 Hz
AQ              2.0447233 sec
RG                   90.5
DW                 62.400 usec
DE                   6.50 usec
TE                  296.2 K
D1             0.01000000 sec
TD0                     1

======== CHANNEL f1 ========
NUC1                   1H
P1                   7.20 usec
PL1                 -5.00 dB
SFO1          400.1332010 MHz

F2 - Processing parameters
SI                 131072
SF            400.1300260 MHz
WDW                    EM
SSB      0
LB                   0.25 Hz
GB       0
PC                   0.20
```

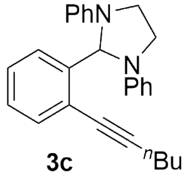

3c

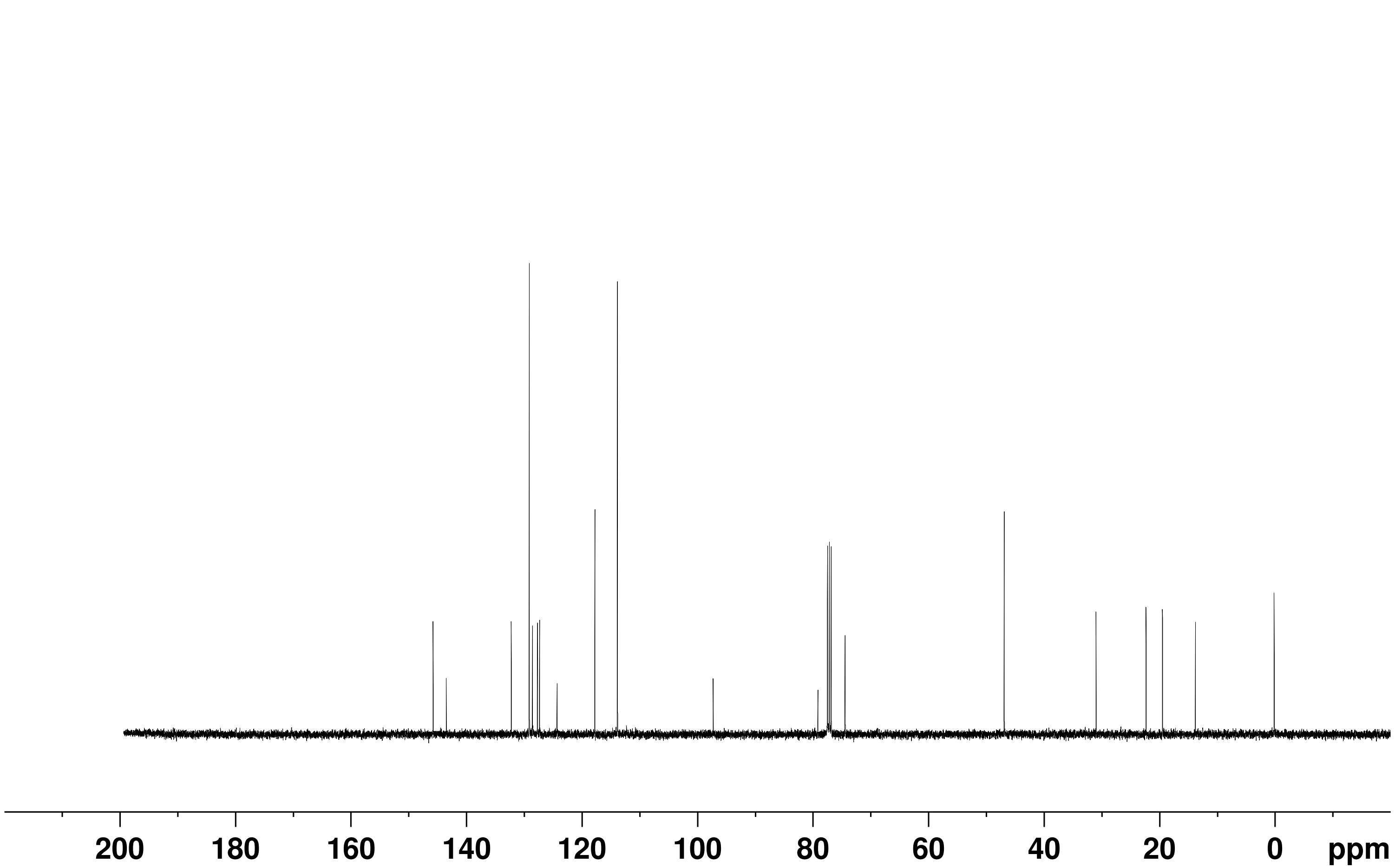

BRUKER

Current Data Parameters
NAME 13C_ST-3-195
EXPNO 1
PROCNO 1

F2 - Acquisition Parameters
Date_ 20190925
Time 12.02
INSTRUM spect
PROBHD 5 mm Multinucl
PULPROG zgdc30
TD 65536
SOLVENT CDCl3
NS 202
DS 4
SWH 26246.719 Hz
FIDRES 0.400493 Hz
AQ 1.2484608 sec
RG 512
DW 19.050 usec
DE 6.50 usec
TE 296.2 K
D1 0.69999999 sec
d11 0.03000000 sec
TD0 1

======== CHANNEL f1 ========
NUC1 13C
P1 8.07 usec
PL1 -6.00 dB
SFO1 100.6196894 MHz

======== CHANNEL f2 ========
CPDPRG[2 waltz16
NUC2 1H
PCPD2 80.00 usec
PL2 0 dB
PL12 18.00 dB
SFO2 400.1318006 MHz

F2 - Processing parameters
SI 131072
SF 100.6127598 MHz
WDW EM
SSB 0
LB 0.80 Hz
GB 0
PC 0.50

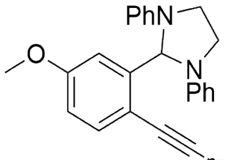

3k

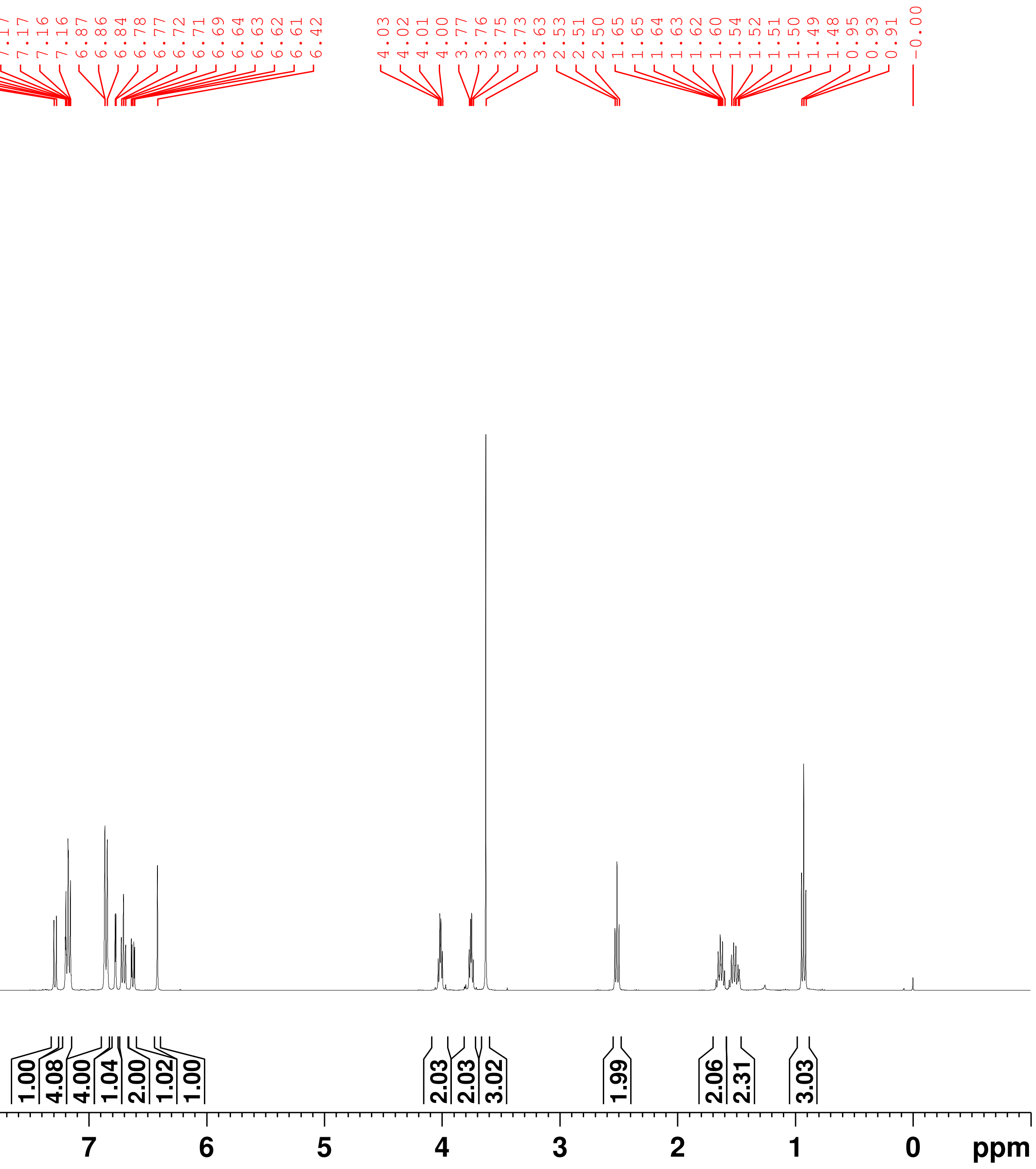

BRUKER

Current Data Parameters
NAME 1H_ST-6-91
EXPNO 1
PROCNO 1

F2 - Acquisition Parameters
Date_ 20210628
Time 14.58 h
INSTRUM Avance
PROBHD Z167430_0032 (
PULPROG zg30
TD 65536
SOLVENT CDCl3
NS 16
DS 2
SWH 8196.722 Hz
FIDRES 0.250144 Hz
AQ 3.9976959 sec
RG 57.4966
DW 61.000 usec
DE 13.20 usec
TE 298.0 K
D1 1.00000000 sec
TD0 1
SFO1 400.3024719 MHz
NUC1 1H
P0 4.00 usec
P1 12.00 usec
PLW1 8.80000019 W

F2 - Processing parameters
SI 65536
SF 400.3000318 MHz
WDW no
SSB 0
LB 0 Hz
GB 0
PC 1.00

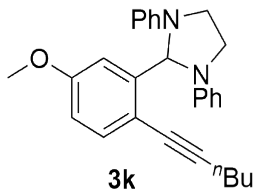

3k

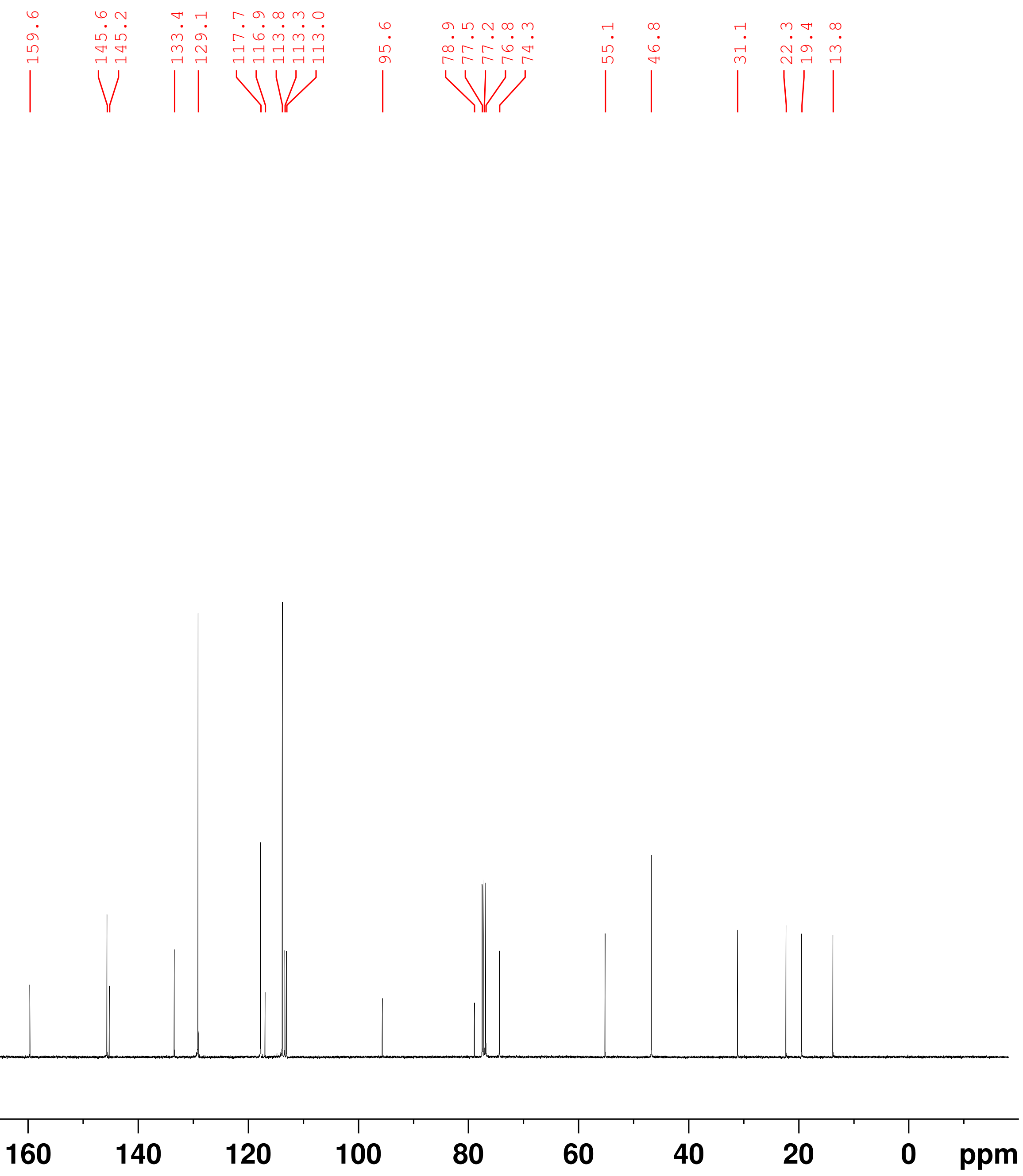

BRUKER

Current Data Parameters
NAME 13C_ST-6-91
EXPNO 1
PROCNO 1

F2 - Acquisition Parameters
Date_ 20210628
Time 15.07 h
INSTRUM Avance
PROBHD Z167430_0032 (
PULPROG zgpg30
TD 65536
SOLVENT CDCl3
NS 100
DS 4
SWH 23809.523 Hz
FIDRES 0.726609 Hz
AQ 1.3762560 sec
RG 3.25
DW 21.000 usec
DE 19.29 usec
TE 298.0 K
D1 2.00000000 sec
D11 0.03000000 sec
TD0 1
SFO1 100.6655806 MHz
NUC1 13C
P0 3.33 usec
P1 10.00 usec
PLW1 39.31399918 W
SFO2 400.3016012 MHz
NUC2 1H
CPDPRG[2 waltz64
PCPD2 80.00 usec
PLW2 8.80000019 W
PLW12 0.20176961 W
PLW13 0.10112690 W

F2 - Processing parameters
SI 131072
SF 100.6555097 MHz
WDW EM
SSB 0
LB 1.00 Hz
GB 0
PC 1.40

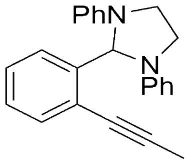

3l

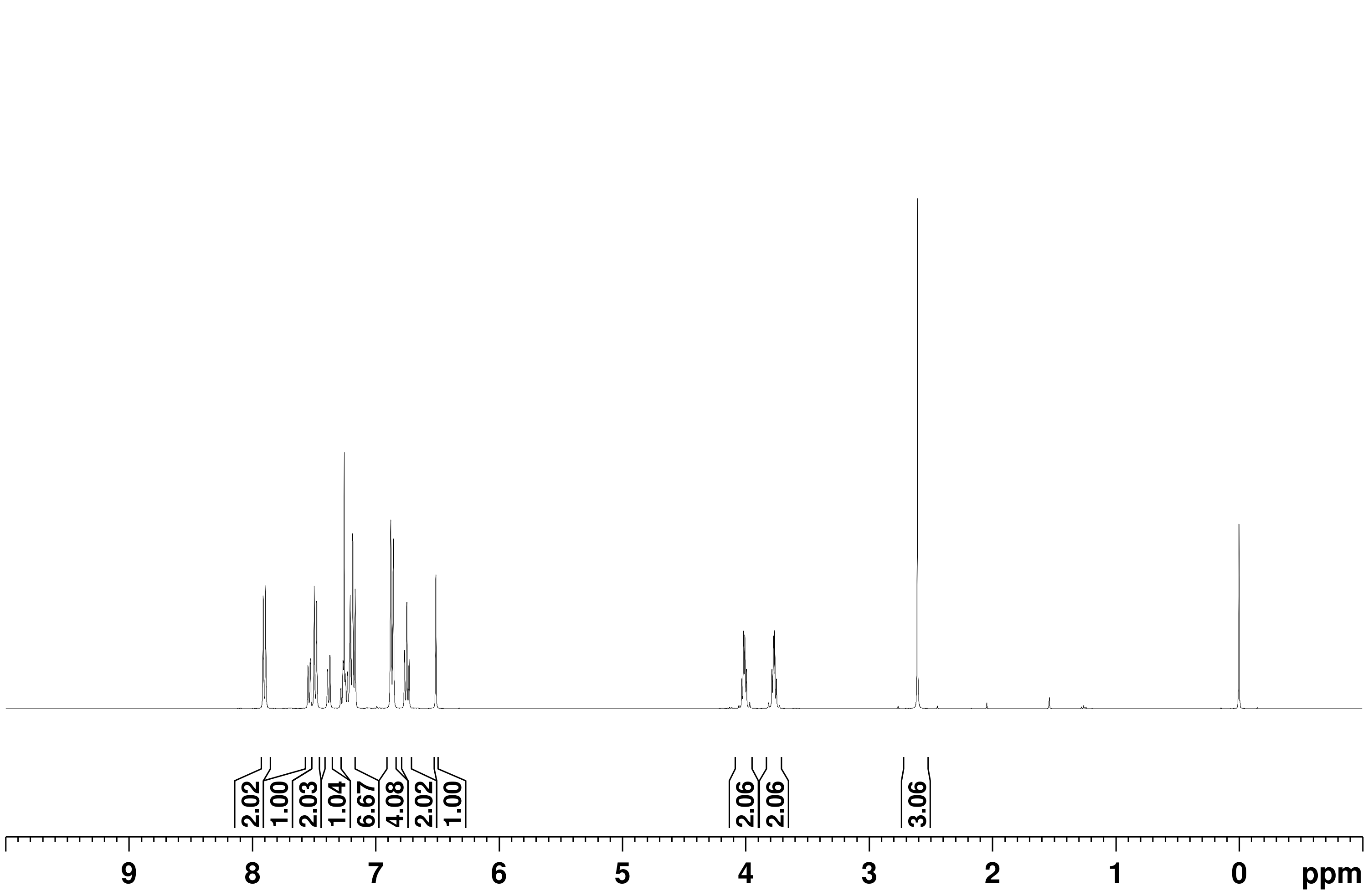


```
Current Data Parameters
NAME          1H_ST-7-163
EXPNO                   5
PROCNO                  1

F2 - Acquisition Parameters
Date_            20220425
Time                14.22 h
INSTRUM            Avance
PROBHD   Z167430_0032 (
PULPROG              zg30
TD                  65536
SOLVENT             CDCl3
NS                     16
DS                      0
SWH              8196.722 Hz
FIDRES           0.250144 Hz
AQ              3.9976959 sec
RG                    101
DW                 61.000 usec
DE                  13.20 usec
TE                  298.0 K
D1             0.10000000 sec
TD0                     1
SFO1          400.3024719 MHz
NUC1                   1H
P0                   4.00 usec
P1                  12.00 usec
PLW1           8.80000019 W

F2 - Processing parameters
SI                  65536
SF            400.3000118 MHz
WDW                    EM
SSB      0
LB                   0.30 Hz
GB       0
PC                   1.00
```

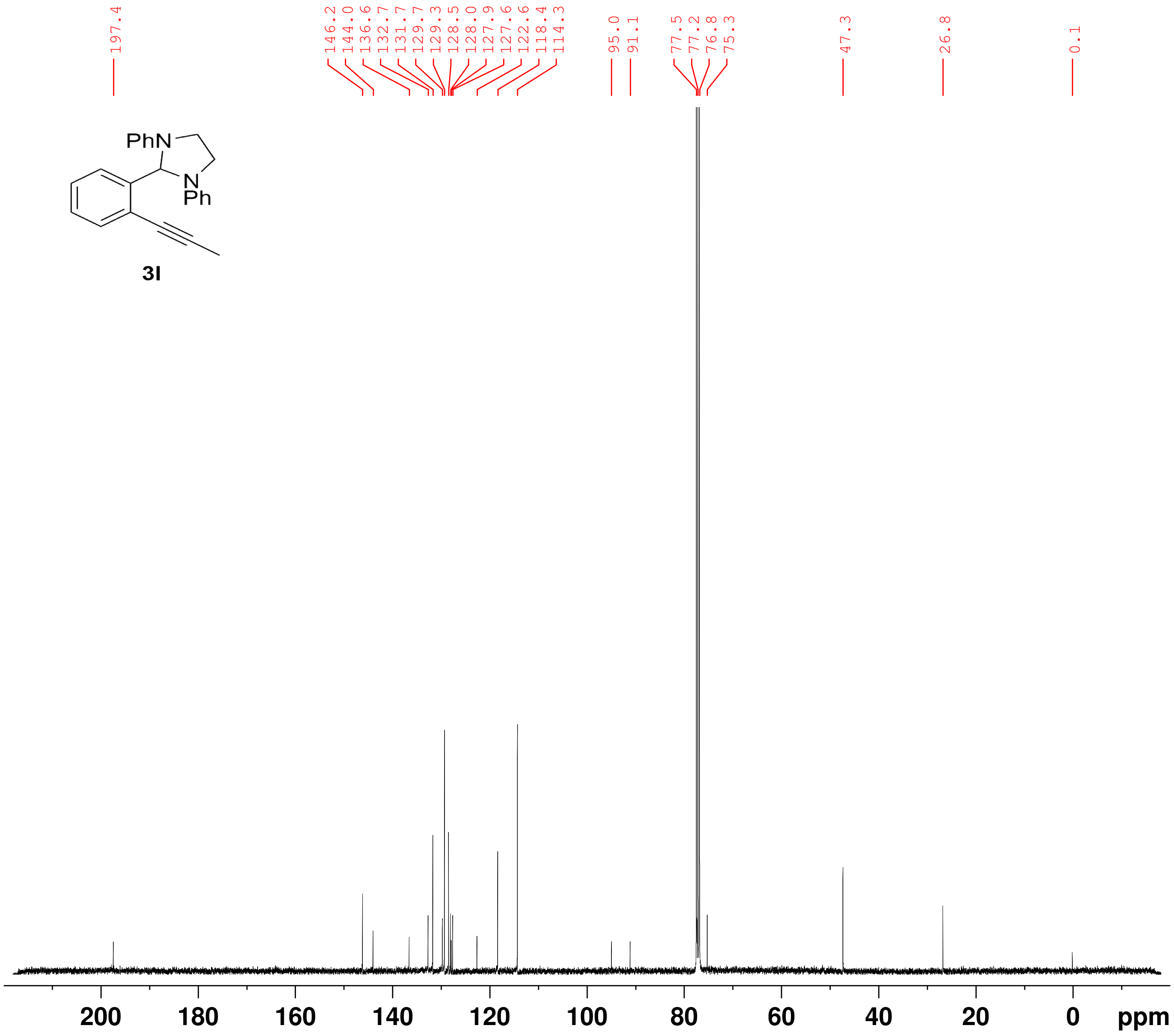

3l

BRUKER

Current Data Parameters
NAME 13C_ST-7-163
EXPNO 2
PROCNO 1

F2 - Acquisition Parameters
Date_ 20220425
Time 14.44 h
INSTRUM Avance
PROBHD Z167430_0032 (
PULPROG zgpg30
TD 65536
SOLVENT CDCl3
NS 256
DS 4
SWH 23809.523 Hz
FIDRES 0.726609 Hz
AQ 1.3762560 sec
RG 3.25
DW 21.000 usec
DE 19.29 usec
TE 298.0 K
D1 3.00000000 sec
D11 0.03000000 sec
TD0 1
SFO1 100.6655806 MHz
NUC1 13C
P0 3.33 usec
P1 10.00 usec
PLW1 39.31399918 W
SFO2 400.3016012 MHz
NUC2 1H
CPDPRG[2 waltz64
PCPD2 80.00 usec
PLW2 8.80000019 W
PLW12 0.20176961 W
PLW13 0.10112690 W

F2 - Processing parameters
SI 131072
SF 100.6555028 MHz
WDW EM
SSB 0
LB 1.00 Hz
GB 0
PC 1.40

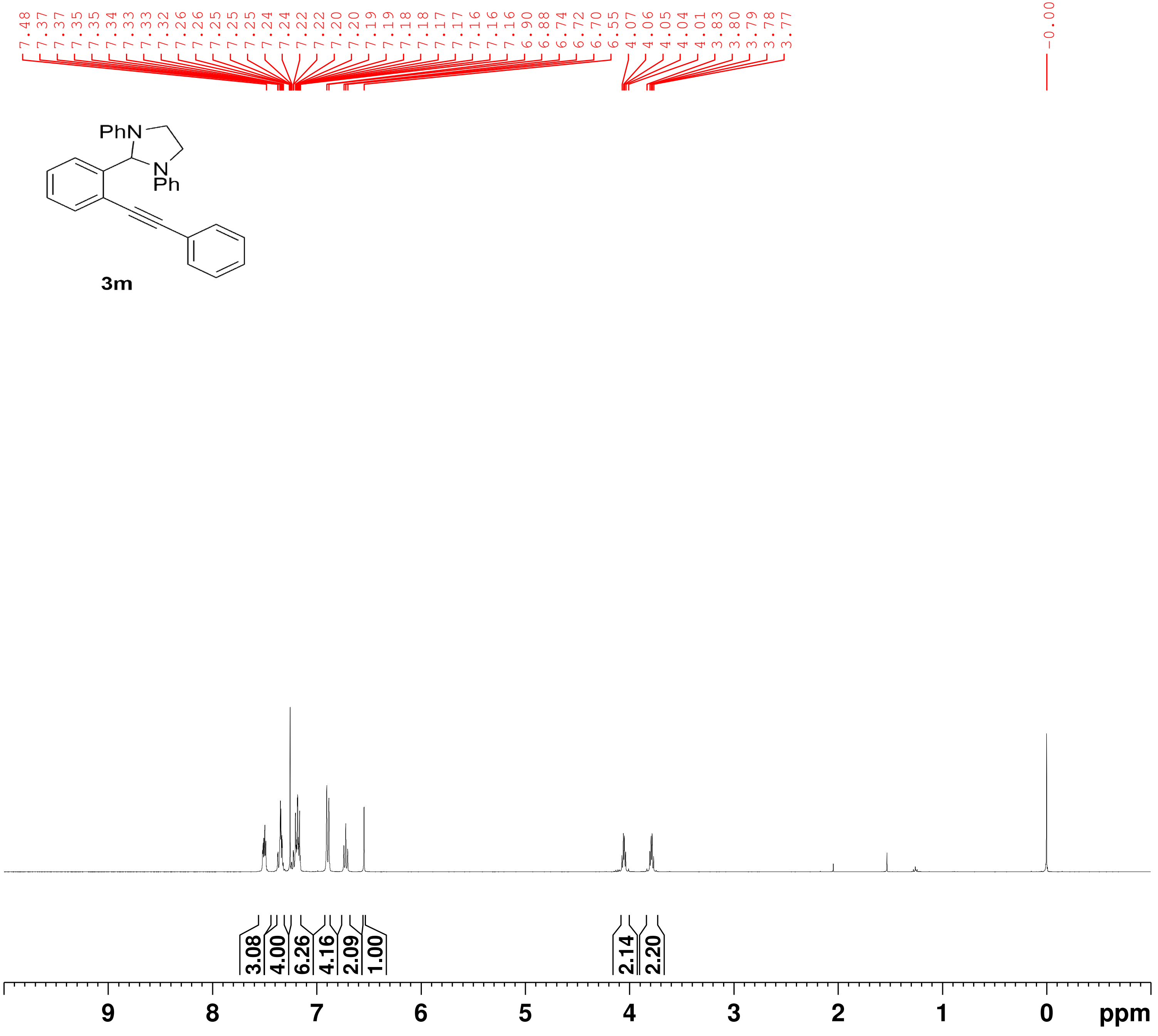


```
Current Data Parameters
NAME          1H_ST-5-187
EXPNO                   1
PROCNO                  1

F2 - Acquisition Parameters
Date_            20210303
Time                15.23
INSTRUM             spect
PROBHD   5 mm Multinucl
PULPROG              zg30
TD                  32768
SOLVENT             CDCl3
NS                     16
DS                      0
SWH              8012.820 Hz
FIDRES           0.244532 Hz
AQ              2.0447233 sec
RG                  456.1
DW                 62.400 usec
DE                   6.50 usec
TE                  295.2 K
D1             0.01000000 sec
TD0                     1

======== CHANNEL f1 ========
NUC1                   1H
P1                   7.20 usec
PL1                 -5.00 dB
SFO1          400.1332010 MHz

F2 - Processing parameters
SI                 131072
SF            400.1300113 MHz
WDW                    EM
SSB      0
LB                   0.25 Hz
GB       0
PC                   0.20
```

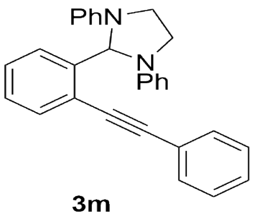

3m

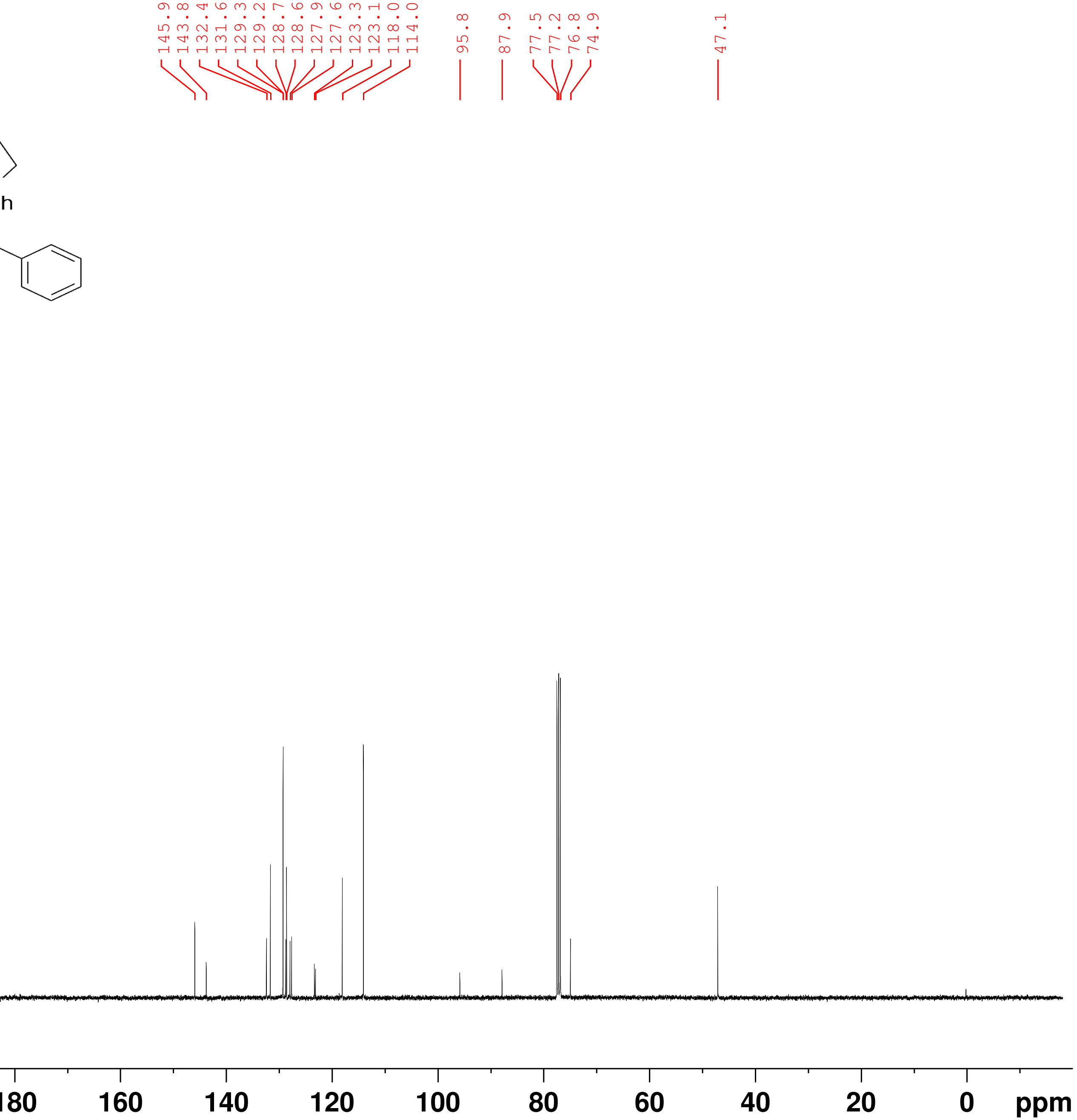

Current Data Parameters
NAME 13C_ST-5-187
EXPNO 1
PROCNO 1

F2 - Acquisition Parameters
Date_ 20210804
Time 15.01 h
INSTRUM Avance
PROBHD Z167430_0032 (
PULPROG zgpg30
TD 65536
SOLVENT CDCl3
NS 64
DS 4
SWH 23809.523 Hz
FIDRES 0.726609 Hz
AQ 1.3762560 sec
RG 3.25
DW 21.000 usec
DE 19.29 usec
TE 298.0 K
D1 2.00000000 sec
D11 0.03000000 sec
TD0 1
SFO1 100.6655806 MHz
NUC1 13C
P0 3.33 usec
P1 10.00 usec
PLW1 39.31399918 W
SFO2 400.3016012 MHz
NUC2 1H
CPDPRG[2 waltz64
PCPD2 80.00 usec
PLW2 8.80000019 W
PLW12 0.20176961 W
PLW13 0.10112690 W

F2 - Processing parameters
SI 131072
SF 100.6555036 MHz
WDW EM
SSB 0
LB 1.00 Hz
GB 0
PC 1.40

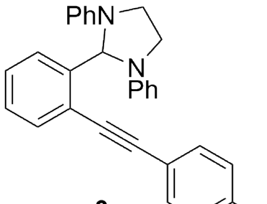

3n

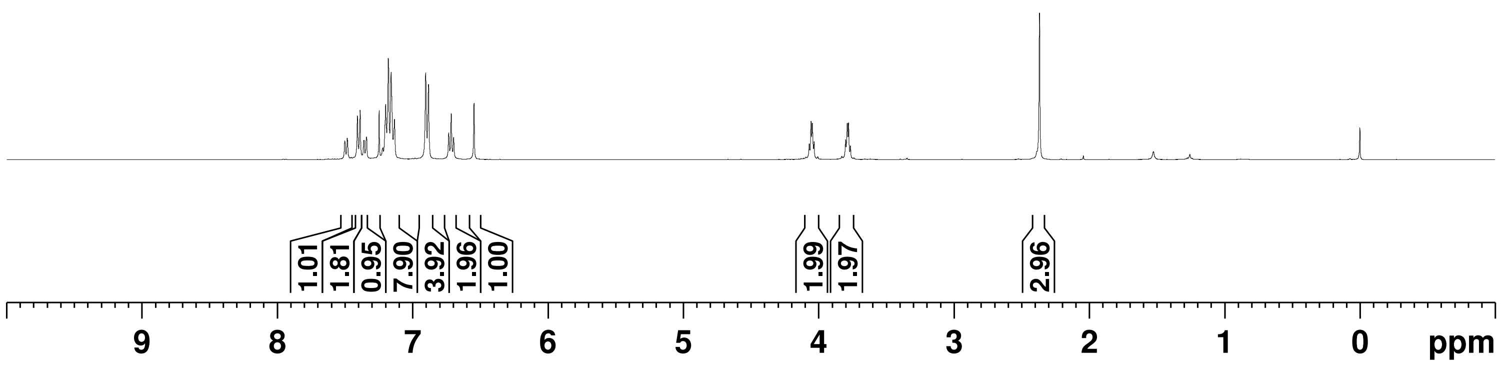

Current Data Parameters
NAME 1H_ST-5-179
EXPNO 1
PROCNO 1

F2 - Acquisition Parameters
Date_ 20210301
Time 11.42
INSTRUM spect
PROBHD 5 mm Multinucl
PULPROG zg30
TD 32768
SOLVENT CDCl3
NS 16
DS 0
SWH 8012.820 Hz
FIDRES 0.244532 Hz
AQ 2.0447233 sec
RG 287.4
DW 62.400 usec
DE 6.50 usec
TE 295.2 K
D1 0.01000000 sec
TD0 1

======== CHANNEL f1 ========
NUC1 1H
P1 7.20 usec
PL1 -5.00 dB
SFO1 400.1332010 MHz

F2 - Processing parameters
SI 131072
SF 400.1300145 MHz
WDW EM
SSB 0
LB 0.25 Hz
GB 0
PC 0.20

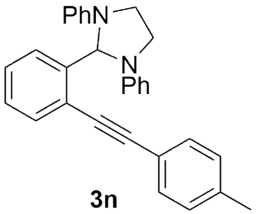

3n

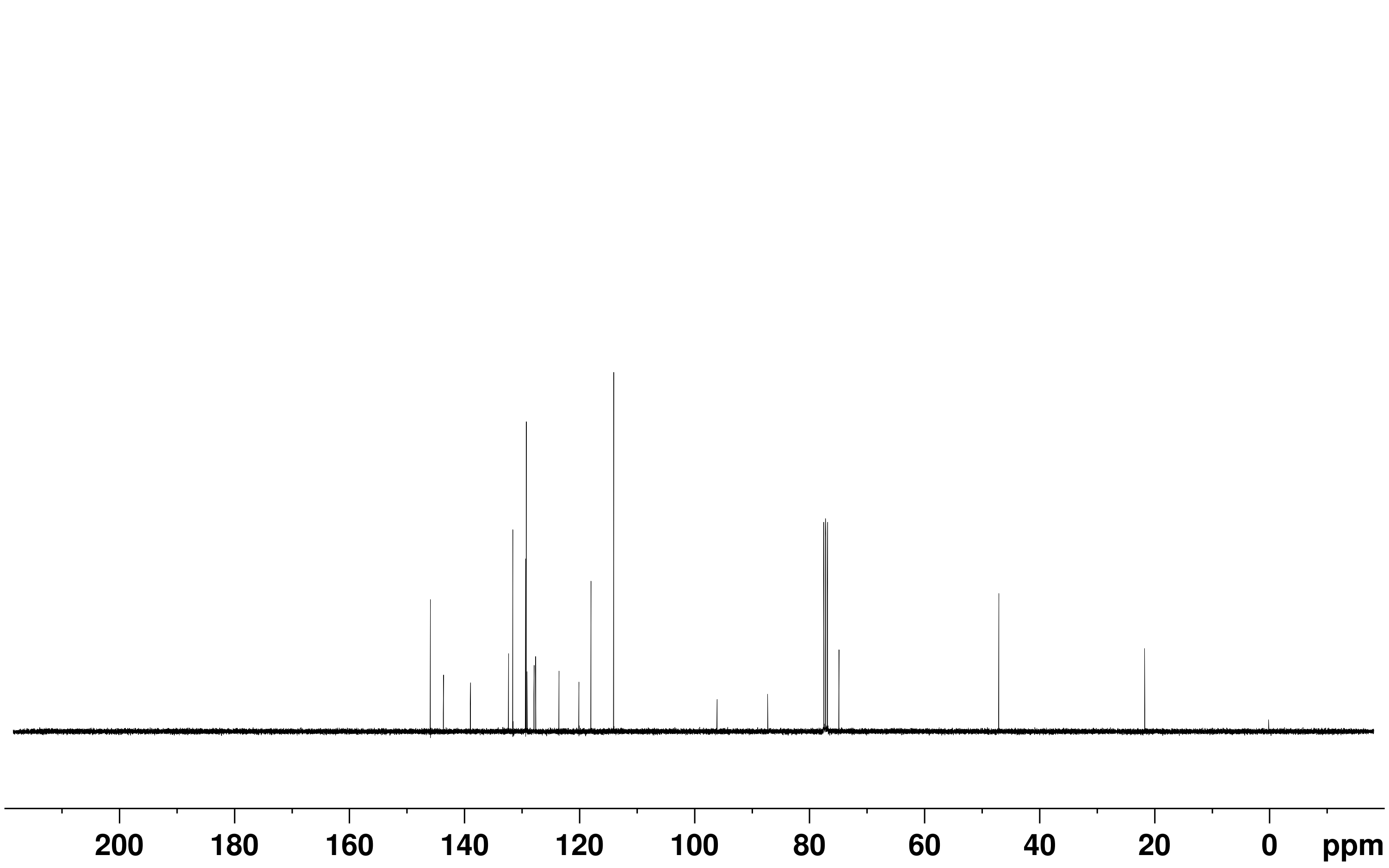

Current Data Parameters
NAME 13C_ST-5-179
EXPNO 1
PROCNO 1

F2 - Acquisition Parameters
Date_ 20210804
Time 14.40 h
INSTRUM Avance
PROBHD Z167430_0032 (
PULPROG zgpg30
TD 65536
SOLVENT CDCl3
NS 100
DS 4
SWH 23809.523 Hz
FIDRES 0.726609 Hz
AQ 1.3762560 sec
RG 3.25
DW 21.000 usec
DE 19.29 usec
TE 298.0 K
D1 2.00000000 sec
D11 0.03000000 sec
TD0 1
SFO1 100.6655806 MHz
NUC1 13C
P0 3.33 usec
P1 10.00 usec
PLW1 39.31399918 W
SFO2 400.3016012 MHz
NUC2 1H
CPDPRG[2 waltz64
PCPD2 80.00 usec
PLW2 8.80000019 W
PLW12 0.20176961 W
PLW13 0.10112690 W

F2 - Processing parameters
SI 131072
SF 100.6555043 MHz
WDW no
SSB 0
LB 0 Hz
GB 0
PC 1.40

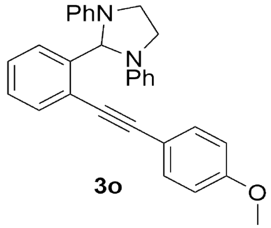

3o

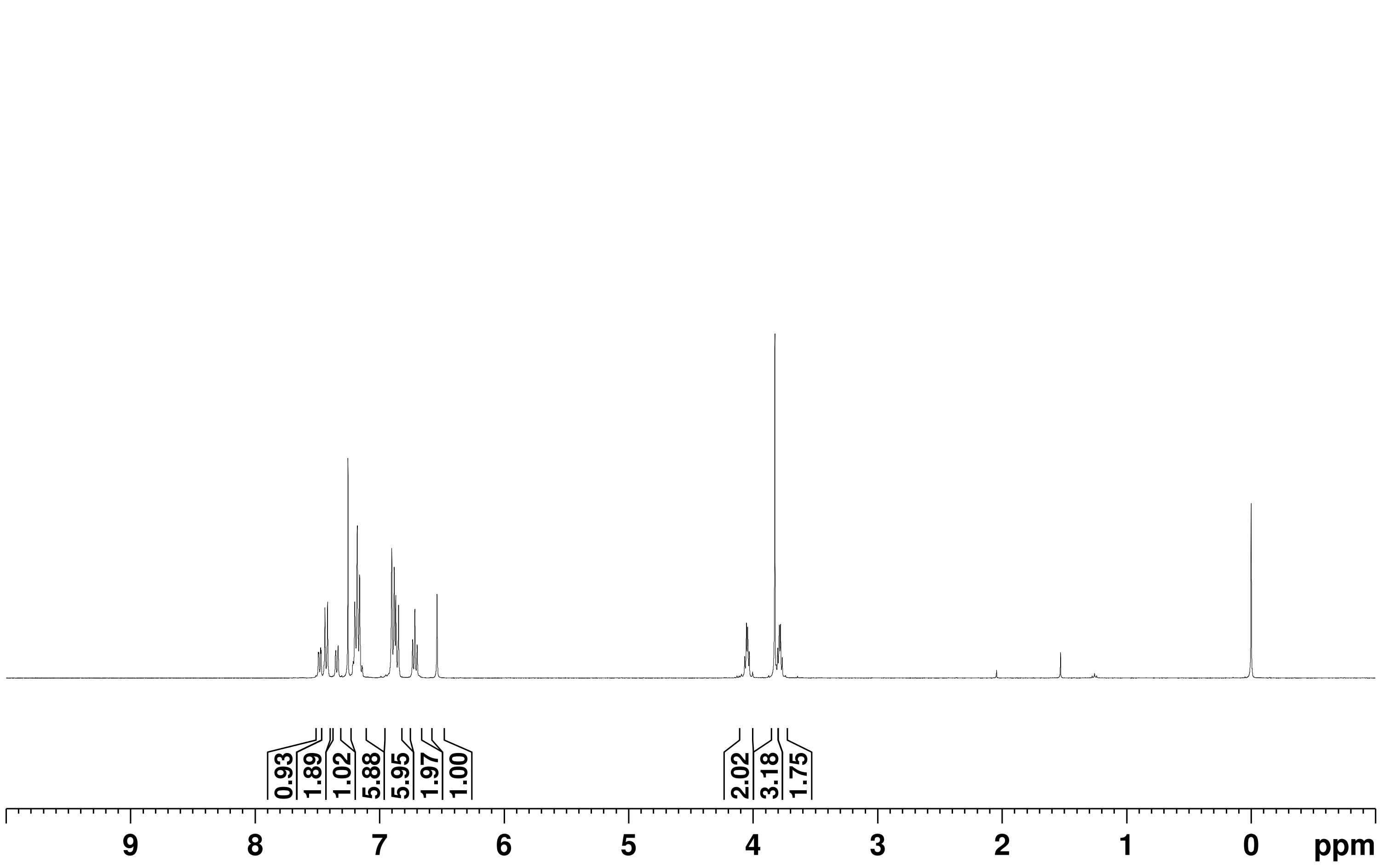


```
Current Data Parameters
NAME      1H_ST-5-181-re
EXPNO                  1
PROCNO                 1

F2 - Acquisition Parameters
Date_           20210303
Time               15.19
INSTRUM            spect
PROBHD    5 mm Multinucl
PULPROG             zg30
TD                 32768
SOLVENT            CDCl3
NS                    16
DS                     0
SWH             8012.820 Hz
FIDRES          0.244532 Hz
AQ             2.0447233 sec
RG                 456.1
DW                62.400 usec
DE                  6.50 usec
TE                 295.2 K
D1            0.01000000 sec
TD0                    1

======== CHANNEL f1 ========
NUC1                  1H
P1                  7.20 usec
PL1                -5.00 dB
SFO1         400.1332010 MHz

F2 - Processing parameters
SI                131072
SF           400.1300118 MHz
WDW                   EM
SSB      0
LB                  0.25 Hz
GB       0
PC                  0.20
```

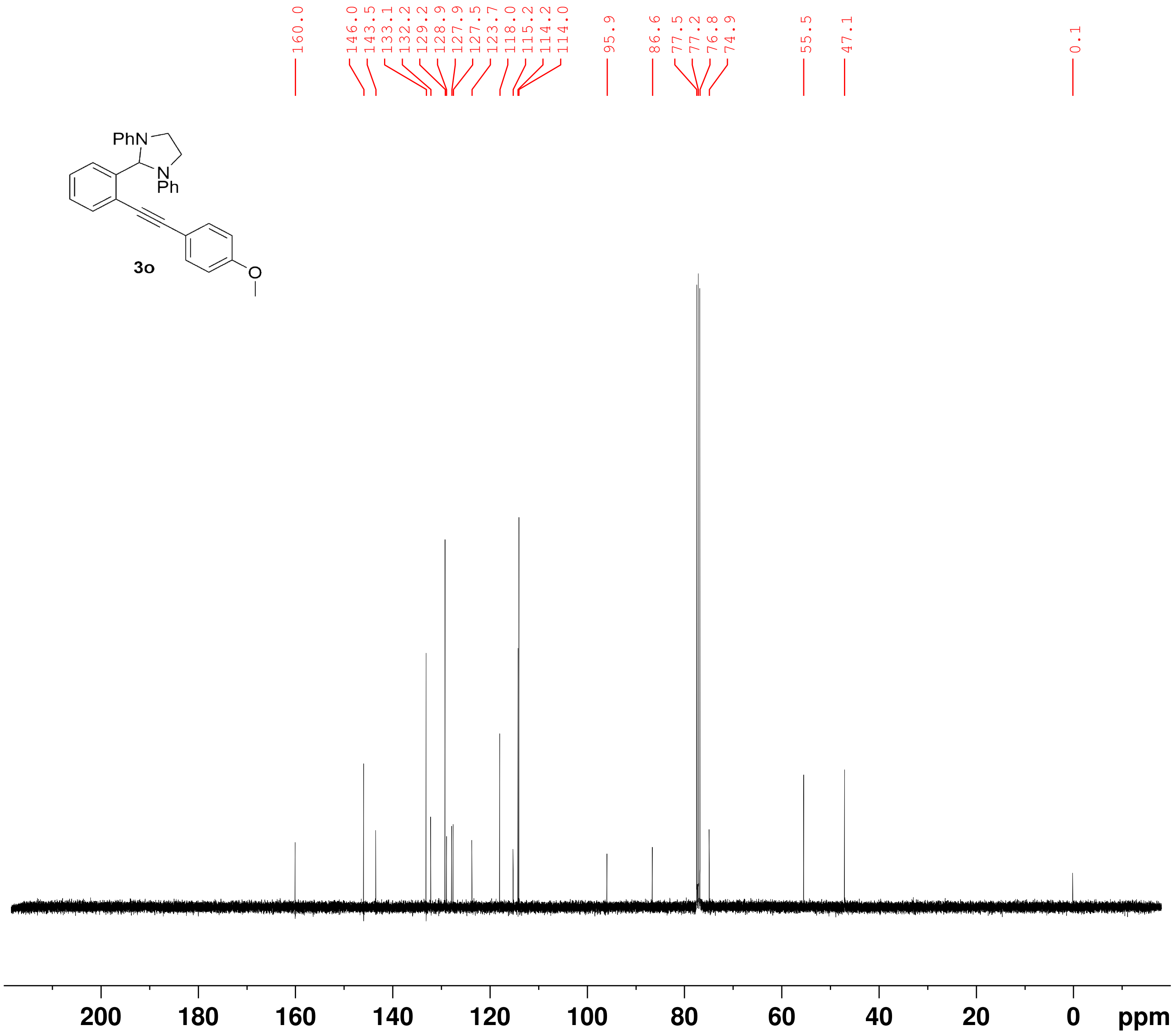


```
Current Data Parameters
NAME          13C_ST-5-181
EXPNO                    1
PROCNO                   1

F2 - Acquisition Parameters
Date_             20210804
Time                 15.14 h
INSTRUM             Avance
PROBHD    Z167430_0032 (
PULPROG            zgpg30
TD                   65536
SOLVENT              CDCl3
NS                     165
DS                       4
SWH              23809.523 Hz
FIDRES            0.726609 Hz
AQ               1.3762560 sec
RG                    3.25
DW                  21.000 usec
DE                   19.29 usec
TE                   298.0 K
D1              2.00000000 sec
D11             0.03000000 sec
TD0                      1
SFO1           100.6655806 MHz
NUC1                   13C
P0                    3.33 usec
P1                   10.00 usec
PLW1           39.31399918 W
SFO2           400.3016012 MHz
NUC2                    1H
CPDPRG[2           waltz64
PCPD2                80.00 usec
PLW2            8.80000019 W
PLW12           0.20176961 W
PLW13           0.10112690 W

F2 - Processing parameters
SI                  131072
SF             100.6555020 MHz
WDW                     no
SSB      0
LB       0 Hz
GB       0
PC                    1.40
```

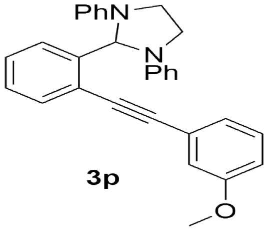

3p

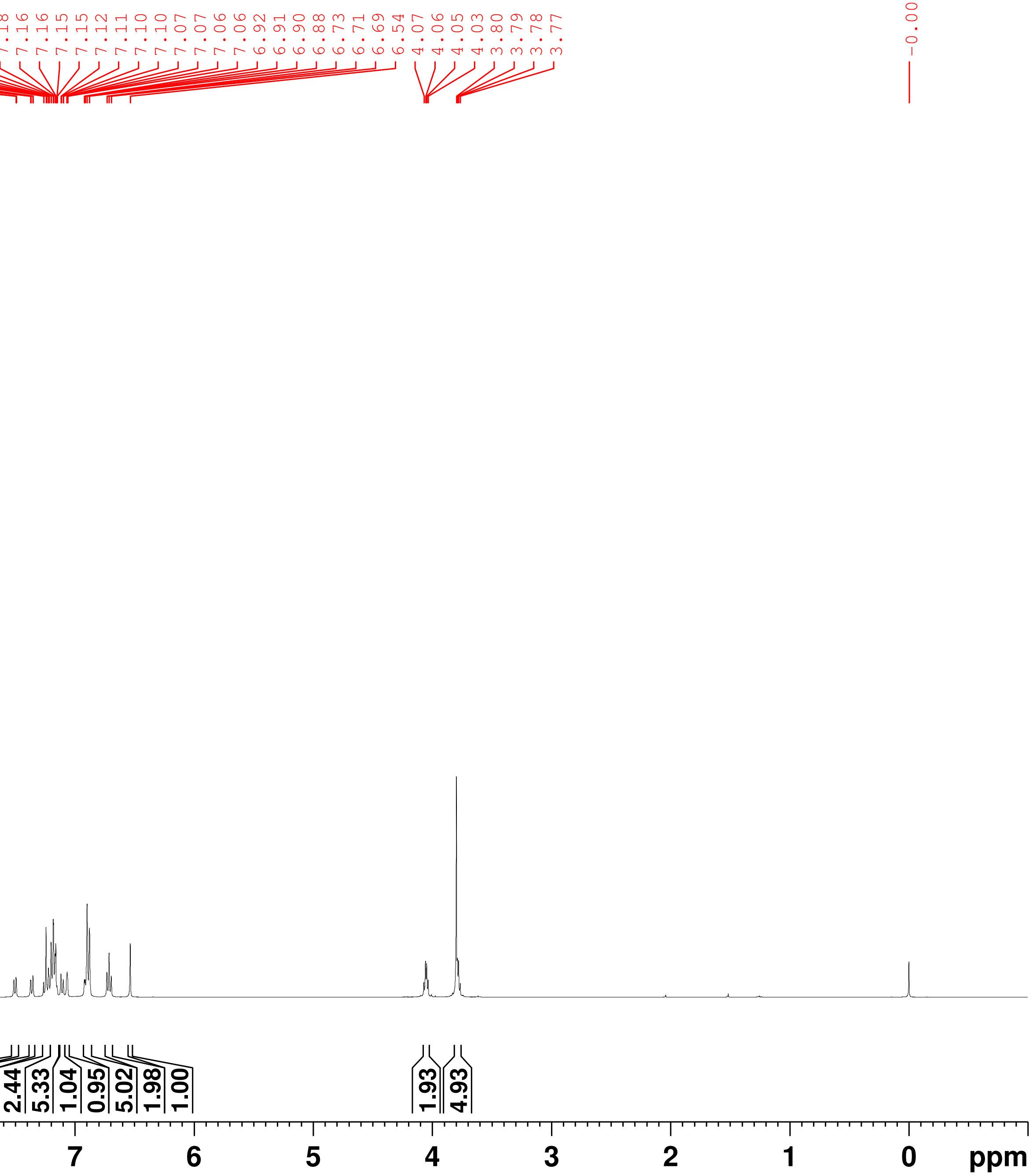


```
Current Data Parameters
NAME          1H_ST-5-189
EXPNO                   1
PROCNO                  1

F2 - Acquisition Parameters
Date_            20210303
Time                14.44
INSTRUM             spect
PROBHD    5 mm Multinucl
PULPROG              zg30
TD                  32768
SOLVENT             CDCl3
NS                     16
DS                      0
SWH             8012.820 Hz
FIDRES          0.244532 Hz
AQ             2.0447233 sec
RG                  203.2
DW                 62.400 usec
DE                   6.50 usec
TE                  295.2 K
D1            0.01000000 sec
TD0                     1

======== CHANNEL f1 ========
NUC1                   1H
P1                   7.20 usec
PL1                 -5.00 dB
SFO1          400.1332010 MHz

F2 - Processing parameters
SI                 131072
SF            400.1300161 MHz
WDW                    EM
SSB       0
LB                   0.25 Hz
GB        0
PC                   0.20
```

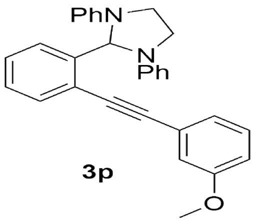

3p

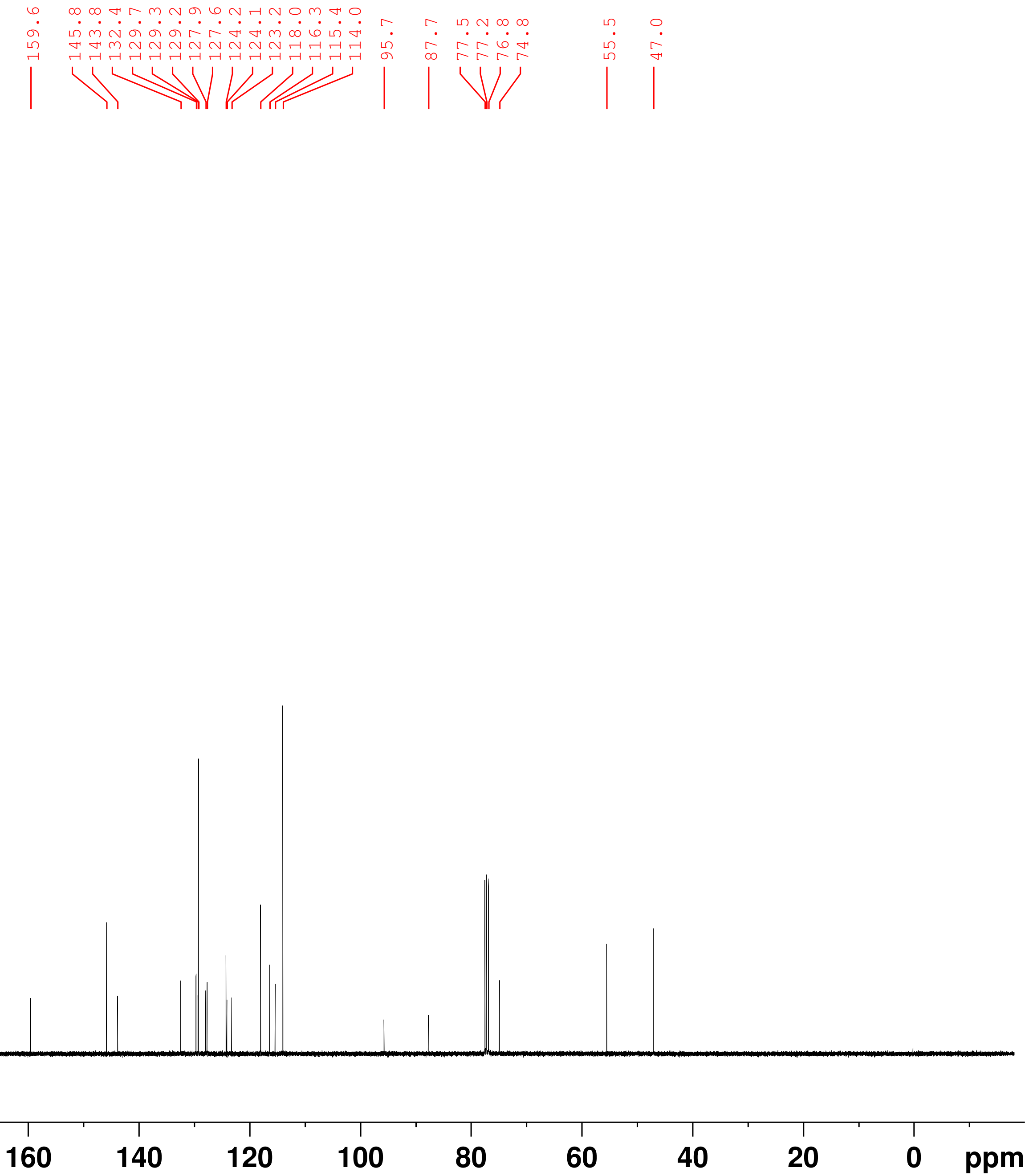

BRUKER

Current Data Parameters

NAME	13C_ST-5-189
EXPNO	1
PROCNO	1

F2 - Acquisition Parameters

Date_	20210804
Time	14.53 h
INSTRUM	Avance
PROBHD	Z167430_0032 (
PULPROG	zgpg30
TD	65536
SOLVENT	CDCl3
NS	100
DS	4
SWH	23809.523 Hz
FIDRES	0.726609 Hz
AQ	1.3762560 sec
RG	3.25
DW	21.000 usec
DE	19.29 usec
TE	298.0 K
D1	2.00000000 sec
D11	0.03000000 sec
TD0	1
SFO1	100.6655806 MHz
NUC1	13C
P0	3.33 usec
P1	10.00 usec
PLW1	39.31399918 W
SFO2	400.3016012 MHz
NUC2	1H
CPDPRG[2	waltz64
PCPD2	80.00 usec
PLW2	8.80000019 W
PLW12	0.20176961 W
PLW13	0.10112690 W

F2 - Processing parameters

SI	131072
SF	100.6555053 MHz
WDW	no
SSB	0
LB	0 Hz
GB	0
PC	1.40

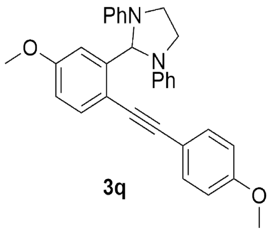

3q

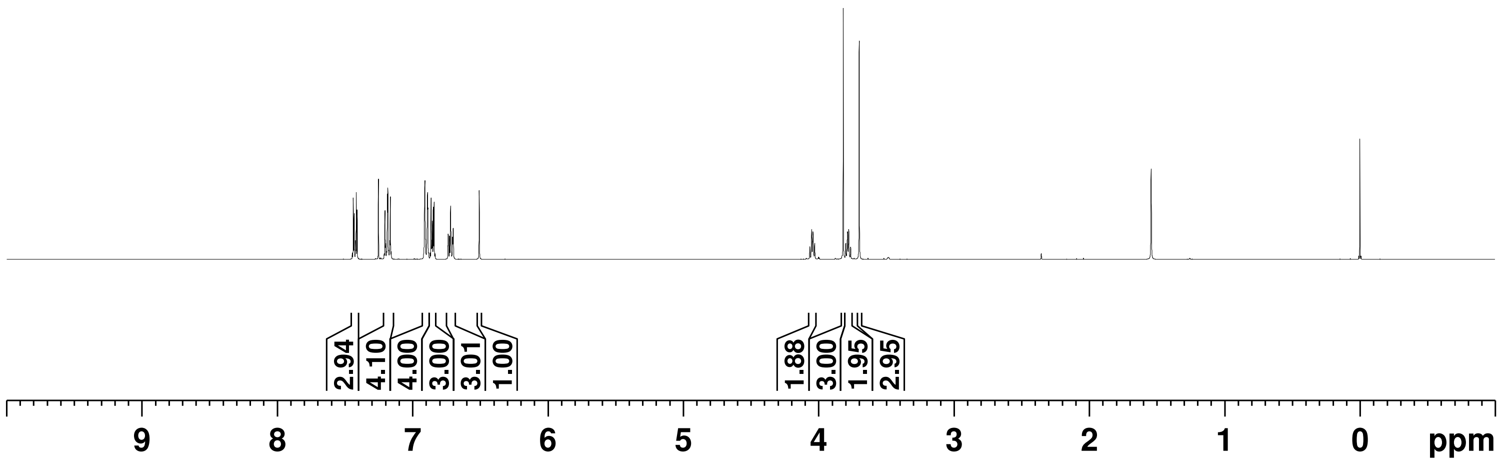

BRUKER

Current Data Parameters
NAME 1H_ST-6-93
EXPNO 1
PROCNO 1

F2 - Acquisition Parameters
Date_ 20210629
Time 10.25 h
INSTRUM Avance
PROBHD Z167430_0032 (
PULPROG zg30
TD 65536
SOLVENT CDCl3
NS 16
DS 2
SWH 8196.722 Hz
FIDRES 0.250144 Hz
AQ 3.9976959 sec
RG 101
DW 61.000 usec
DE 13.20 usec
TE 298.0 K
D1 1.00000000 sec
TD0 1
SFO1 400.3024719 MHz
NUC1 1H
P0 4.00 usec
P1 12.00 usec
PLW1 8.80000019 W

F2 - Processing parameters
SI 65536
SF 400.3000117 MHz
WDW no
SSB 0
LB 0 Hz
GB 0
PC 1.00

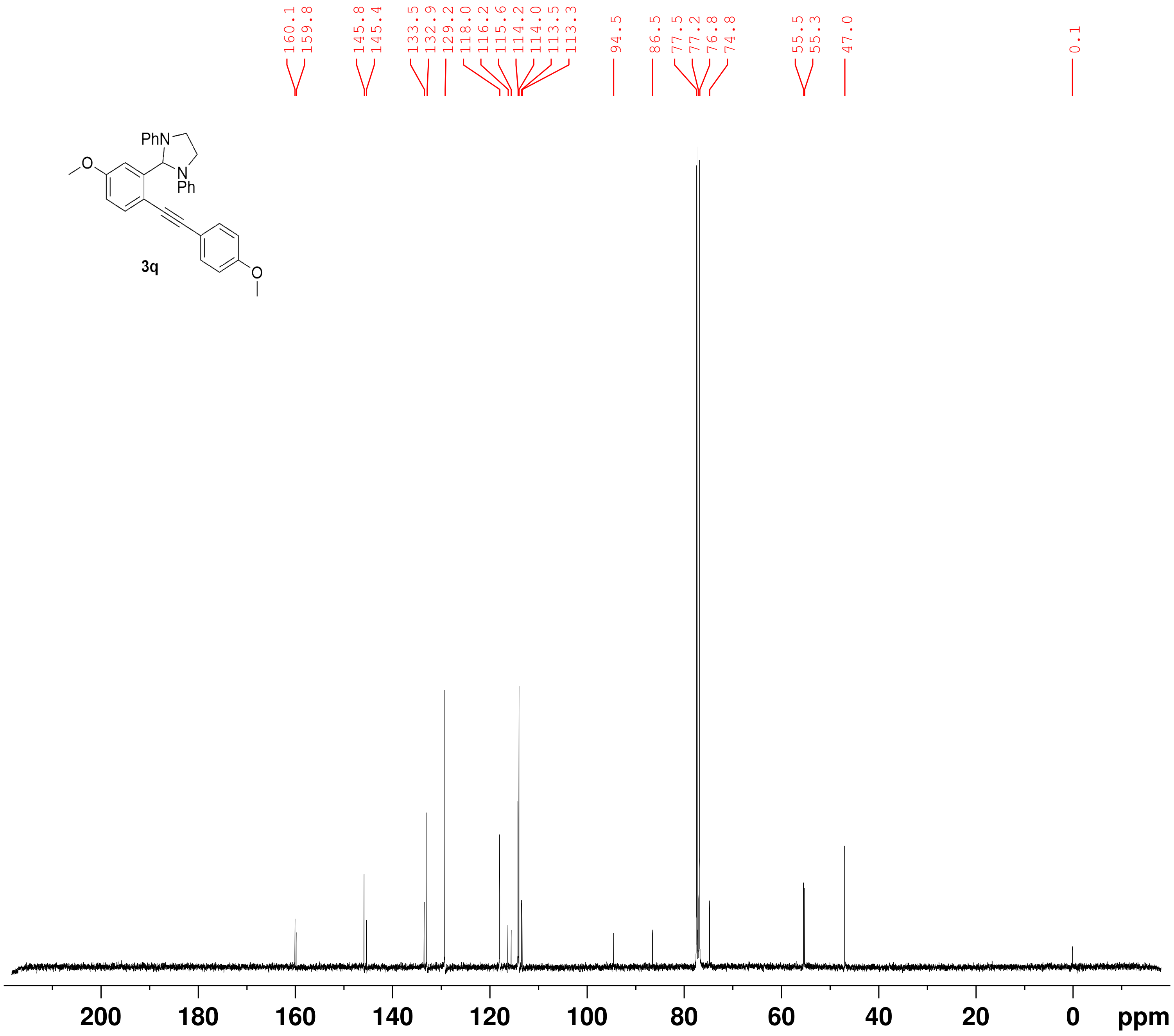

BRUKER

Current Data Parameters
NAME 13C_ST-6-93
EXPNO 1
PROCNO 1

F2 - Acquisition Parameters
Date_ 20210629
Time 10.39 h
INSTRUM Avance
PROBHD Z167430_0032 (
PULPROG zgpg30
TD 65536
SOLVENT CDCl3
NS 200
DS 4
SWH 23809.523 Hz
FIDRES 0.726609 Hz
AQ 1.3762560 sec
RG 3.25
DW 21.000 usec
DE 19.29 usec
TE 298.0 K
D1 2.00000000 sec
D11 0.03000000 sec
TD0 1
SFO1 100.6655806 MHz
NUC1 13C
P0 3.33 usec
P1 10.00 usec
PLW1 39.31399918 W
SFO2 400.3016012 MHz
NUC2 1H
CPDPRG[2 waltz64
PCPD2 80.00 usec
PLW2 8.80000019 W
PLW12 0.20176961 W
PLW13 0.10112690 W

F2 - Processing parameters
SI 131072
SF 100.6555023 MHz
WDW EM
SSB 0
LB 1.00 Hz
GB 0
PC 1.40

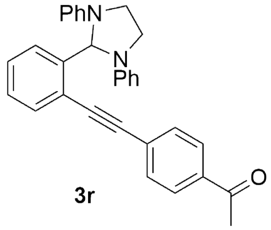

3r

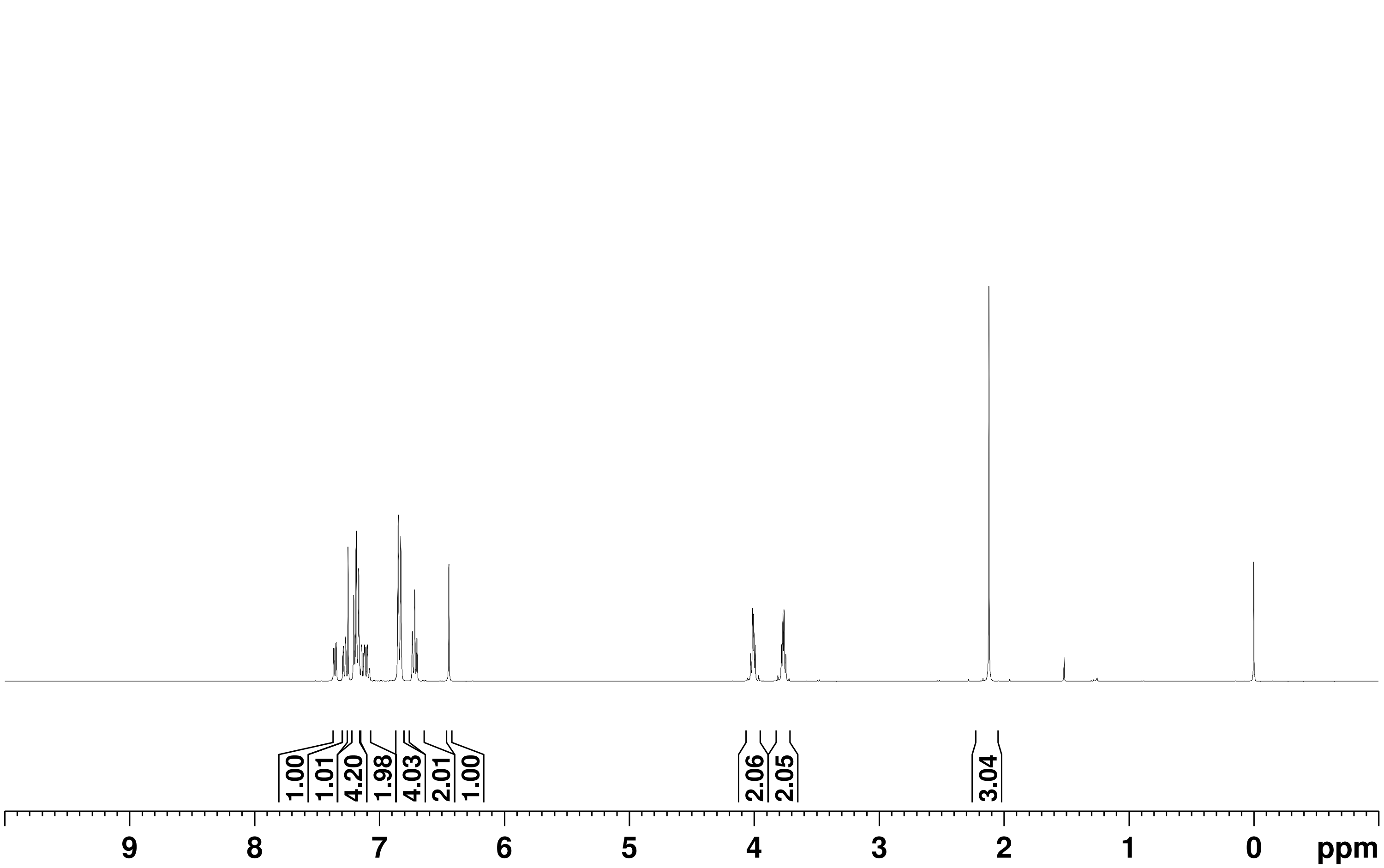


```
Current Data Parameters
NAME          1H ST-07-169
EXPNO                    1
PROCNO                   1

F2 - Acquisition Parameters
Date_             20220427
Time                 16.37 h
INSTRUM             Avance
PROBHD    Z167430_0032 (
PULPROG               zg30
TD                   65536
SOLVENT              CDCl3
NS                      16
DS                       0
SWH               8196.722 Hz
FIDRES            0.250144 Hz
AQ               3.9976959 sec
RG                     101
DW                  61.000 usec
DE                   13.20 usec
TE                   298.0 K
D1              0.10000000 sec
TD0                      1
SFO1           400.3024719 MHz
NUC1                    1H
P0                    4.00 usec
P1                   12.00 usec
PLW1            8.80000019 W

F2 - Processing parameters
SI                   65536
SF             400.3000135 MHz
WDW                     EM
SSB         0
LB                    0.30 Hz
GB          0
PC                    1.00
```

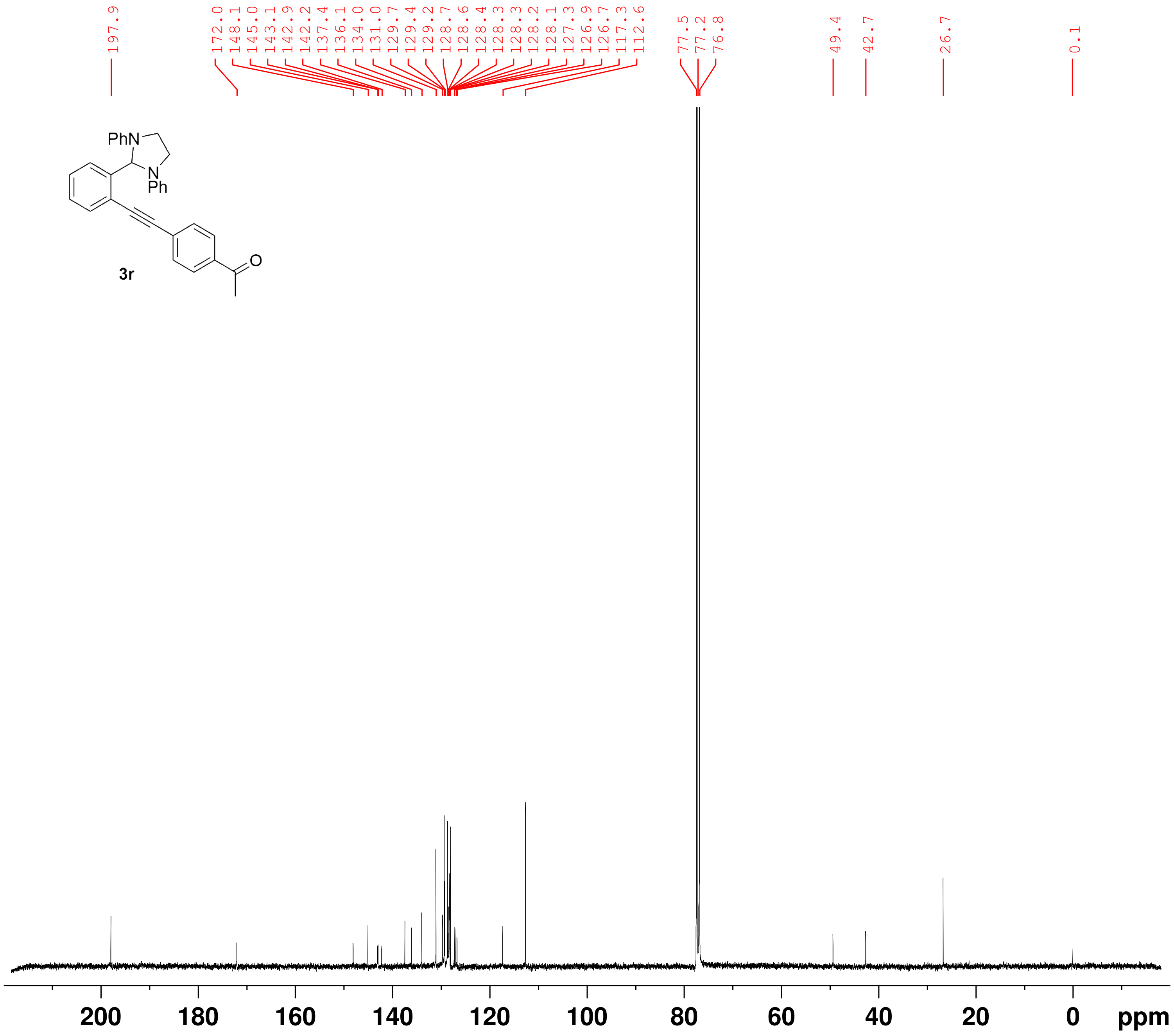


```
Current Data Parameters
NAME          13C_ST-7-167
EXPNO                    2
PROCNO                   1

F2 - Acquisition Parameters
Date_             20220428
Time                 11.44 h
INSTRUM             Avance
PROBHD     Z167430_0032 (
PULPROG            zgpg30
TD                   65536
SOLVENT              CDCl3
NS                     256
DS                       4
SWH              23809.523 Hz
FIDRES            0.726609 Hz
AQ               1.3762560 sec
RG                    3.25
DW                  21.000 usec
DE                   19.29 usec
TE                   298.0 K
D1              3.00000000 sec
D11             0.03000000 sec
TD0                      1
SFO1           100.6655806 MHz
NUC1                   13C
P0                    3.33 usec
P1                   10.00 usec
PLW1           39.31399918 W
SFO2           400.3016012 MHz
NUC2                    1H
CPDPRG[2           waltz64
PCPD2                80.00 usec
PLW2            8.80000019 W
PLW12           0.20176961 W
PLW13           0.10112690 W

F2 - Processing parameters
SI                  131072
SF             100.6555038 MHz
WDW                     EM
SSB      0
LB                    1.00 Hz
GB       0
PC                    1.40
```

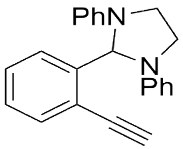

3s

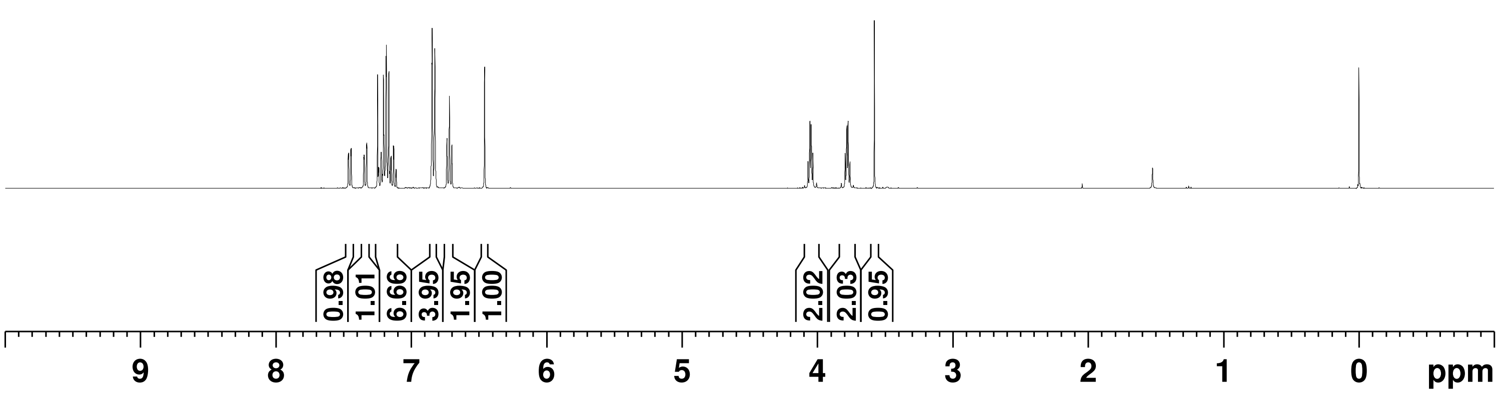

Current Data Parameters
NAME pig-st-4-253-1H-re
EXPNO 1
PROCNO 1

F2 - Acquisition Parameters
Date_ 20200901
Time 12.24 h
INSTRUM spect
PROBHD Z104450_0192 (
PULPROG zg30
TD 65536
SOLVENT CDCl3
NS 16
DS 2
SWH 8012.820 Hz
FIDRES 0.244532 Hz
AQ 4.0894465 sec
RG 256
DW 62.400 usec
DE 16.92 usec
TE 294.4 K
D1 1.00000000 sec
TD0 1
SFO1 400.1324708 MHz
NUC1 1H
P0 5.00 usec
P1 15.00 usec
PLW1 8.47000027 W

F2 - Processing parameters
SI 65536
SF 400.1300145 MHz
WDW EM
SSB 0
LB 0.30 Hz
GB 0
PC 1.00

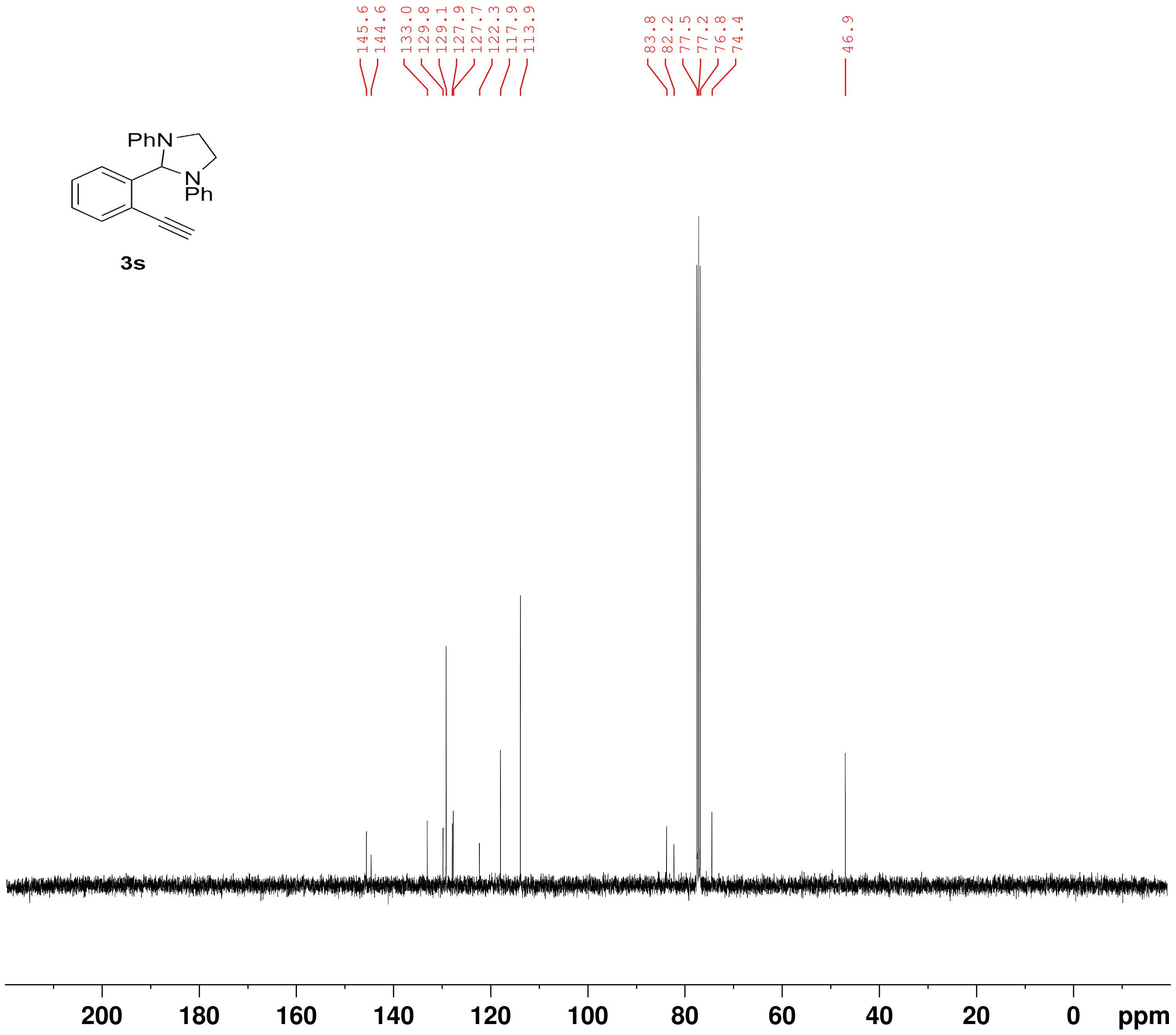

BRUKER

```
Current Data Parameters
NAME       pig-st-4-253-13C-re
EXPNO                        1
PROCNO                       1

F2 - Acquisition Parameters
Date_              20200901
Time                  12.34 h
INSTRUM               spect
PROBHD     Z104450_0192 (
PULPROG             zgpg30
TD                   65536
SOLVENT              CDCl3
NS                     100
DS                       2
SWH              24038.461 Hz
FIDRES            0.733596 Hz
AQ               1.3631488 sec
RG                     203
DW                  20.800 usec
DE                    6.50 usec
TE                   295.1 K
D1              2.00000000 sec
D11             0.03000000 sec
TD0                      1
SFO1           100.6228298 MHz
NUC1                   13C
P0                    3.28 usec
P1                    9.85 usec
PLW1           28.63999939 W
SFO2           400.1316005 MHz
NUC2                    1H
CPDPRG[2           waltz65
PCPD2                90.00 usec
PLW2            8.47000027 W
PLW12           0.23528001 W
PLW13           0.11834000 W

F2 - Processing parameters
SI                   32768
SF             100.6127571 MHz
WDW                     EM
SSB        0
LB                    1.00 Hz
GB         0
PC                    1.40
```

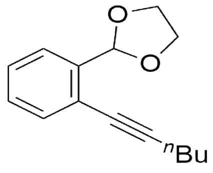

3t

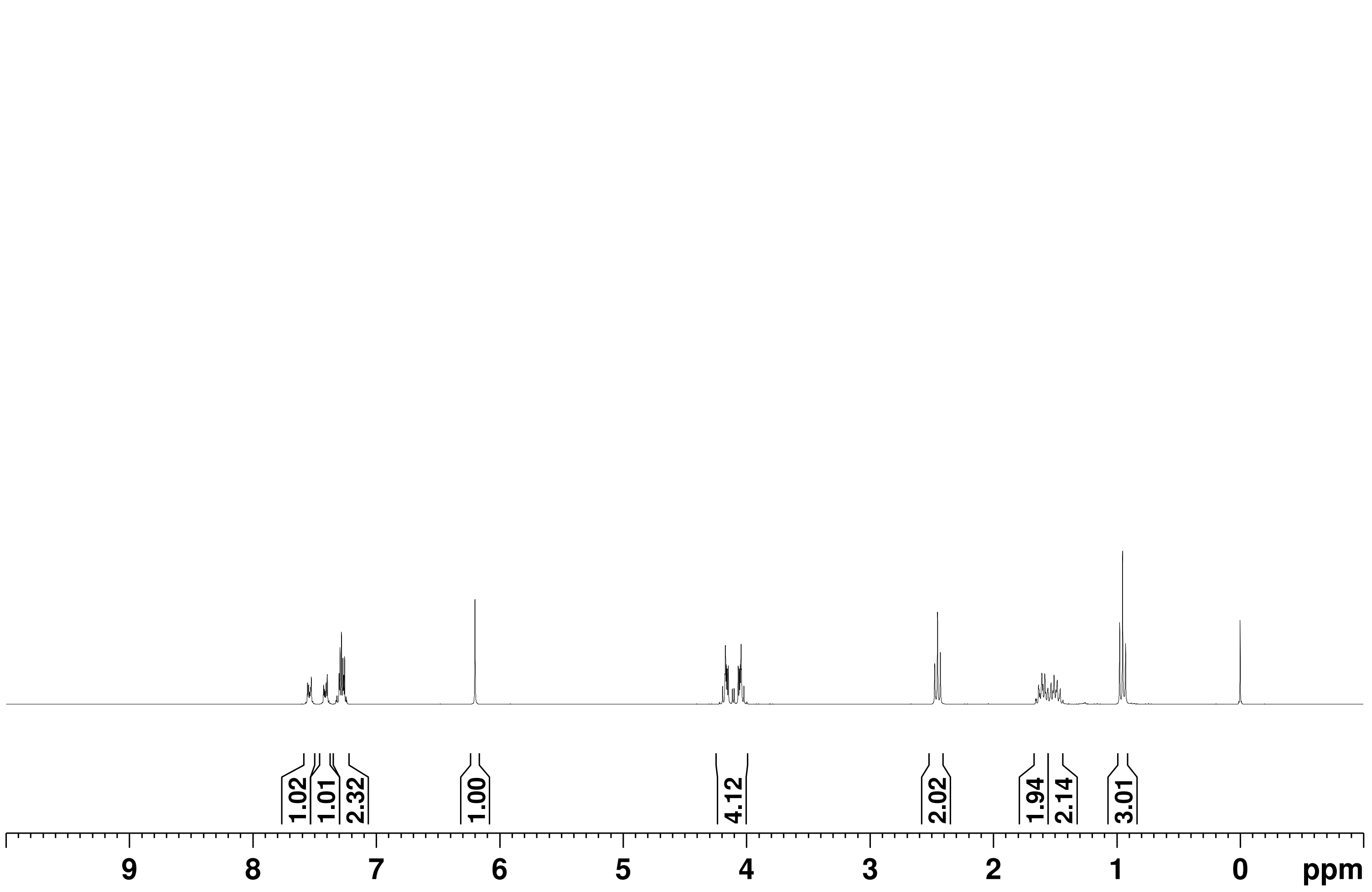


```
Current Data Parameters
NAME        pig-St-5-111-1H
EXPNO                     1
PROCNO                    1

F2 - Acquisition Parameters
Date_              20210108
Time                  14.20
INSTRUM          FOURIER300
PROBHD    5 mm DUL 13C-1
PULPROG                zg30
TD                    65536
SOLVENT               CDCl3
NS                       32
DS                        4
SWH               6103.516 Hz
FIDRES            0.093132 Hz
AQ               5.3687091 sec
RG                  46.9159
DW                   81.920 usec
DE                     6.50 usec
TE                    300.0 K
D1               1.00000000 sec
TD0                       1

======== CHANNEL f1 ========
SFO1           300.1818537 MHz
NUC1                     1H
P1                    15.00 usec
PLW1            13.80399990 W

F2 - Processing parameters
SI                    65536
SF             300.1800061 MHz
WDW                      EM
SSB      0
LB                     0.30 Hz
GB       0
PC                     1.00
```

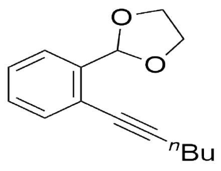

3t

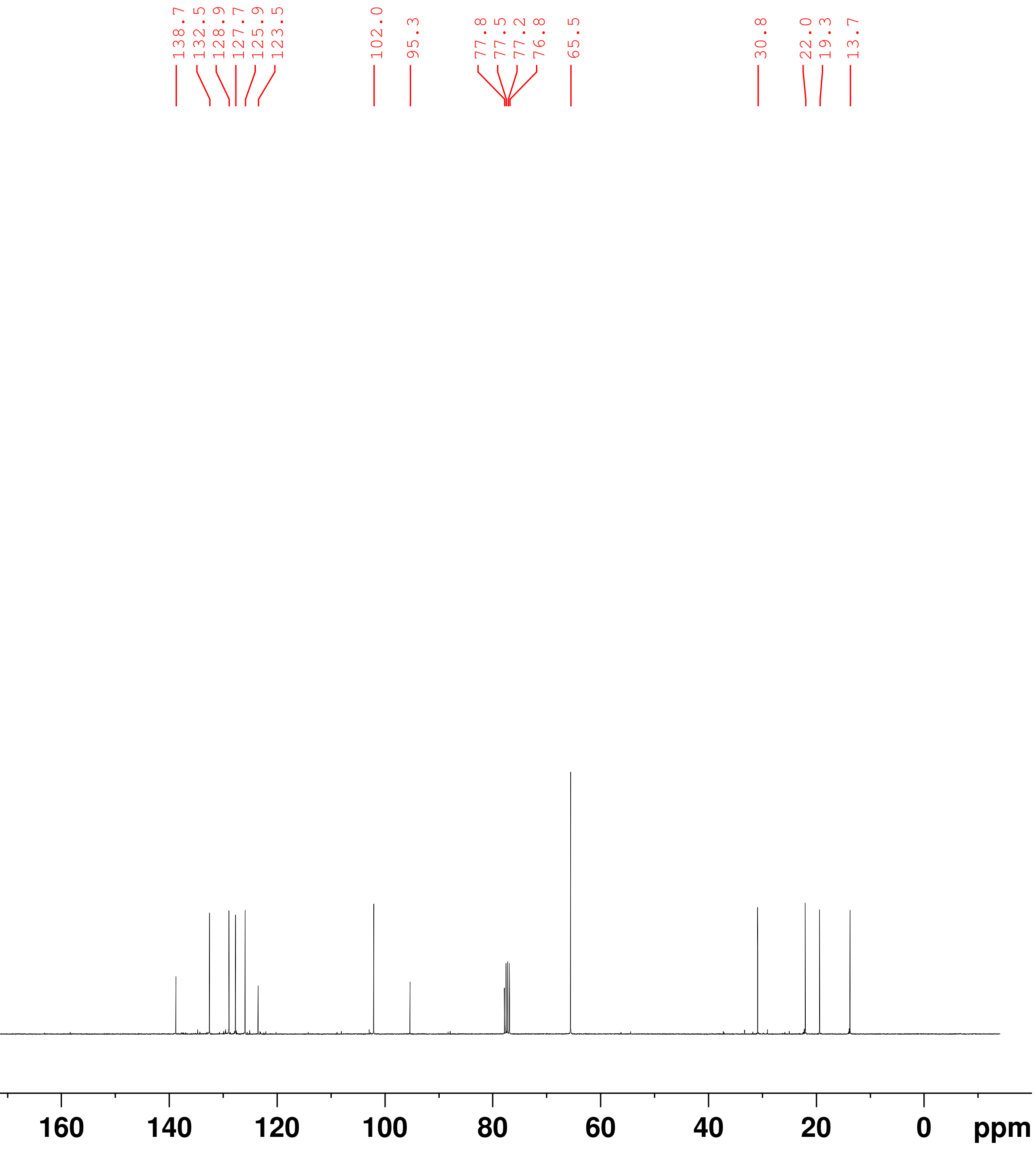

Current Data Parameters

NAME	13C_ST-5-111-ree
EXPNO	1
PROCNO	1

F2 - Acquisition Parameters

Date_	20220112
Time	11.55 h
INSTRUM	Avance
PROBHD	Z167430_0032 (
PULPROG	zgpg30
TD	65536
SOLVENT	CDCl3
NS	64
DS	4
SWH	25000.000 Hz
FIDRES	0.762939 Hz
AQ	1.3107200 sec
RG	3.25
DW	20.000 usec
DE	18.29 usec
TE	298.0 K
D1	2.00000000 sec
D11	0.03000000 sec
TD0	1
SFO1	100.6665872 MHz
NUC1	13C
P0	3.33 usec
P1	10.00 usec
PLW1	39.31399918 W
SFO2	400.3016012 MHz
NUC2	1H
CPDPRG[2	waltz64
PCPD2	80.00 usec
PLW2	8.80000019 W
PLW12	0.20176961 W
PLW13	0.10112690 W

F2 - Processing parameters

SI	131072
SF	100.6555157 MHz
WDW	EM
SSB	0
LB	1.00 Hz
GB	0
PC	1.40

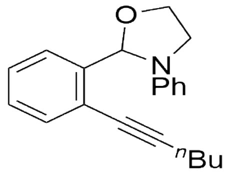

3u

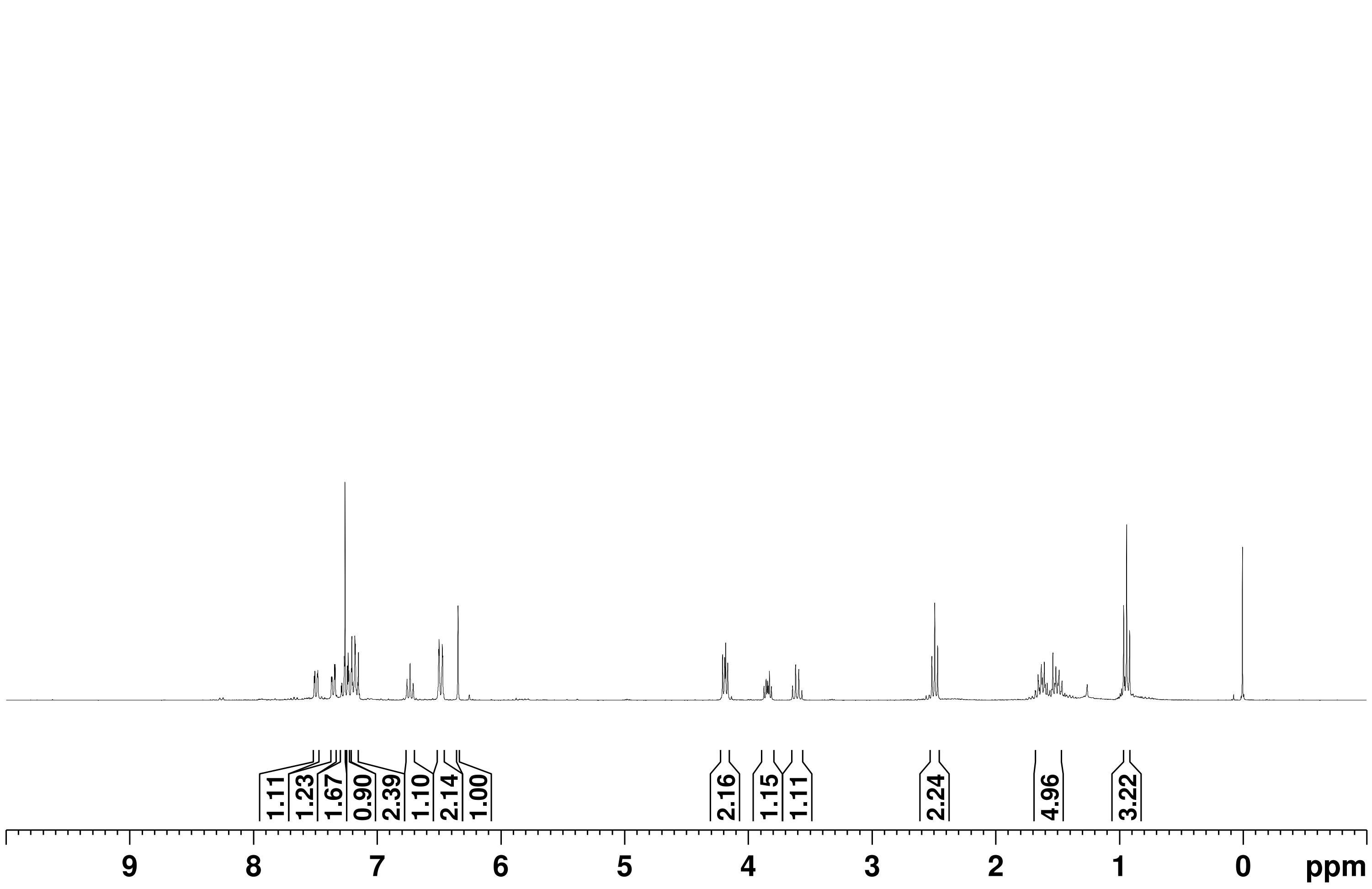

Current Data Parameters
NAME 1H_ST-6-285
EXPNO 1
PROCNO 1

F2 - Acquisition Parameters
Date_ 20220106
Time 13.59 h
INSTRUM Avance NEO nanobay
PROBHD Z104275_0486 (
PULPROG zg30
TD 32768
SOLVENT CDCl3
NS 16
DS 0
SWH 5882.353 Hz
FIDRES 0.359030 Hz
AQ 2.7852800 sec
RG 101
DW 85.000 usec
DE 6.50 usec
TE 298.5 K
D1 0.01000000 sec
TD0 1
SFO1 300.1362005 MHz
NUC1 1H
P0 4.67 usec
P1 14.00 usec
PLW1 6.14340019 W

F2 - Processing parameters
SI 65536
SF 300.1350074 MHz
WDW EM
SSB 0
LB 0.30 Hz
GB 0
PC 1.00

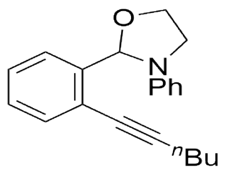

3u

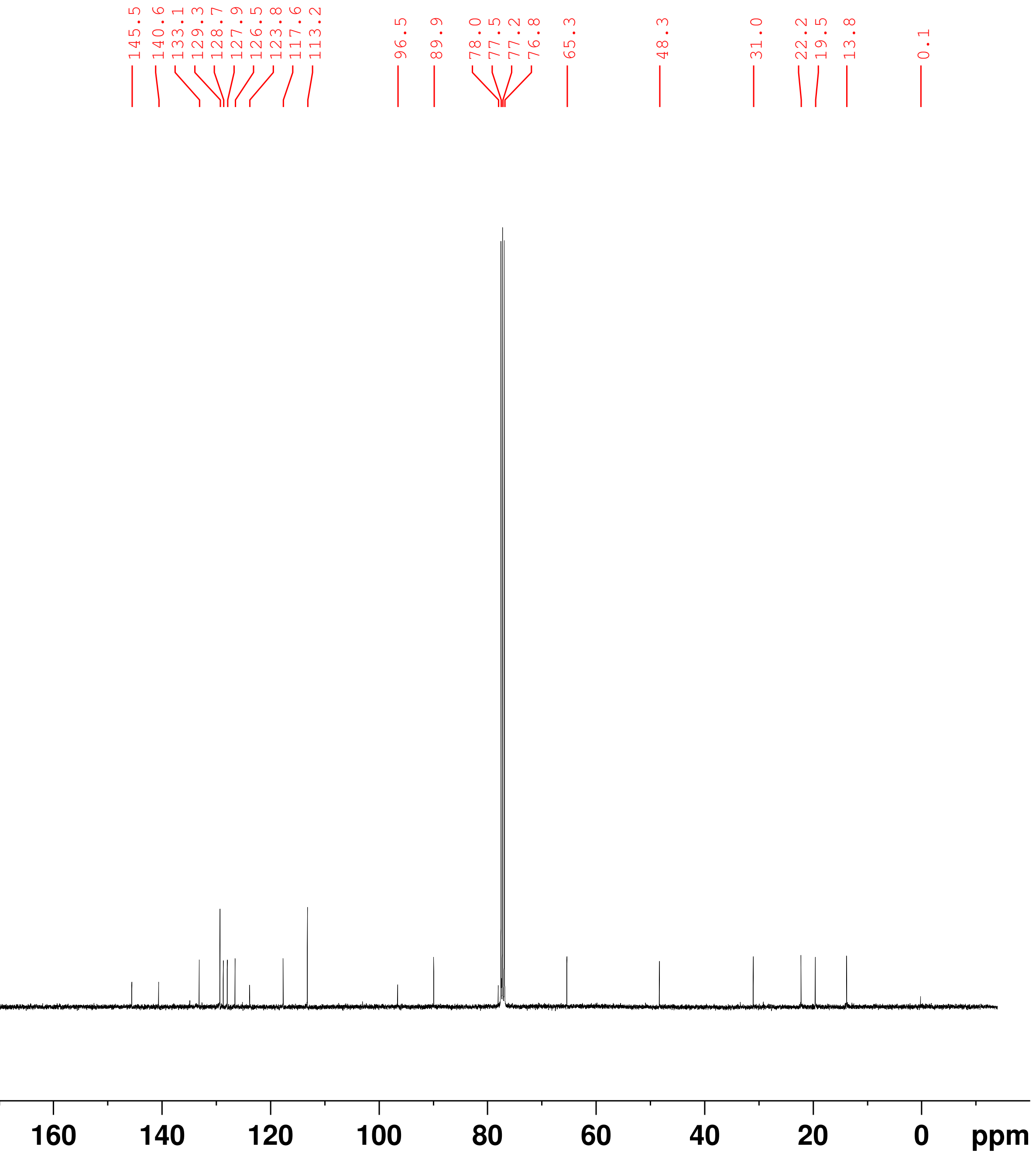

Current Data Parameters
NAME 13C_ST-6-285
EXPNO 1
PROCNO 1

F2 - Acquisition Parameters
Date_ 20220106
Time 14.37 h
INSTRUM Avance
PROBHD Z167430_0032 (
PULPROG zgpg30
TD 65536
SOLVENT CDCl3
NS 200
DS 4
SWH 25000.000 Hz
FIDRES 0.762939 Hz
AQ 1.3107200 sec
RG 3.25
DW 20.000 usec
DE 18.29 usec
TE 298.0 K
D1 2.00000000 sec
D11 0.03000000 sec
TD0 1
SFO1 100.6665872 MHz
NUC1 13C
P0 3.33 usec
P1 10.00 usec
PLW1 39.31399918 W
SFO2 400.3016012 MHz
NUC2 1H
CPDPRG[2 waltz64
PCPD2 80.00 usec
PLW2 8.80000019 W
PLW12 0.20176961 W
PLW13 0.10112690 W

F2 - Processing parameters
SI 131072
SF 100.6555018 MHz
WDW EM
SSB 0
LB 1.00 Hz
GB 0
PC 1.40

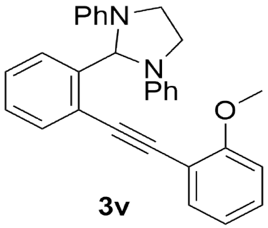

3v

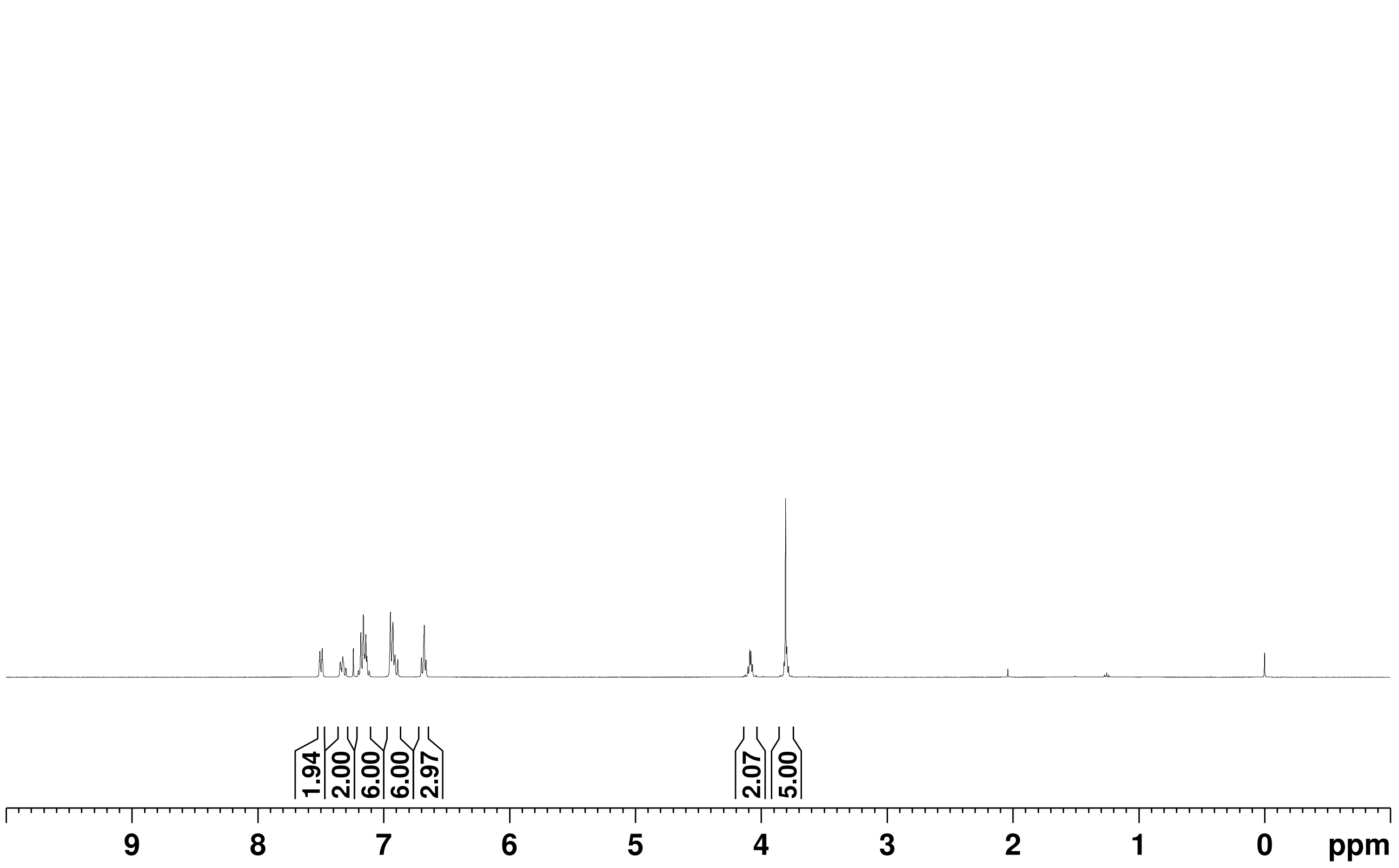


```
Current Data Parameters
NAME       1H_ST-5-213-re
EXPNO                   1
PROCNO                  1

F2 - Acquisition Parameters
Date_            20220111
Time                17.48 h
INSTRUM            Avance
PROBHD    Z167430_0032 (
PULPROG              zg30
TD                  65536
SOLVENT             CDCl3
NS                     16
DS                      2
SWH              8196.722 Hz
FIDRES           0.250144 Hz
AQ              3.9976959 sec
RG                    101
DW                 61.000 usec
DE                  13.20 usec
TE                  298.0 K
D1             1.00000000 sec
TD0                     1
SFO1          400.3024719 MHz
NUC1                   1H
P0                   4.00 usec
P1                  12.00 usec
PLW1           8.80000019 W

F2 - Processing parameters
SI                  65536
SF            400.3000174 MHz
WDW                    EM
SSB      0
LB                   0.30 Hz
GB       0
PC                   1.00
```

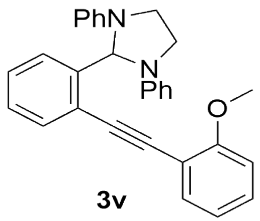

3v

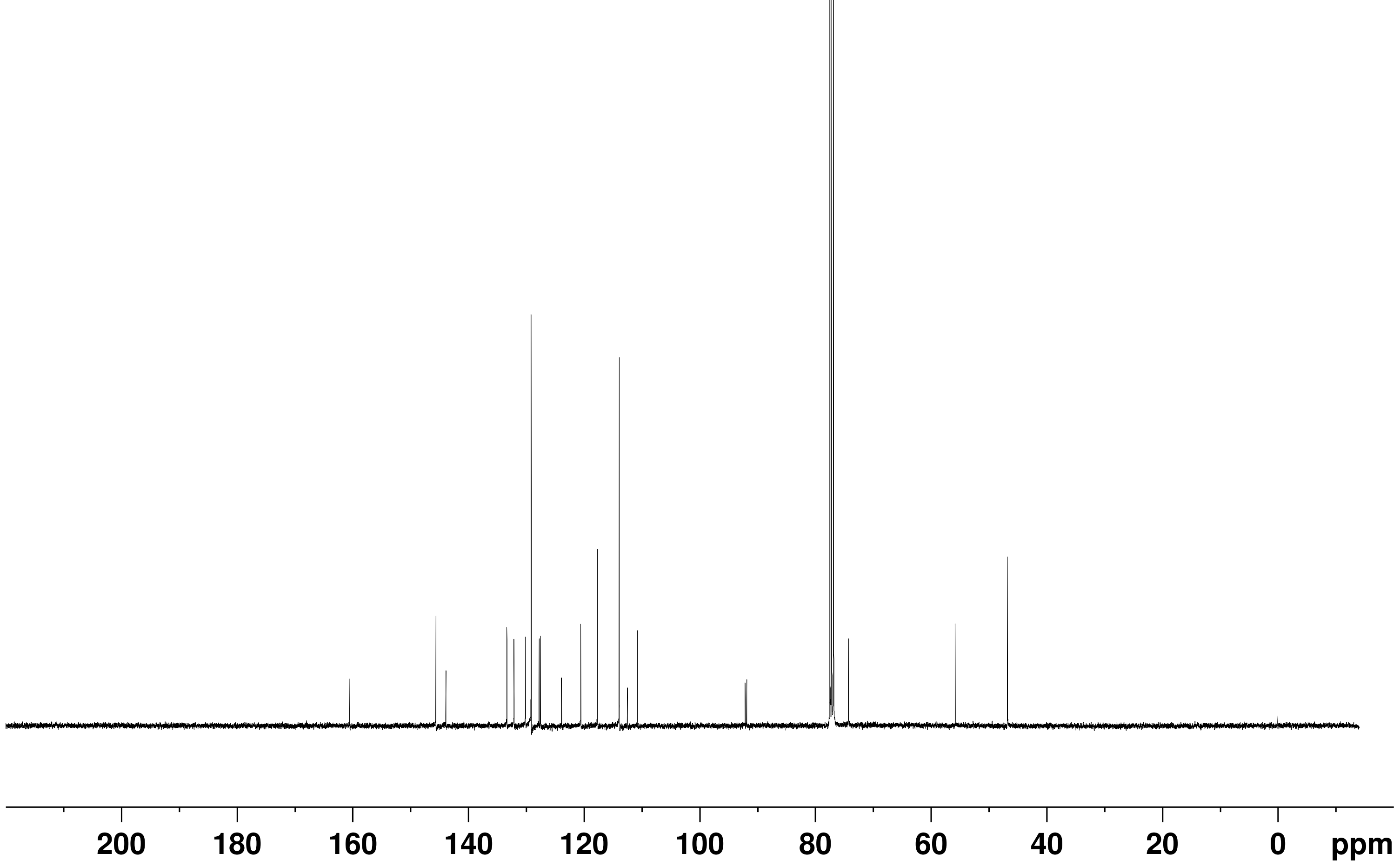

BRUKER

Current Data Parameters
NAME 13C_ST-5-213-re
EXPNO 1
PROCNO 1

F2 - Acquisition Parameters
Date_ 20220111
Time 18.05 h
INSTRUM Avance
PROBHD Z167430_0032 (
PULPROG zgpg30
TD 65536
SOLVENT CDCl3
NS 150
DS 4
SWH 25000.000 Hz
FIDRES 0.762939 Hz
AQ 1.3107200 sec
RG 3.25
DW 20.000 usec
DE 18.29 usec
TE 298.0 K
D1 2.00000000 sec
D11 0.03000000 sec
TD0 1
SFO1 100.6665872 MHz
NUC1 13C
P0 3.33 usec
P1 10.00 usec
PLW1 39.31399918 W
SFO2 400.3016012 MHz
NUC2 1H
CPDPRG[2 waltz64
PCPD2 80.00 usec
PLW2 8.80000019 W
PLW12 0.20176961 W
PLW13 0.10112690 W

F2 - Processing parameters
SI 131072
SF 100.6555045 MHz
WDW EM
SSB 0
LB 1.00 Hz
GB 0
PC 1.40

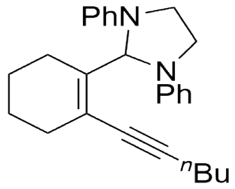

3w

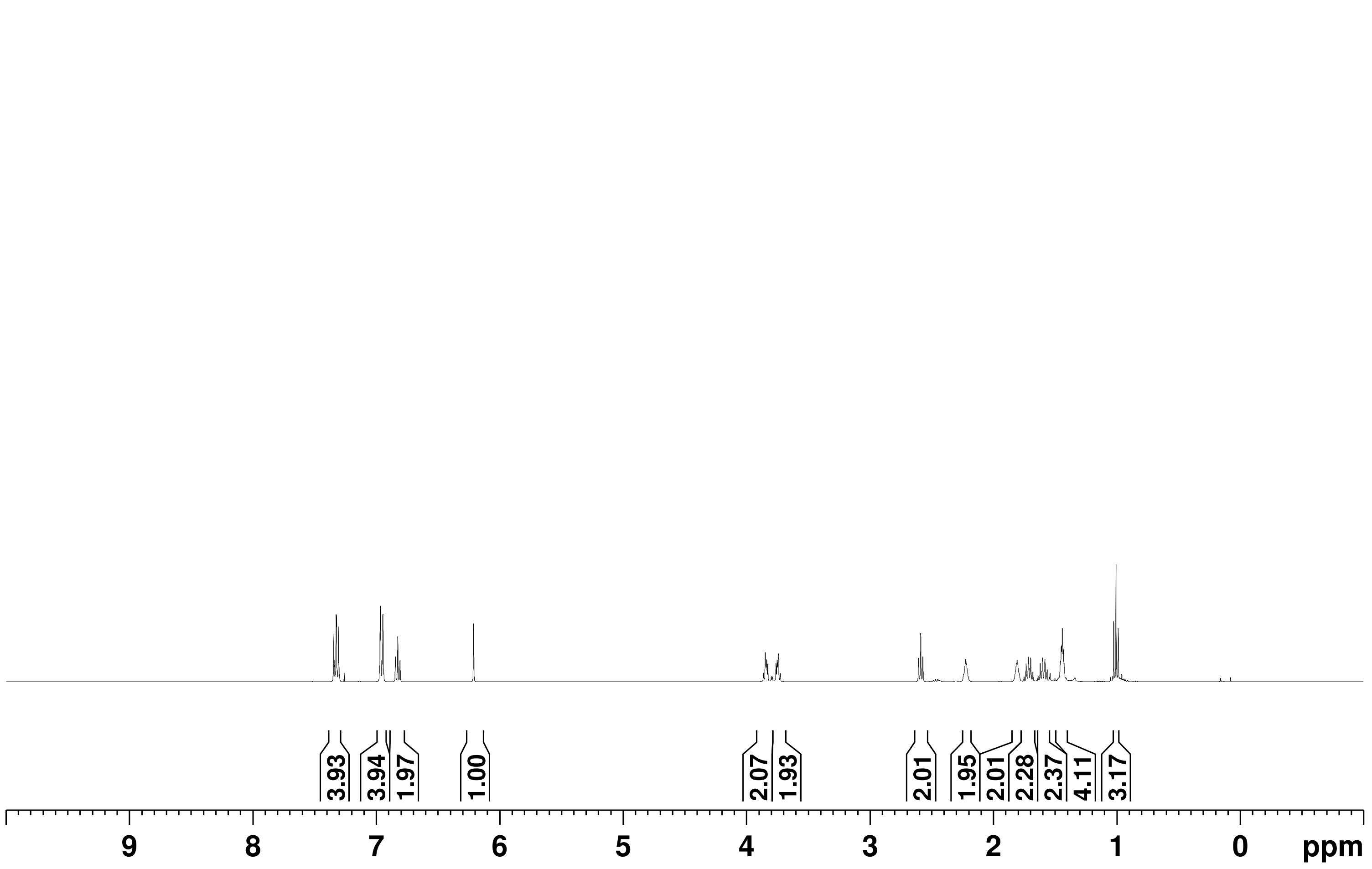


```
Current Data Parameters
NAME       1H_ST-6-65-ree
EXPNO                   2
PROCNO                  1

F2 - Acquisition Parameters
Date_            20210616
Time                 8.45 h
INSTRUM            Avance
PROBHD    Z167430_0032 (
PULPROG              zg30
TD                  65536
SOLVENT             CDCl3
NS                     16
DS                      2
SWH              8196.722 Hz
FIDRES           0.250144 Hz
AQ              3.9976959 sec
RG                36.1407
DW                 61.000 usec
DE                  13.20 usec
TE                  298.0 K
D1             1.00000000 sec
TD0                     1
SFO1          400.3024719 MHz
NUC1                   1H
P0                   4.00 usec
P1                  12.00 usec
PLW1           8.80000019 W

F2 - Processing parameters
SI                  65536
SF            400.3000082 MHz
WDW                    EM
SSB       0
LB                   0.30 Hz
GB        0
PC                   1.00
```

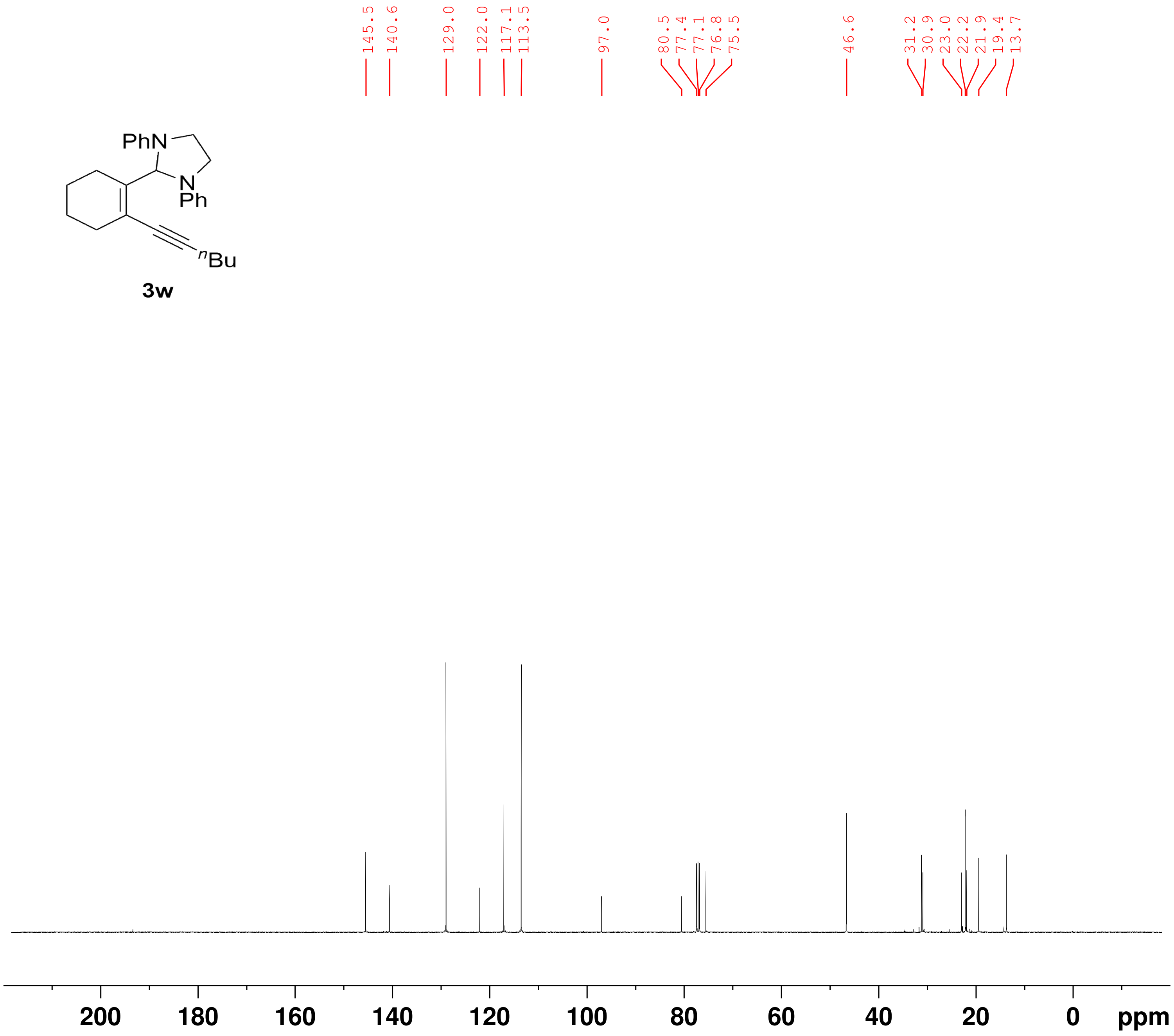

BRUKER

Current Data Parameters
NAME 13C_ST-6-65-ree
EXPNO 2
PROCNO 1

F2 - Acquisition Parameters
Date_ 20210616
Time 8.56 h
INSTRUM Avance
PROBHD Z167430_0032 (
PULPROG zgpg30
TD 65536
SOLVENT CDCl3
NS 100
DS 4
SWH 23809.523 Hz
FIDRES 0.726609 Hz
AQ 1.3762560 sec
RG 3.25
DW 21.000 usec
DE 19.29 usec
TE 298.0 K
D1 2.00000000 sec
D11 0.03000000 sec
TD0 1
SFO1 100.6655806 MHz
NUC1 13C
P0 3.33 usec
P1 10.00 usec
PLW1 39.31399918 W
SFO2 400.3016012 MHz
NUC2 1H
CPDPRG[2 waltz64
PCPD2 80.00 usec
PLW2 8.80000019 W
PLW12 0.20176961 W
PLW13 0.10112690 W

F2 - Processing parameters
SI 131072
SF 100.6555151 MHz
WDW EM
SSB 0
LB 1.00 Hz
GB 0
PC 1.40

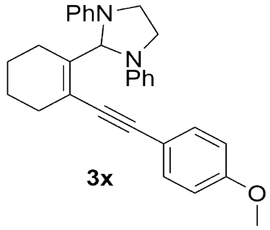

3x

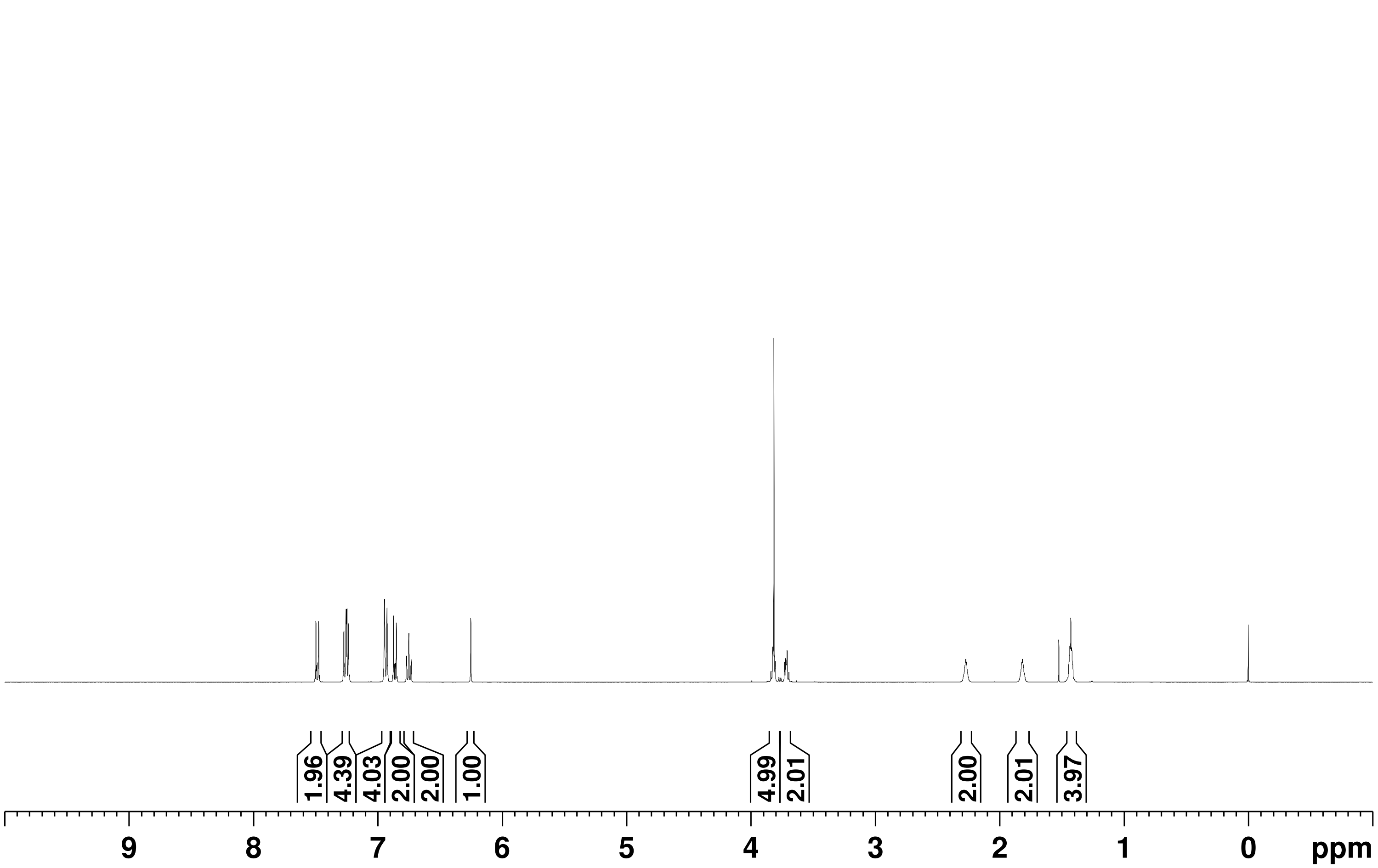

Current Data Parameters
NAME 1H_ST-6-89
EXPNO 1
PROCNO 1

F2 - Acquisition Parameters
Date_ 20210628
Time 16.31 h
INSTRUM Avance
PROBHD Z167430_0032 (
PULPROG zg30
TD 65536
SOLVENT CDCl3
NS 16
DS 2
SWH 8196.722 Hz
FIDRES 0.250144 Hz
AQ 3.9976959 sec
RG 101
DW 61.000 usec
DE 13.20 usec
TE 298.0 K
D1 0.01000000 sec
TD0 1
SFO1 400.3024719 MHz
NUC1 1H
P0 4.00 usec
P1 12.00 usec
PLW1 8.80000019 W

F2 - Processing parameters
SI 65536
SF 400.3000139 MHz
WDW no
SSB 0
LB 0 Hz
GB 0
PC 1.00

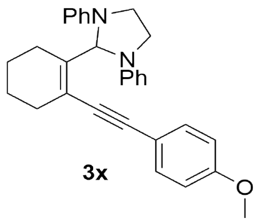

3x

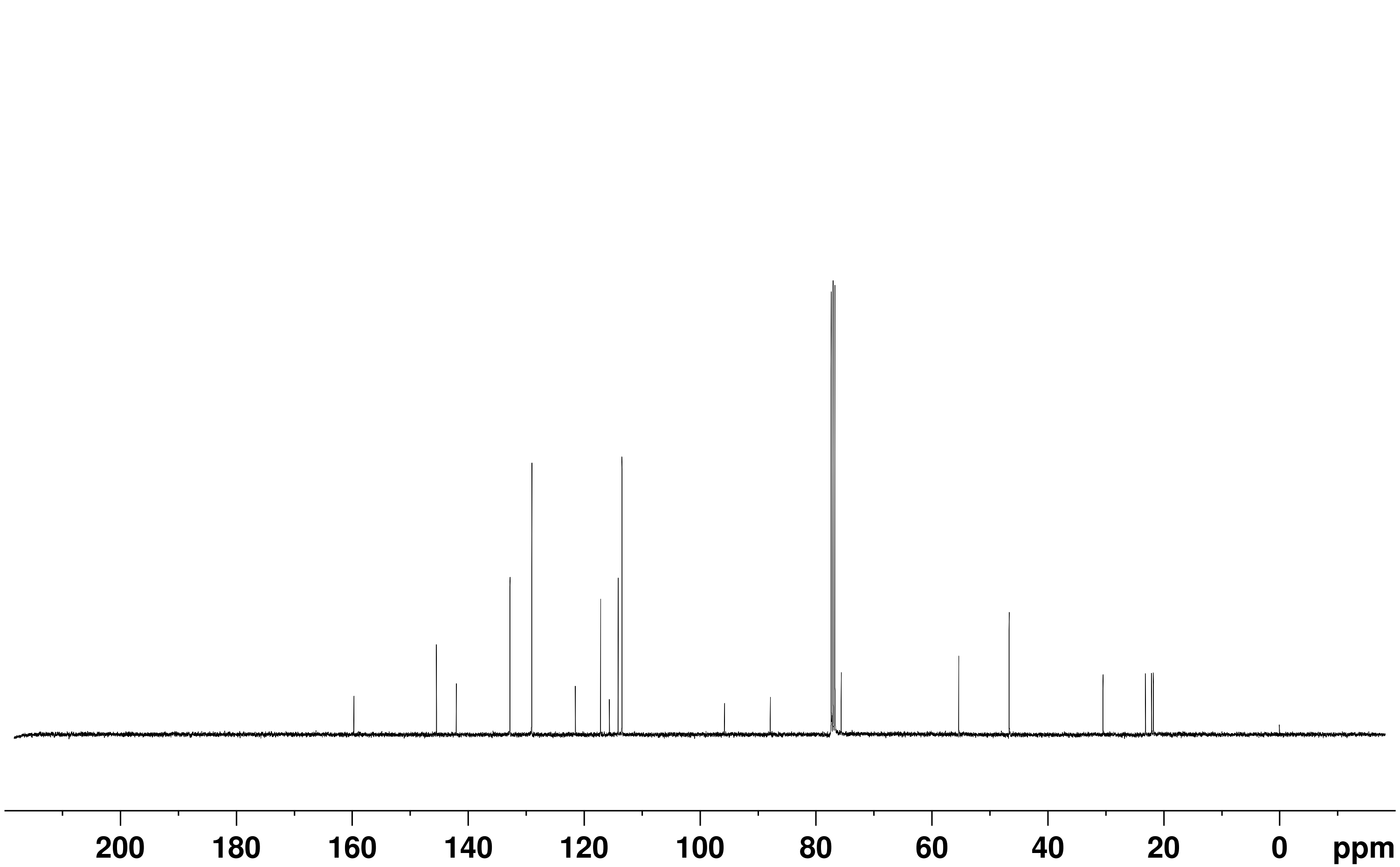

BRUKER

Current Data Parameters
NAME 13C_ST-6-89
EXPNO 1
PROCNO 1

F2 - Acquisition Parameters
Date_ 20210628
Time 16.43 h
INSTRUM Avance
PROBHD Z167430_0032 (
PULPROG zgpg30
TD 65536
SOLVENT CDCl3
NS 150
DS 4
SWH 23809.523 Hz
FIDRES 0.726609 Hz
AQ 1.3762560 sec
RG 3.25
DW 21.000 usec
DE 19.29 usec
TE 298.0 K
D1 2.00000000 sec
D11 0.03000000 sec
TD0 1
SFO1 100.6655806 MHz
NUC1 13C
P0 3.33 usec
P1 10.00 usec
PLW1 39.31399918 W
SFO2 400.3016012 MHz
NUC2 1H
CPDPRG[2 waltz64
PCPD2 80.00 usec
PLW2 8.80000019 W
PLW12 0.20176961 W
PLW13 0.10112690 W

F2 - Processing parameters
SI 131072
SF 100.6555151 MHz
WDW EM
SSB 0
LB 1.00 Hz
GB 0
PC 1.40

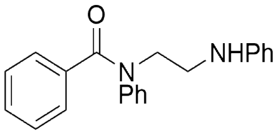

4a

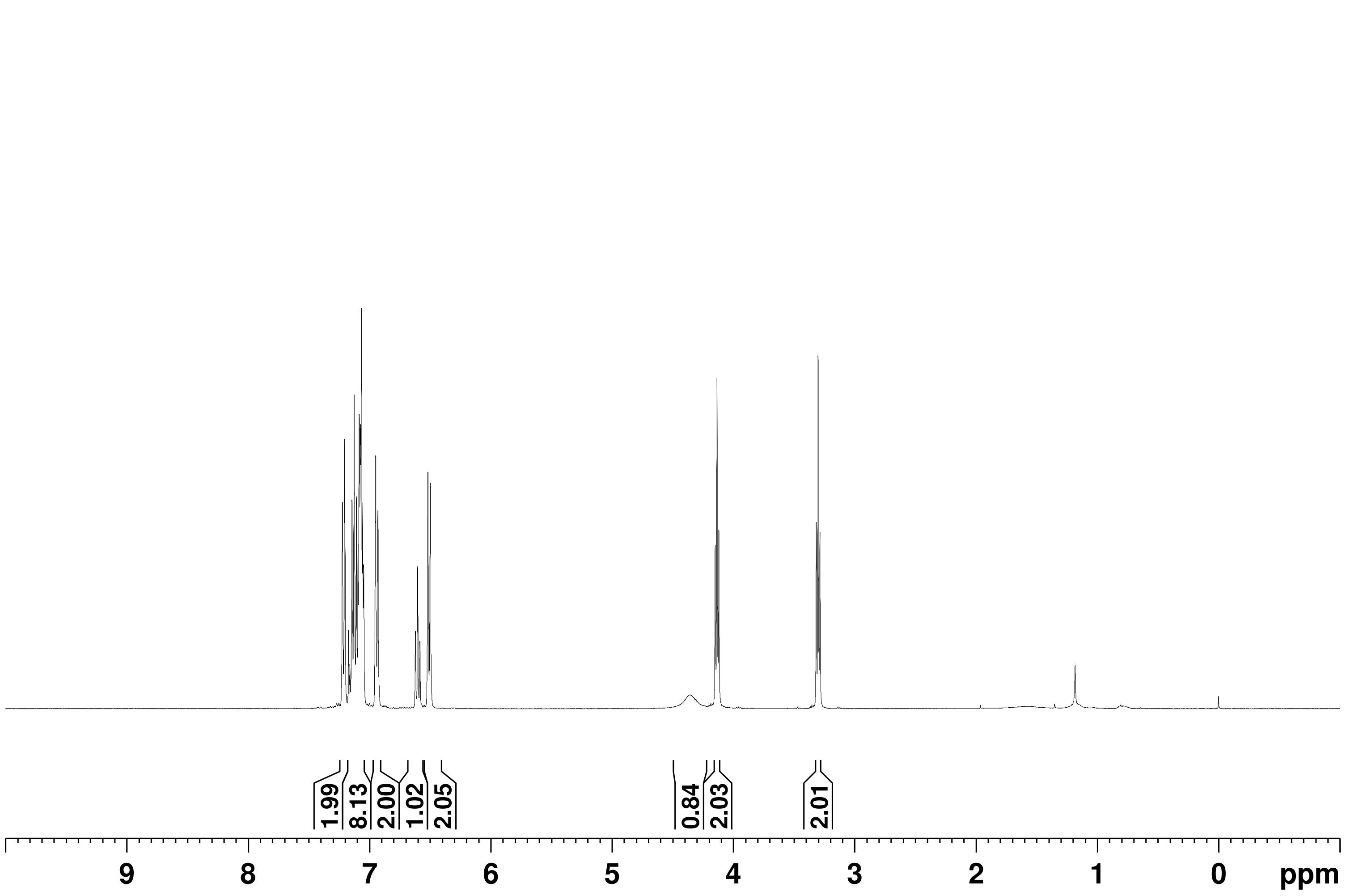

Current Data Parameters
NAME 1H_ST-3-105B-re
EXPNO 1
PROCNO 1

F2 - Acquisition Parameters
Date_ 20190826
Time 10.13
INSTRUM spect
PROBHD 5 mm Multinucl
PULPROG zg30
TD 32768
SOLVENT CDCl3
NS 16
DS 2
SWH 8012.820 Hz
FIDRES 0.244532 Hz
AQ 2.0447233 sec
RG 161.3
DW 62.400 usec
DE 6.50 usec
TE 298.2 K
D1 0.01000000 sec
TD0 1

======== CHANNEL f1 ========
NUC1 1H
P1 7.20 usec
PL1 -5.00 dB
SFO1 400.1332010 MHz

F2 - Processing parameters
SI 131072
SF 400.1300444 MHz
WDW EM
SSB 0
LB 0.25 Hz
GB 0
PC 0.20

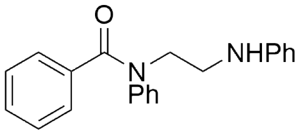

4a

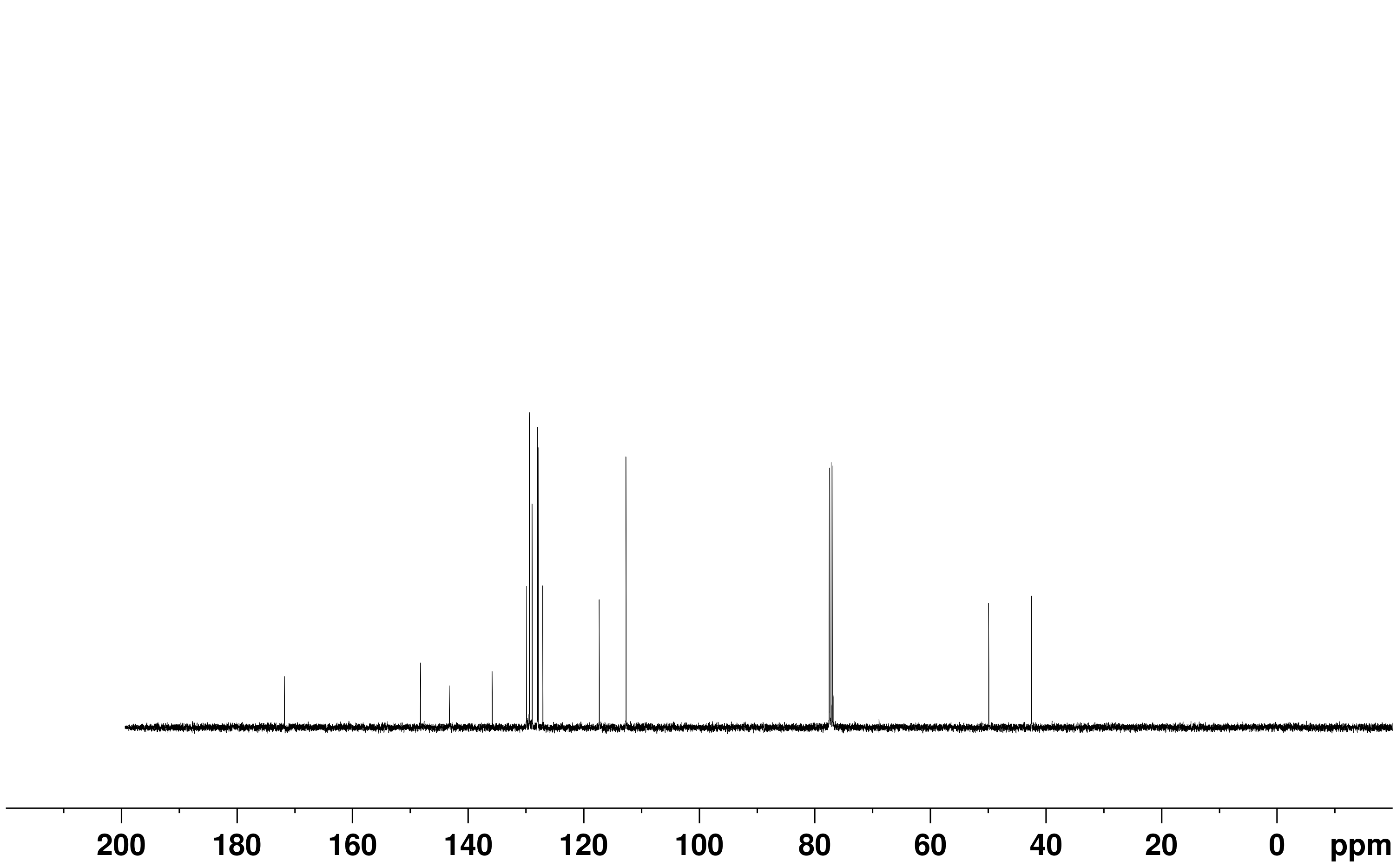


```
Current Data Parameters
NAME      13C_ST-3-105B-re
EXPNO                    2
PROCNO                   1

F2 - Acquisition Parameters
Date_             20190826
Time                 10.26
INSTRUM              spect
PROBHD   5 mm Multinucl
PULPROG             zgdc30
TD                   65536
SOLVENT              CDCl3
NS                     300
DS                       4
SWH          26246.719 Hz
FIDRES        0.400493 Hz
AQ           1.2484608 sec
RG                   406.4
DW              19.050 usec
DE                6.50 usec
TE               298.2 K
D1          0.69999999 sec
d11         0.03000000 sec
TD0                      1

======== CHANNEL f1 ========
NUC1                   13C
P1                8.07 usec
PL1              -6.00 dB
SFO1       100.6196894 MHz

======== CHANNEL f2 ========
CPDPRG[2           waltz16
NUC2                    1H
PCPD2            80.00 usec
PL2       0 dB
PL12             18.00 dB
SFO2       400.1318006 MHz

F2 - Processing parameters
SI                  131072
SF         100.6127588 MHz
WDW                     EM
SSB       0
LB                0.80 Hz
GB        0
PC                    0.50
```

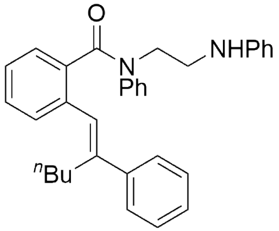

4c

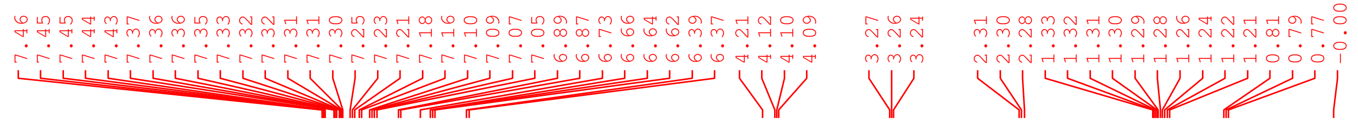
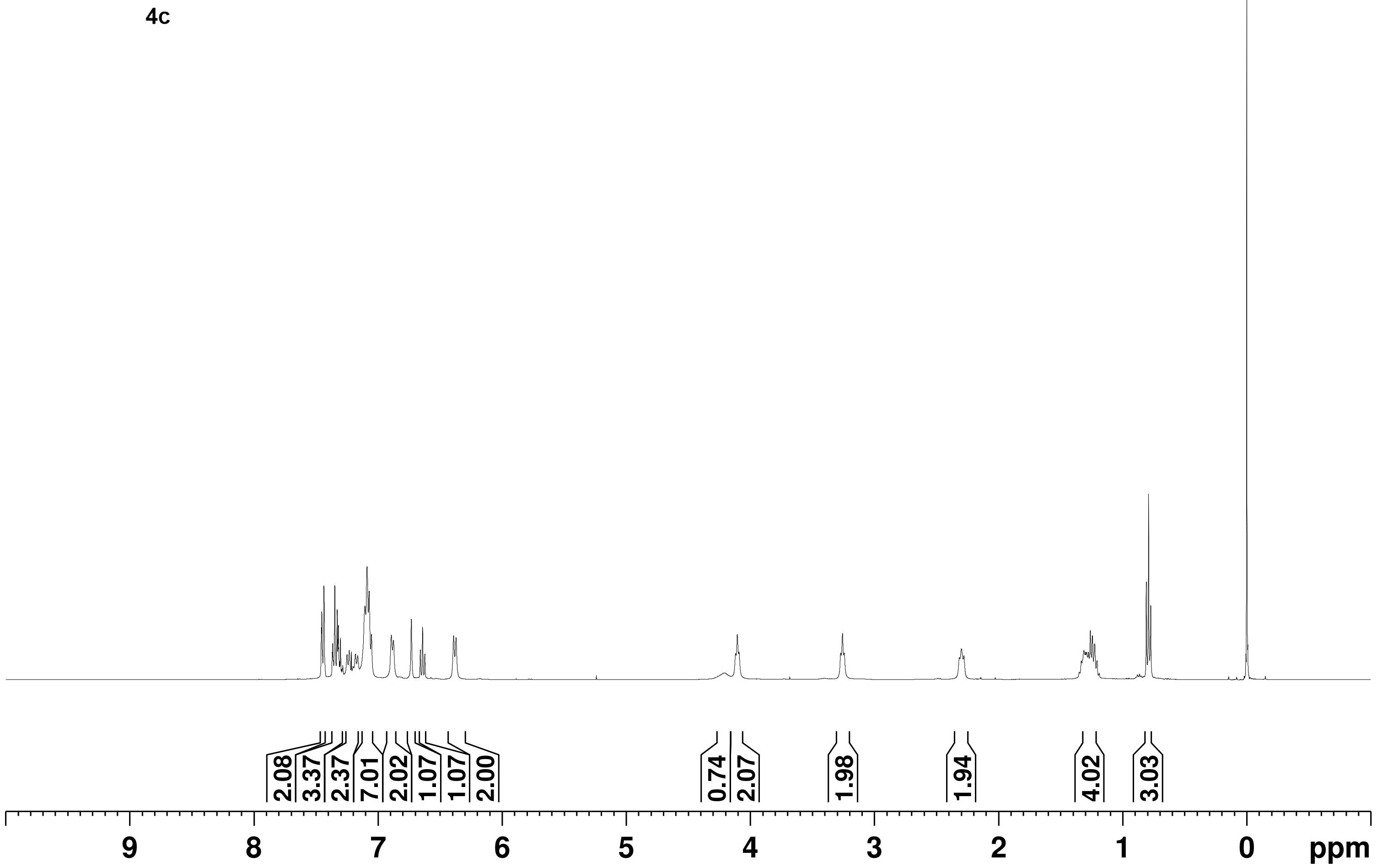

Current Data Parameters
NAME pig-st-4-131-1H
EXPNO 1
PROCNO 1

F2 - Acquisition Parameters
Date_ 20200625
Time 14.51 h
INSTRUM spect
PROBHD Z104450_0192 (
PULPROG zg30
TD 65536
SOLVENT CDCl3
NS 16
DS 2
SWH 8012.820 Hz
FIDRES 0.244532 Hz
AQ 4.0894465 sec
RG 32
DW 62.400 usec
DE 16.92 usec
TE 294.9 K
D1 1.00000000 sec
TD0 1
SFO1 400.1324708 MHz
NUC1 1H
P0 5.00 usec
P1 15.00 usec
PLW1 8.47000027 W

F2 - Processing parameters
SI 65536
SF 400.1300282 MHz
WDW EM
SSB 0
LB 0.30 Hz
GB 0
PC 1.00

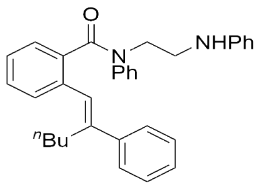

4c

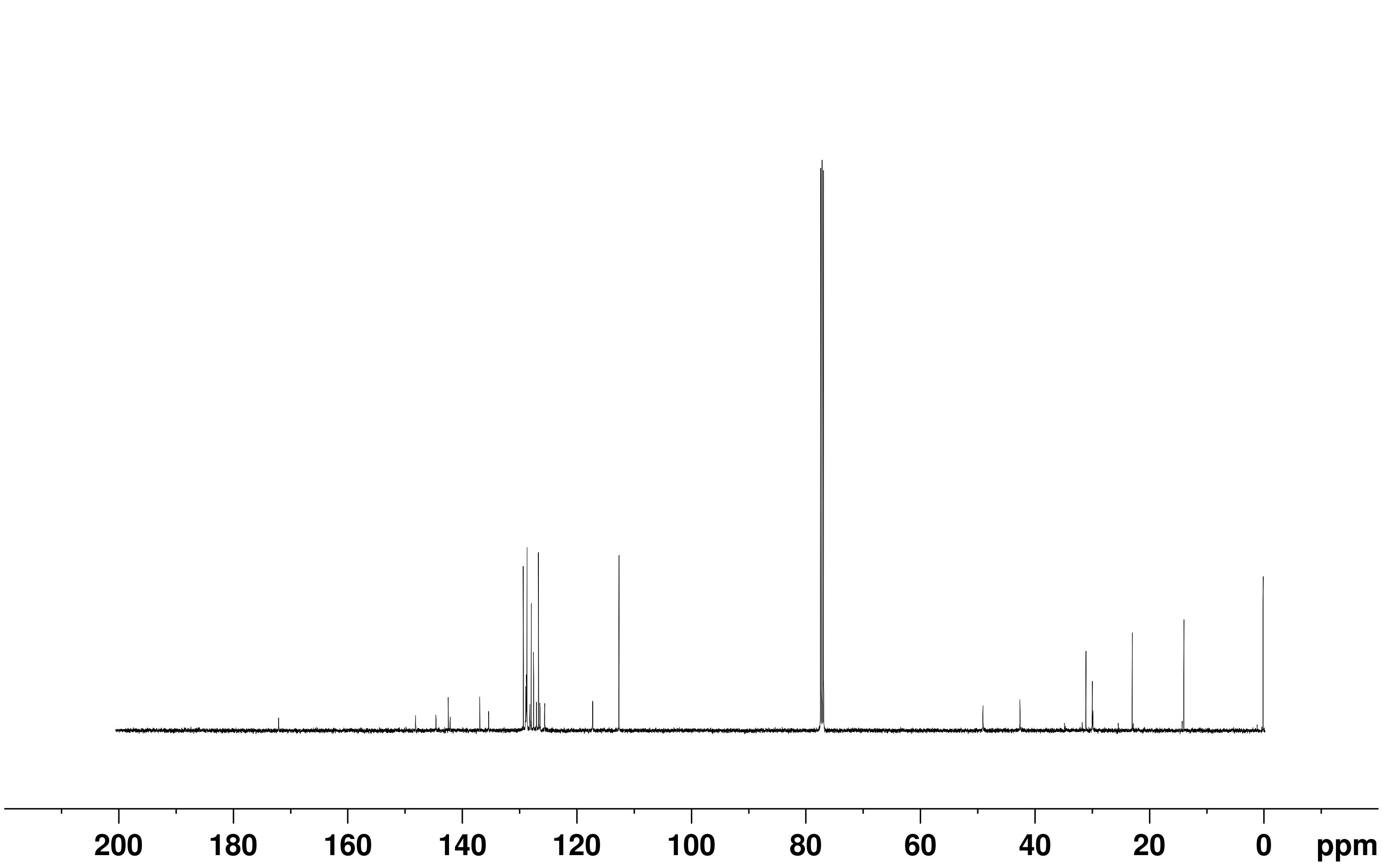

BRUKER

Current Data Parameters
NAME pig-st-4-131-13C
EXPNO 8
PROCNO 1

F2 - Acquisition Parameters
Date_ 20190913
Time 8.06
INSTRUM spect
PROBHD 5 mm PABBO BB-
PULPROG zgpg30
TD 65536
SOLVENT CDCl3
NS 1024
DS 2
SWH 25252.525 Hz
FIDRES 0.385323 Hz
AQ 1.2976128 sec
RG 18390.4
DW 19.800 usec
DE 6.50 usec
TE 298.2 K
D1 1.25000000 sec
D11 0.03000000 sec
TD0 1

======== CHANNEL f1 ========
NUC1 13C
P1 10.00 usec
PL1 -1.70 dB
PL1W 148.61408997 W
SFO1 125.8131151 MHz

======== CHANNEL f2 ========
CPDPRG[2 waltz16
NUC2 1H
PCPD2 80.00 usec
PL2 -1.10 dB
PL12 15.40 dB
PL13 17.40 dB
PL2W 19.41561890 W
PL12W 0.43466163 W
PL13W 0.27425295 W
SFO2 500.3020012 MHz

F2 - Processing parameters
SI 32768
SF 125.8005191 MHz
WDW EM
SSB 0
LB 1.00 Hz
GB 0
PC 1.40

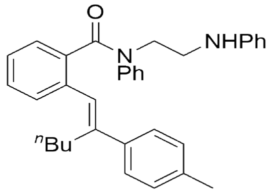

4d

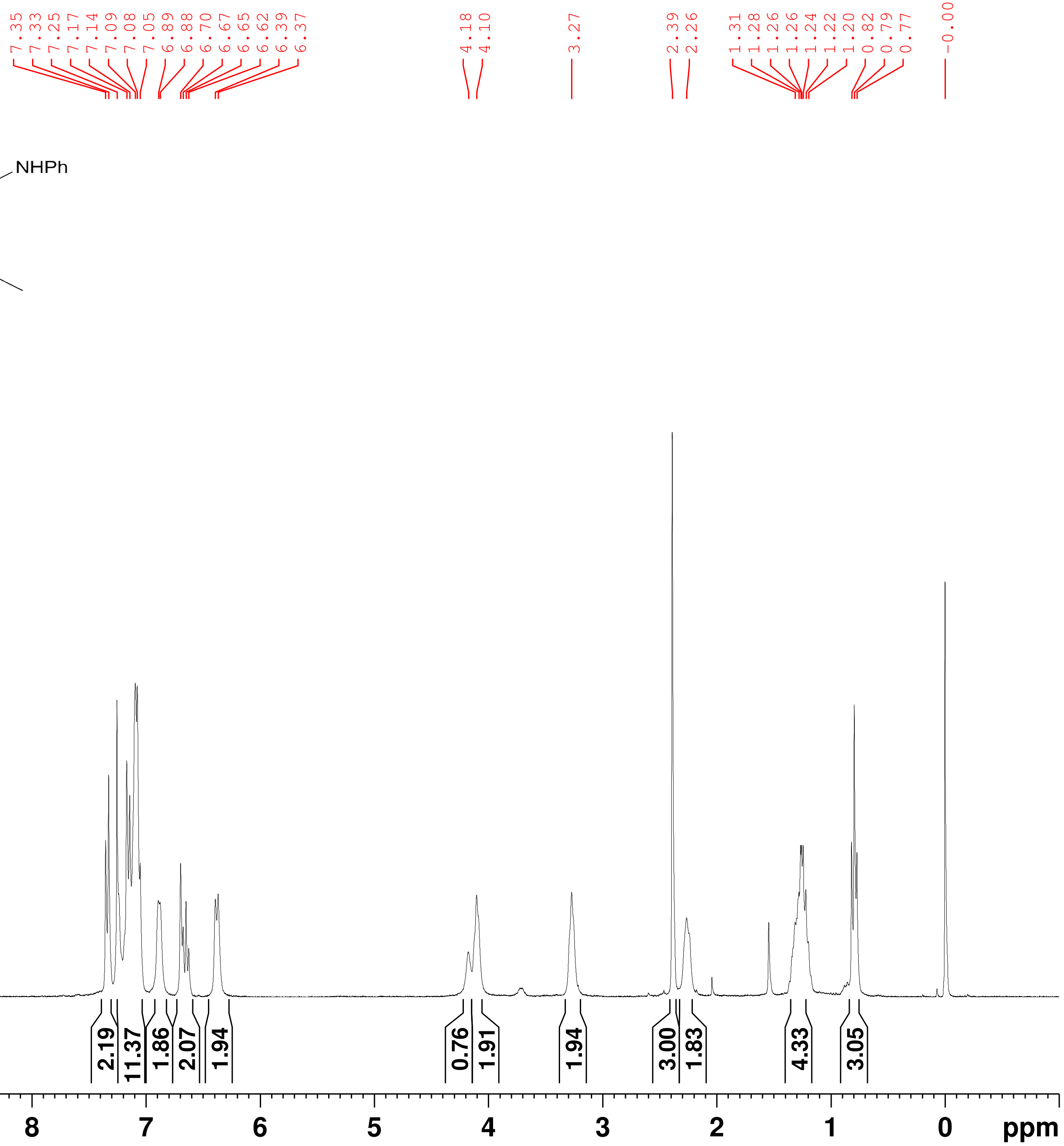

Current Data Parameters
NAME pig-St-5-137-1H
EXPNO 1
PROCNO 1

F2 - Acquisition Parameters
Date_ 20210201
Time 15.29
INSTRUM FOURIER300
PROBHD 5 mm DUL 13C-1
PULPROG zg30
TD 65536
SOLVENT CDCl3
NS 32
DS 4
SWH 6103.516 Hz
FIDRES 0.093132 Hz
AQ 5.3687091 sec
RG 66.8047
DW 81.920 usec
DE 6.50 usec
TE 300.0 K
D1 1.00000000 sec
TD0 1

======== CHANNEL f1 ========
SFO1 300.1818537 MHz
NUC1 1H
P1 15.00 usec
PLW1 13.80399990 W

F2 - Processing parameters
SI 65536
SF 300.1800071 MHz
WDW EM
SSB 0
LB 0.30 Hz
GB 0
PC 1.00

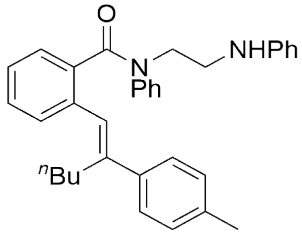

4d

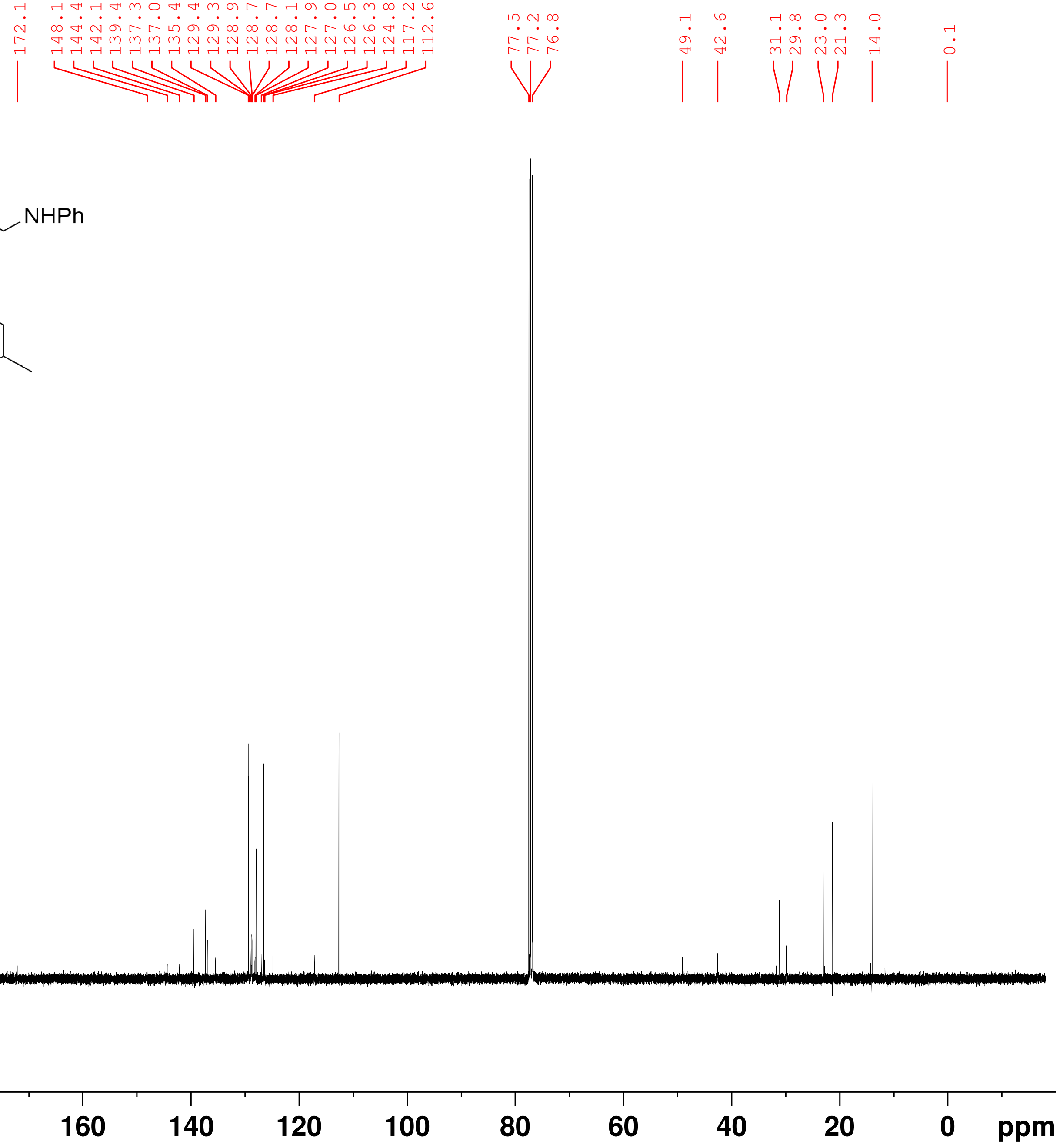

Current Data Parameters
NAME 13C_ST-5-137
EXPNO 1
PROCNO 1

F2 - Acquisition Parameters
Date_ 20210804
Time 17.24 h
INSTRUM Avance
PROBHD Z167430_0032 (
PULPROG zgpg30
TD 65536
SOLVENT CDCl3
NS 300
DS 4
SWH 23809.523 Hz
FIDRES 0.726609 Hz
AQ 1.3762560 sec
RG 3.25
DW 21.000 usec
DE 19.29 usec
TE 298.0 K
D1 2.00000000 sec
D11 0.03000000 sec
TD0 1
SFO1 100.6655806 MHz
NUC1 13C
P0 3.33 usec
P1 10.00 usec
PLW1 39.31399918 W
SFO2 400.3016012 MHz
NUC2 1H
CPDPRG[2 waltz64
PCPD2 80.00 usec
PLW2 8.80000019 W
PLW12 0.20176961 W
PLW13 0.10112690 W

F2 - Processing parameters
SI 131072
SF 100.6555021 MHz
WDW no
SSB 0
LB 0 Hz
GB 0
PC 1.40

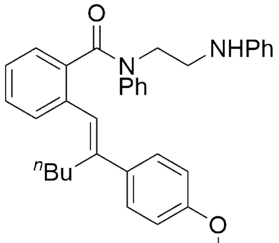

4e

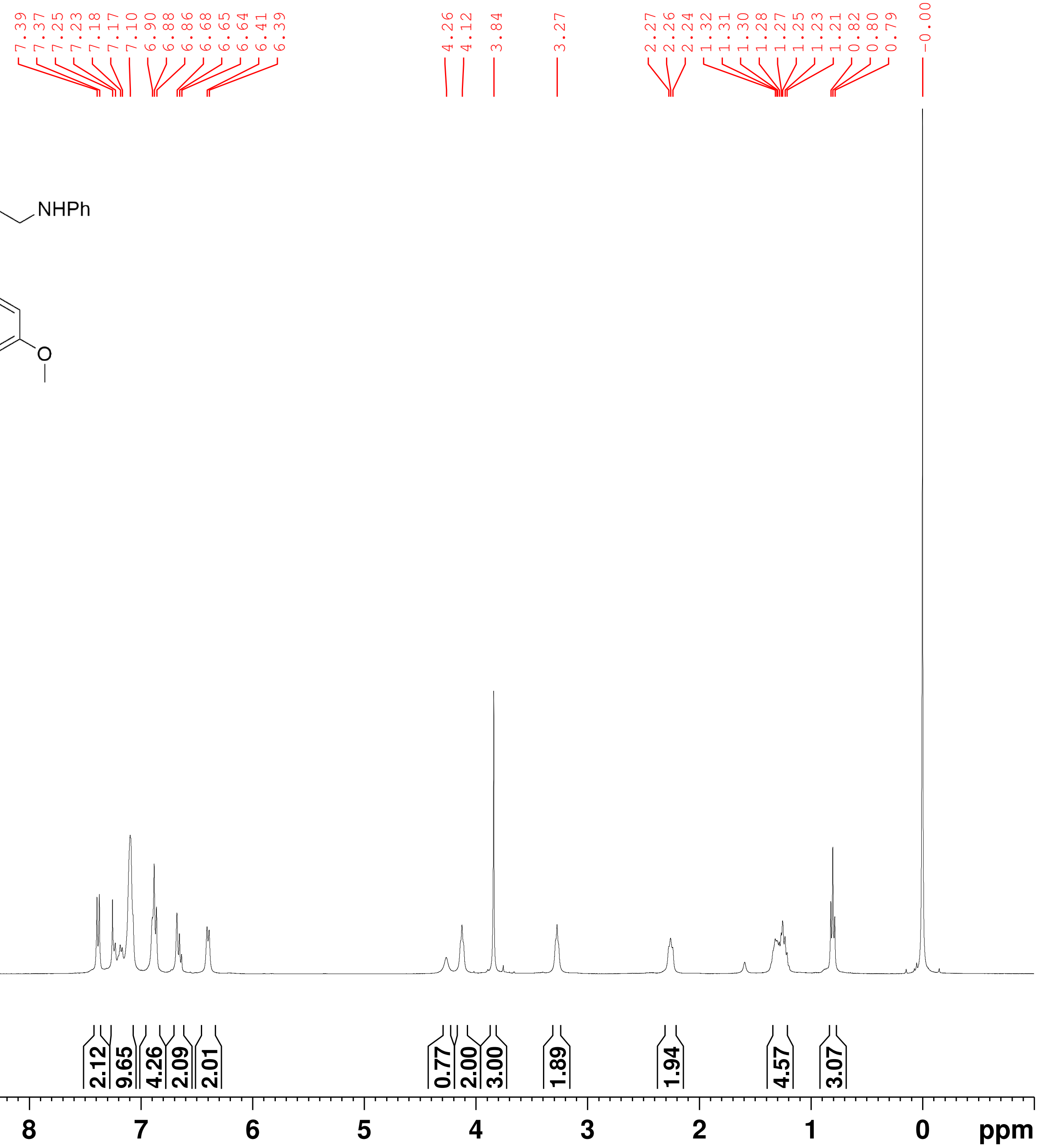


```
Current Data Parameters
NAME          1H_ST-4-171
EXPNO                   1
PROCNO                  1

F2 - Acquisition Parameters
Date_            20200311
Time                18.04
INSTRUM             spect
PROBHD   5 mm Multinucl
PULPROG              zg30
TD                  32768
SOLVENT             CDCl3
NS                     16
DS                      0
SWH              8012.820 Hz
FIDRES           0.244532 Hz
AQ              2.0447233 sec
RG                    128
DW                 62.400 usec
DE                   6.50 usec
TE                  294.2 K
D1             0.01000000 sec
TD0                     1

======== CHANNEL f1 ========
NUC1                   1H
P1                   7.20 usec
PL1                 -5.00 dB
SFO1          400.1332010 MHz

F2 - Processing parameters
SI                 131072
SF            400.1300123 MHz
WDW                    EM
SSB       0
LB                   0.25 Hz
GB        0
PC                   0.20
```

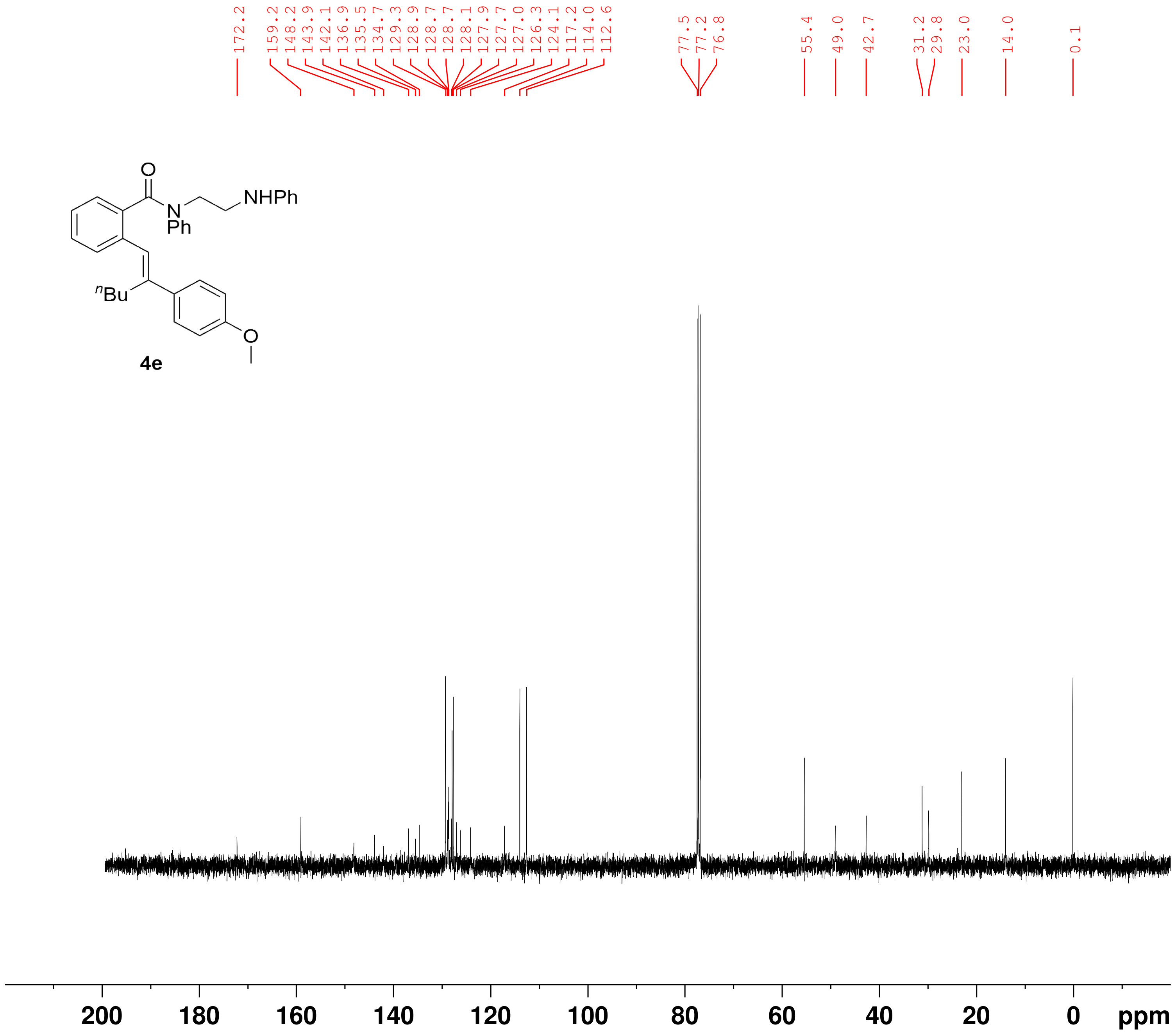


```
Current Data Parameters
NAME          13C_ST-4-171
EXPNO                    1
PROCNO                   1

F2 - Acquisition Parameters
Date_             20200311
Time                 18.20
INSTRUM              spect
PROBHD     5 mm Multinucl
PULPROG            zgdc30
TD                  65536
SOLVENT             CDCl3
NS                    300
DS                      4
SWH             26246.719 Hz
FIDRES           0.400493 Hz
AQ              1.2484608 sec
RG                  456.1
DW                 19.050 usec
DE                   6.50 usec
TE                  294.2 K
D1             0.69999999 sec
d11            0.03000000 sec
TD0                     1

======== CHANNEL f1 ========
NUC1                  13C
P1                   8.07 usec
PL1                 -6.00 dB
SFO1          100.6196894 MHz

======== CHANNEL f2 ========
CPDPRG[2          waltz16
NUC2                   1H
PCPD2               80.00 usec
PL2       0 dB
PL12                18.00 dB
SFO2          400.1318006 MHz

F2 - Processing parameters
SI                 131072
SF            100.6127576 MHz
WDW                    EM
SSB       0
LB                   0.80 Hz
GB        0
PC                   0.50
```

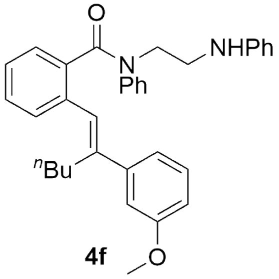

4f

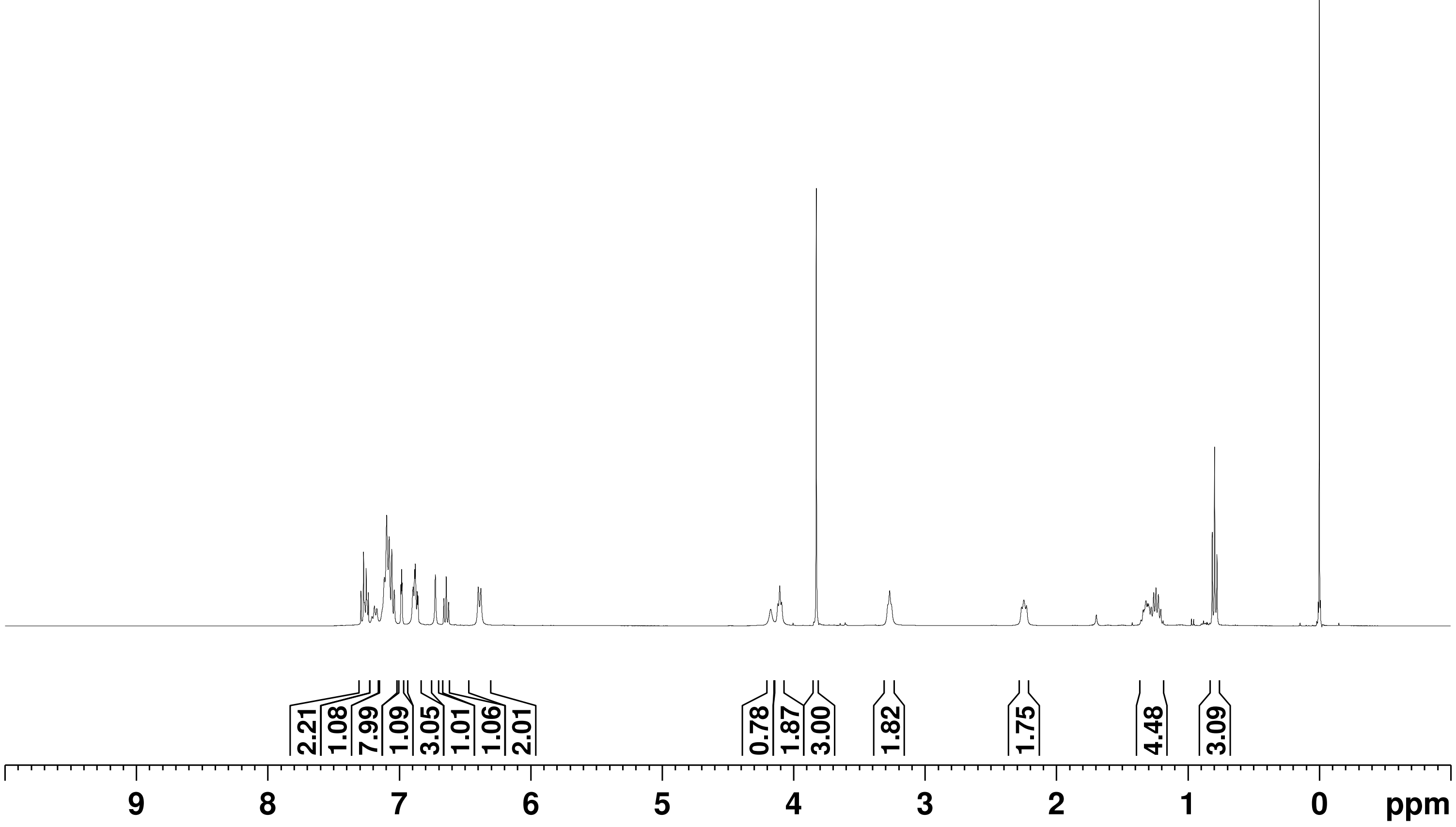

Current Data Parameters
NAME pig-st-4-195-1H
EXPNO 1
PROCNO 1

F2 - Acquisition Parameters
Date_ 20200625
Time 15.20 h
INSTRUM spect
PROBHD Z104450_0192 (
PULPROG zg30
TD 65536
SOLVENT CDCl3
NS 16
DS 2
SWH 8012.820 Hz
FIDRES 0.244532 Hz
AQ 4.0894465 sec
RG 64
DW 62.400 usec
DE 16.92 usec
TE 294.9 K
D1 1.00000000 sec
TD0 1
SFO1 400.1324708 MHz
NUC1 1H
P0 5.00 usec
P1 15.00 usec
PLW1 8.47000027 W

F2 - Processing parameters
SI 65536
SF 400.1300198 MHz
WDW EM
SSB 0
LB 0.30 Hz
GB 0
PC 1.00

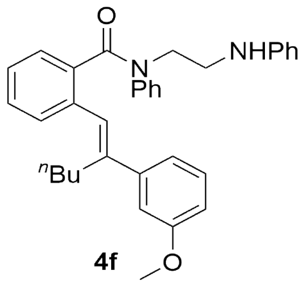

4f

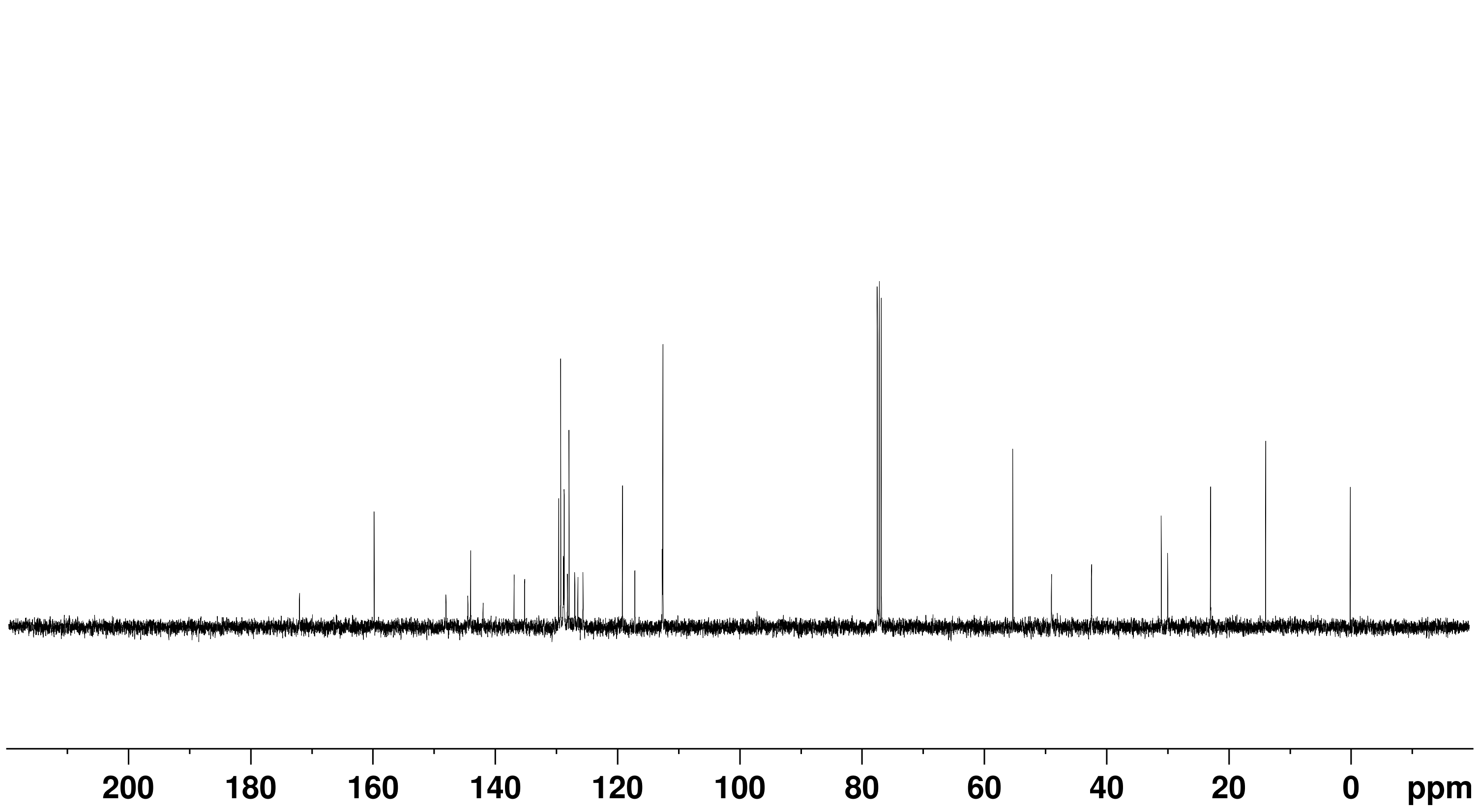

Current Data Parameters

NAME	pig-st-4-195-13C
EXPNO	1
PROCNO	1

F2 - Acquisition Parameters

Date_	20200626
Time	14.40 h
INSTRUM	spect
PROBHD	Z104450_0192 (
PULPROG	zgpg30
TD	65536
SOLVENT	CDCl3
NS	32
DS	2
SWH	24038.461 Hz
FIDRES	0.733596 Hz
AQ	1.3631488 sec
RG	144
DW	20.800 usec
DE	6.50 usec
TE	295.2 K
D1	2.00000000 sec
D11	0.03000000 sec
TD0	1
SFO1	100.6228298 MHz
NUC1	13C
P0	3.28 usec
P1	9.85 usec
PLW1	28.63999939 W
SFO2	400.1316005 MHz
NUC2	1H
CPDPRG[2	waltz65
PCPD2	90.00 usec
PLW2	8.47000027 W
PLW12	0.23528001 W
PLW13	0.11834000 W

F2 - Processing parameters

SI	32768
SF	100.6127631 MHz
WDW	EM
SSB	0
LB	1.00 Hz
GB	0
PC	1.40

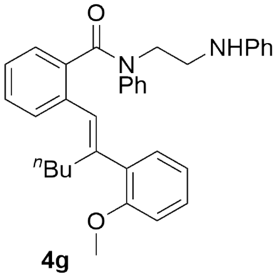

4g

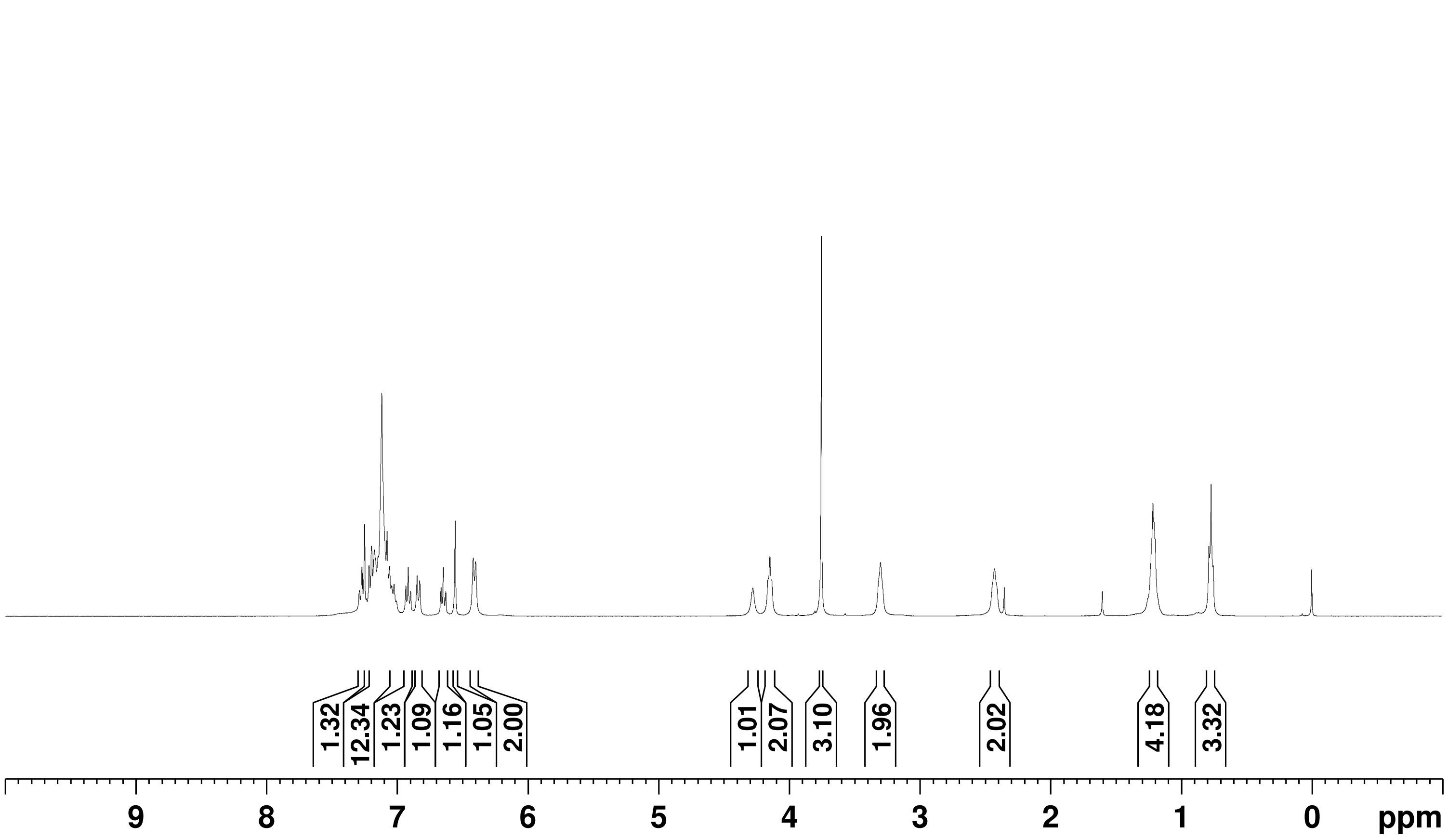


```
Current Data Parameters
NAME          1H_ST-5-147
EXPNO                   1
PROCNO                  1

F2 - Acquisition Parameters
Date_            20210301
Time                11.57
INSTRUM             spect
PROBHD   5 mm Multinucl
PULPROG              zg30
TD                  32768
SOLVENT             CDCl3
NS                     16
DS                      0
SWH              8012.820 Hz
FIDRES           0.244532 Hz
AQ              2.0447233 sec
RG                    128
DW                 62.400 usec
DE                   6.50 usec
TE                  295.2 K
D1             0.01000000 sec
TD0                     1

======== CHANNEL f1 ========
NUC1                   1H
P1                   7.20 usec
PL1                 -5.00 dB
SFO1          400.1332010 MHz

F2 - Processing parameters
SI                 131072
SF            400.1300134 MHz
WDW                    EM
SSB      0
LB                   0.25 Hz
GB       0
PC                   0.20
```

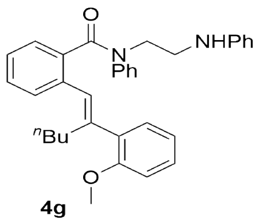

4g

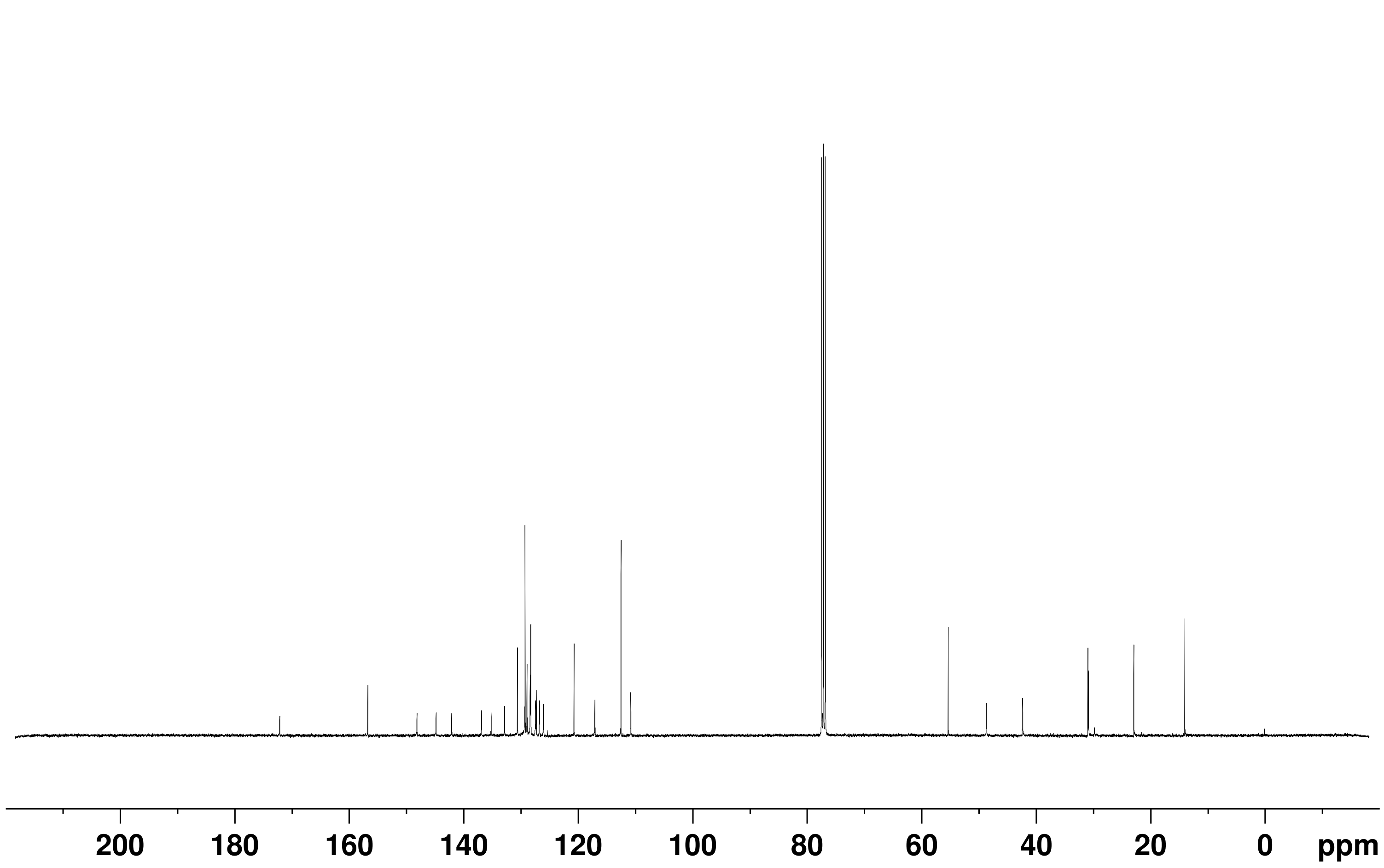

BRUKER

Current Data Parameters

NAME	13C_ST-5-147
EXPNO	1
PROCNO	1

F2 - Acquisition Parameters

Date_	20220321
Time	10.38 h
INSTRUM	Avance
PROBHD	Z167430_0032 (
PULPROG	zgpg30
TD	65536
SOLVENT	CDCl3
NS	256
DS	4
SWH	23809.523 Hz
FIDRES	0.726609 Hz
AQ	1.3762560 sec
RG	3.25
DW	21.000 usec
DE	19.29 usec
TE	298.0 K
D1	3.00000000 sec
D11	0.03000000 sec
TD0	1
SFO1	100.6655806 MHz
NUC1	13C
P0	3.33 usec
P1	10.00 usec
PLW1	39.31399918 W
SFO2	400.3016012 MHz
NUC2	1H
CPDPRG[2	waltz64
PCPD2	80.00 usec
PLW2	8.80000019 W
PLW12	0.20176961 W
PLW13	0.10112690 W

F2 - Processing parameters

SI	131072
SF	100.6555151 MHz
WDW	EM
SSB	0
LB	1.00 Hz
GB	0
PC	1.40

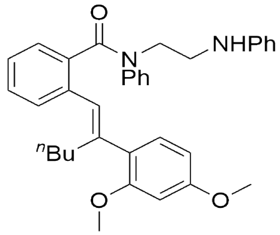

4h

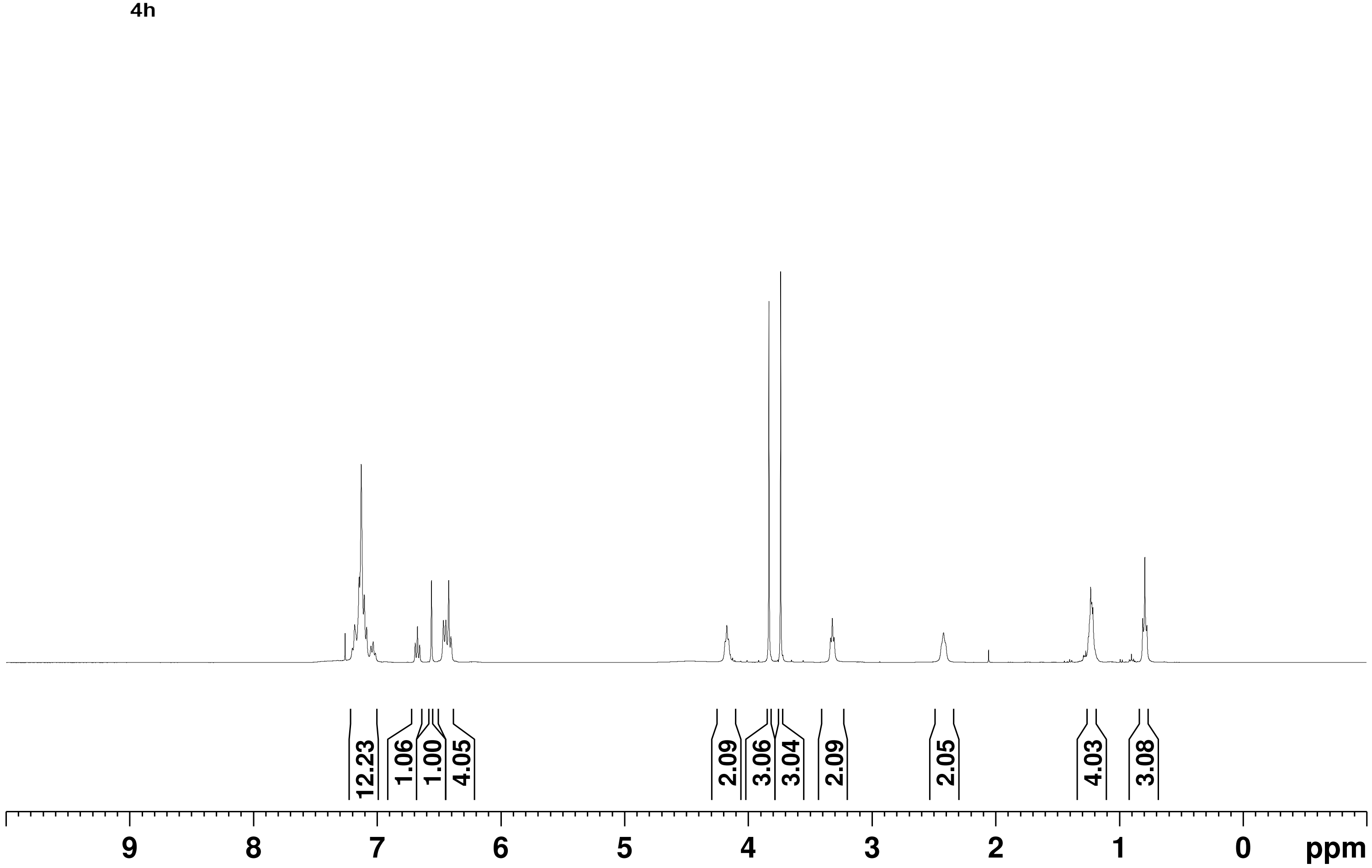

BRUKER

Current Data Parameters
NAME pig-st-4-217-1H-re
EXPNO 1
PROCNO 1

F2 - Acquisition Parameters
Date_ 20200717
Time 14.07 h
INSTRUM spect
PROBHD Z104450_0192 (
PULPROG zg30
TD 65536
SOLVENT CDCl3
NS 16
DS 2
SWH 8012.820 Hz
FIDRES 0.244532 Hz
AQ 4.0894465 sec
RG 71.8
DW 62.400 usec
DE 16.92 usec
TE 295.2 K
D1 1.00000000 sec
TD0 1
SFO1 400.1324708 MHz
NUC1 1H
P0 5.00 usec
P1 15.00 usec
PLW1 8.47000027 W

F2 - Processing parameters
SI 65536
SF 400.1300102 MHz
WDW EM
SSB 0
LB 0.30 Hz
GB 0
PC 1.00

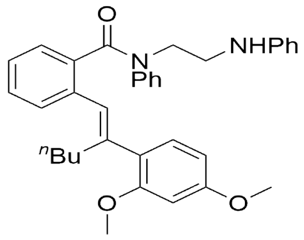

4h

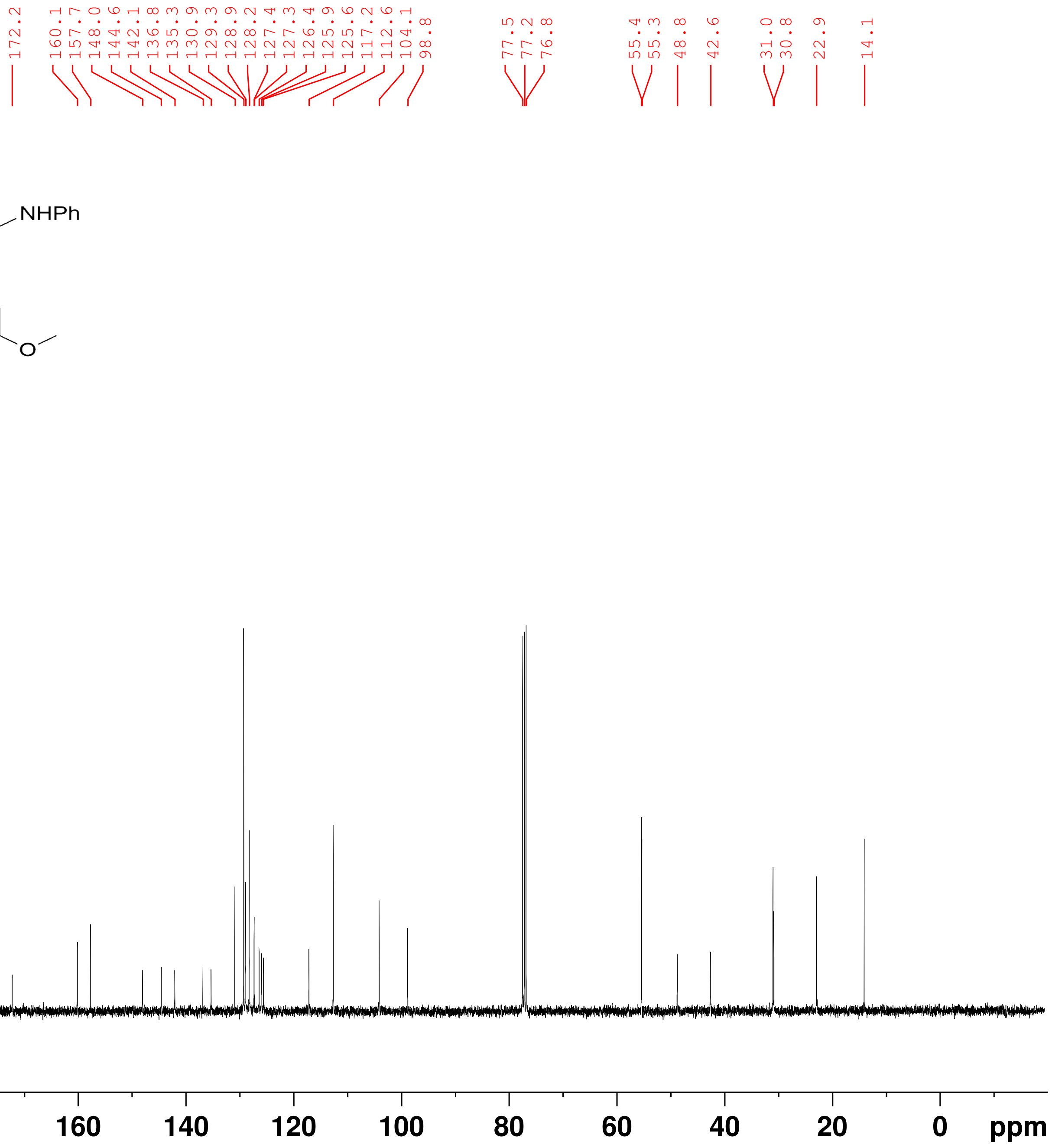


```
Current Data Parameters
NAME      pig-st-4-217-13C-re
EXPNO                       1
PROCNO                      1

F2 - Acquisition Parameters
Date_              20200717
Time                  14.19 h
INSTRUM               spect
PROBHD     Z104450_0192 (
PULPROG              zgpg30
TD                    65536
SOLVENT               CDCl3
NS                      128
DS                        2
SWH              24038.461 Hz
FIDRES            0.733596 Hz
AQ               1.3631488 sec
RG                      181
DW                   20.800 usec
DE                     6.50 usec
TE                    295.9 K
D1               2.00000000 sec
D11              0.03000000 sec
TD0                       1
SFO1            100.6228298 MHz
NUC1                    13C
P0                     3.28 usec
P1                     9.85 usec
PLW1            28.63999939 W
SFO2            400.1316005 MHz
NUC2                     1H
CPDPRG[2            waltz65
PCPD2                 90.00 usec
PLW2             8.47000027 W
PLW12            0.23528001 W
PLW13            0.11834000 W

F2 - Processing parameters
SI                    32768
SF              100.6127631 MHz
WDW                      EM
SSB        0
LB                     1.00 Hz
GB         0
PC                     1.40
```

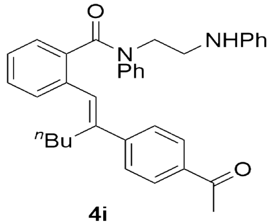

4i

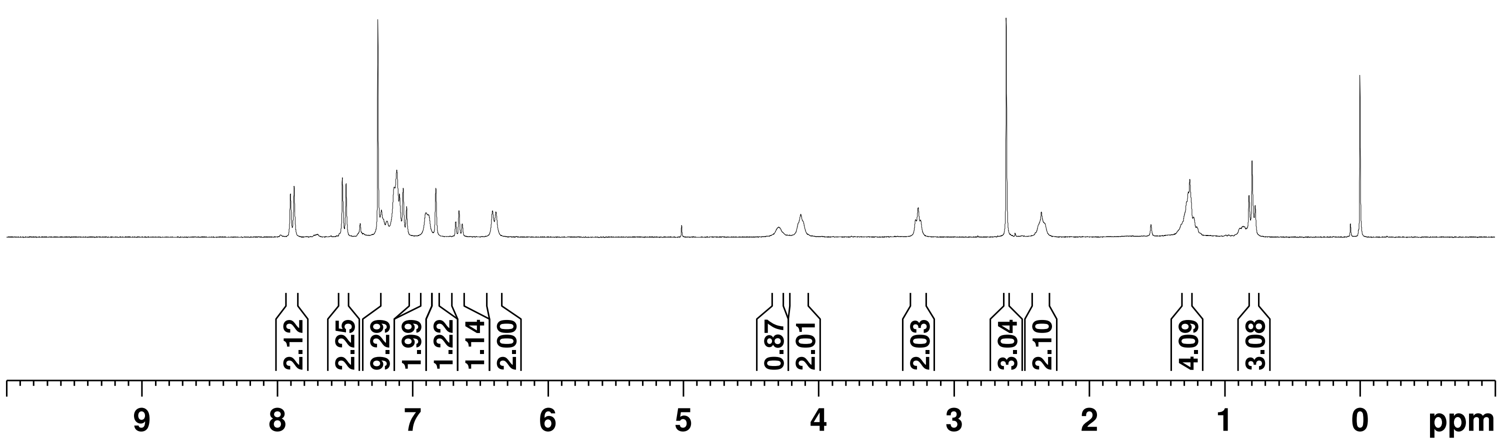

Current Data Parameters
NAME pig-St-5-139-Tol-1H
EXPNO 1
PROCNO 1

F2 - Acquisition Parameters
Date_ 20210215
Time 14.48
INSTRUM FOURIER300
PROBHD 5 mm DUL 13C-1
PULPROG zg30
TD 65536
SOLVENT CDCl3
NS 32
DS 4
SWH 6103.516 Hz
FIDRES 0.093132 Hz
AQ 5.3687091 sec
RG 95.3172
DW 81.920 usec
DE 6.50 usec
TE 300.0 K
D1 1.00000000 sec
TD0 1

======== CHANNEL f1 ========
SFO1 300.1818537 MHz
NUC1 1H
P1 15.00 usec
PLW1 13.80399990 W

F2 - Processing parameters
SI 65536
SF 300.1800062 MHz
WDW EM
SSB 0
LB 0.30 Hz
GB 0
PC 1.00

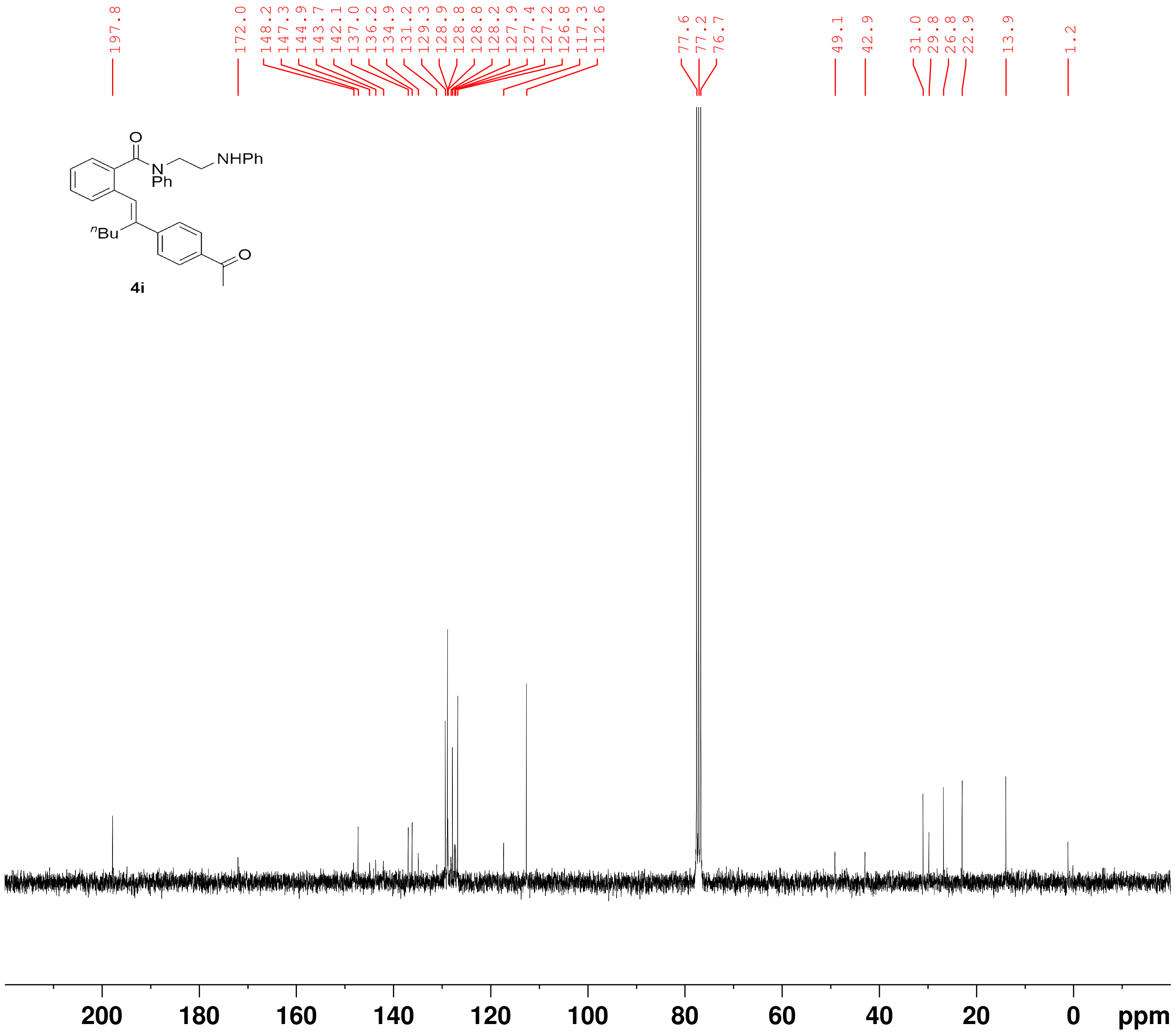

Current Data Parameters
NAME pig-St-5-139-13C
EXPNO 1
PROCNO 1

F2 - Acquisition Parameters
Date_ 20210209
Time 15.34
INSTRUM FOURIER300
PROBHD 5 mm DUL 13C-1
PULPROG zgpg30
TD 65536
SOLVENT CDCl3
NS 1024
DS 4
SWH 24414.063 Hz
FIDRES 0.372529 Hz
AQ 1.3421773 sec
RG 501.187
DW 20.480 usec
DE 6.50 usec
TE 300.1 K
D1 2.00000000 sec
D11 0.03000000 sec
D31 0.00001500 sec
D40 0.02898005 sec
L4 40
L5 57
P32 90.00 usec
TD0 1

======== CHANNEL f1 ========
SFO1 75.4878687 MHz
NUC1 13C
P1 15.00 usec
PLW1 22.00000000 W

======== CHANNEL f2 ========
SFO2 300.1812007 MHz
NUC2 1H
CPDPRG[2 waltz16
PCPD2 90.00 usec
PLW2 13.80399990 W
PLW12 0.38343999 W
PLW13 0.31059000 W

F2 - Processing parameters
SI 32768
SF 75.4803108 MHz
WDW EM
SSB 0
LB 1.00 Hz
GB 0
PC 1.40

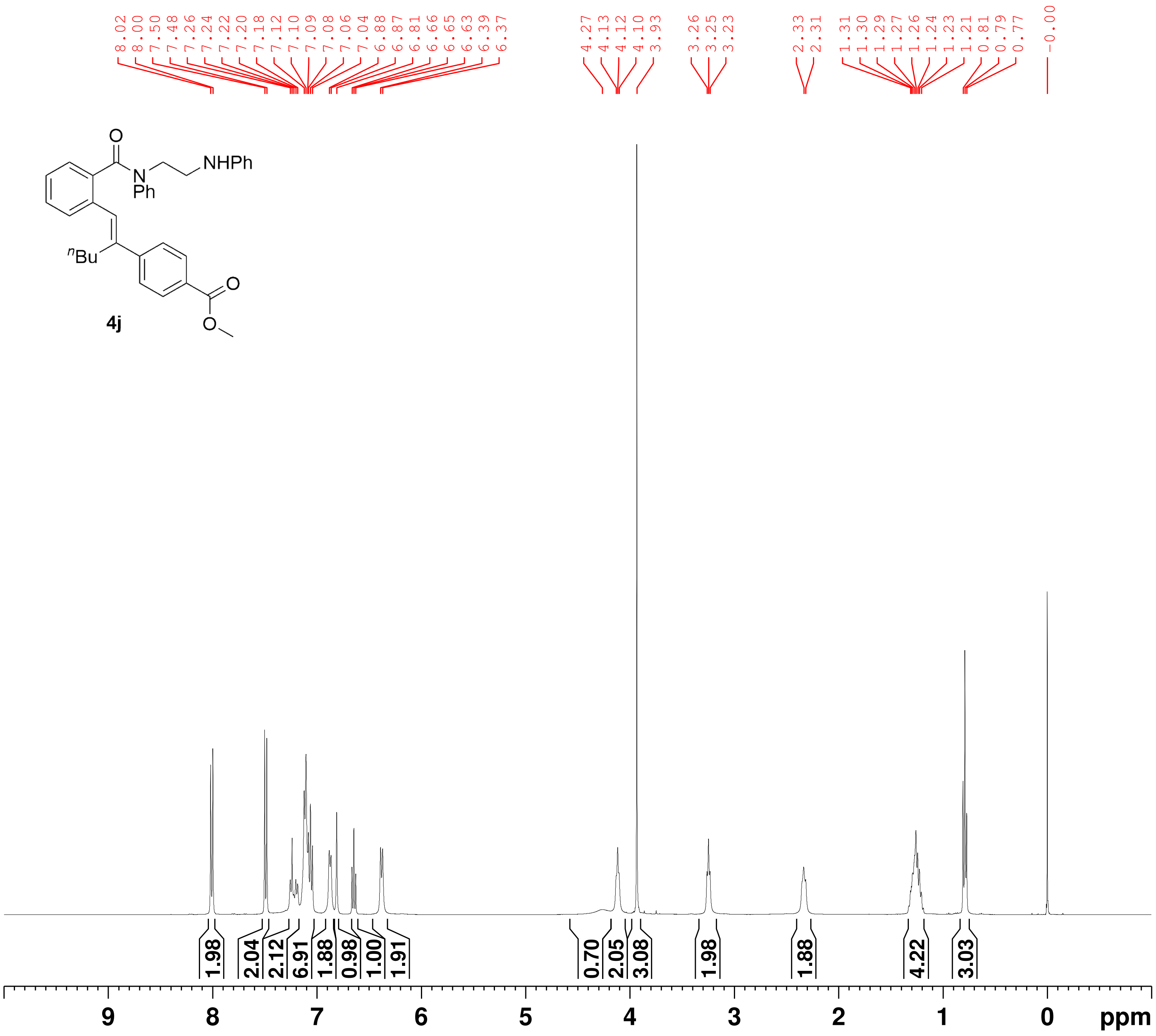

Current Data Parameters
NAME pig-st-4-237-1H-re
EXPNO 1
PROCNO 1

F2 - Acquisition Parameters
Date_ 20200814
Time 11.56 h
INSTRUM spect
PROBHD Z104450_0192 (
PULPROG zg30
TD 65536
SOLVENT CDCl3
NS 17
DS 2
SWH 8012.820 Hz
FIDRES 0.244532 Hz
AQ 4.0894465 sec
RG 32
DW 62.400 usec
DE 16.92 usec
TE 294.9 K
D1 1.00000000 sec
TD0 1
SFO1 400.1324708 MHz
NUC1 1H
P0 5.00 usec
P1 15.00 usec
PLW1 8.47000027 W

F2 - Processing parameters
SI 65536
SF 400.1300184 MHz
WDW EM
SSB 0
LB 0.30 Hz
GB 0
PC 1.00

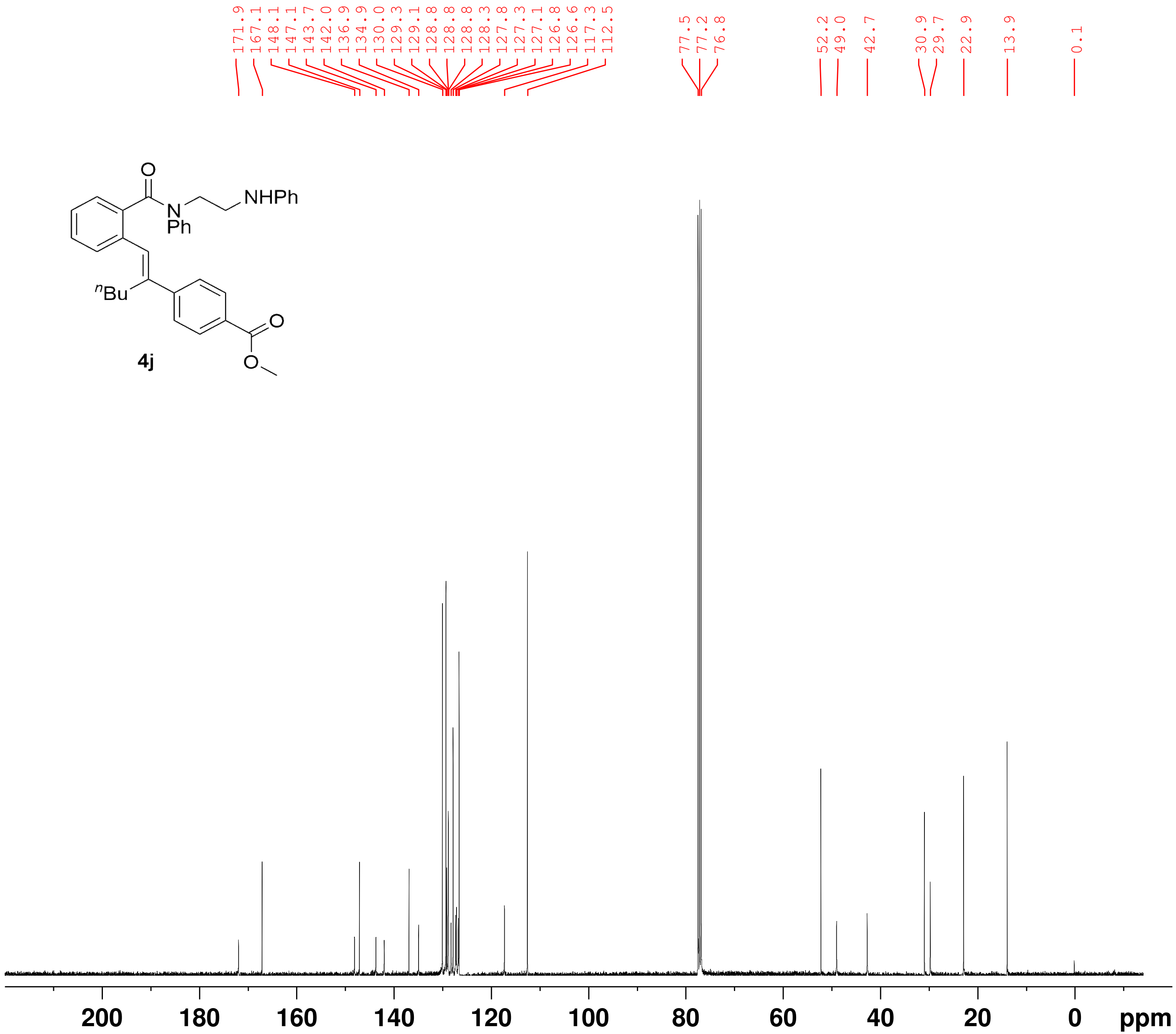

Current Data Parameters
NAME 13C_ST-4-247-recheck
EXPNO 1
PROCNO 1

F2 - Acquisition Parameters
Date_ 20220112
Time 14.59 h
INSTRUM Avance
PROBHD Z167430_0032 (
PULPROG zgpg30
TD 65536
SOLVENT CDCl3
NS 300
DS 4
SWH 25000.000 Hz
FIDRES 0.762939 Hz
AQ 1.3107200 sec
RG 3.25
DW 20.000 usec
DE 18.29 usec
TE 298.0 K
D1 2.00000000 sec
D11 0.03000000 sec
TD0 1
SFO1 100.6665872 MHz
NUC1 13C
P0 3.33 usec
P1 10.00 usec
PLW1 39.31399918 W
SFO2 400.3016012 MHz
NUC2 1H
CPDPRG[2 waltz64
PCPD2 80.00 usec
PLW2 8.80000019 W
PLW12 0.20176961 W
PLW13 0.10112690 W

F2 - Processing parameters
SI 131072
SF 100.6555053 MHz
WDW EM
SSB 0
LB 1.00 Hz
GB 0
PC 1.40

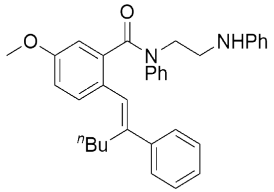

4k

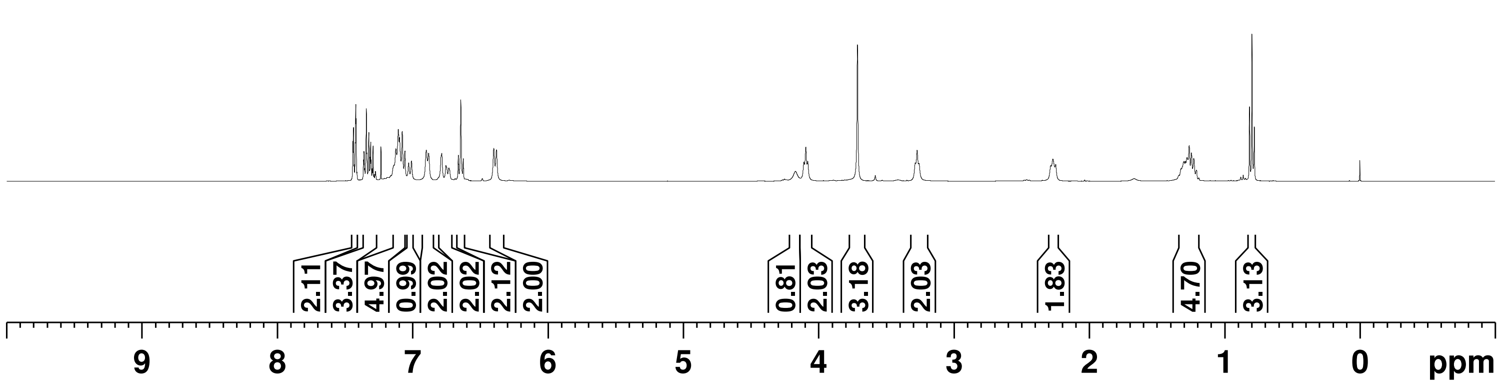

Current Data Parameters
NAME 1H_ST-6-97
EXPNO 1
PROCNO 1

F2 - Acquisition Parameters
Date_ 20210708
Time 8.49 h
INSTRUM Avance
PROBHD Z167430_0032 (
PULPROG zg30
TD 65536
SOLVENT CDCl3
NS 16
DS 2
SWH 8196.722 Hz
FIDRES 0.250144 Hz
AQ 3.9976959 sec
RG 64.4864
DW 61.000 usec
DE 13.20 usec
TE 298.0 K
D1 1.00000000 sec
TD0 1
SFO1 400.3024719 MHz
NUC1 1H
P0 4.00 usec
P1 12.00 usec
PLW1 8.80000019 W

F2 - Processing parameters
SI 65536
SF 400.3000186 MHz
WDW no
SSB 0
LB 0 Hz
GB 0
PC 1.00

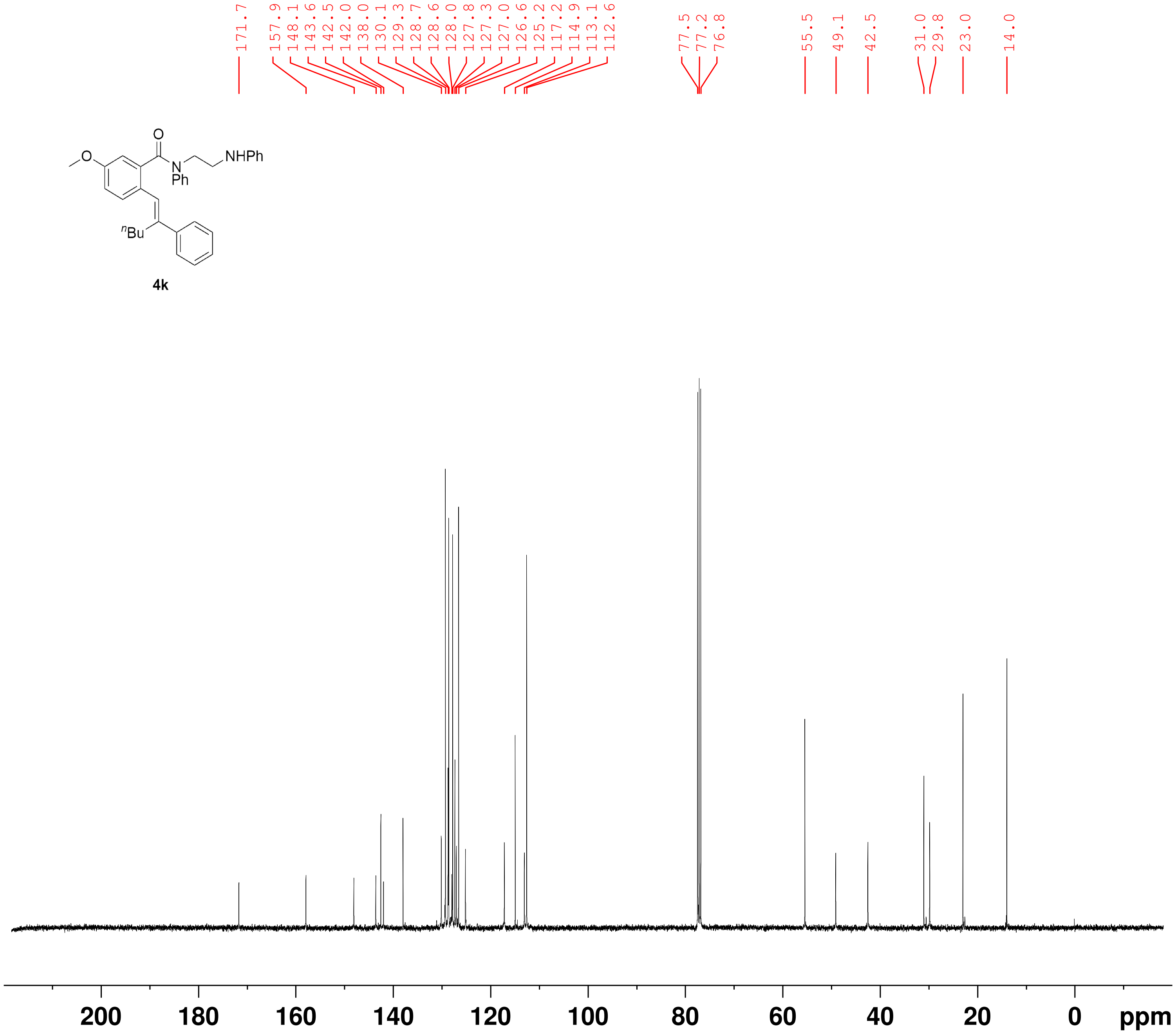


```
Current Data Parameters
NAME          13C_ST-6-97
EXPNO                   1
PROCNO                  1

F2 - Acquisition Parameters
Date_            20210707
Time                17.47 h
INSTRUM            Avance
PROBHD     Z167430_0032 (
PULPROG           zgpg30
TD                  65536
SOLVENT             CDCl3
NS                    128
DS                      4
SWH             23809.523 Hz
FIDRES           0.726609 Hz
AQ              1.3762560 sec
RG                   3.25
DW                 21.000 usec
DE                  19.29 usec
TE                  298.0 K
D1             2.00000000 sec
D11            0.03000000 sec
TD0                     1
SFO1          100.6655806 MHz
NUC1                  13C
P0                   3.33 usec
P1                  10.00 usec
PLW1          39.31399918 W
SFO2          400.3016012 MHz
NUC2                   1H
CPDPRG[2          waltz64
PCPD2               80.00 usec
PLW2           8.80000019 W
PLW12          0.20176961 W
PLW13          0.10112690 W

F2 - Processing parameters
SI                 131072
SF            100.6555082 MHz
WDW                    EM
SSB      0
LB                   1.00 Hz
GB       0
PC                   1.40
```

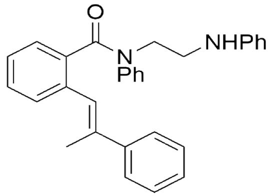

4l

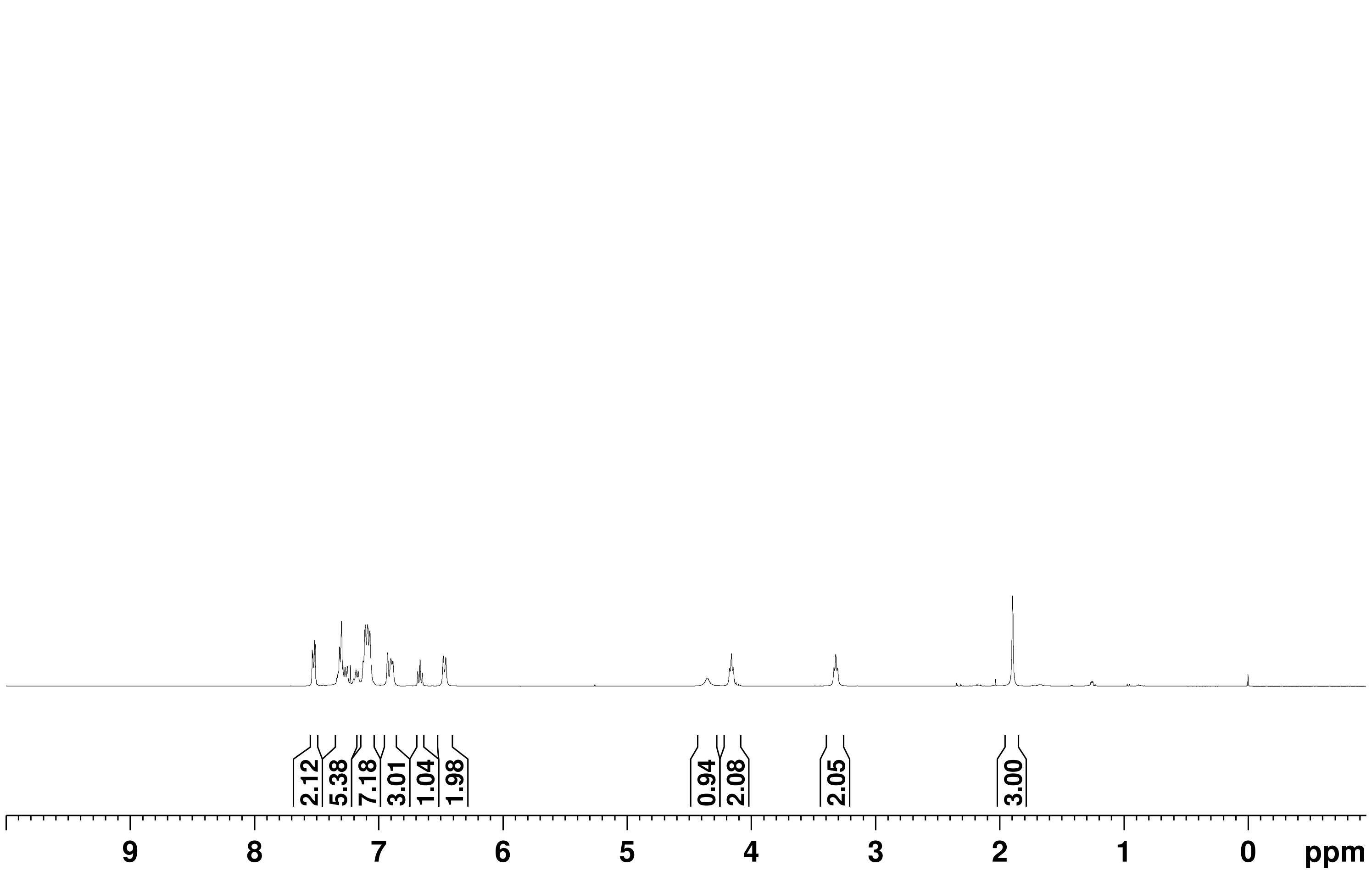

Current Data Parameters
NAME 1H ST-07-173
EXPNO 2
PROCNO 1

F2 - Acquisition Parameters
Date_ 20220429
Time 9.12 h
INSTRUM Avance
PROBHD Z167430_0032 (
PULPROG zg30
TD 65536
SOLVENT CDCl3
NS 16
DS 0
SWH 8196.722 Hz
FIDRES 0.250144 Hz
AQ 3.9976959 sec
RG 101
DW 61.000 usec
DE 13.20 usec
TE 298.0 K
D1 0.10000000 sec
TD0 1
SFO1 400.3024719 MHz
NUC1 1H
P0 4.00 usec
P1 12.00 usec
PLW1 8.80000019 W

F2 - Processing parameters
SI 65536
SF 400.3000226 MHz
WDW EM
SSB 0
LB 0.30 Hz
GB 0
PC 1.00

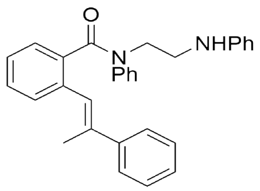

4l

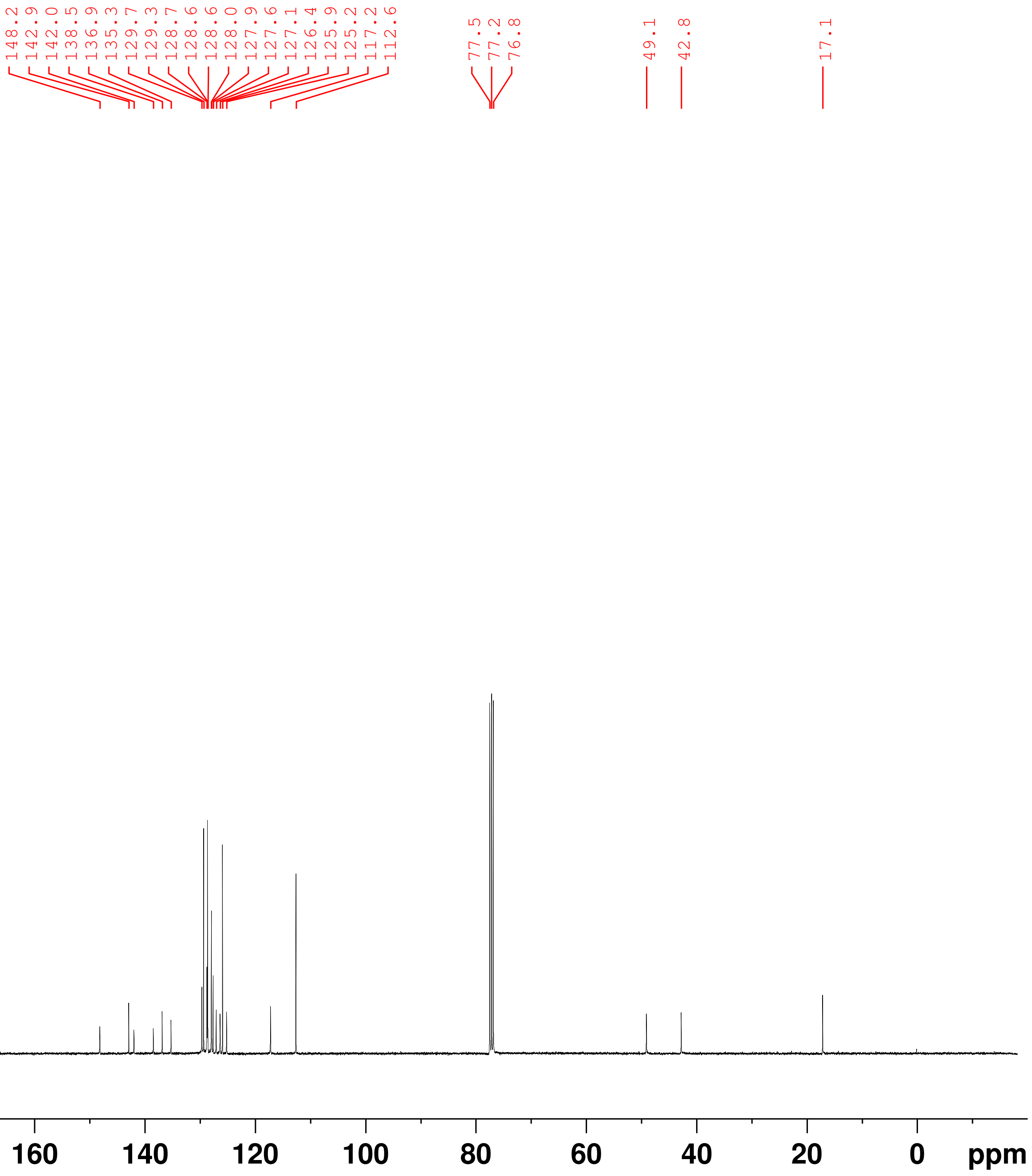

Current Data Parameters
NAME 13C_ST-7-173
EXPNO 3
PROCNO 1

F2 - Acquisition Parameters
Date_ 20220429
Time 9.25 h
INSTRUM Avance
PROBHD Z167430_0032 (
PULPROG zgpg30
TD 65536
SOLVENT CDCl3
NS 128
DS 4
SWH 23809.523 Hz
FIDRES 0.726609 Hz
AQ 1.3762560 sec
RG 3.25
DW 21.000 usec
DE 19.29 usec
TE 298.0 K
D1 3.00000000 sec
D11 0.03000000 sec
TD0 1
SFO1 100.6655806 MHz
NUC1 13C
P0 3.33 usec
P1 10.00 usec
PLW1 39.31399918 W
SFO2 400.3016012 MHz
NUC2 1H
CPDPRG[2 waltz64
PCPD2 80.00 usec
PLW2 8.80000019 W
PLW12 0.20176961 W
PLW13 0.10112690 W

F2 - Processing parameters
SI 131072
SF 100.6555100 MHz
WDW EM
SSB 0
LB 1.00 Hz
GB 0
PC 1.40

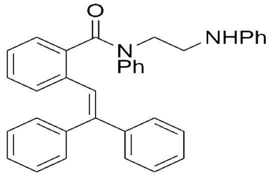

4m

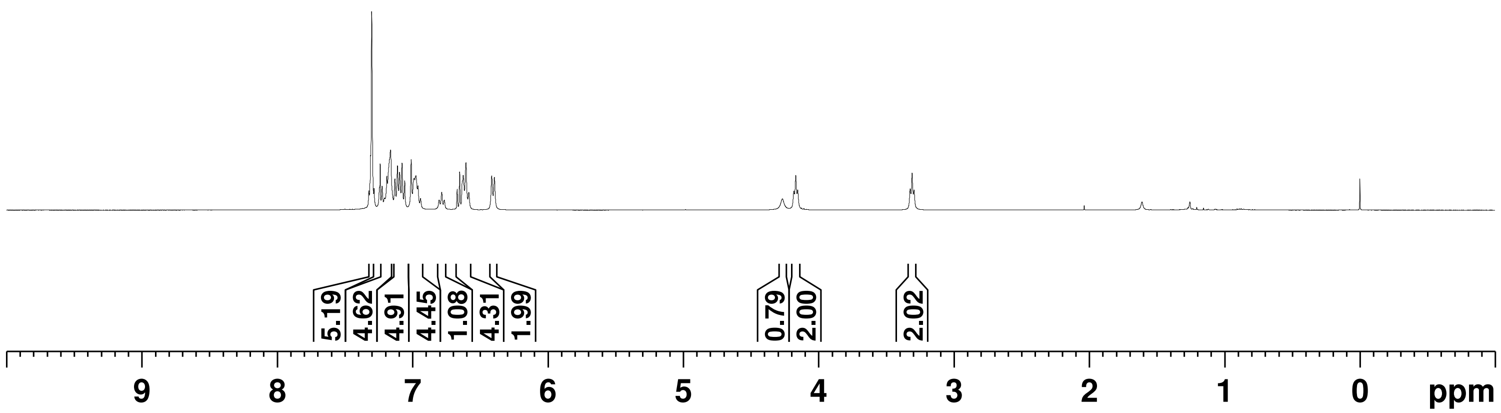


```
Current Data Parameters
NAME      1H_ST-5-269-re
EXPNO                  2
PROCNO                 1

F2 - Acquisition Parameters
Date_           20210701
Time               16.12 h
INSTRUM           Avance
PROBHD    Z167430_0032 (
PULPROG             zg30
TD                 65536
SOLVENT            CDCl3
NS                    16
DS                     2
SWH             8196.722 Hz
FIDRES          0.250144 Hz
AQ             3.9976959 sec
RG                   101
DW                61.000 usec
DE                 13.20 usec
TE                 298.0 K
D1            1.00000000 sec
TD0                    1
SFO1         400.3024719 MHz
NUC1                  1H
P0                  4.00 usec
P1                 12.00 usec
PLW1          8.80000019 W

F2 - Processing parameters
SI                 65536
SF           400.3000167 MHz
WDW                   EM
SSB       0
LB                  0.30 Hz
GB        0
PC                  1.00
```

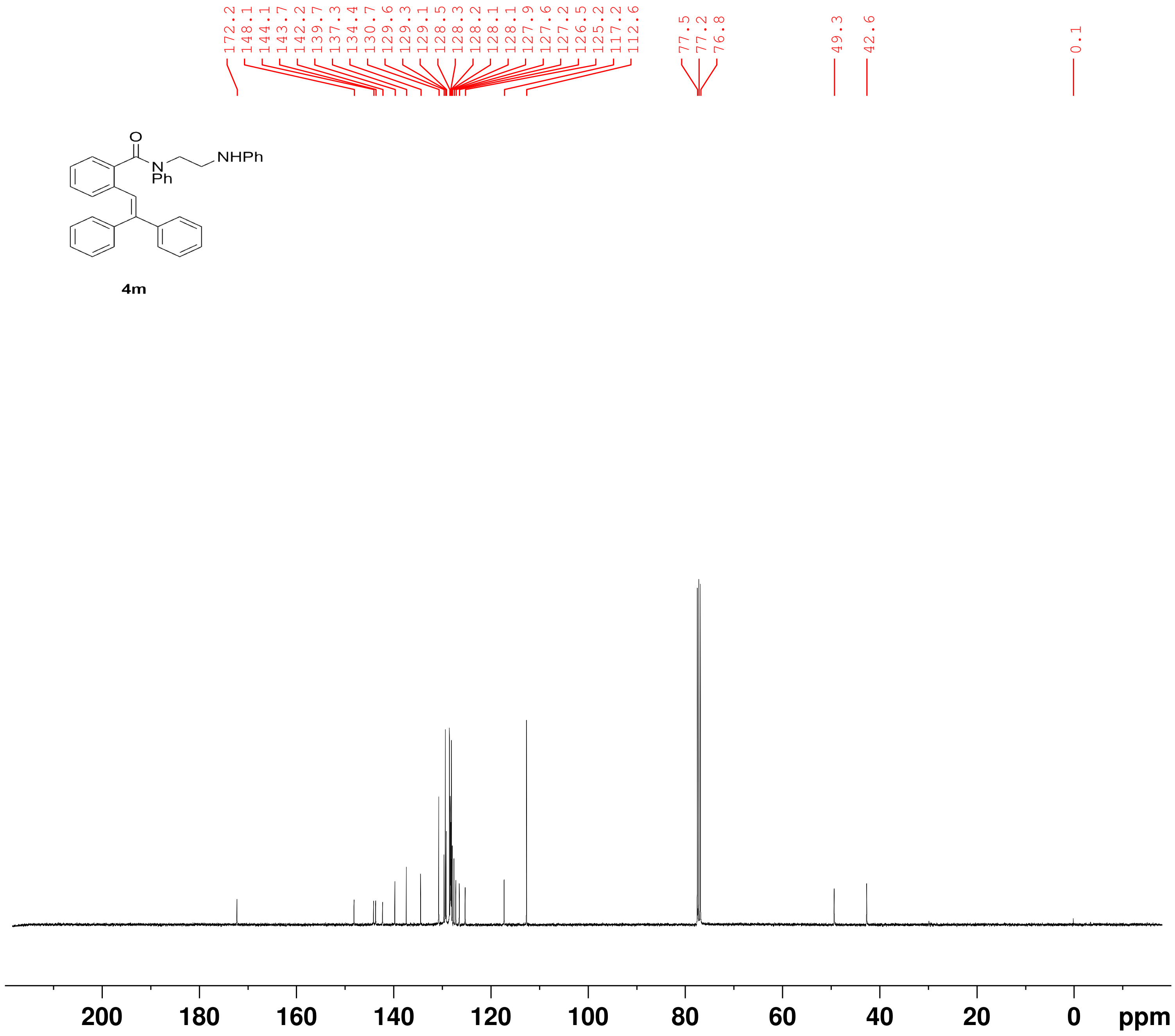

Current Data Parameters
NAME 13C_ST-5-269-re
EXPNO 1
PROCNO 1

F2 - Acquisition Parameters

Date_	20210701	
Time	16.29	h
INSTRUM	Avance	
PROBHD	Z167430_0032 (	
PULPROG	zgpg30	
TD	65536	
SOLVENT	CDCl3	
NS	200	
DS	4	
SWH	23809.523	Hz
FIDRES	0.726609	Hz
AQ	1.3762560	sec
RG	3.25	
DW	21.000	usec
DE	19.29	usec
TE	298.0	K
D1	2.00000000	sec
D11	0.03000000	sec
TD0	1	
SFO1	100.6655806	MHz
NUC1	13C	
P0	3.33	usec
P1	10.00	usec
PLW1	39.31399918	W
SFO2	400.3016012	MHz
NUC2	1H	
CPDPRG[2	waltz64	
PCPD2	80.00	usec
PLW2	8.80000019	W
PLW12	0.20176961	W
PLW13	0.10112690	W

F2 - Processing parameters

SI	131072	
SF	100.6555058	MHz
WDW	EM	
SSB	0	
LB	1.00	Hz
GB	0	
PC	1.40	

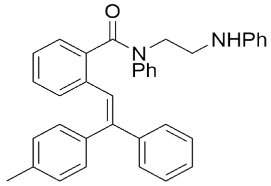

4n

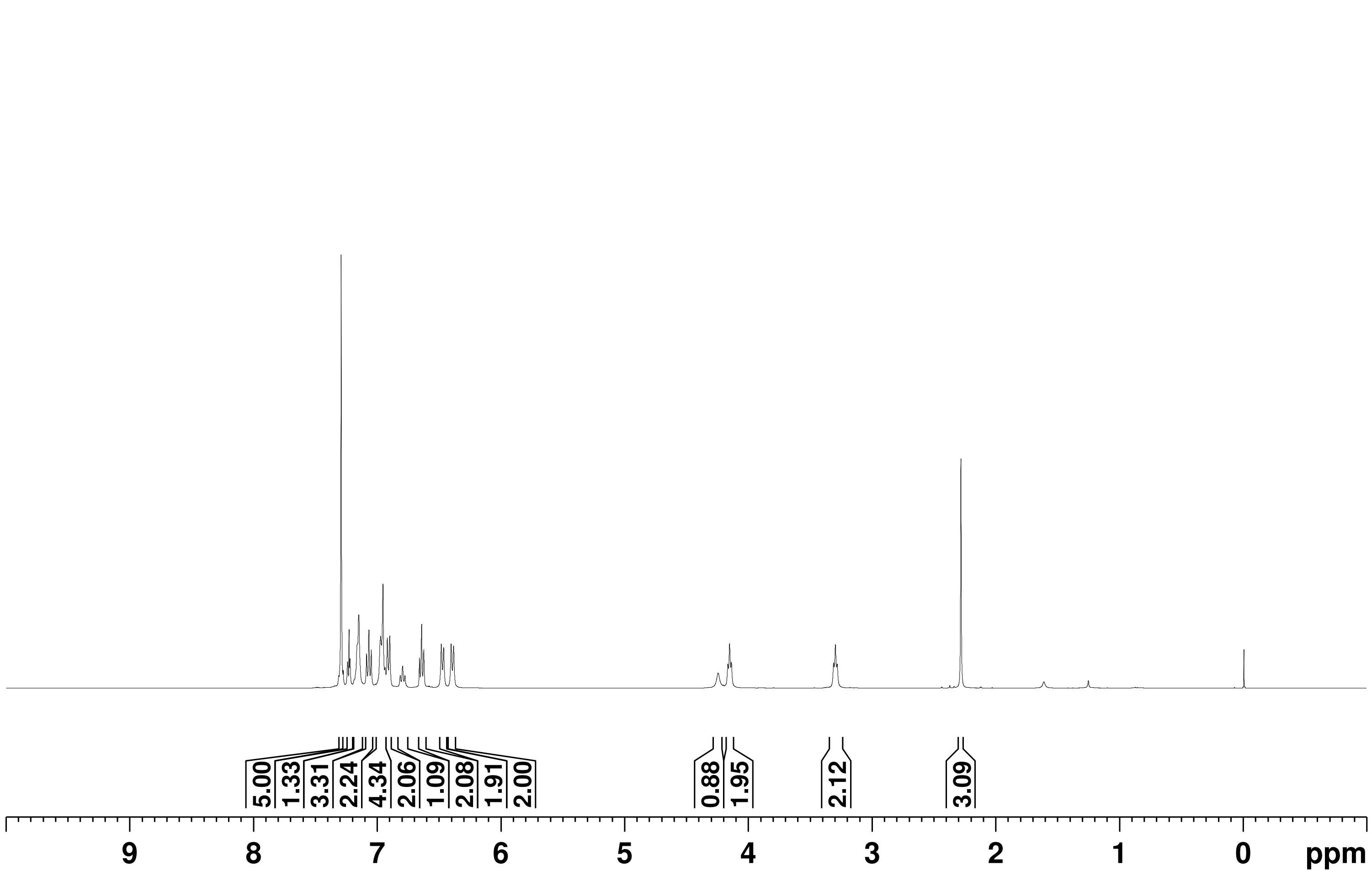

BRUKER

Current Data Parameters
NAME 1H_ST-6-45
EXPNO 1
PROCNO 1

F2 - Acquisition Parameters
Date_ 20210701
Time 19.13 h
INSTRUM Avance
PROBHD Z167430_0032 (
PULPROG zg30
TD 65536
SOLVENT CDCl3
NS 16
DS 2
SWH 8196.722 Hz
FIDRES 0.250144 Hz
AQ 3.9976959 sec
RG 101
DW 61.000 usec
DE 13.20 usec
TE 298.0 K
D1 1.00000000 sec
TD0 1
SFO1 400.3024719 MHz
NUC1 1H
P0 4.00 usec
P1 12.00 usec
PLW1 8.80000019 W

F2 - Processing parameters
SI 65536
SF 400.3000214 MHz
WDW EM
SSB 0
LB 0.30 Hz
GB 0
PC 1.00

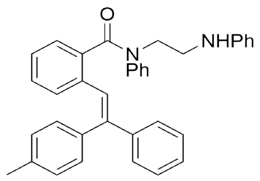

4n

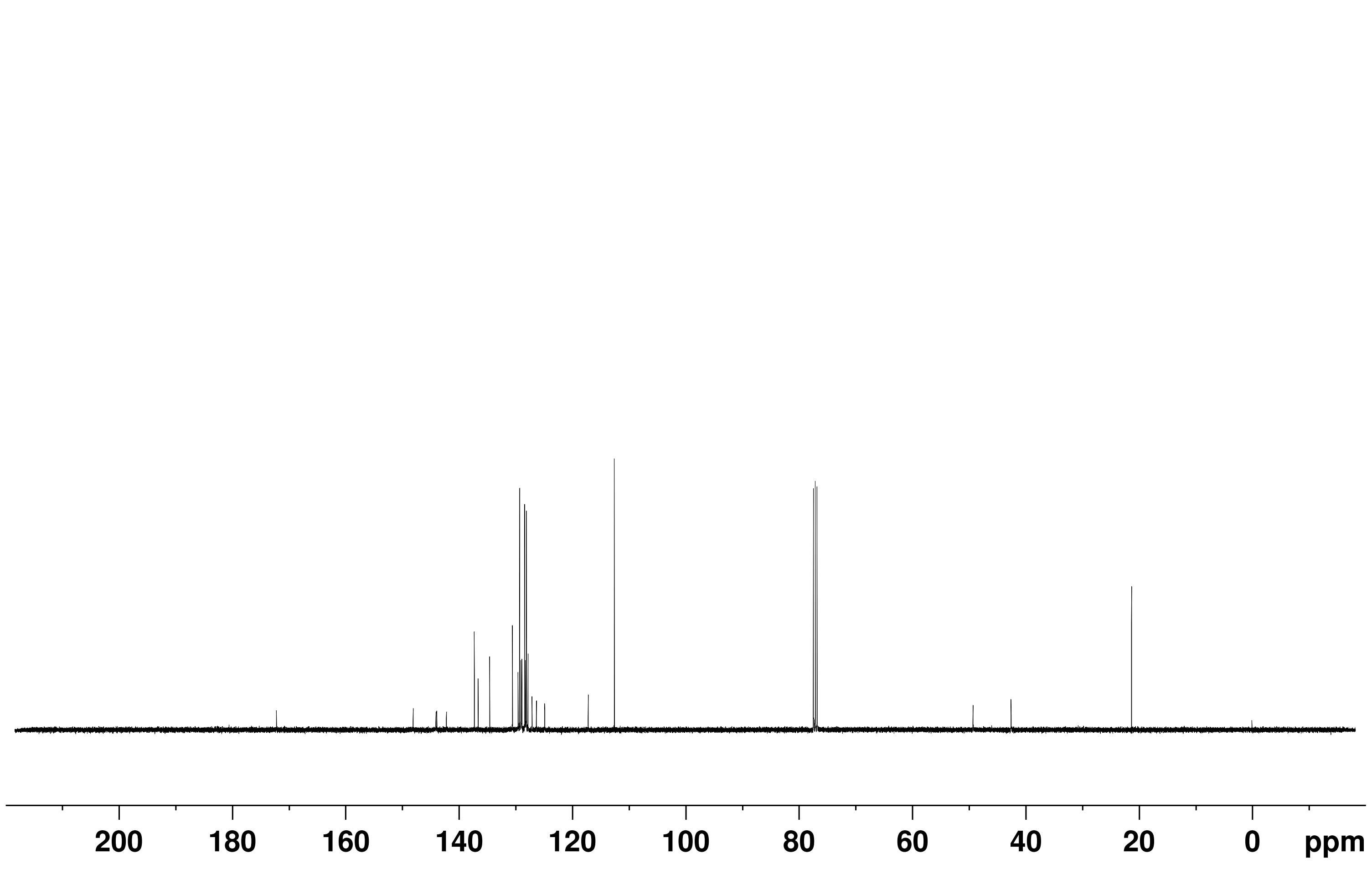

BRUKER

Current Data Parameters
NAME 13C_ST-6-45
EXPNO 1
PROCNO 1

F2 - Acquisition Parameters
Date_ 20210701
Time 19.23 h
INSTRUM Avance
PROBHD Z167430_0032 (
PULPROG zgpg30
TD 65536
SOLVENT CDCl3
NS 128
DS 4
SWH 23809.523 Hz
FIDRES 0.726609 Hz
AQ 1.3762560 sec
RG 3.25
DW 21.000 usec
DE 19.29 usec
TE 298.0 K
D1 2.00000000 sec
D11 0.03000000 sec
TD0 1
SFO1 100.6655806 MHz
NUC1 13C
P0 3.33 usec
P1 10.00 usec
PLW1 39.31399918 W
SFO2 400.3016012 MHz
NUC2 1H
CPDPRG[2 waltz64
PCPD2 80.00 usec
PLW2 8.80000019 W
PLW12 0.20176961 W
PLW13 0.10112690 W

F2 - Processing parameters
SI 131072
SF 100.6555067 MHz
WDW no
SSB 0
LB 0 Hz
GB 0
PC 1.40

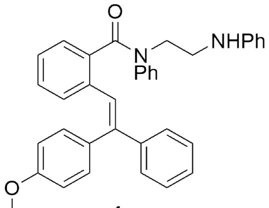

4o

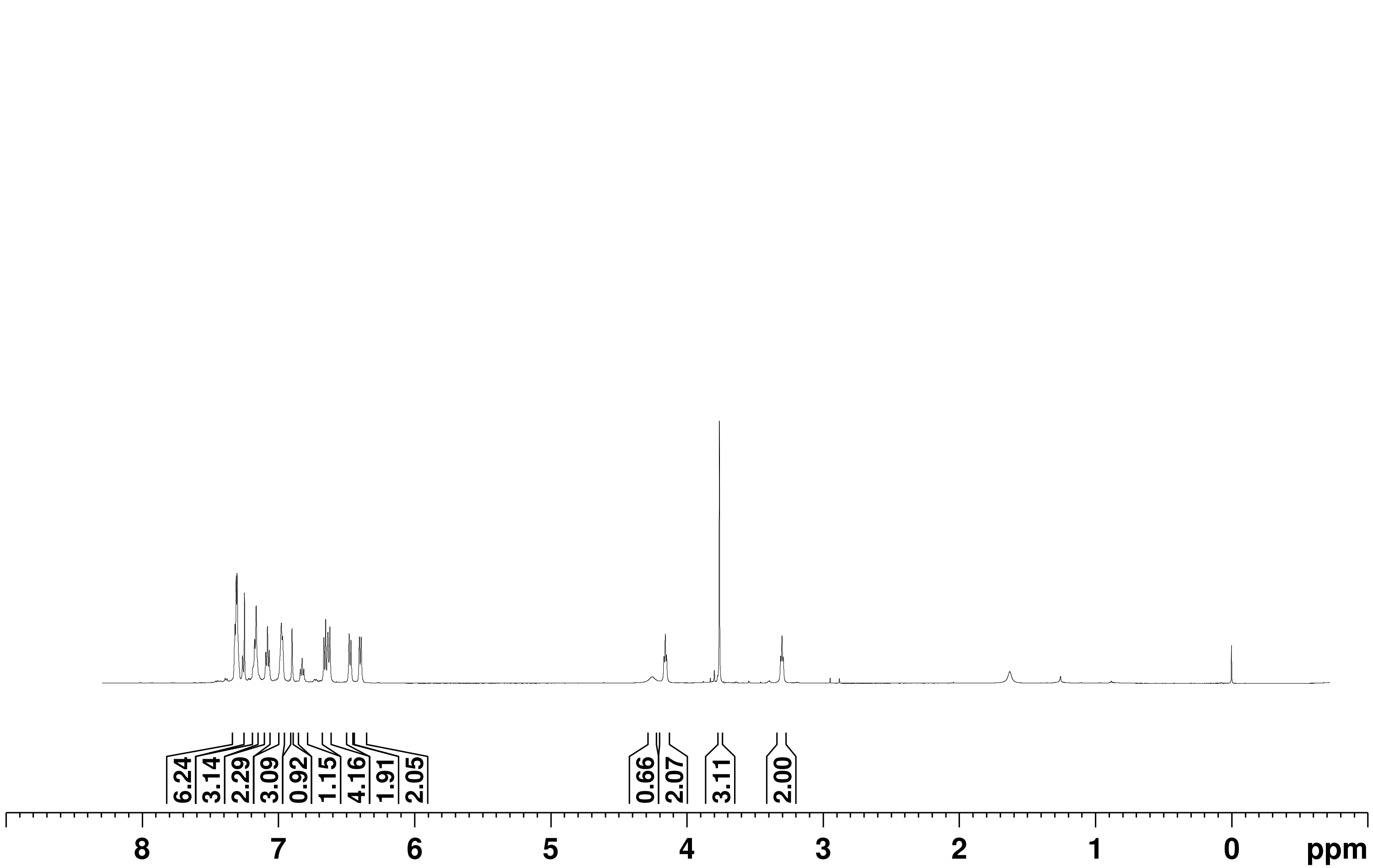

BRUKER

Current Data Parameters
NAME 1H_ST-6-7
EXPNO 1
PROCNO 1

F2 - Acquisition Parameters
Date_ 20210612
Time 20.11
INSTRUM spect
PROBHD 5 mm PABBO BB-
PULPROG zg30
TD 32768
SOLVENT CDCl3
NS 16
DS 4
SWH 5411.255 Hz
FIDRES 0.165138 Hz
AQ 3.0277631 sec
RG 114
DW 92.400 usec
DE 6.50 usec
TE 298.0 K
D1 1.00000000 sec

======== CHANNEL f1 ========
NUC1 1H
P1 12.00 usec
PLW1 26.91500092 W
SFO1 600.2122931 MHz

F2 - Processing parameters
SI 65536
SF 600.2100206 MHz
WDW EM
SSB 0
LB 0.30 Hz
GB 0
PC 1.00

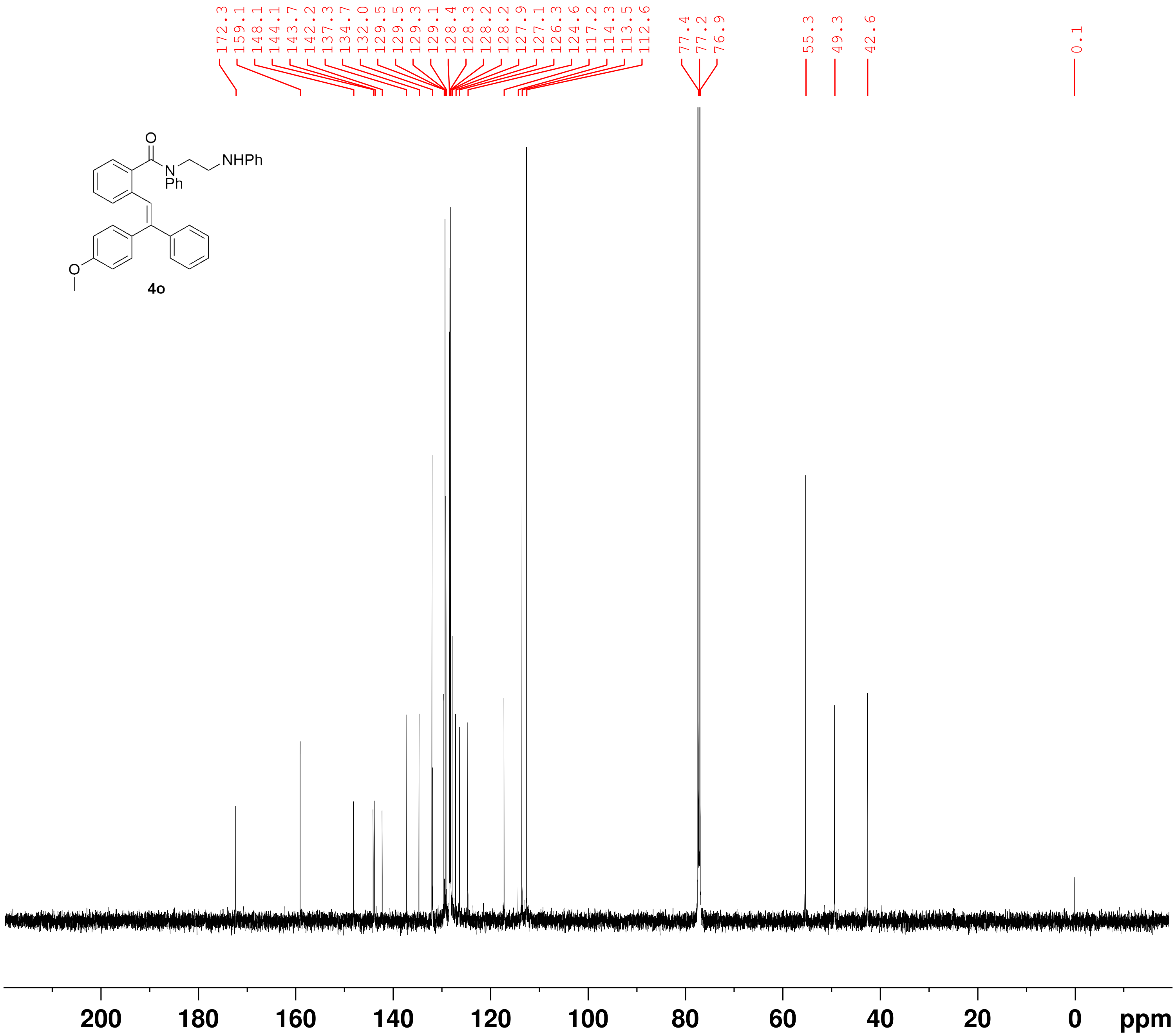


```
Current Data Parameters
NAME             13C_ST-6-7
EXPNO                    10
PROCNO                    1

F2 - Acquisition Parameters
Date_              20210612
Time                  20.52
INSTRUM               spect
PROBHD     5 mm PABBO BB-
PULPROG             zgpg30
TD                    65536
SOLVENT               CDCl3
NS                     1024
DS                        4
SWH              36057.691 Hz
FIDRES            0.550197 Hz
AQ               0.9087659 sec
RG                     2050
DW                   13.867 usec
DE                     6.50 usec
TE                    298.0 K
D1               2.00000000 sec
D11              0.03000000 sec

======== CHANNEL f1 ========
NUC1                    13C
P1                    10.00 usec
PLW1            60.25600052 W
SFO1            150.9380173 MHz

======== CHANNEL f2 ========
CPDPRG[2            waltz16
NUC2                     1H
PCPD2                 70.00 usec
PLW2            26.91500092 W
PLW12            0.79097998 W
PLW13            0.38758001 W
SFO2            600.2124008 MHz

F2 - Processing parameters
SI                    65536
SF              150.9229109 MHz
WDW                      EM
SSB      0
LB                     1.00 Hz
GB       0
PC                     1.40
```

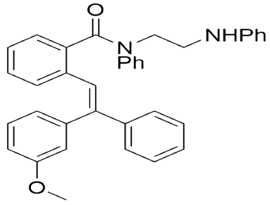

4p

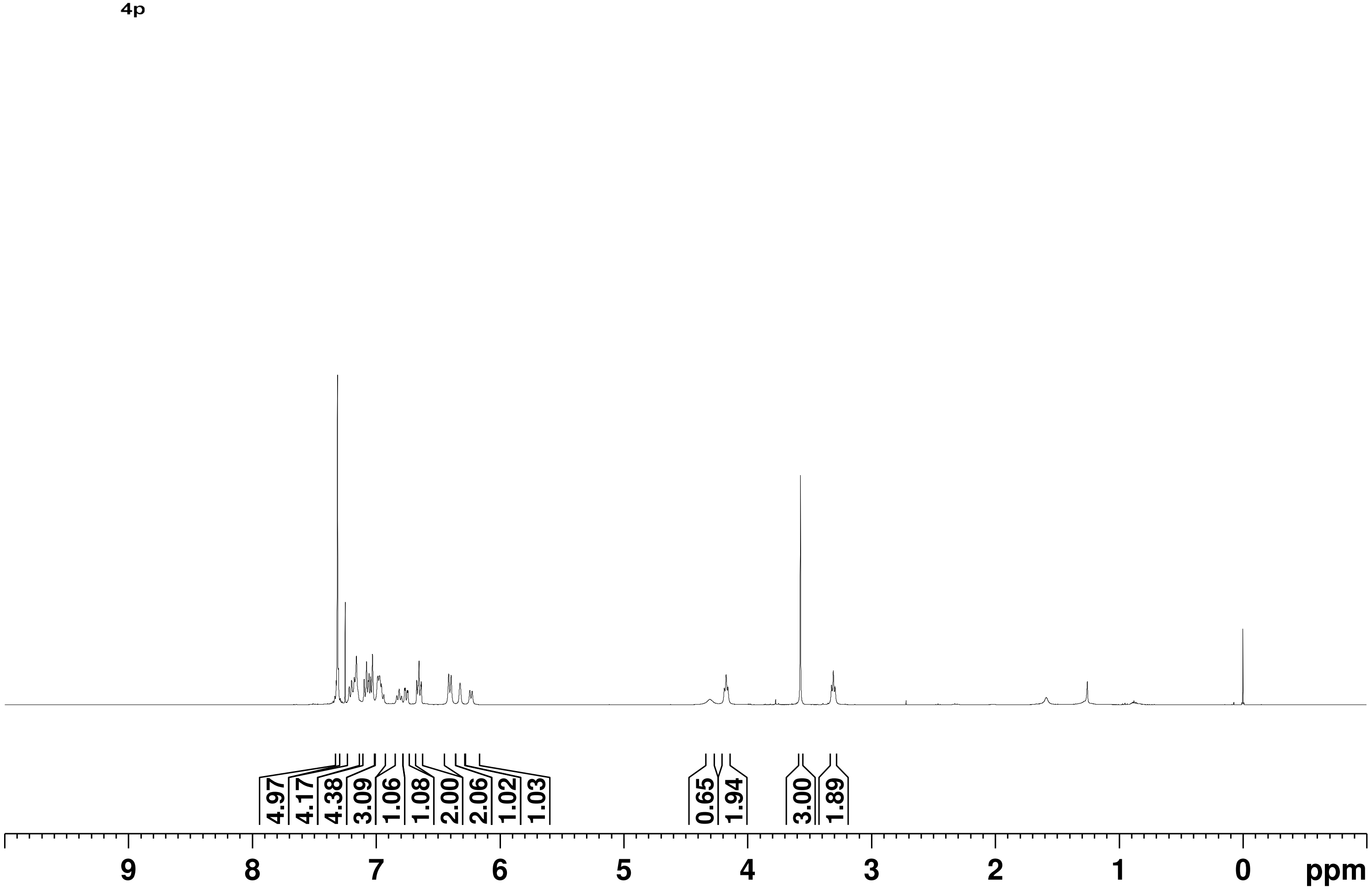


```
Current Data Parameters
NAME       1H_ST-5-273-re
EXPNO                   1
PROCNO                  1

F2 - Acquisition Parameters
Date_            20210701
Time                19.28 h
INSTRUM            Avance
PROBHD     Z167430_0032 (
PULPROG              zg30
TD                  65536
SOLVENT             CDCl3
NS                     16
DS                      2
SWH              8196.722 Hz
FIDRES           0.250144 Hz
AQ              3.9976959 sec
RG                    101
DW                 61.000 usec
DE                  13.20 usec
TE                  298.0 K
D1             1.00000000 sec
TD0                     1
SFO1          400.3024719 MHz
NUC1                   1H
P0                   4.00 usec
P1                  12.00 usec
PLW1           8.80000019 W

F2 - Processing parameters
SI                  65536
SF            400.3000124 MHz
WDW                    no
SSB       0
LB        0 Hz
GB        0
PC                   1.00
```

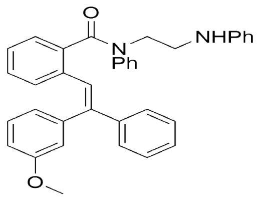

4p

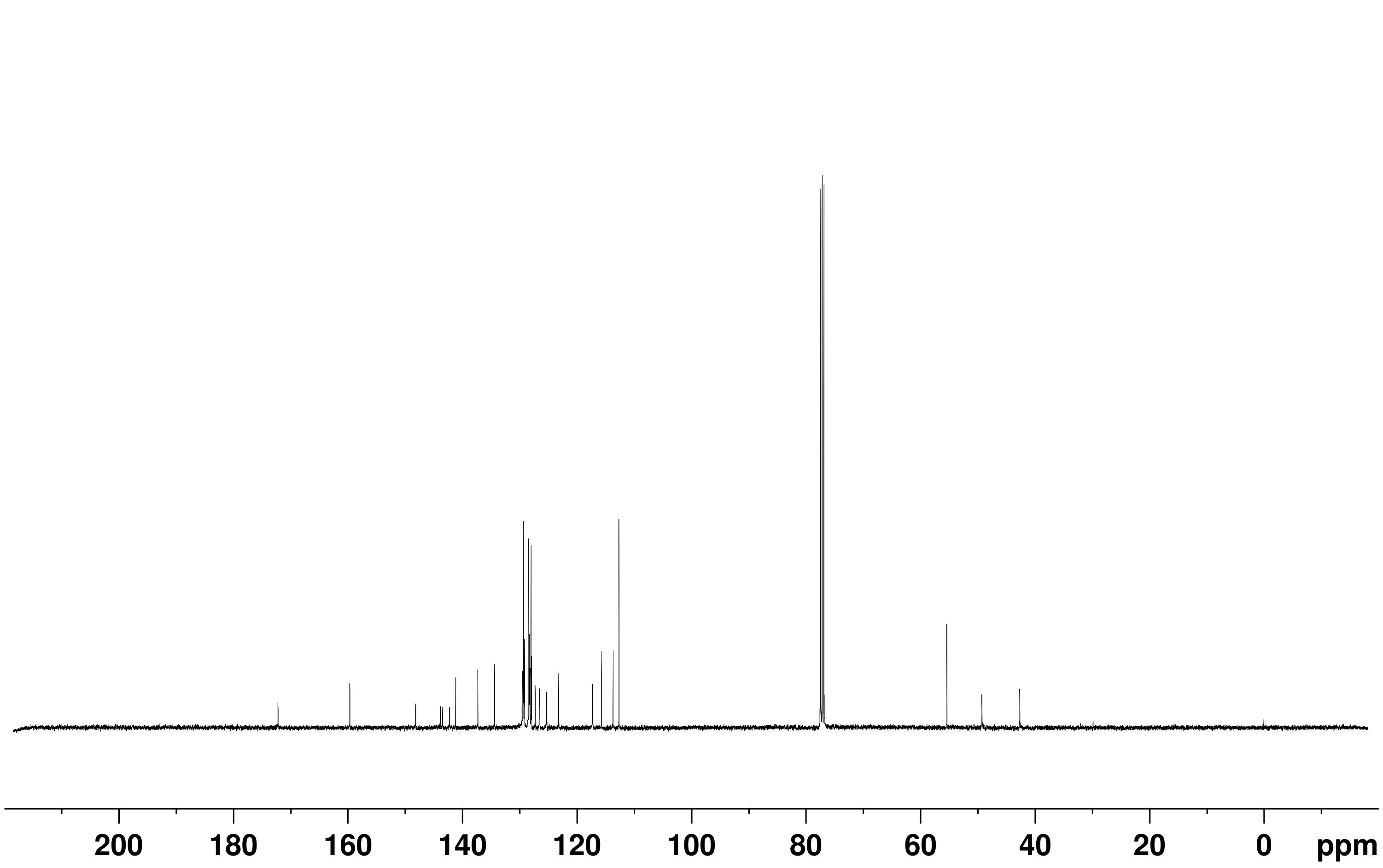

BRUKER

Current Data Parameters
NAME 13C_ST-5-273-re
EXPNO 1
PROCNO 1

F2 - Acquisition Parameters
Date_ 20210701
Time 16.57 h
INSTRUM Avance
PROBHD Z167430_0032 (
PULPROG zgpg30
TD 65536
SOLVENT CDCl3
NS 201
DS 4
SWH 23809.523 Hz
FIDRES 0.726609 Hz
AQ 1.3762560 sec
RG 3.25
DW 21.000 usec
DE 19.29 usec
TE 298.0 K
D1 2.00000000 sec
D11 0.03000000 sec
TD0 1
SFO1 100.6655806 MHz
NUC1 13C
P0 3.33 usec
P1 10.00 usec
PLW1 39.31399918 W
SFO2 400.3016012 MHz
NUC2 1H
CPDPRG[2 waltz64
PCPD2 80.00 usec
PLW2 8.80000019 W
PLW12 0.20176961 W
PLW13 0.10112690 W

F2 - Processing parameters
SI 131072
SF 100.6555038 MHz
WDW EM
SSB 0
LB 1.00 Hz
GB 0
PC 1.40

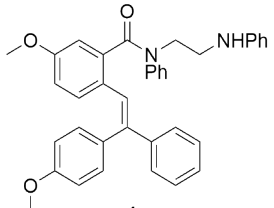

4q

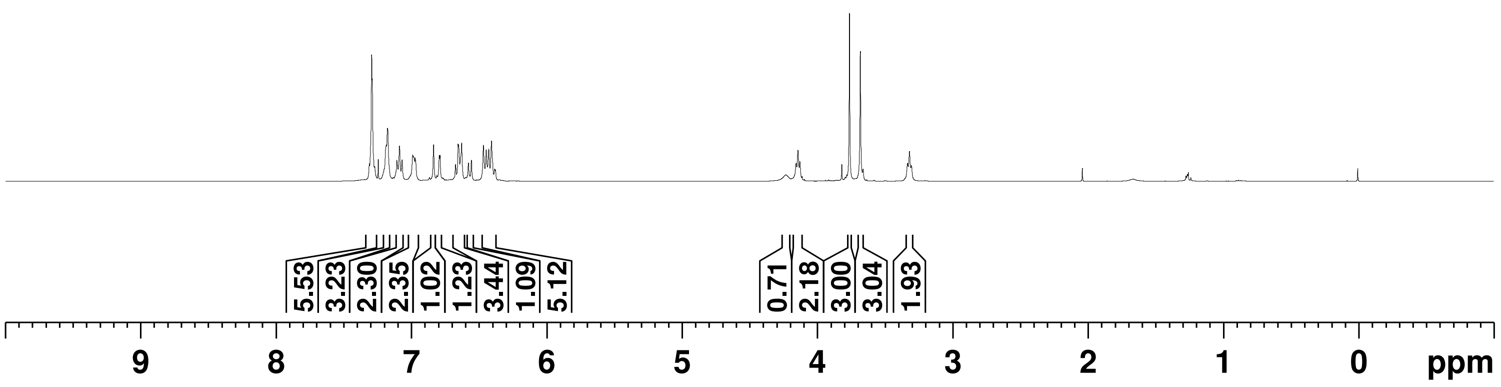


```
Current Data Parameters
NAME          1H_ST-6-103
EXPNO                  10
PROCNO                  1

F2 - Acquisition Parameters
Date_            20210713
Time                14.09 h
INSTRUM            Avance
PROBHD     Z167430_0032 (
PULPROG              zg30
TD                  65536
SOLVENT             CDCl3
NS                     16
DS                      2
SWH              8196.722 Hz
FIDRES           0.250144 Hz
AQ              3.9976959 sec
RG                    101
DW                 61.000 usec
DE                  13.20 usec
TE                  298.0 K
D1             0.01000000 sec
TD0                     1
SFO1          400.3024719 MHz
NUC1                   1H
P0                   4.00 usec
P1                  12.00 usec
PLW1           8.80000019 W

F2 - Processing parameters
SI                  65536
SF            400.3000139 MHz
WDW                    no
SSB      0
LB       0 Hz
GB       0
PC                   1.00
```

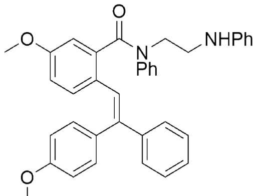

4q

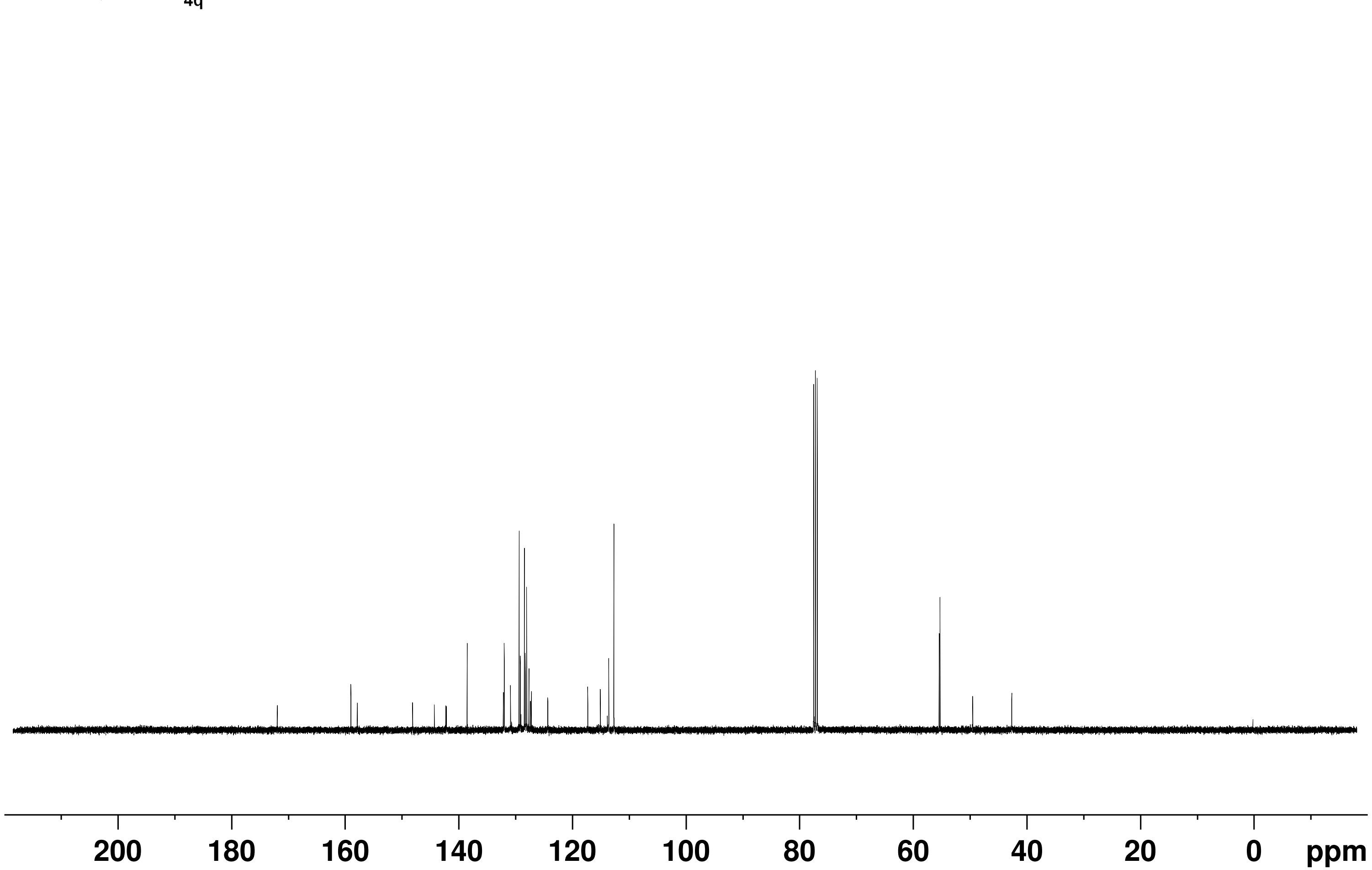

Current Data Parameters
NAME 13C_ST-6-103
EXPNO 1
PROCNO 1

F2 - Acquisition Parameters
Date_ 20210712
Time 17.56 h
INSTRUM Avance
PROBHD Z167430_0032 (
PULPROG zgpg30
TD 65536
SOLVENT CDCl3
NS 150
DS 4
SWH 23809.523 Hz
FIDRES 0.726609 Hz
AQ 1.3762560 sec
RG 3.25
DW 21.000 usec
DE 19.29 usec
TE 298.0 K
D1 2.00000000 sec
D11 0.03000000 sec
TD0 1
SFO1 100.6655806 MHz
NUC1 13C
P0 3.33 usec
P1 10.00 usec
PLW1 39.31399918 W
SFO2 400.3016012 MHz
NUC2 1H
CPDPRG[2 waltz64
PCPD2 80.00 usec
PLW2 8.80000019 W
PLW12 0.20176961 W
PLW13 0.10112690 W

F2 - Processing parameters
SI 131072
SF 100.6555056 MHz
WDW no
SSB 0
LB 0 Hz
GB 0
PC 1.40

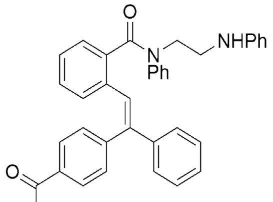

4r

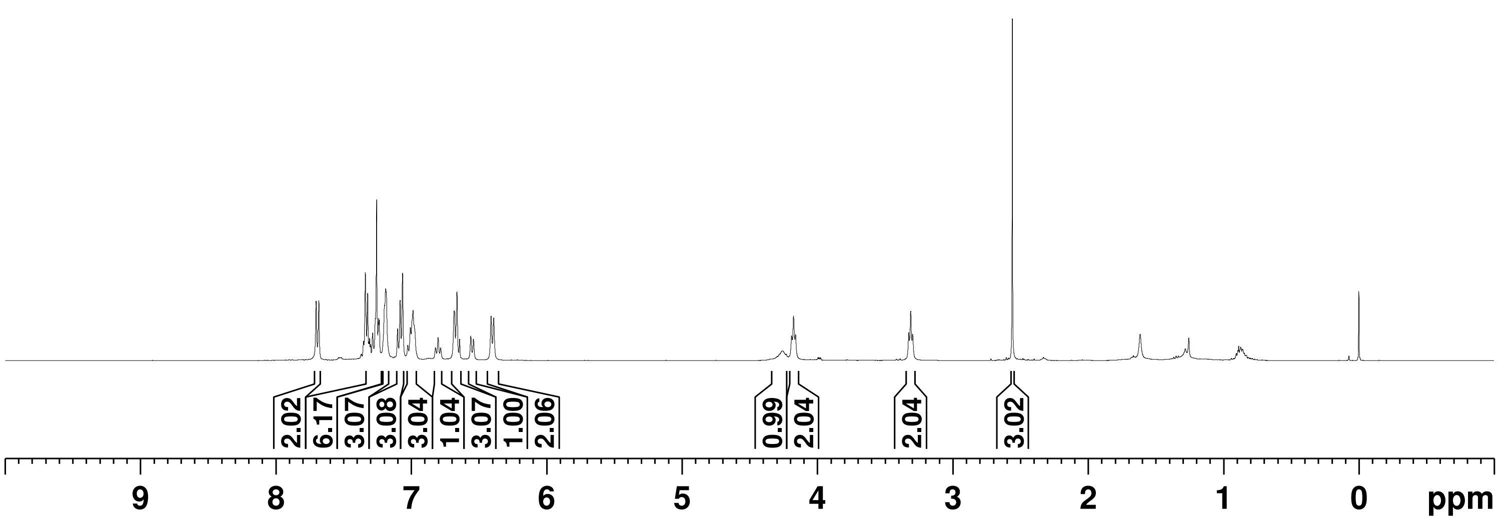

Current Data Parameters
NAME 1H ST-07-167-re2
EXPNO 1
PROCNO 1

F2 - Acquisition Parameters
Date_ 20220429
Time 9.04 h
INSTRUM Avance
PROBHD Z167430_0032 (
PULPROG zg30
TD 65536
SOLVENT CDCl3
NS 16
DS 0
SWH 8196.722 Hz
FIDRES 0.250144 Hz
AQ 3.9976959 sec
RG 101
DW 61.000 usec
DE 13.20 usec
TE 298.0 K
D1 0.10000000 sec
TD0 1
SFO1 400.3024719 MHz
NUC1 1H
P0 4.00 usec
P1 12.00 usec
PLW1 8.80000019 W

F2 - Processing parameters
SI 65536
SF 400.3000117 MHz
WDW EM
SSB 0
LB 0.30 Hz
GB 0
PC 1.00

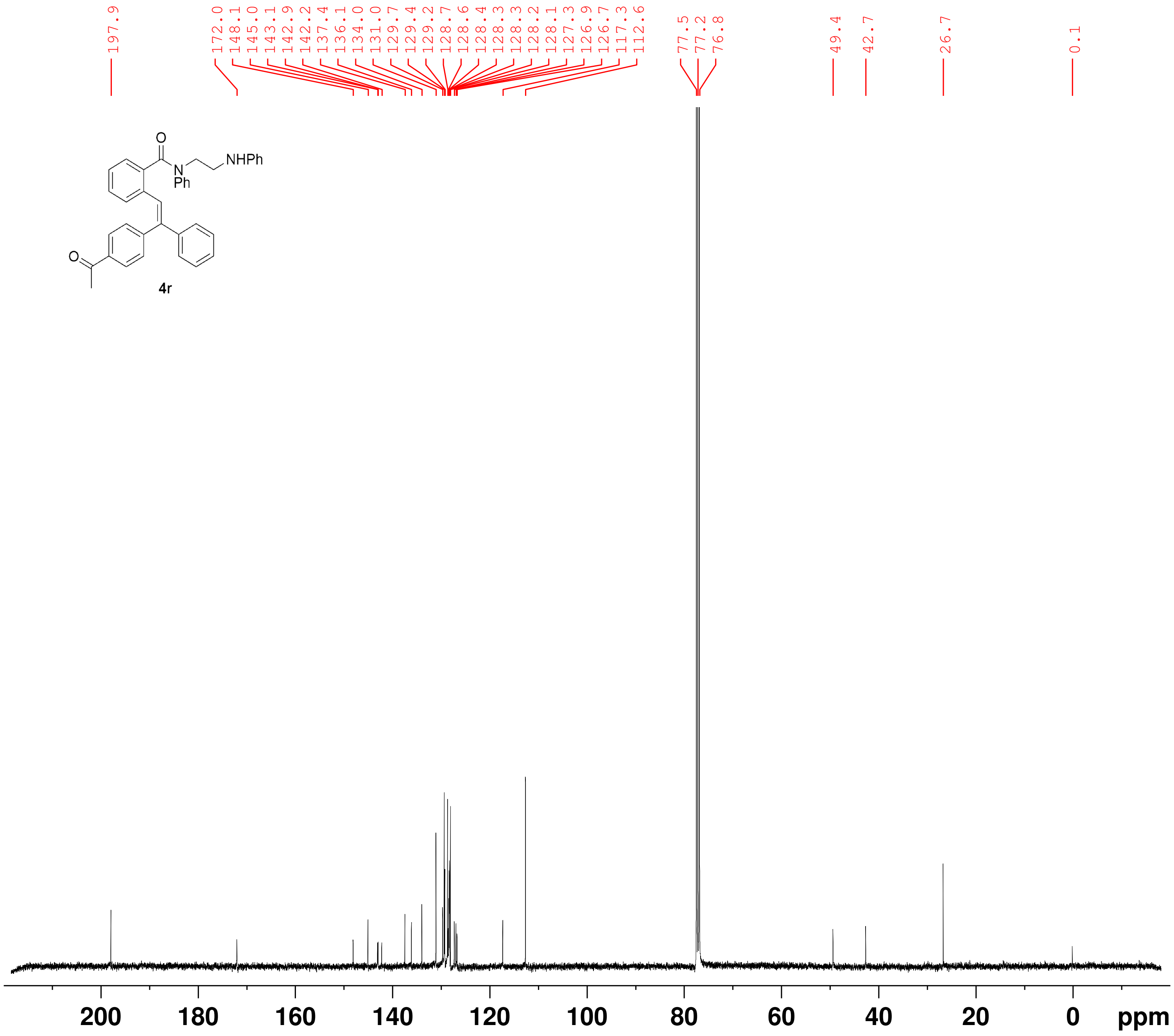


```
Current Data Parameters
NAME          13C_ST-7-167
EXPNO                    2
PROCNO                   1

F2 - Acquisition Parameters
Date_             20220428
Time                 11.44 h
INSTRUM             Avance
PROBHD   Z167430_0032 (
PULPROG             zgpg30
TD                   65536
SOLVENT              CDCl3
NS                     256
DS                       4
SWH              23809.523 Hz
FIDRES            0.726609 Hz
AQ               1.3762560 sec
RG                    3.25
DW                  21.000 usec
DE                   19.29 usec
TE                   298.0 K
D1              3.00000000 sec
D11             0.03000000 sec
TD0                      1
SFO1           100.6655806 MHz
NUC1                   13C
P0                    3.33 usec
P1                   10.00 usec
PLW1           39.31399918 W
SFO2           400.3016012 MHz
NUC2                    1H
CPDPRG[2           waltz64
PCPD2                80.00 usec
PLW2            8.80000019 W
PLW12           0.20176961 W
PLW13           0.10112690 W

F2 - Processing parameters
SI                  131072
SF             100.6555038 MHz
WDW                     EM
SSB      0
LB                    1.00 Hz
GB       0
PC                    1.40
```

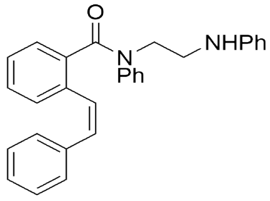

4s

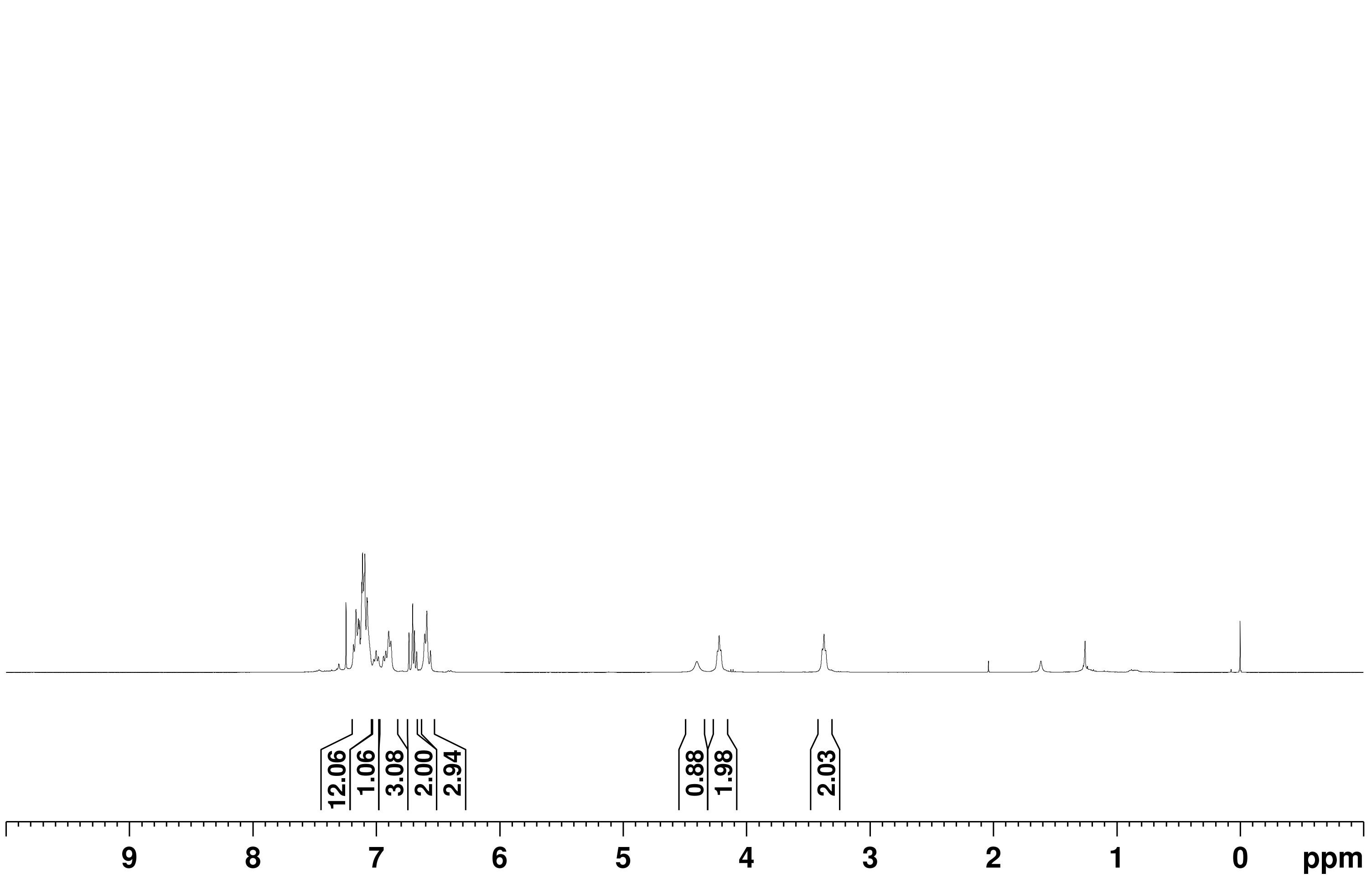


```
Current Data Parameters
NAME          1H_ST-6-11
EXPNO                  9
PROCNO                 1

F2 - Acquisition Parameters
Date_           20210713
Time               14.04 h
INSTRUM           Avance
PROBHD    Z167430_0032 (
PULPROG             zg30
TD                 65536
SOLVENT            CDCl3
NS                    16
DS                     2
SWH             8196.722 Hz
FIDRES          0.250144 Hz
AQ             3.9976959 sec
RG                   101
DW                61.000 usec
DE                 13.20 usec
TE                 298.0 K
D1            0.01000000 sec
TD0                    1
SFO1         400.3024719 MHz
NUC1                  1H
P0                  4.00 usec
P1                 12.00 usec
PLW1          8.80000019 W

F2 - Processing parameters
SI                 65536
SF           400.3000142 MHz
WDW                   no
SSB      0
LB       0 Hz
GB       0
PC                  1.00
```

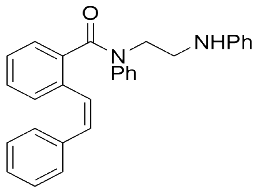

4s

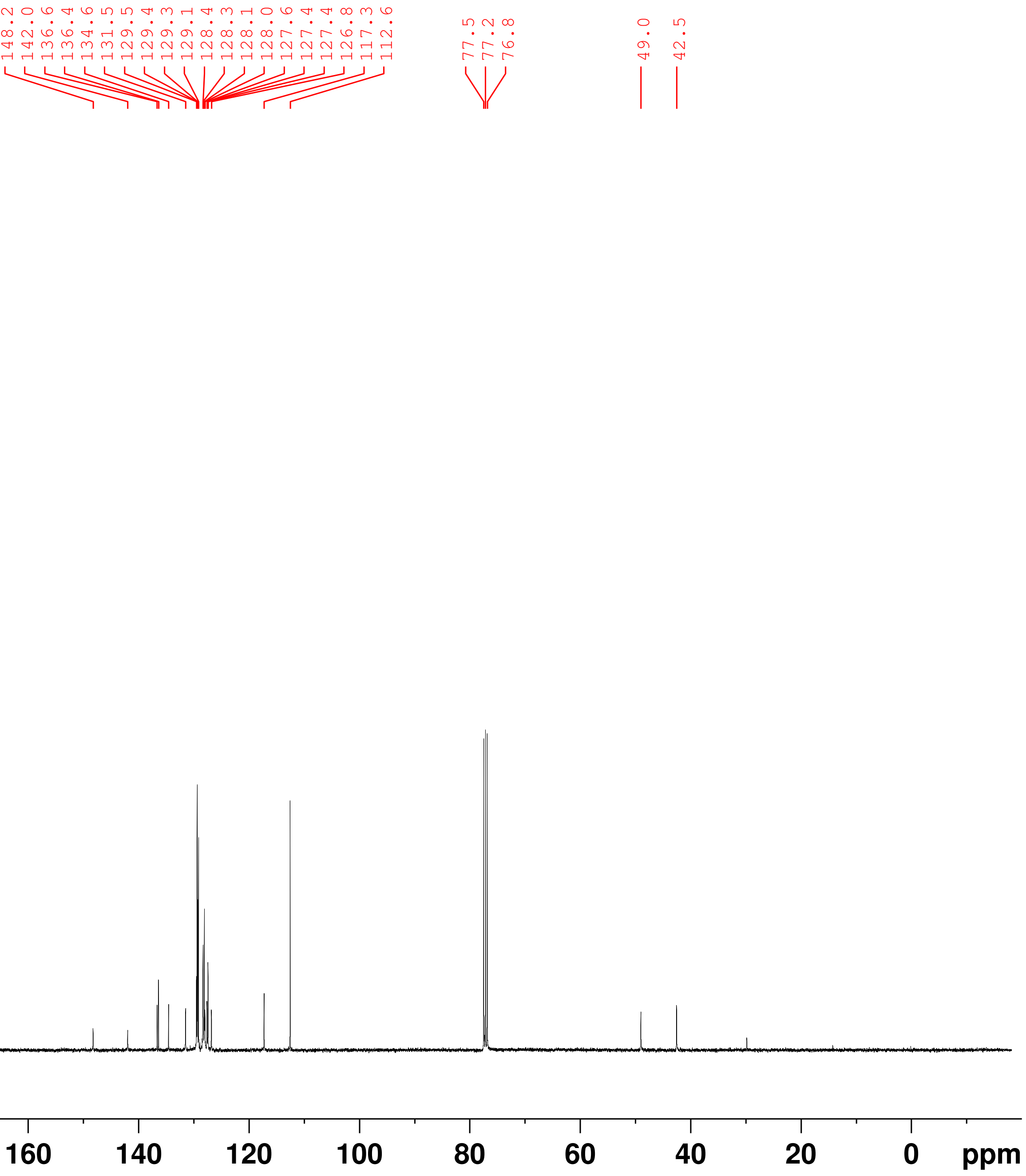

Current Data Parameters
NAME 13C_ST-6-11
EXPNO 1
PROCNO 1

F2 - Acquisition Parameters
Date_ 20210713
Time 11.13 h
INSTRUM Avance
PROBHD Z167430_0032 (
PULPROG zgpg30
TD 65536
SOLVENT CDCl3
NS 128
DS 4
SWH 23809.523 Hz
FIDRES 0.726609 Hz
AQ 1.3762560 sec
RG 3.25
DW 21.000 usec
DE 19.29 usec
TE 298.0 K
D1 2.00000000 sec
D11 0.03000000 sec
TD0 1
SFO1 100.6655806 MHz
NUC1 13C
P0 3.33 usec
P1 10.00 usec
PLW1 39.31399918 W
SFO2 400.3016012 MHz
NUC2 1H
CPDPRG[2 waltz64
PCPD2 80.00 usec
PLW2 8.80000019 W
PLW12 0.20176961 W
PLW13 0.10112690 W

F2 - Processing parameters
SI 131072
SF 100.6555069 MHz
WDW EM
SSB 0
LB 1.00 Hz
GB 0
PC 1.40

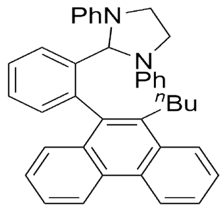

5

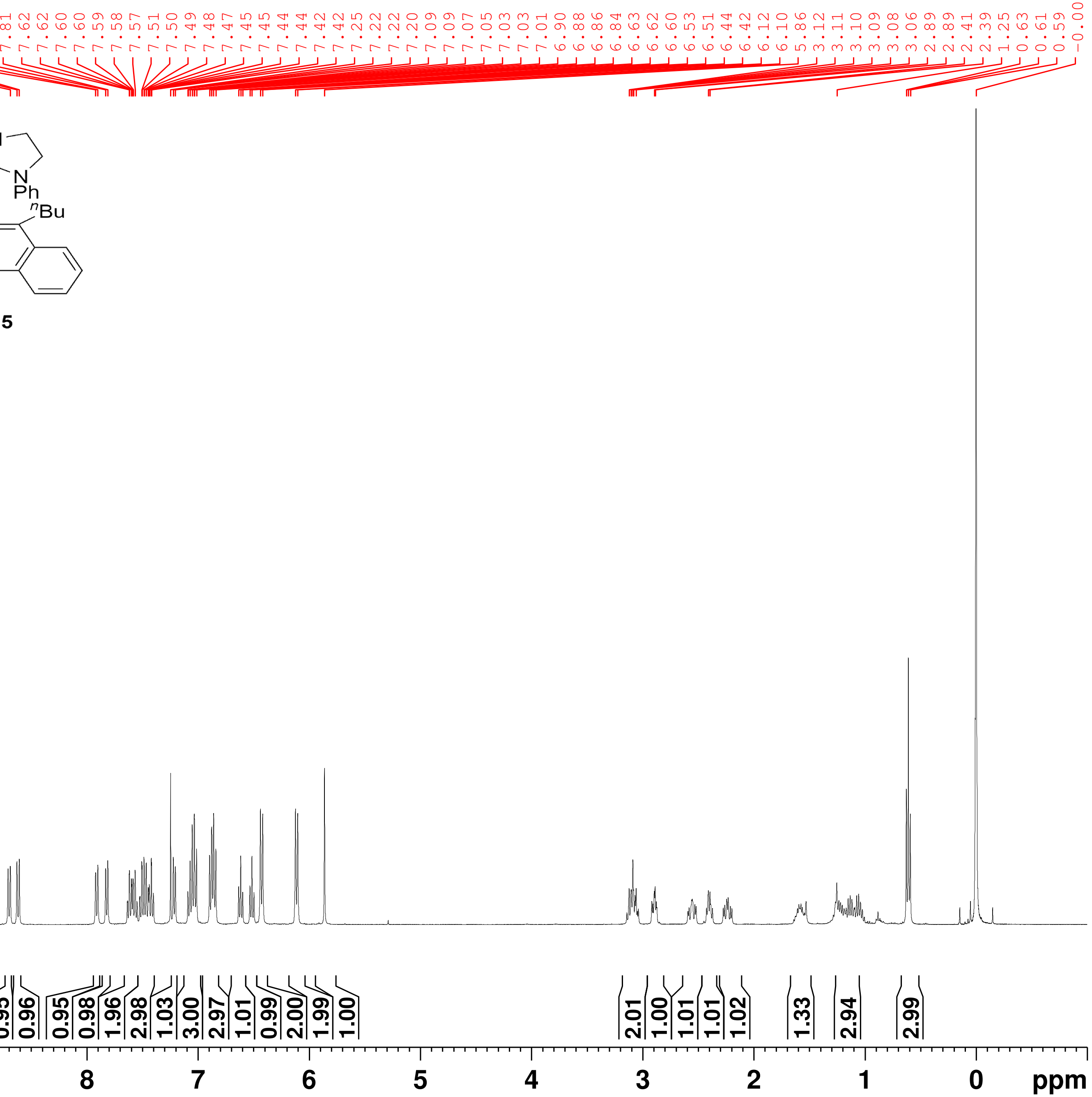


```
Current Data Parameters
NAME      1H_ST-4-94-cs2co3-re
EXPNO                        1
PROCNO                       1

F2 - Acquisition Parameters
Date_              20200210
Time                   8.56
INSTRUM               spect
PROBHD    5 mm Multinucl
PULPROG                zg30
TD                    32768
SOLVENT               CDCl3
NS                       16
DS                        0
SWH                8012.820 Hz
FIDRES             0.244532 Hz
AQ                2.0447233 sec
RG                    161.3
DW                   62.400 usec
DE                     6.50 usec
TE                    293.2 K
D1               0.01000000 sec
TD0                       1

======== CHANNEL f1 ========
NUC1                     1H
P1                     7.20 usec
PL1                   -5.00 dB
SFO1            400.1332010 MHz

F2 - Processing parameters
SI                   131072
SF              400.1300153 MHz
WDW                      EM
SSB      0
LB                     0.25 Hz
GB       0
PC                     0.20
```

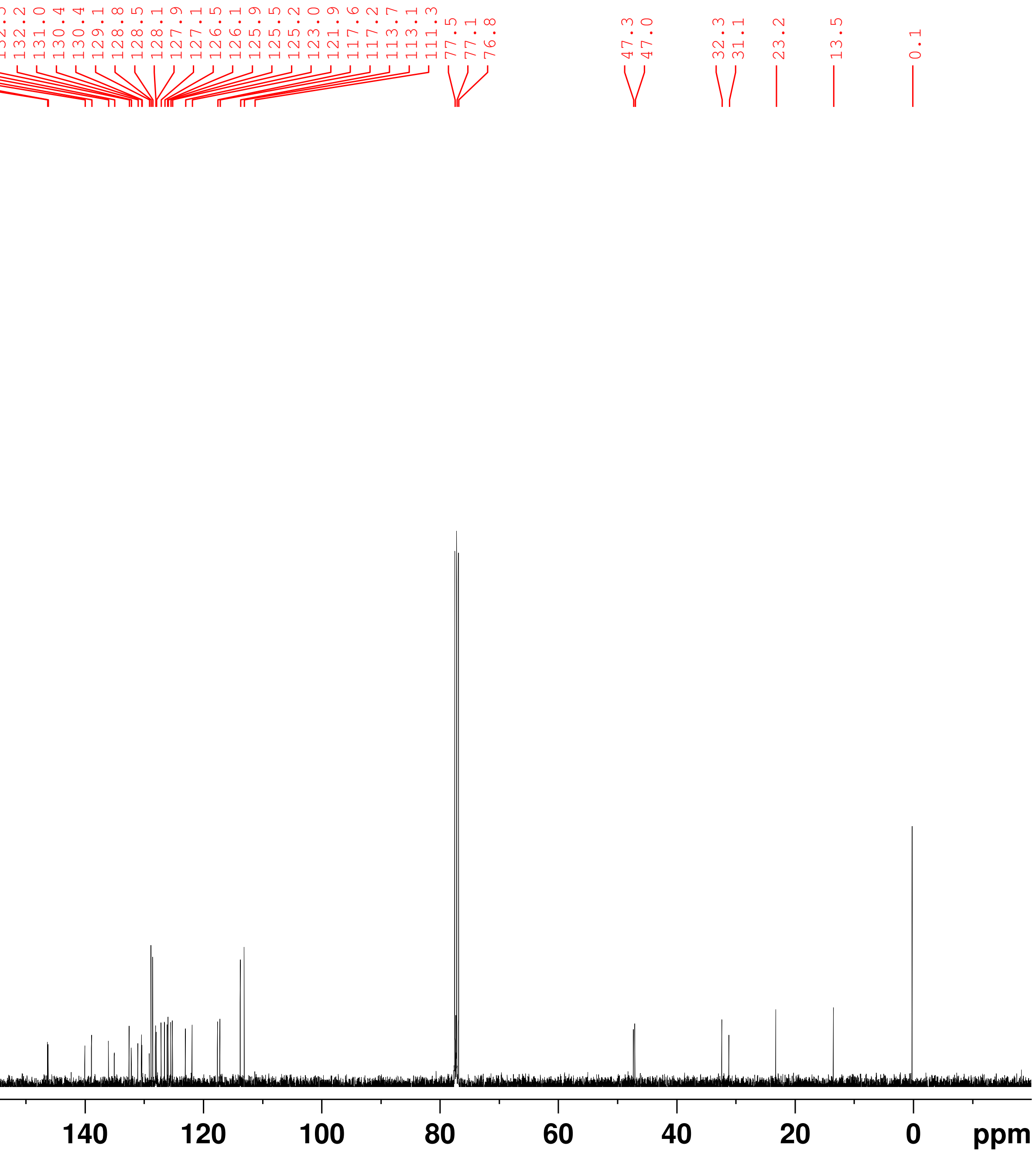

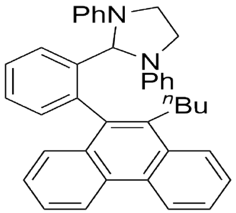

5

```
Current Data Parameters
NAME       13C_ST-4-95-cs2co3-re
EXPNO                          1
PROCNO                         1

F2 - Acquisition Parameters
Date_               20200210
Time                    9.10
INSTRUM                spect
PROBHD     5 mm Multinucl
PULPROG              zgdc30
TD                     65536
SOLVENT                CDCl3
NS                       350
DS                         4
SWH               26246.719 Hz
FIDRES             0.400493 Hz
AQ                1.2484608 sec
RG                    1625.5
DW                    19.050 usec
DE                      6.50 usec
TE                     293.2 K
D1                0.69999999 sec
d11               0.03000000 sec
TD0                        1

======== CHANNEL f1 ========
NUC1                     13C
P1                      8.07 usec
PL1                    -6.00 dB
SFO1             100.6196894 MHz

======== CHANNEL f2 ========
CPDPRG[2             waltz16
NUC2                      1H
PCPD2                  80.00 usec
PL2        0 dB
PL12                   18.00 dB
SFO2             400.1318006 MHz

F2 - Processing parameters
SI                    131072
SF               100.6127590 MHz
WDW                       EM
SSB        0
LB                      0.80 Hz
GB         0
PC                      0.50
```

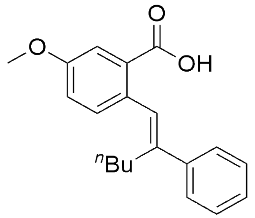

8k

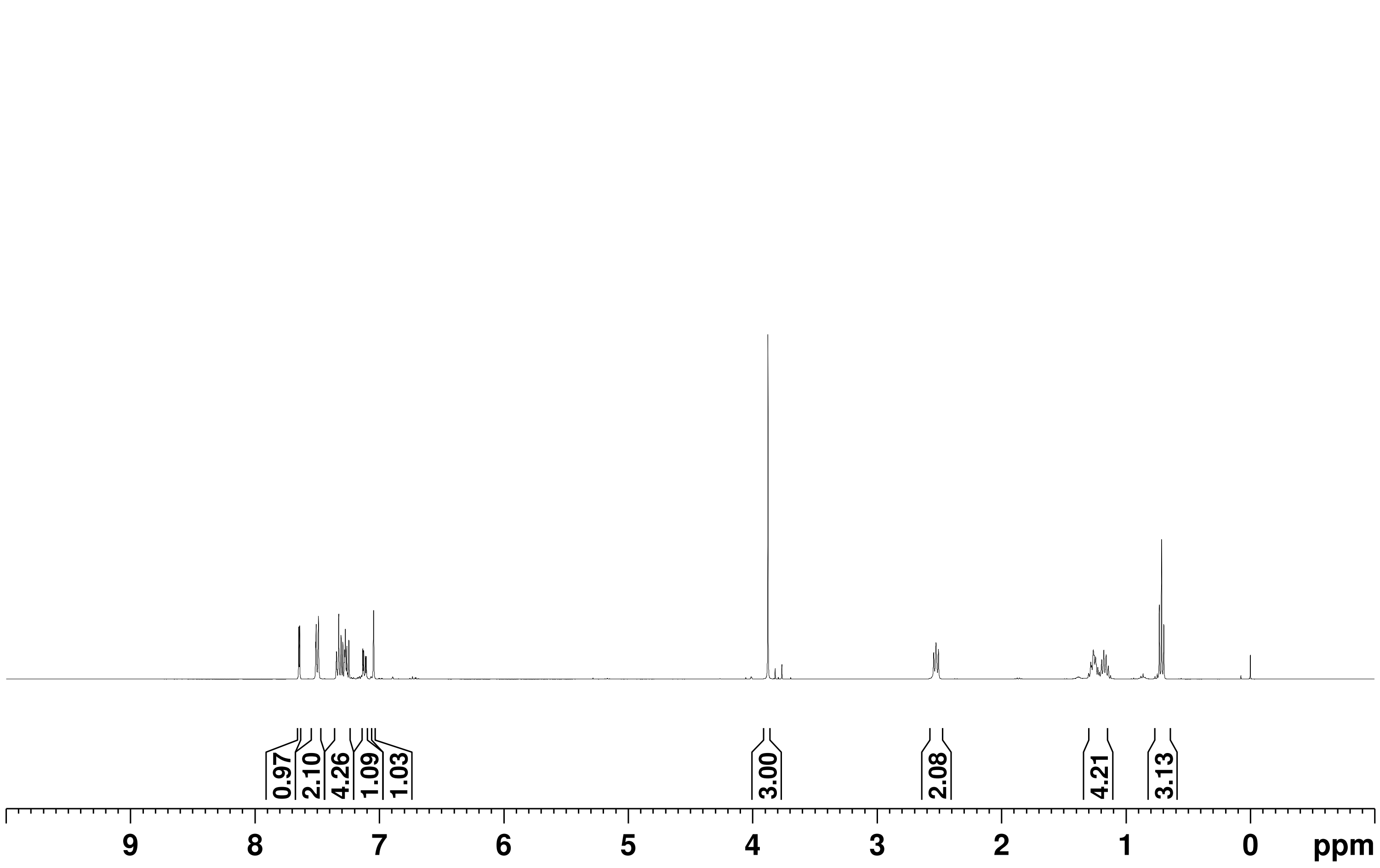

Current Data Parameters
NAME 1H_ST-6-113
EXPNO 1
PROCNO 1

F2 - Acquisition Parameters
Date_ 20210721
Time 8.41 h
INSTRUM Avance
PROBHD Z167430_0032 (
PULPROG zg30
TD 65536
SOLVENT CDCl3
NS 16
DS 2
SWH 8196.722 Hz
FIDRES 0.250144 Hz
AQ 3.9976959 sec
RG 101
DW 61.000 usec
DE 13.20 usec
TE 298.0 K
D1 0.01000000 sec
TD0 1
SFO1 400.3024719 MHz
NUC1 1H
P0 4.00 usec
P1 12.00 usec
PLW1 8.80000019 W

F2 - Processing parameters
SI 65536
SF 400.3000144 MHz
WDW no
SSB 0
LB 0 Hz
GB 0
PC 1.00

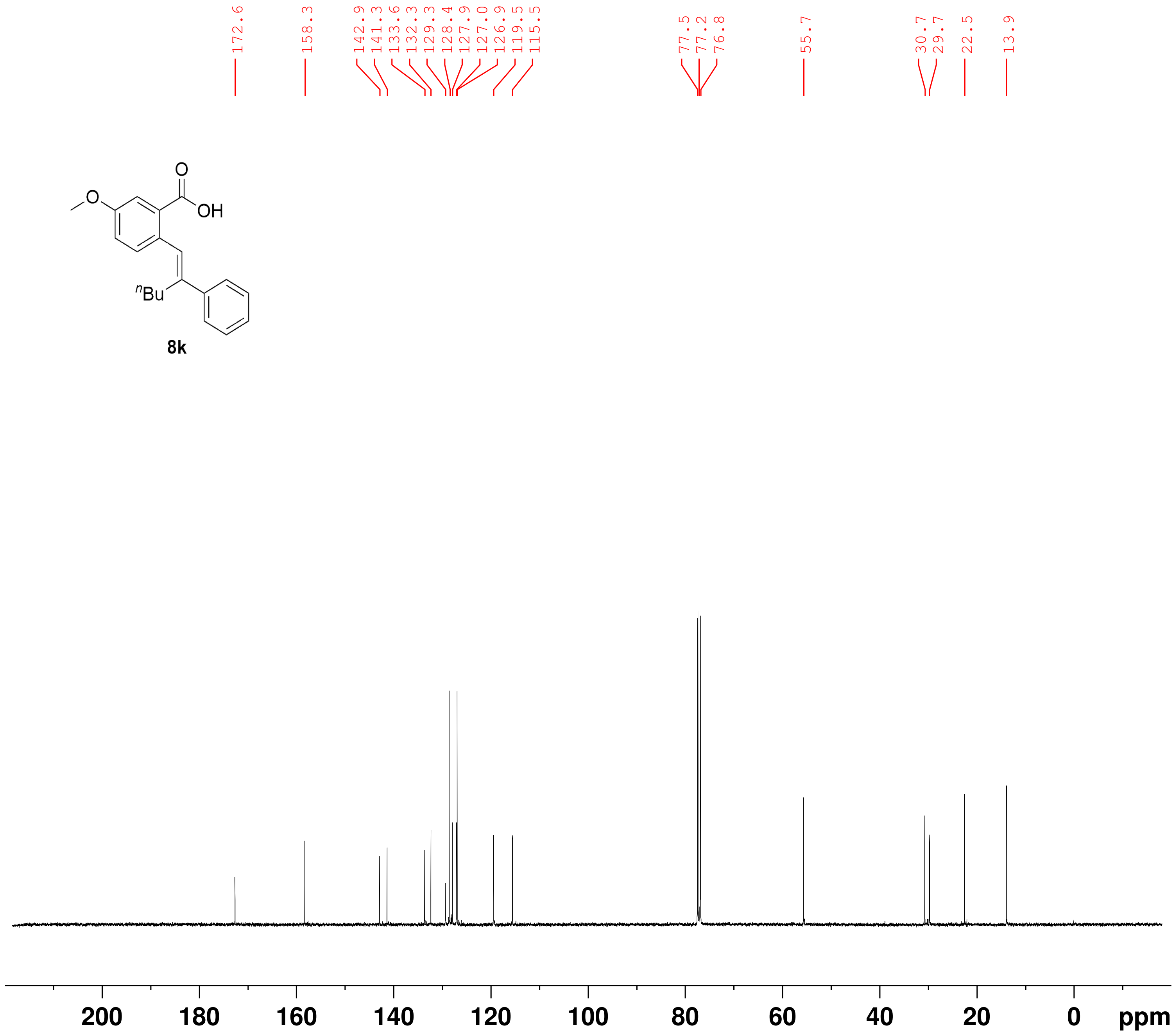

Current Data Parameters
NAME 13C_ST-6-113
EXPNO 1
PROCNO 1

F2 - Acquisition Parameters
Date_ 20210721
Time 8.50 h
INSTRUM Avance
PROBHD Z167430_0032 (
PULPROG zgpg30
TD 65536
SOLVENT CDCl3
NS 128
DS 4
SWH 23809.523 Hz
FIDRES 0.726609 Hz
AQ 1.3762560 sec
RG 3.25
DW 21.000 usec
DE 19.29 usec
TE 298.0 K
D1 2.00000000 sec
D11 0.03000000 sec
TD0 1
SFO1 100.6655806 MHz
NUC1 13C
P0 3.33 usec
P1 10.00 usec
PLW1 39.31399918 W
SFO2 400.3016012 MHz
NUC2 1H
CPDPRG[2 waltz64
PCPD2 80.00 usec
PLW2 8.80000019 W
PLW12 0.20176961 W
PLW13 0.10112690 W

F2 - Processing parameters
SI 131072
SF 100.6555029 MHz
WDW EM
SSB 0
LB 1.00 Hz
GB 0
PC 1.40

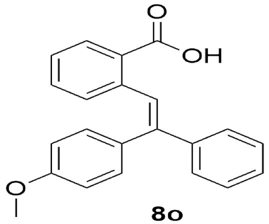

8o

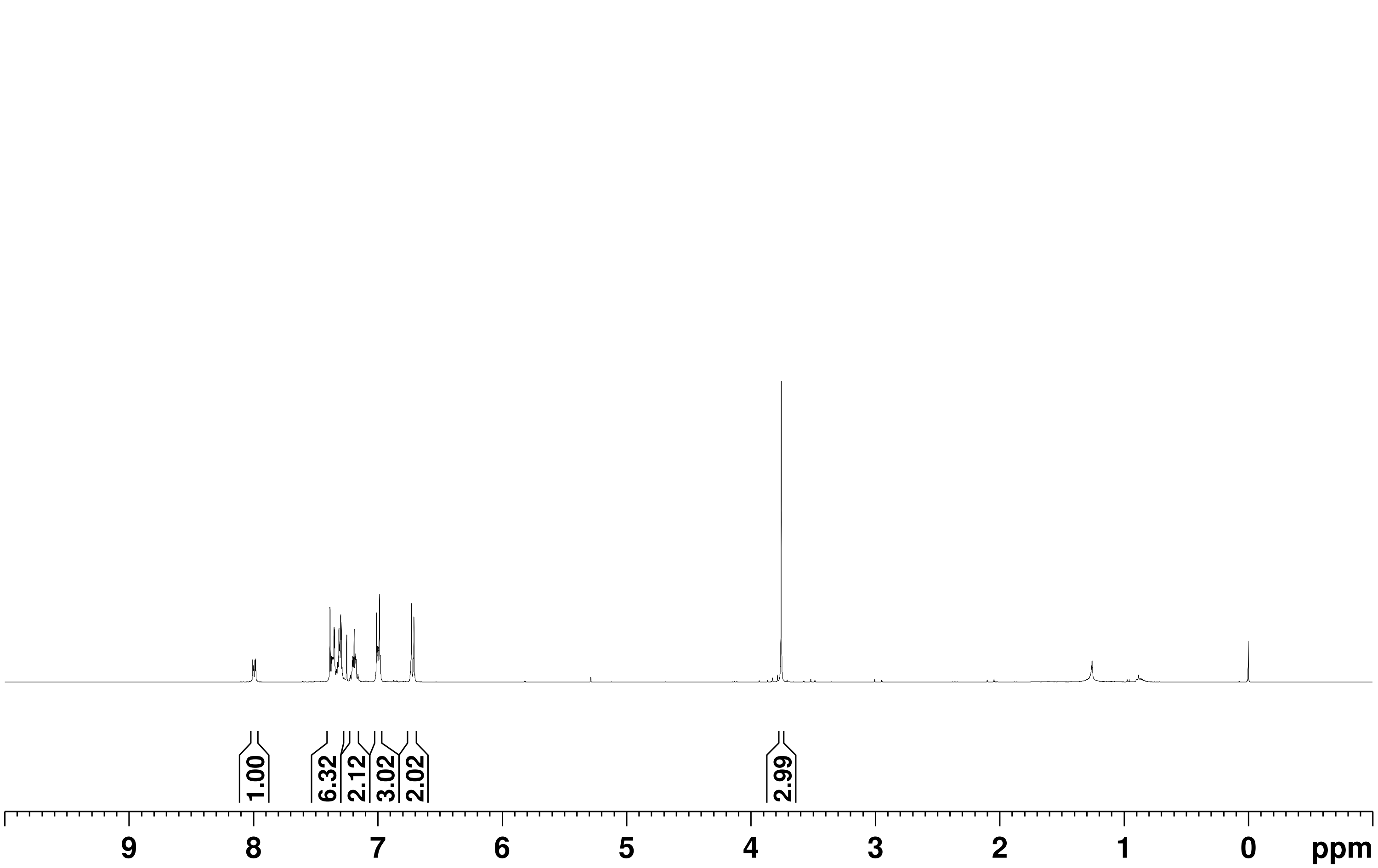

Current Data Parameters
NAME 1H_ST-6-71
EXPNO 1
PROCNO 1

F2 - Acquisition Parameters
Date_ 20210716
Time 13.37 h
INSTRUM Avance
PROBHD Z167430_0032 (
PULPROG zg30
TD 65536
SOLVENT CDCl3
NS 16
DS 2
SWH 8196.722 Hz
FIDRES 0.250144 Hz
AQ 3.9976959 sec
RG 101
DW 61.000 usec
DE 13.20 usec
TE 298.0 K
D1 1.00000000 sec
TD0 1
SFO1 400.3024719 MHz
NUC1 1H
P0 4.00 usec
P1 12.00 usec
PLW1 8.80000019 W

F2 - Processing parameters
SI 65536
SF 400.3000123 MHz
WDW no
SSB 0
LB 0 Hz
GB 0
PC 1.00

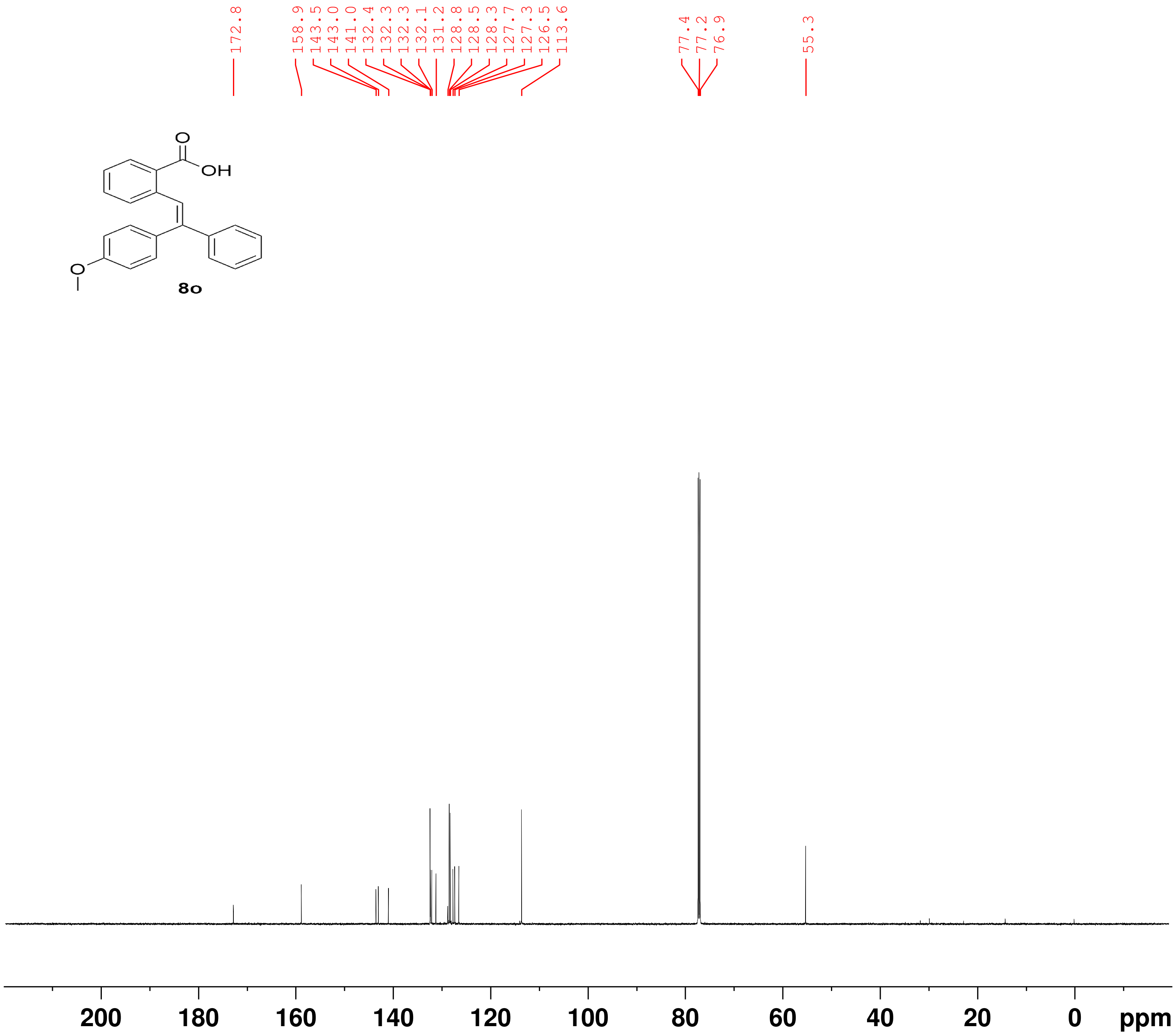

Current Data Parameters
NAME 13C_ST-6-71
EXPNO 10
PROCNO 1

F2 - Acquisition Parameters
Date_ 20210730
Time 18.39
INSTRUM spect
PROBHD 5 mm PABBO BB-
PULPROG zgpg30
TD 65536
SOLVENT CDCl3
NS 1024
DS 4
SWH 36057.691 Hz
FIDRES 0.550197 Hz
AQ 0.9087659 sec
RG 2050
DW 13.867 usec
DE 6.50 usec
TE 298.0 K
D1 2.00000000 sec
D11 0.03000000 sec

======== CHANNEL f1 ========
NUC1 13C
P1 10.00 usec
PLW1 60.25600052 W
SFO1 150.9380173 MHz

======== CHANNEL f2 ========
CPDPRG[2 waltz16
NUC2 1H
PCPD2 70.00 usec
PLW2 26.91500092 W
PLW12 0.79097998 W
PLW13 0.38758001 W
SFO2 600.2124008 MHz

F2 - Processing parameters
SI 65536
SF 150.9229088 MHz
WDW EM
SSB 0
LB 1.00 Hz
GB 0
PC 1.40

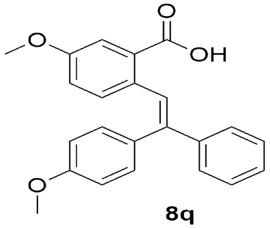

8q

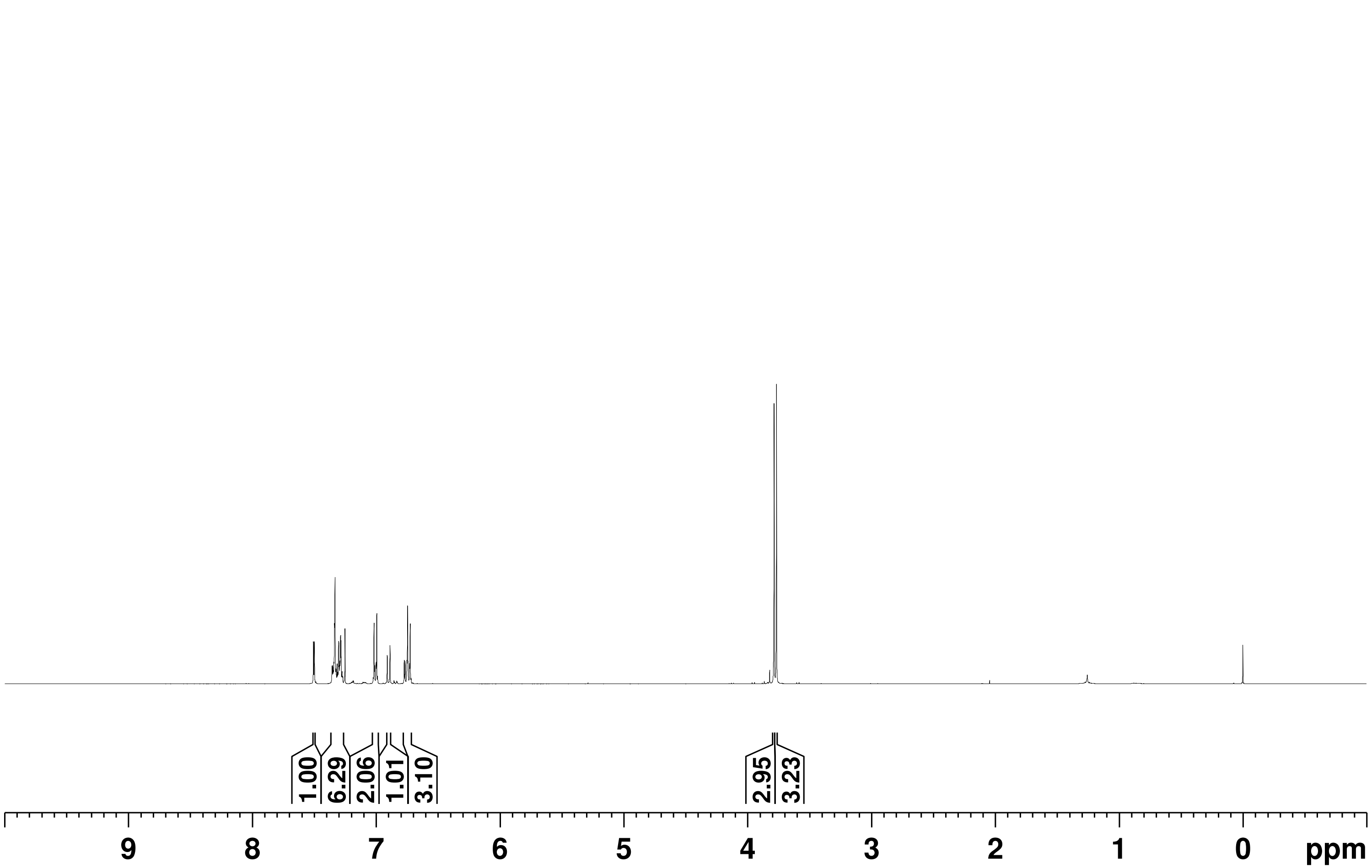


```
Current Data Parameters
NAME          1H_ST-6-115
EXPNO                   1
PROCNO                  1

F2 - Acquisition Parameters
Date_            20210721
Time                 8.57 h
INSTRUM            Avance
PROBHD   Z167430_0032 (
PULPROG              zg30
TD                  65536
SOLVENT             CDCl3
NS                     16
DS                      2
SWH              8196.722 Hz
FIDRES           0.250144 Hz
AQ              3.9976959 sec
RG                    101
DW                 61.000 usec
DE                  13.20 usec
TE                  298.0 K
D1             0.01000000 sec
TD0                     1
SFO1          400.3024719 MHz
NUC1                   1H
P0                   4.00 usec
P1                  12.00 usec
PLW1           8.80000019 W

F2 - Processing parameters
SI                  65536
SF            400.3000118 MHz
WDW                    no
SSB      0
LB       0 Hz
GB       0
PC                   1.00
```

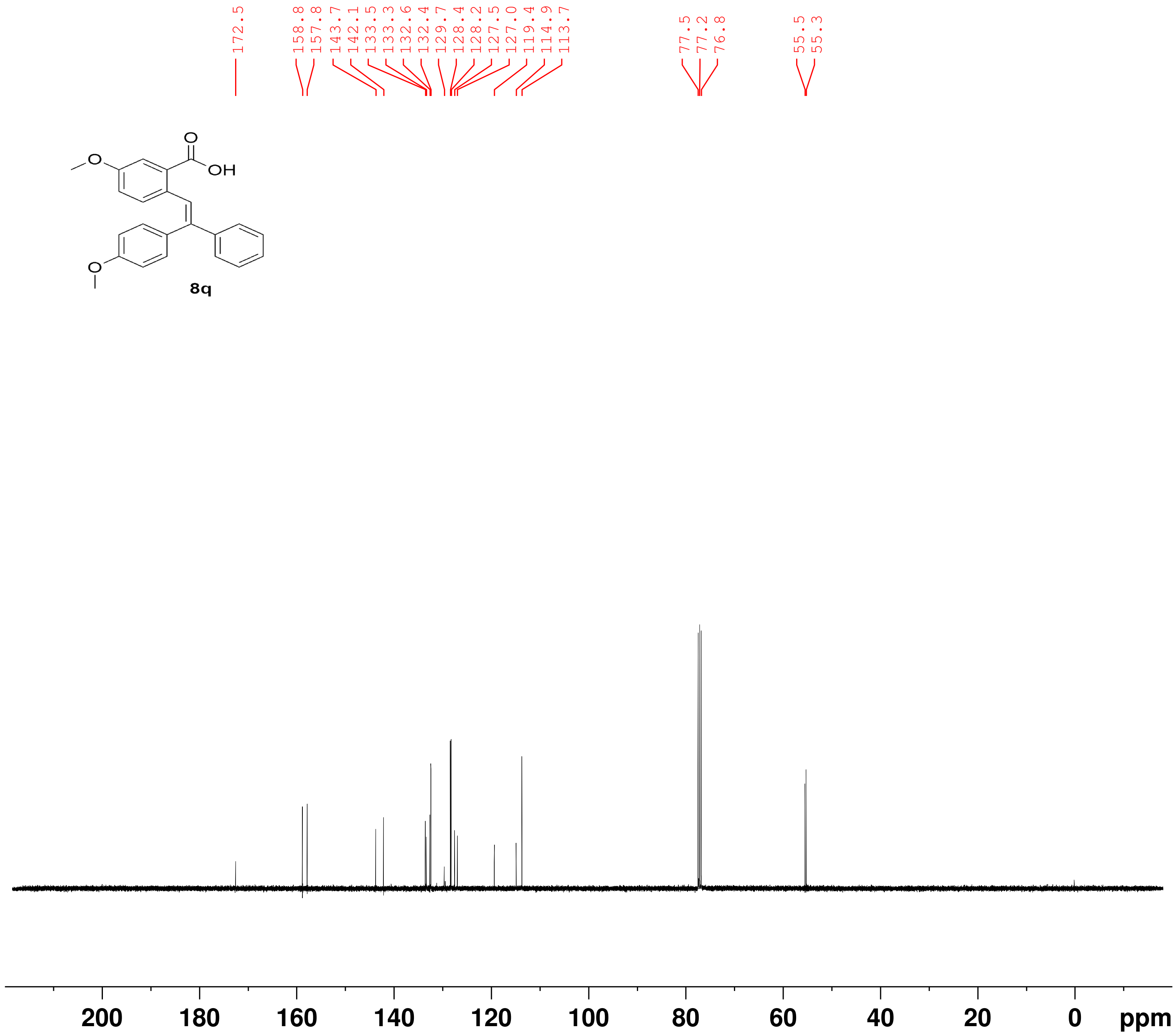

Current Data Parameters
NAME 13C_ST-6-115
EXPNO 1
PROCNO 1

F2 - Acquisition Parameters
Date_ 20210721
Time 9.07 h
INSTRUM Avance
PROBHD Z167430_0032 (
PULPROG zgpg30
TD 65536
SOLVENT CDCl3
NS 150
DS 4
SWH 23809.523 Hz
FIDRES 0.726609 Hz
AQ 1.3762560 sec
RG 3.25
DW 21.000 usec
DE 19.29 usec
TE 298.0 K
D1 2.00000000 sec
D11 0.03000000 sec
TD0 1
SFO1 100.6655806 MHz
NUC1 13C
P0 3.33 usec
P1 10.00 usec
PLW1 39.31399918 W
SFO2 400.3016012 MHz
NUC2 1H
CPDPRG[2 waltz64
PCPD2 80.00 usec
PLW2 8.80000019 W
PLW12 0.20176961 W
PLW13 0.10112690 W

F2 - Processing parameters
SI 131072
SF 100.6555026 MHz
WDW no
SSB 0
LB 0 Hz
GB 0
PC 1.40

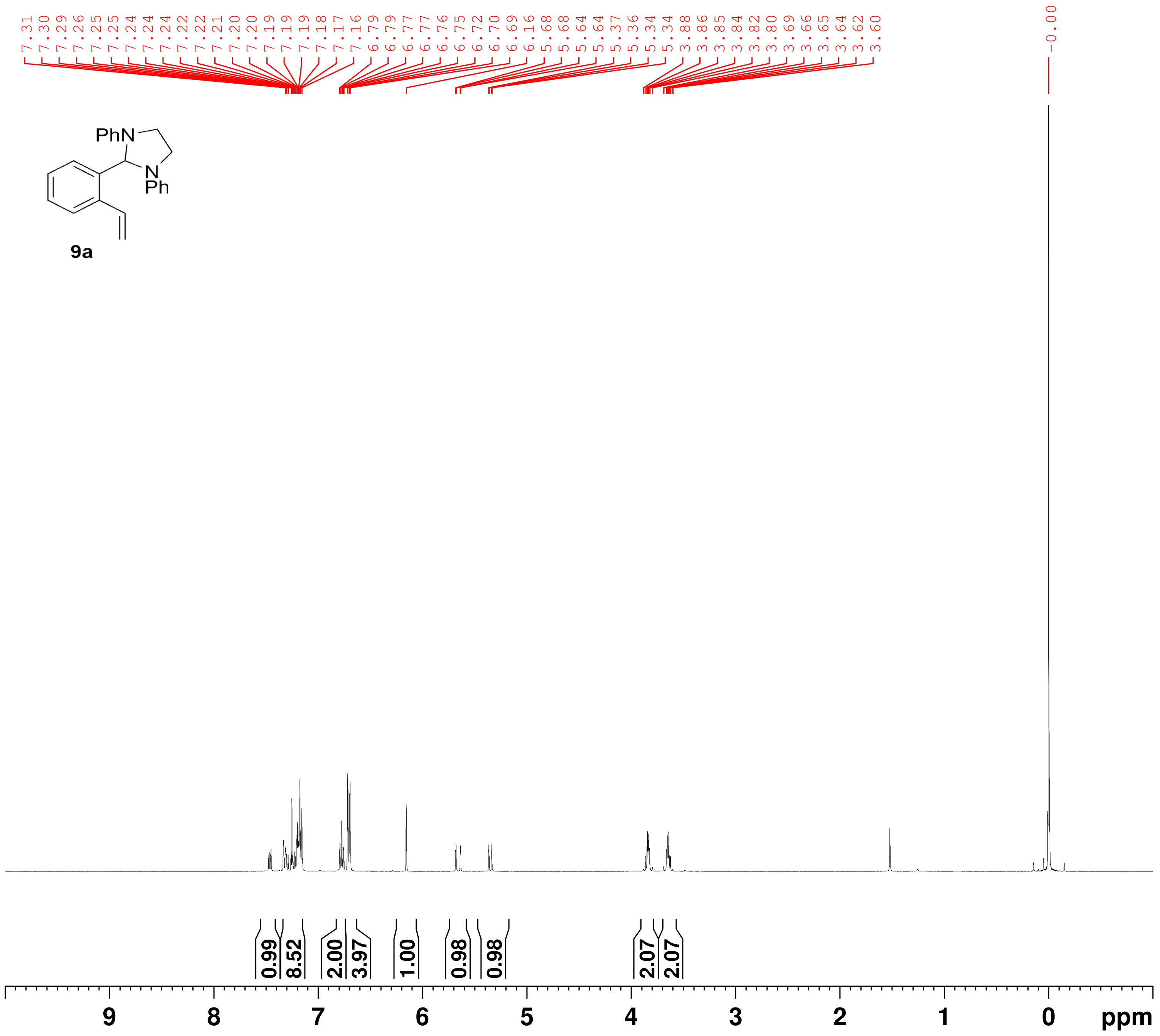

BRUKER

```
Current Data Parameters
NAME          1H_ST-3-287
EXPNO                   1
PROCNO                  1

F2 - Acquisition Parameters
Date_            20191111
Time                15.48
INSTRUM             spect
PROBHD   5 mm Multinucl
PULPROG              zg30
TD                  32768
SOLVENT             CDCl3
NS                     16
DS                      0
SWH              8012.820 Hz
FIDRES           0.244532 Hz
AQ              2.0447233 sec
RG                  228.1
DW                 62.400 usec
DE                   6.50 usec
TE                  298.2 K
D1             0.01000000 sec
TD0                     1

======== CHANNEL f1 ========
NUC1                   1H
P1                   7.20 usec
PL1                 -5.00 dB
SFO1          400.1332010 MHz

F2 - Processing parameters
SI                 131072
SF            400.1300135 MHz
WDW                    EM
SSB      0
LB                   0.25 Hz
GB       0
PC                   0.20
```

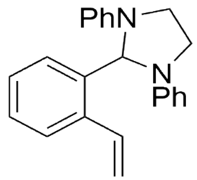

9a

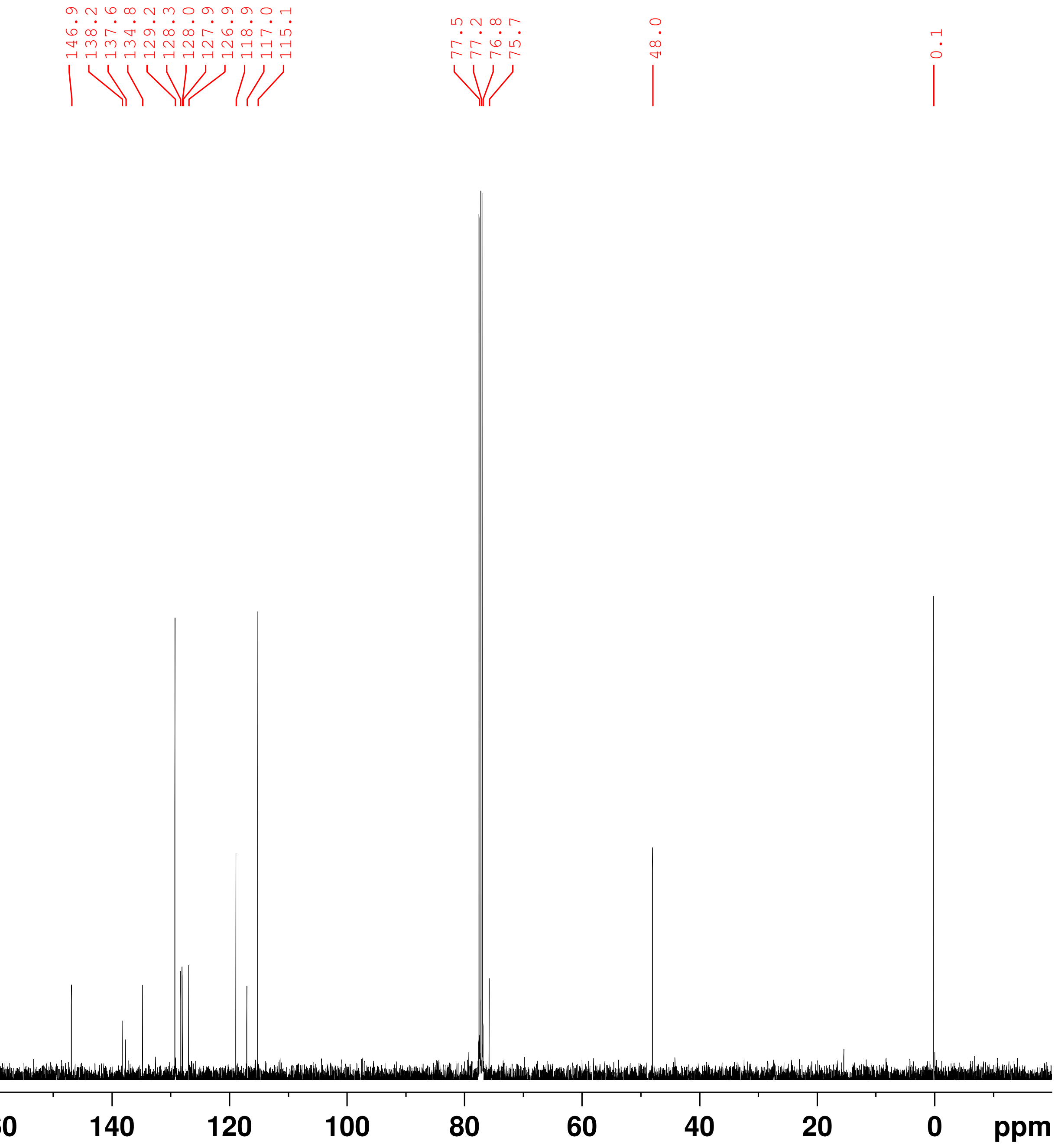

Current Data Parameters
NAME 13C_ST-3-287
EXPNO 1
PROCNO 1

F2 - Acquisition Parameters
Date_ 20191111
Time 16.01
INSTRUM spect
PROBHD 5 mm Multinucl
PULPROG zgdc30
TD 65536
SOLVENT CDCl3
NS 300
DS 4
SWH 26246.719 Hz
FIDRES 0.400493 Hz
AQ 1.2484608 sec
RG 512
DW 19.050 usec
DE 6.50 usec
TE 298.2 K
D1 0.69999999 sec
d11 0.03000000 sec
TD0 1

======== CHANNEL f1 ========
NUC1 13C
P1 8.07 usec
PL1 -6.00 dB
SFO1 100.6196894 MHz

======== CHANNEL f2 ========
CPDPRG[2 waltz16
NUC2 1H
PCPD2 80.00 usec
PL2 0 dB
PL12 18.00 dB
SFO2 400.1318006 MHz

F2 - Processing parameters
SI 131072
SF 100.6127545 MHz
WDW EM
SSB 0
LB 0.80 Hz
GB 0
PC 0.50

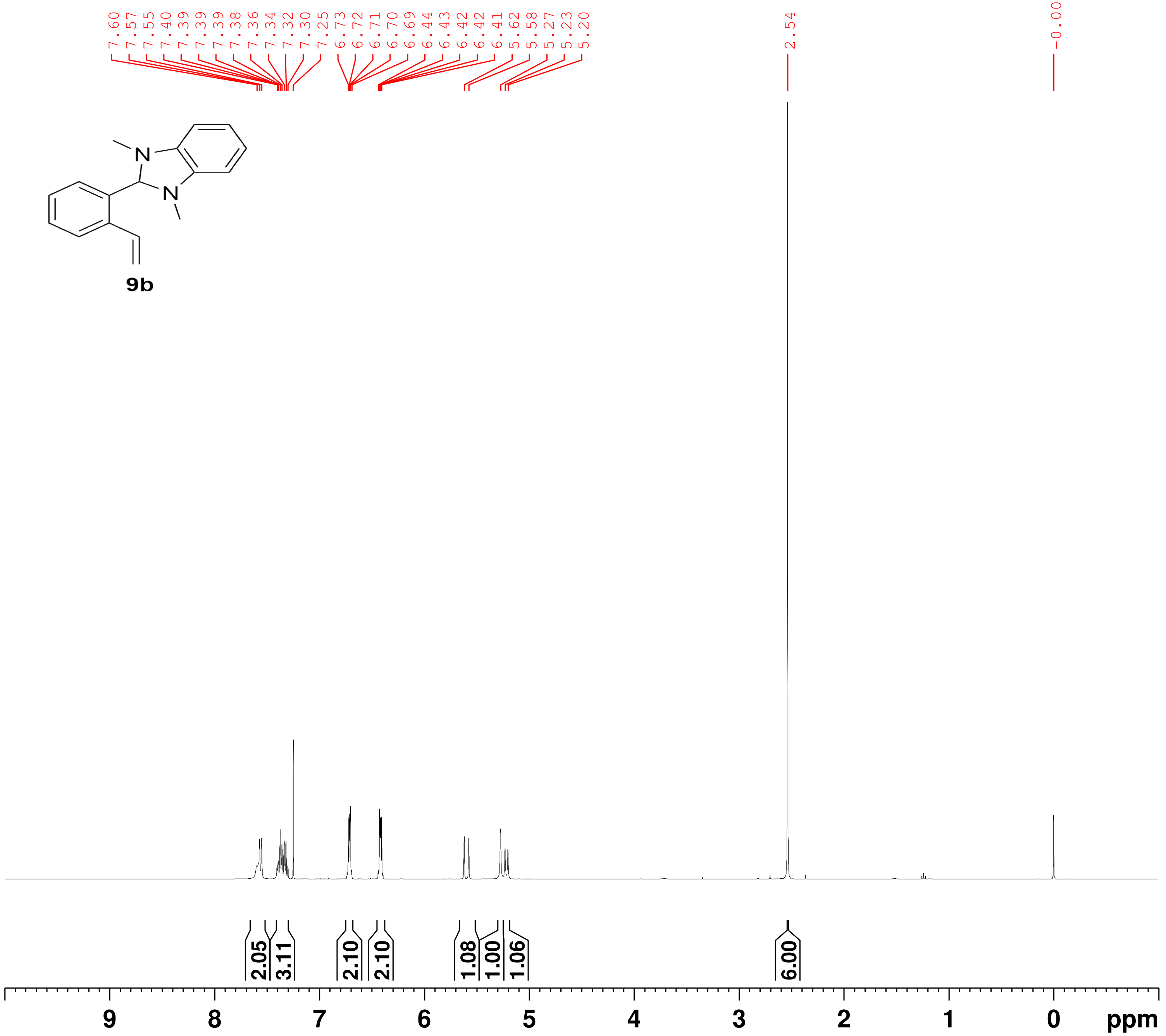


```
Current Data Parameters
NAME       1H_ST-6-295-ree
EXPNO                    1
PROCNO                   1

F2 - Acquisition Parameters
Date_             20220323
Time                 12.01 h
INSTRUM             Avance
PROBHD     Z167430_0032 (
PULPROG               zg30
TD                   65536
SOLVENT              CDCl3
NS                      16
DS                       2
SWH               8196.722 Hz
FIDRES            0.250144 Hz
AQ               3.9976959 sec
RG                     101
DW                  61.000 usec
DE                   13.20 usec
TE                   298.0 K
D1              1.00000000 sec
TD0                      1
SFO1           400.3024719 MHz
NUC1                    1H
P0                    4.00 usec
P1                   12.00 usec
PLW1            8.80000019 W

F2 - Processing parameters
SI                   65536
SF             400.3000139 MHz
WDW                     EM
SSB      0
LB                    0.30 Hz
GB       0
PC                    1.00
```

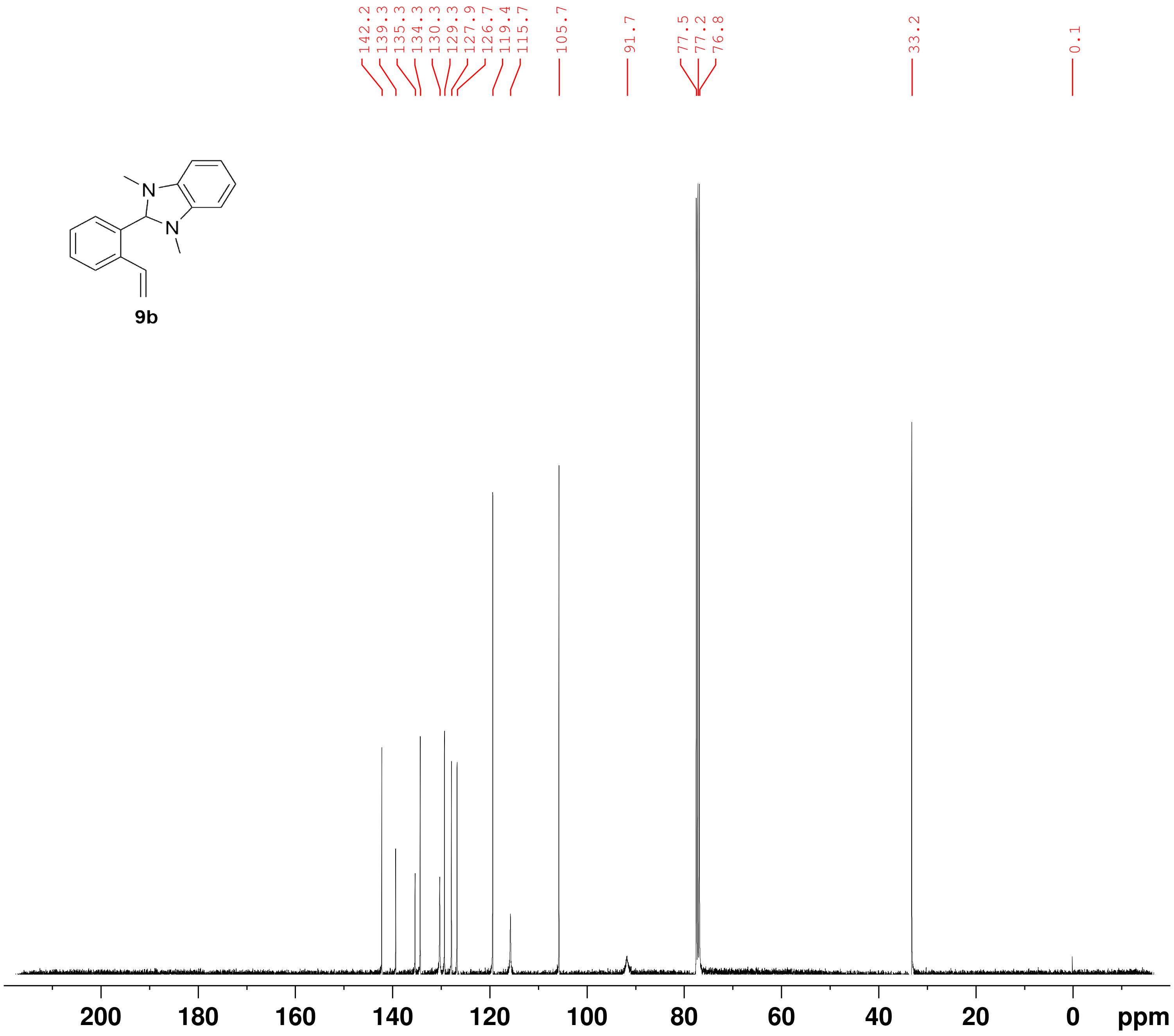

Current Data Parameters

NAME	13C_ST-6-275-reee
EXPNO	1
PROCNO	1

F2 - Acquisition Parameters

Date_	20211227
Time	14.31 h
INSTRUM	Avance
PROBHD	Z167430_0032 (
PULPROG	zgpg30
TD	65536
SOLVENT	CDCl3
NS	500
DS	4
SWH	23809.523 Hz
FIDRES	0.726609 Hz
AQ	1.3762560 sec
RG	3.25
DW	21.000 usec
DE	19.29 usec
TE	298.0 K
D1	2.00000000 sec
D11	0.03000000 sec
TD0	1
SFO1	100.6655806 MHz
NUC1	13C
P0	3.33 usec
P1	10.00 usec
PLW1	39.31399918 W
SFO2	400.3016012 MHz
NUC2	1H
CPDPRG[2	waltz64
PCPD2	80.00 usec
PLW2	8.80000019 W
PLW12	0.20176961 W
PLW13	0.10112690 W

F2 - Processing parameters

SI	131072
SF	100.6555040 MHz
WDW	EM
SSB	0
LB	1.00 Hz
GB	0
PC	1.40

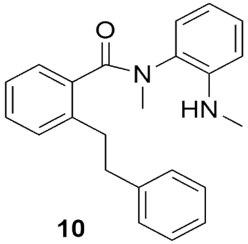

10

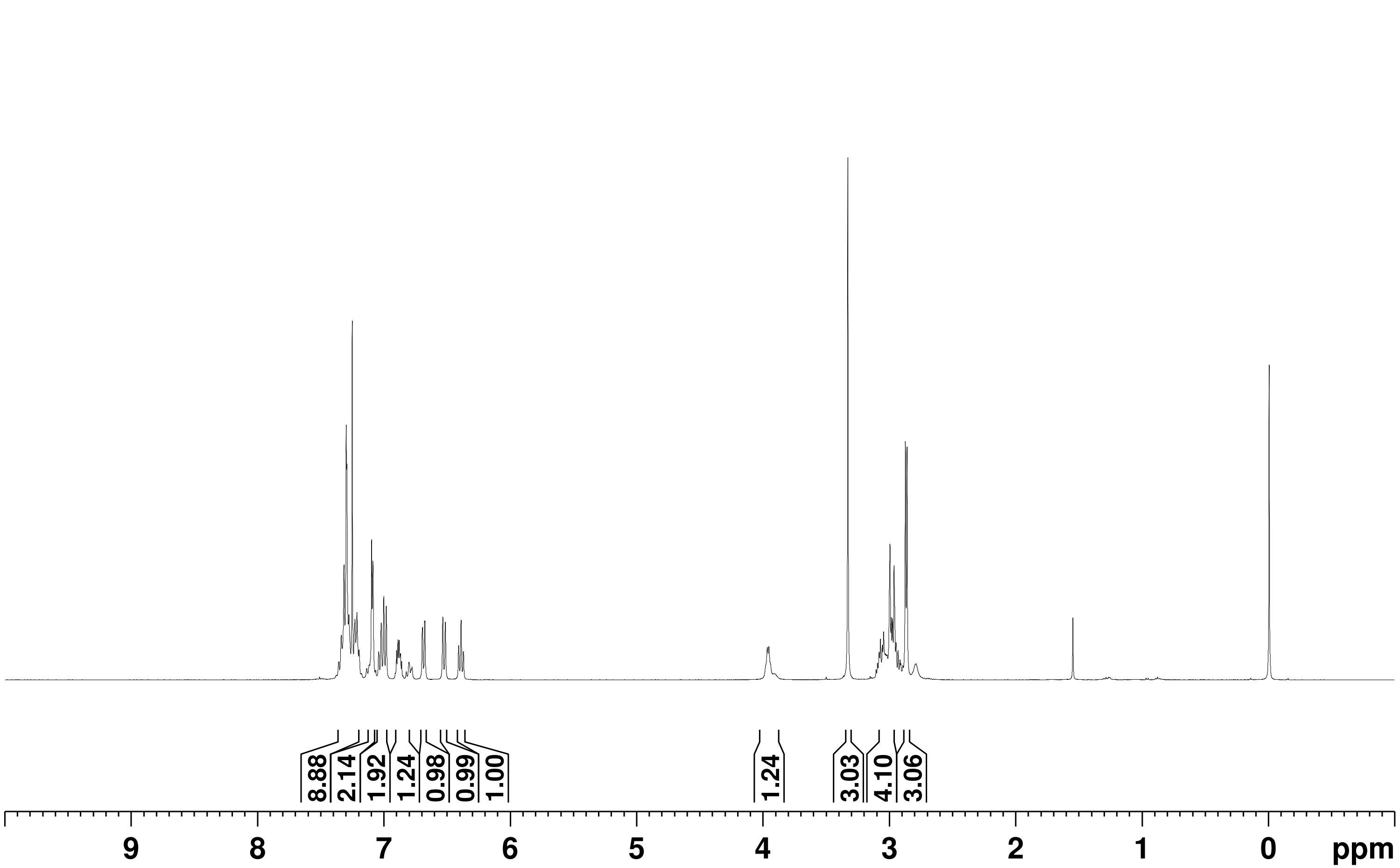


```
Current Data Parameters
NAME      1H_ST-6-redheck-rec-hexane
EXPNO                        1
PROCNO                       1

F2 - Acquisition Parameters
Date_               20220221
Time                   16.28 h
INSTRUM               Avance
PROBHD    Z167430_0032 (
PULPROG                 zg30
TD                     65536
SOLVENT                CDCl3
NS                        16
DS                         2
SWH                 8196.722 Hz
FIDRES              0.250144 Hz
AQ                 3.9976959 sec
RG                       101
DW                    61.000 usec
DE                     13.20 usec
TE                     298.0 K
D1                1.00000000 sec
TD0                        1
SFO1             400.3024719 MHz
NUC1                      1H
P0                      4.00 usec
P1                     12.00 usec
PLW1              8.80000019 W

F2 - Processing parameters
SI                     65536
SF               400.3000139 MHz
WDW                       EM
SSB          0
LB                      0.30 Hz
GB           0
PC                      1.00
```

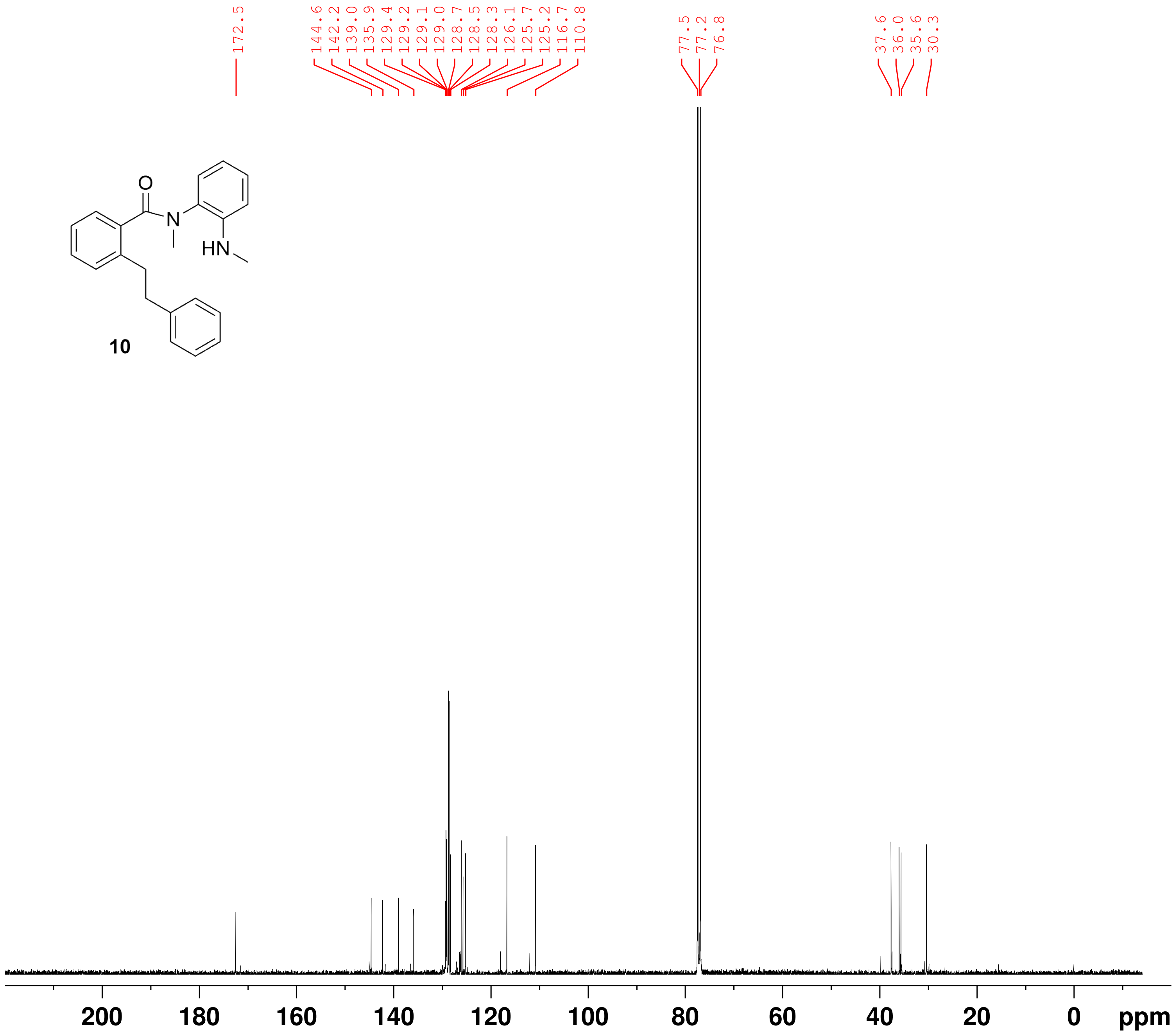

10

```
Current Data Parameters
NAME       13C_ST-6-277-1st
EXPNO                     2
PROCNO                    1

F2 - Acquisition Parameters
Date_              20211214
Time                  14.54 h
INSTRUM              Avance
PROBHD     Z167430_0032 (
PULPROG              zgpg30
TD                    65536
SOLVENT               CDCl3
NS                       97
DS                        4
SWH               25000.000 Hz
FIDRES             0.762939 Hz
AQ                1.3107200 sec
RG                     3.25
DW                   20.000 usec
DE                    18.29 usec
TE                    298.0 K
D1               2.00000000 sec
D11              0.03000000 sec
TD0                       1
SFO1            100.6665872 MHz
NUC1                    13C
P0                     3.33 usec
P1                    10.00 usec
PLW1            39.31399918 W
SFO2            400.3016012 MHz
NUC2                     1H
CPDPRG[2            waltz64
PCPD2                 80.00 usec
PLW2             8.80000019 W
PLW12            0.20176961 W
PLW13            0.10112690 W

F2 - Processing parameters
SI                   131072
SF              100.6555058 MHz
WDW                      EM
SSB      0
LB                     1.00 Hz
GB       0
PC                     1.40
```

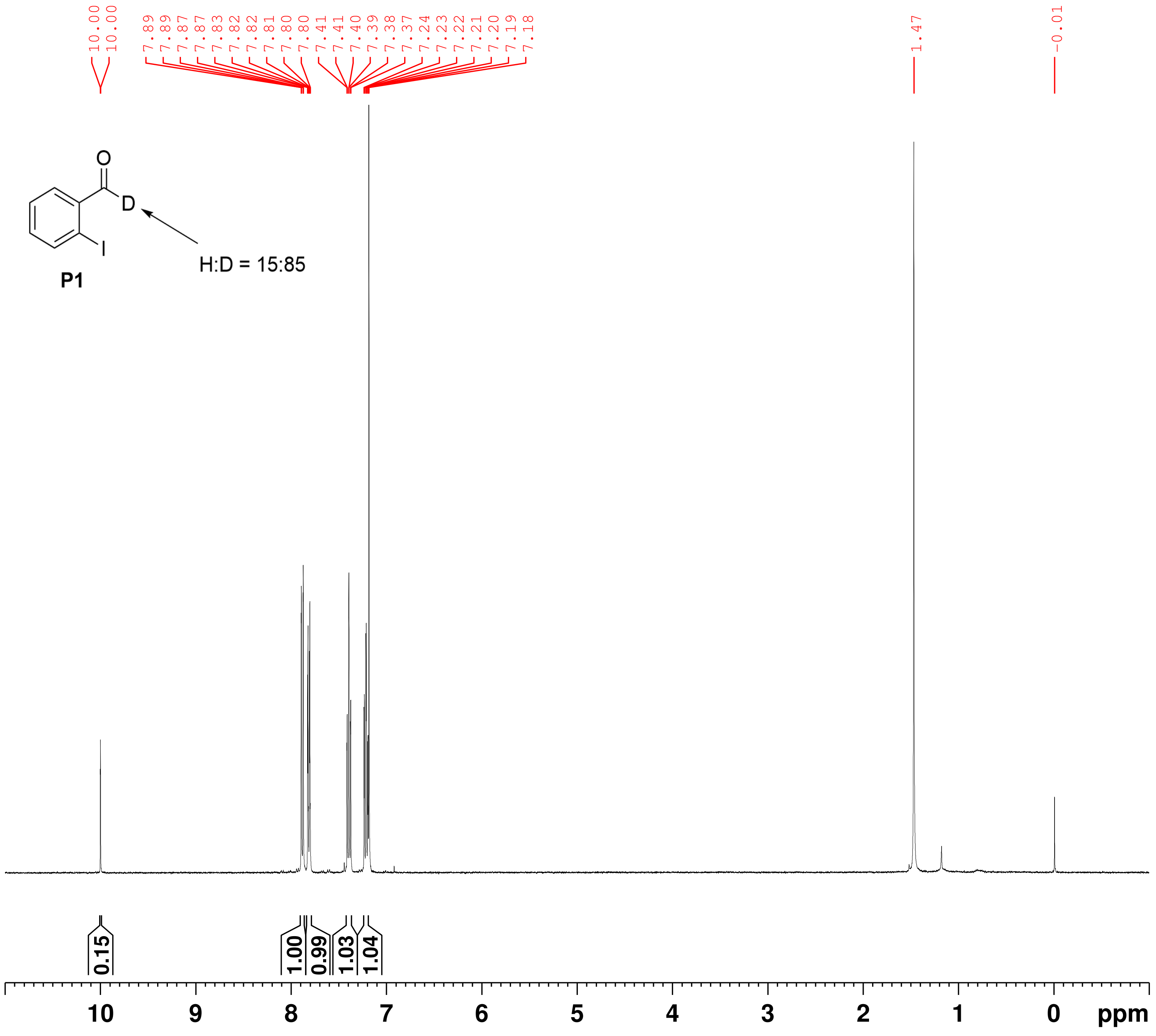


```
Current Data Parameters
NAME              1H_ST-3-143
EXPNO                       1
PROCNO                      1

F2 - Acquisition Parameters
Date_                20190829
Time                    16.23
INSTRUM                 spect
PROBHD     5 mm Multinucl
PULPROG                  zg30
TD                      32768
SOLVENT                 CDCl3
NS                         32
DS                          0
SWH                  8012.820 Hz
FIDRES               0.244532 Hz
AQ                  2.0447233 sec
RG                      645.1
DW                     62.400 usec
DE                       6.50 usec
TE                      298.2 K
D1                 0.01000000 sec
TD0                         1

======== CHANNEL f1 ========
NUC1                       1H
P1                       7.20 usec
PL1                     -5.00 dB
SFO1              400.1332010 MHz

F2 - Processing parameters
SI                     131072
SF                400.1300403 MHz
WDW                        EM
SSB          0
LB                       0.25 Hz
GB           0
PC                       0.20
```

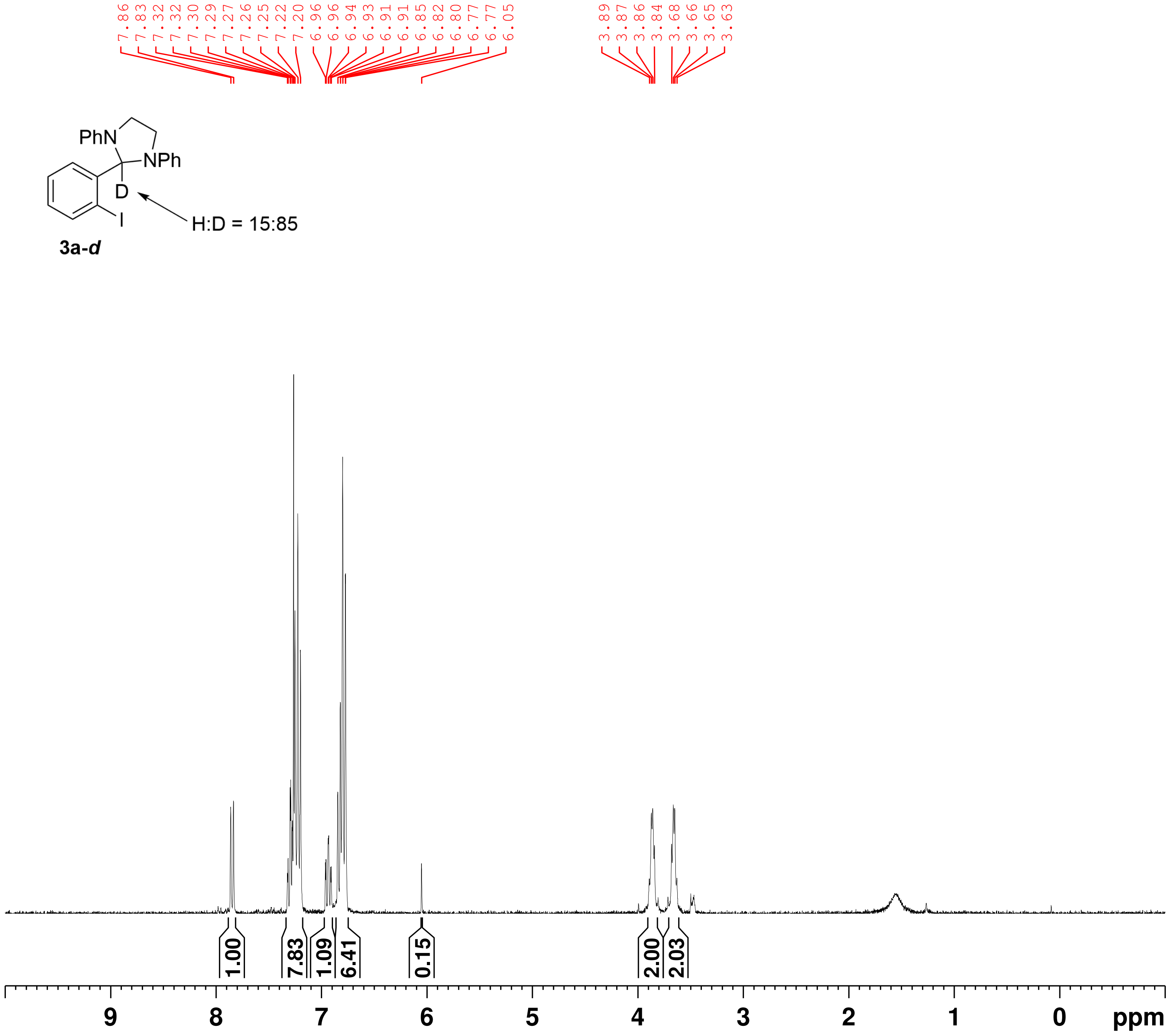

BRUKER

```
Current Data Parameters
NAME          1H-ST_3-145
EXPNO                   1
PROCNO                  1

F2 - Acquisition Parameters
Date_            20190830
Time                 9.02
INSTRUM             spect
PROBHD    5 mm QNP 1H/13
PULPROG              zg30
TD                  32768
SOLVENT             CDCl3
NS                     16
DS                      0
SWH              5995.204 Hz
FIDRES           0.182959 Hz
AQ              2.7328513 sec
RG                 1625.5
DW                 83.400 usec
DE                   6.50 usec
TE                  300.0 K
D1             0.01000000 sec
TD0                     1

======== CHANNEL f1 ========
NUC1                   1H
P1                   9.00 usec
PL1                 -0.50 dB
SFO1          300.1524012 MHz

F2 - Processing parameters
SI                  16384
SF            300.1500038 MHz
WDW                    EM
SSB       0
LB        0 Hz
GB        0
PC                   1.00
```

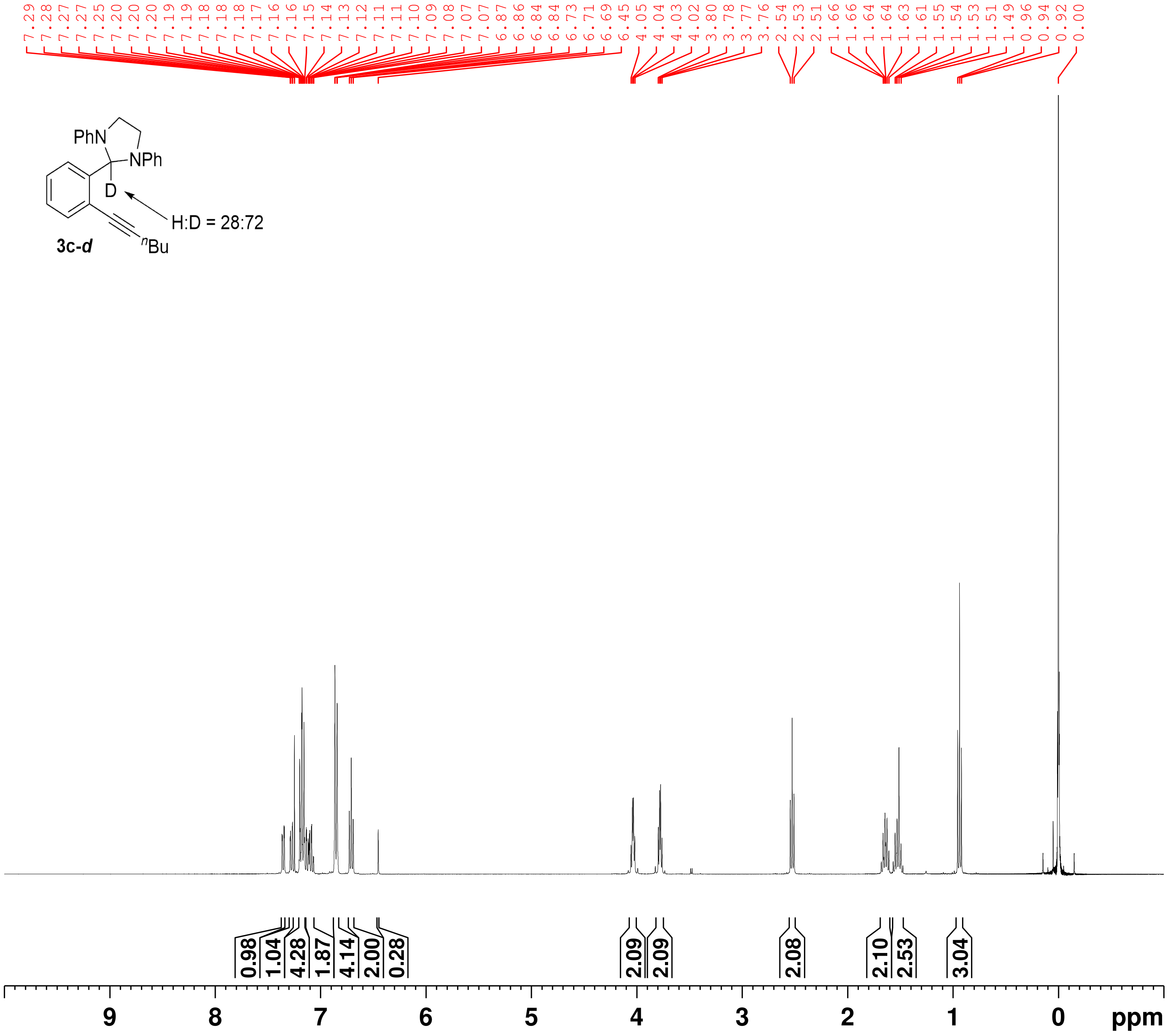


```
Current Data Parameters
NAME          1H_ST_3-183
EXPNO                   1
PROCNO                  1

F2 - Acquisition Parameters
Date_            20190916
Time                14.03
INSTRUM             spect
PROBHD    5 mm Multinucl
PULPROG              zg30
TD                  32768
SOLVENT             CDCl3
NS                     32
DS                      0
SWH              8012.820 Hz
FIDRES           0.244532 Hz
AQ              2.0447233 sec
RG                  203.2
DW                 62.400 usec
DE                   6.50 usec
TE                  298.2 K
D1             0.01000000 sec
TD0                     1

======== CHANNEL f1 ========
NUC1                   1H
P1                   7.20 usec
PL1                 -5.00 dB
SFO1          400.1332010 MHz

F2 - Processing parameters
SI                 131072
SF            400.1300147 MHz
WDW                    EM
SSB      0
LB                   0.25 Hz
GB       0
PC                   0.20
```

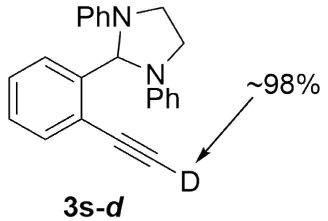

3s-*d*

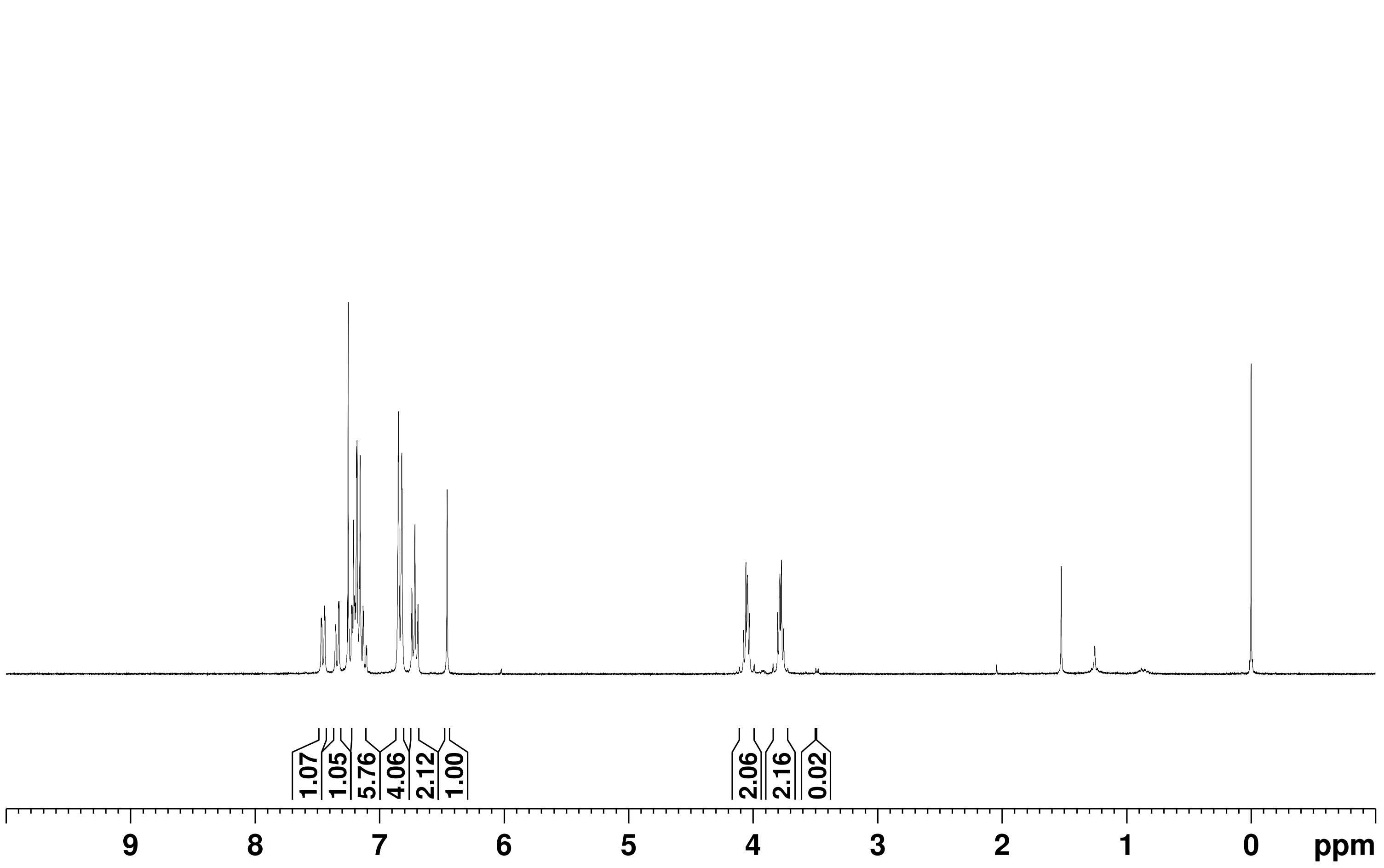


```
Current Data Parameters
NAME          1H_ST-7-3
EXPNO                 1
PROCNO                1

F2 - Acquisition Parameters
Date_          20220126
Time              10.20 h
INSTRUM  Avance NEO nanobay
PROBHD   Z104275_0486 (
PULPROG            zg30
TD                32768
SOLVENT           CDCl3
NS                   16
DS                    0
SWH            5882.353 Hz
FIDRES         0.359030 Hz
AQ            2.7852800 sec
RG                  101
DW               85.000 usec
DE                 6.50 usec
TE                298.5 K
D1           1.00000000 sec
TD0                   1
SFO1        300.1362005 MHz
NUC1                 1H
P0                 4.67 usec
P1                14.00 usec
PLW1         6.14340019 W

F2 - Processing parameters
SI                65536
SF          300.1350100 MHz
WDW                  no
SSB      0
LB       0 Hz
GB       0
PC                 1.00
```

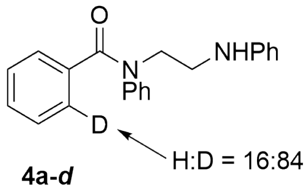

4a-*d*

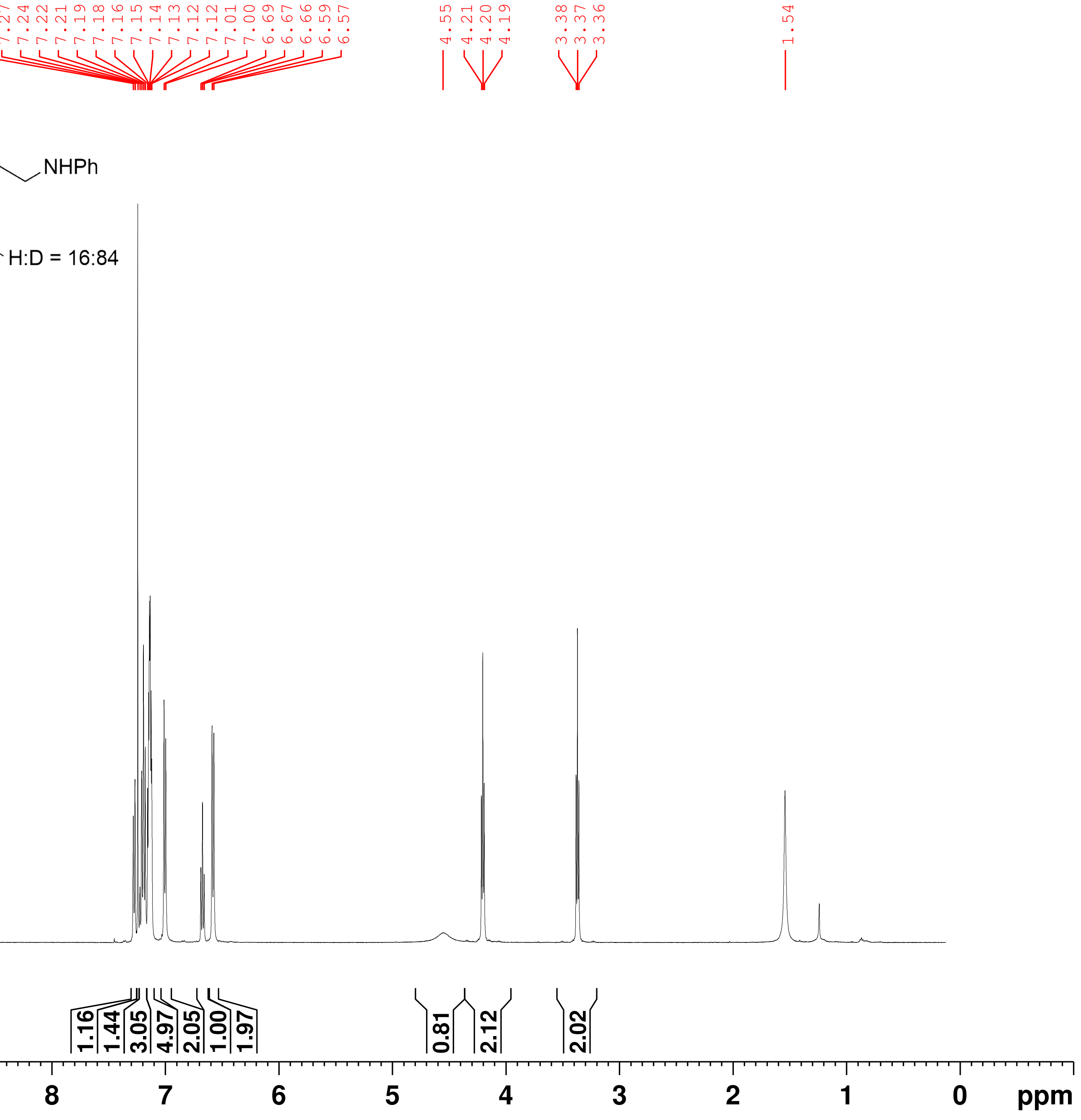

Current Data Parameters
NAME 4a-d
EXPNO 1
PROCNO 1

F2 - Acquisition Parameters
Date_ 20190909
Time 17.27
INSTRUM spect
PROBHD 5 mm PABBO BB-
PULPROG zg30
TD 65536
SOLVENT CDCl3
NS 32
DS 2
SWH 4340.278 Hz
FIDRES 0.066227 Hz
AQ 7.5497470 sec
RG 256
DW 115.200 usec
DE 6.50 usec
TE 297.2 K
D1 1.00000000 sec
TD0 1

======== CHANNEL f1 ========
NUC1 1H
P1 12.00 usec
PL1 -1.10 dB
PL1W 19.41561890 W
SFO1 500.3022533 MHz

F2 - Processing parameters
SI 65536
SF 500.3000217 MHz
WDW EM
SSB 0
LB 0.30 Hz
GB 0
PC 1.00

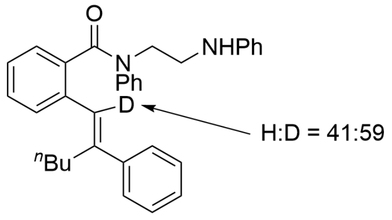

4c-*d*

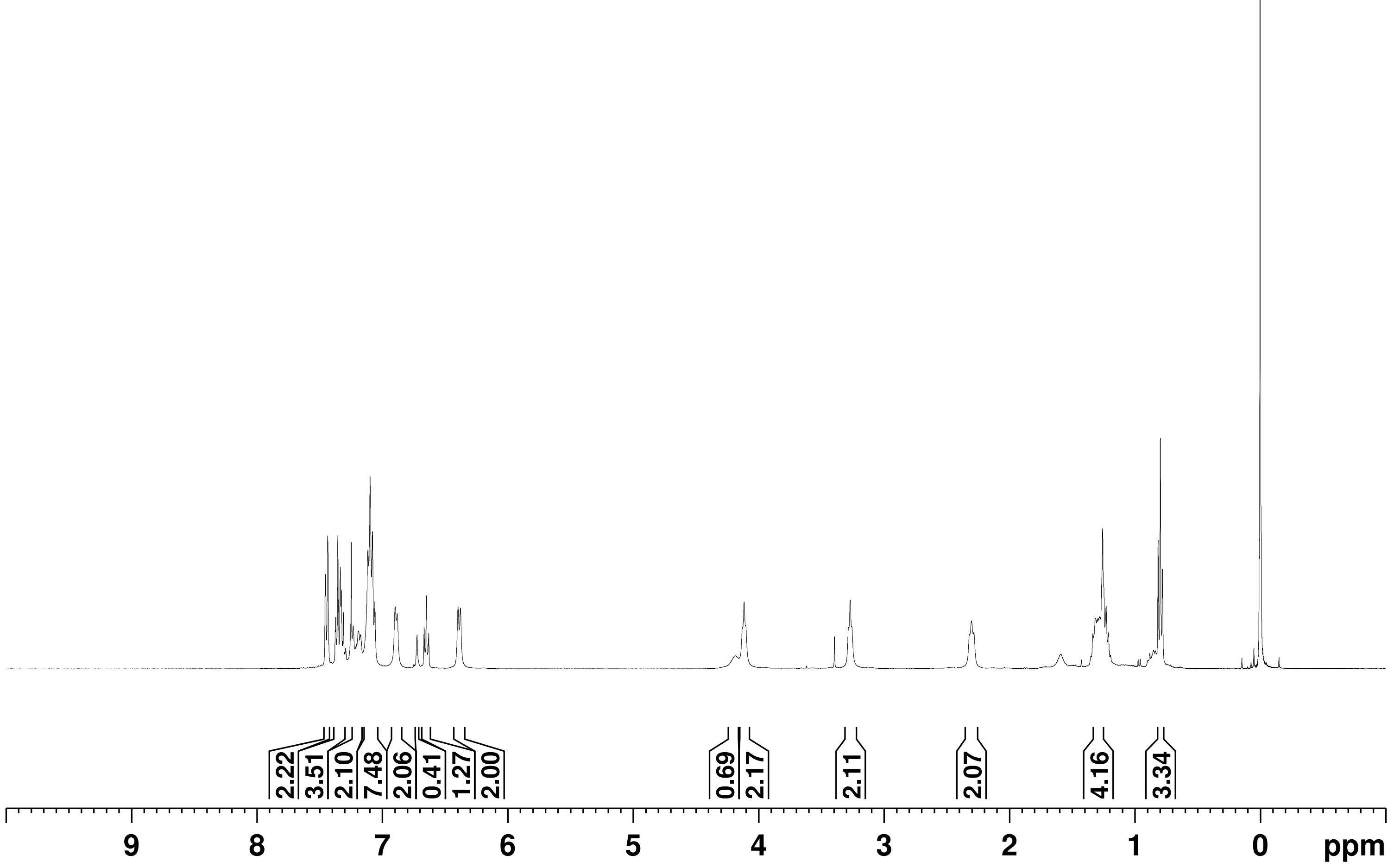


```
Current Data Parameters
NAME                 4c-d
EXPNO                   1
PROCNO                  1

F2 - Acquisition Parameters
Date_            20190916
Time                14.08
INSTRUM             spect
PROBHD   5 mm Multinucl
PULPROG              zg30
TD                  32768
SOLVENT             CDCl3
NS                     32
DS                      0
SWH              8012.820 Hz
FIDRES           0.244532 Hz
AQ              2.0447233 sec
RG                    181
DW                 62.400 usec
DE                   6.50 usec
TE                  298.2 K
D1             0.01000000 sec
TD0                     1

======== CHANNEL f1 ========
NUC1                   1H
P1                   7.20 usec
PL1                 -5.00 dB
SFO1          400.1332010 MHz

F2 - Processing parameters
SI                 131072
SF            400.1300148 MHz
WDW                    EM
SSB       0
LB                   0.25 Hz
GB        0
PC                   0.20
```

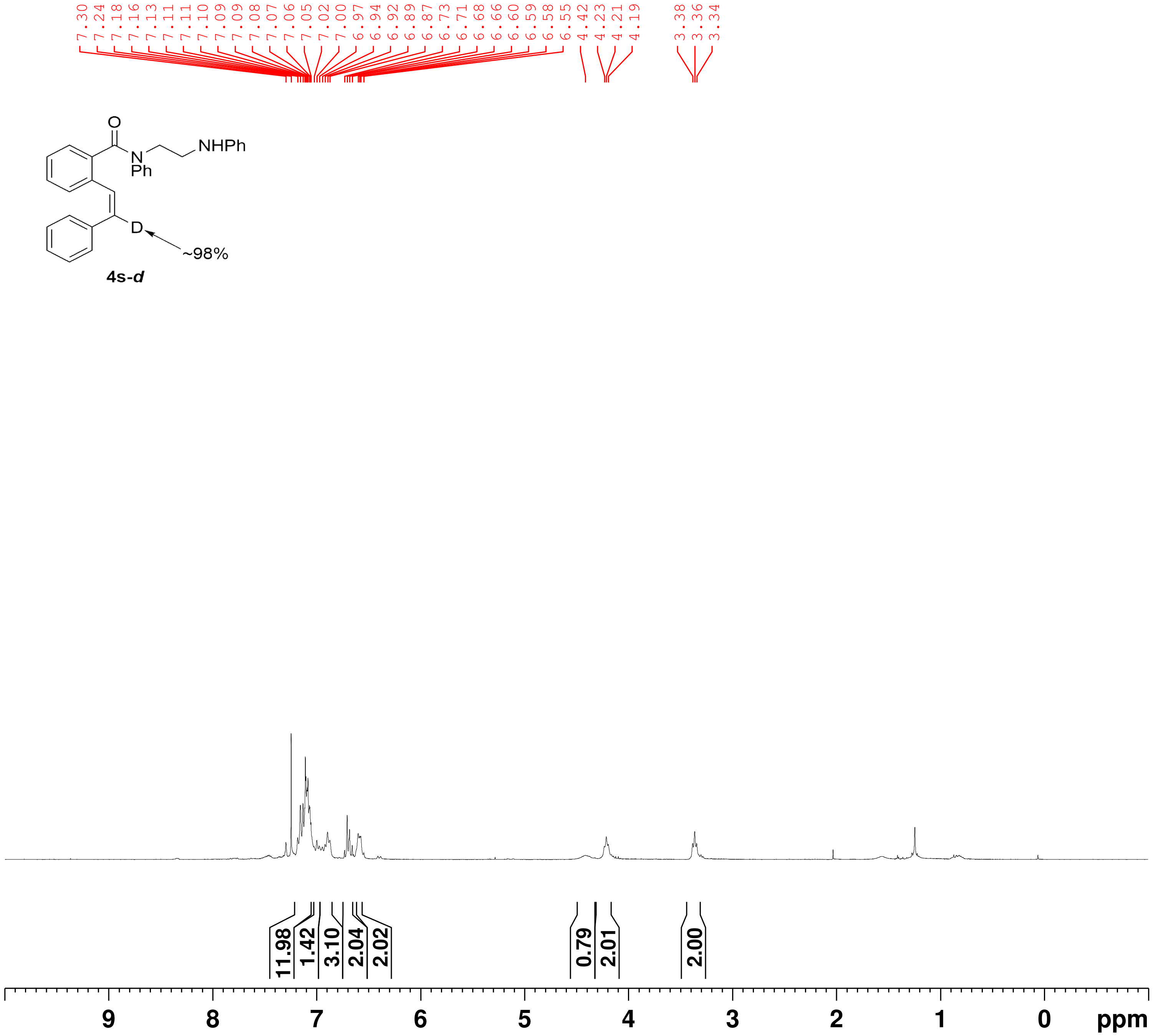

Current Data Parameters

NAME	1H_ST-7-7-1re
EXPNO	3
PROCNO	1

F2 - Acquisition Parameters

Date_	20220203
Time	15.21 h
INSTRUM	Avance NEO nanobay
PROBHD	Z104275_0486 (
PULPROG	zg30
TD	32768
SOLVENT	CDCl3
NS	16
DS	0
SWH	5882.353 Hz
FIDRES	0.359030 Hz
AQ	2.7852800 sec
RG	101
DW	85.000 usec
DE	6.50 usec
TE	298.5 K
D1	1.00000000 sec
TD0	1
SFO1	300.1362005 MHz
NUC1	1H
P0	4.67 usec
P1	14.00 usec
PLW1	6.14340019 W

F2 - Processing parameters

SI	65536
SF	300.1350117 MHz
WDW	EM
SSB	0
LB	0.30 Hz
GB	0
PC	1.00
